# Supplementary material for: Prediction of pregnancy-related hypertensive disorders using metabolomics: a systematic review
Source: BMJ Open. 2022 Apr 25;12(4):e054697. doi: 10.1136/bmjopen-2021-054697 (PMC9039389; doi:10.1136/bmjopen-2021-054697)
Supplement: Supplementary data [file bmjopen-2021-054697supp001.pdf]

Prediction of pregnancy-related hypertensive disorders using metabolomics: a systematic review

Jussara Mayrink, Debora Farias B. Leite, Guilherme M Nóbrega, Maria Laura Costa, José Guilherme Cecatti

Supplementary material 1 – PRISMA checklist

Section/topic	#	Checklist item	Section where the item was reported
**Title**			
Title	1	Identify the report as a systematic review, meta-analysis, or both.	Page 1/title
**ABSTRACT**			
Structured summary	2	Provide a structured summary including, as applicable: background; objectives; data sources; study eligibility criteria, participants, and interventions; study appraisal and synthesis methods; results; limitations; conclusions and implications of key findings; systematic review registration number.	Page 2/abstract
**INTRODUCTION**			
Rationale	3	Describe the rationale for the review in the context of what is already known.	Page 4
Objectives	4	Provide an explicit statement of questions being addressed with reference to participants, interventions, comparisons, outcomes, and study design (PICOS).	Page 5; Lines 11-12
**METHODS**			
Protocol and registration	5	Indicate if a review protocol exists, if and where it can be accessed (e.g., Web address), and, if available, provide registration information including registration number.	Page 3, Line 3<br>Page 5, Line 15
Eligibility criteria	6	Specify study characteristics (e.g., PICOS, length of follow-up) and report characteristics (e.g., years considered, language, publication status) used as criteria for eligibility, giving rationale.	Page 5, Lines 20-28<br>Page 6, Lines 1-13<br>Page 7, Lines 6-13
Information sources	7	Describe all information sources (e.g., databases with dates of coverage, contact with study authors to identify additional studies) in the search and date last searched.	Page 6, Lines 14-23<br>Page 8, Lines 10-11
Search	8	Present full electronic search strategy for at least one database, including any limits used, such that it could be repeated.	Supplementary Material 2
Study selection	9	State the process for selecting studies (i.e., screening, eligibility, included in systematic review, and, if applicable, included in the meta-analysis).	Page 7, Lines 16-27<br>Page 8, Lines 1-7
Data collection process	10	Describe method of data extraction from reports (e.g., piloted forms, independently, in duplicate) and any processes for obtaining and confirming data from investigators.	Page 7, Lines 16-27<br>Page 8, Lines 1-7
Data items	11	List and define all variables for which data were sought (e.g., PICOS, funding sources) and any assumptions and simplifications made.	Page 7, Lines 16-27<br>Page 8, Lines 1-7
Risk of bias in individual studies	12	Describe methods used for assessing risk of bias of individual studies (including specification of whether this was done at the study or outcome level), and how this information is to be used in any data synthesis.	Page 7, Lines 16-20
Summary measures	13	State the principal summary measures (e.g., risk ratio, difference in means).	Page 8, Lines 3-7

Section/topic	#	Checklist item	Section where the item was reported
Synthesis of results	14	Describe the methods of handling data and combining results of studies, if done, including measures of consistency (e.g., I^2) for each meta-analysis.	NA
Risk of bias across studies	15	Specify any assessment of risk of bias that may affect the cumulative evidence (e.g., publication bias, selective reporting within studies).	NA
Additional analyses	16	Describe methods of additional analyses (e.g., sensitivity or subgroup analyses, meta-regression), if done, indicating which were pre-specified.	NA
RESULTS			
Study selection	17	Give numbers of studies screened, assessed for eligibility, and included in the review, with reasons for exclusions at each stage, ideally with a flow diagram.	Page 8, Lines 11-27
Study characteristics	18	For each study, present characteristics for which data were extracted (e.g., study size, PICOS, follow-up period) and provide the citations.	Page 11, Line 11 – Page 15, Line 5
Risk of bias within studies	19	Present data on risk of bias of each study and, if available, any outcome level assessment (see item 12).	Page 15, Lines 6-15
Results of individual studies	20	For all outcomes considered (benefits or harms), present, for each study: (a) simple summary data for each intervention group (b) effect estimates and confidence intervals, ideally with a forest plot.	NA
Synthesis of results	21	Present results of each meta-analysis done, including confidence intervals and measures of consistency.	NA
Risk of bias across studies	22	Present results of any assessment of risk of bias across studies (see Item 15).	NA
Additional analysis	23	Give results of additional analyses, if done (e.g., sensitivity or subgroup analyses, meta-regression [see Item 16]).	NA
**DISCUSSION**			
Summary of evidence	24	Summarize the main findings including the strength of evidence for each main outcome; consider their relevance to key groups (e.g., healthcare providers, users, and policy makers).	Page 16, Line 6 – Page 19, Line 30
Limitations	25	Discuss limitations at study and outcome level (e.g., risk of bias), and at review-level (e.g., incomplete retrieval of identified research, reporting bias).	Page 20, Lines 15-28
Conclusions	26	Provide a general interpretation of the results in the context of other evidence, and implications for future research.	Page 21, Lines 1-20
**FUNDING**			
Funding	27	Describe sources of funding for the systematic review and other support (e.g., supply of data); role of funders for the systematic review.	Page 29, Lines 8-15

Supplementary material 2 – Full search strategy for each database.

PUBMED

(((((((((((preeclampsia) OR (pre-eclampsia)) OR (eclampsia)) OR (gestational hypertension)) OR (white coat hypertension)) OR (severe preeclampsia)) OR (late preeclampsia)) OR (early preeclampsia)) OR (pregnancy-induced hypertension)) OR (hypertensive syndromes of pregnancy)) AND ((((((((((((((metabolomic*) OR (metabonomic*)) OR (metabolit*)) OR (HNMR)) OR (proton NMR)) OR (proton nuclear magnetic resonance)) OR (liquid chromatogra*)) OR (gas chromatogra*)) OR (UPLC)) OR (HPLC)) OR (high pressure liquid chromatograph*)) OR (ultra-performance liquid chromatograph*)) OR (ultra performance liquid chromatograph*)) OR (lipidomic*)) OR (mass spectrometr*))) AND (((screen*) OR (predict*)) OR (profil*)))

EMBASE

('preeclampsia' OR 'pre-eclampsia' OR 'eclampsia' OR 'gestational hypertension' OR 'white coat hypertension' OR 'severe preeclampsia' OR 'late preeclampsia' OR 'early preeclampsia' OR 'pregnancy-induced hypertension' OR 'hypertensive syndromes of pregnancy') AND ('metabolomic*' OR 'metabonomic*' OR 'metabolit*' OR 'HNMR' OR 'proton NMR' OR 'proton nuclear magnetic resonance' OR 'liquid chromatogra*' OR 'gas chromatogra*' OR 'UPLC' OR 'HPLC' OR 'high pressure liquid chromatograph*' OR 'ultra-performance liquid chromatograph*' OR 'ultra performance liquid chromatograph*' OR 'lipidomic*' OR 'mass spectrometr*') AND ('screen*' OR 'predict*' OR 'profil*').

SCOPUS

((TITLE-ABS-KEY (screen*) OR TITLE-ABS-KEY (predict*) OR TITLE-ABS-KEY (profil*))) AND ((TITLE-ABS-KEY (preeclampsia) OR TITLE-ABS-KEY (pre-eclampsia) OR TITLE-ABS-KEY (eclampsia) OR TITLE-ABS-KEY (gestational AND hypertension) OR TITLE-ABS-KEY (white AND coat AND hypertension) OR TITLE-ABS-KEY (severe AND preeclampsia) OR TITLE-ABS-KEY (late AND preeclampsia) OR TITLE-ABS-KEY (early AND preeclampsia) OR TITLE-ABS-KEY (pregnancy- AND induced AND hypertension) OR TITLE-ABS-KEY (hypertensive AND syndromes AND of AND pregnancy))) AND ((TITLE-ABS-KEY (metabolomic) OR TITLE-ABS-KEY (metabonomic*) OR TITLE-ABS-KEY (metabolit*) OR TITLE-ABS-KEY (hnmr) OR TITLE-ABS-KEY (proton AND nmr) OR TITLE-ABS-KEY (proton AND nuclear AND magnetic AND resonance) OR TITLE-ABS-KEY (liquid AND chromatogra*) OR TITLE-ABS-KEY (gas AND chromatogra*) OR TITLE-ABS-KEY (uplc) OR TITLE-ABS-KEY (hplc) OR TITLE-ABS-KEY (high AND pressure AND liquid AND chromatograph*) OR TITLE-ABS-KEY (ultra-performance AND liquid AND chromatograph*) OR TITLE-ABS-KEY (ultra AND performance AND liquid AND chromatograph*) OR TITLE-ABS-KEY (lipidomic*) OR TITLE-ABS-KEY (mass AND spectrometr*)))

Web of Knowledge

#1

((((((((ALL=(preeclampsia)) OR ALL=(pre-eclampsia)) OR ALL=(eclampsia)) OR ALL=(gestational hypertension)) OR ALL=(white coat hypertension)) OR ALL=(severe preeclampsia)) OR ALL=(late preeclampsia)) OR ALL=(early preeclampsia)) OR ALL=(pregnancy- induced hypertension)) OR ALL=(hypertensive syndromes of pregnancy)

#2

((((((((((((((ALL=(metabolomic*)) OR ALL=(metabonomic*)) OR ALL=(metabolit*)) OR ALL=(HNMR)) OR ALL=(proton NMR)) OR ALL=(proton nuclear magnetic resonance)) OR ALL=(liquid chromatogra*)) OR ALL=(gas chromatogra*)) OR ALL=(UPLC)) OR ALL=(HPLC)) OR ALL=(high pressure liquid chromatograph*)) OR ALL=(ultra-performance liquid chromatograph*)) OR ALL=(ultra performance liquid chromatograph*)) OR ALL=(lipidomic*)) OR ALL=(mass spectrometr*)

#3

((ALL=(screen*)) OR ALL=(predict*)) OR ALL=(profil*)

#4

#1 AND #2 AND #3

LILACS

(‘preeclampsia’ OR ‘pre-eclampsia’ OR ‘eclampsia’ OR ‘gestational hypertension’ OR ‘white coat hypertension’ OR ‘severe preeclampsia’ OR ‘late preeclampsia’ OR ‘early preeclampsia’ OR ‘pregnancy-induced hypertension’ OR ‘hypertensive syndromes of pregnancy’) AND (‘metabolomic*’ OR ‘metabonomic*’ OR ‘metabolit*’ OR ‘HNMR’ OR ‘proton NMR’ OR ‘proton nuclear magnetic resonance’ OR ‘liquid chromatogra*’ OR ‘gas chromatogra*’ OR ‘UPLC’ OR ‘HPLC’ OR ‘high pressure liquid chromatograph*’ OR ‘ultra-performance liquid chromatograph*’ OR ‘ultra performance liquid chromatograph*’ OR ‘lipidomic*’ OR ‘mass spectrometr*’) AND (‘screen*’ OR ‘predict*’ OR ‘profil*’)

SciELO

(‘preeclampsia’ OR ‘pre-eclampsia’ OR ‘eclampsia’ OR ‘gestational hypertension’ OR ‘white coat hypertension’ OR ‘severe preeclampsia’ OR ‘late preeclampsia’ OR ‘early preeclampsia’ OR ‘pregnancy-induced hypertension’ OR ‘hypertensive syndromes of pregnancy’) AND (‘metabolomic*’ OR ‘metabonomic*’ OR ‘metabolit*’ OR ‘HNMR’ OR ‘proton NMR’ OR ‘proton nuclear magnetic resonance’ OR ‘liquid chromatogra*’ OR ‘gas chromatogra*’ OR ‘UPLC’ OR ‘HPLC’ OR ‘high pressure liquid chromatograph*’ OR ‘ultra-performance liquid chromatograph*’ OR ‘ultra performance liquid chromatograph*’ OR ‘lipidomic*’ OR ‘mass spectrometr*’) AND (‘screen*’ OR ‘predict*’ OR ‘profil*’)

DARE and HTA

(‘preeclampsia’ OR ‘pre-eclampsia’ OR ‘eclampsia’ OR ‘gestational hypertension’ OR ‘white coat hypertension’ OR ‘severe preeclampsia’ OR ‘late preeclampsia’ OR ‘early preeclampsia’ OR ‘pregnancy-induced hypertension’ OR ‘hypertensive syndromes of pregnancy’) AND (‘metabolomic*’ OR ‘metabonomic*’ OR ‘metabolit*’ OR ‘HNMR’ OR ‘proton NMR’ OR ‘proton nuclear magnetic resonance’ OR ‘liquid chromatogra*’ OR ‘gas chromatogra*’ OR ‘UPLC’ OR ‘HPLC’ OR ‘high pressure liquid chromatograph*’

OR 'ultra-performance liquid chromatograph*' OR 'ultra performance liquid chromatograph*' OR 'lipidomic*' OR 'mass spectrometr*') AND ('screen*' OR 'predict*' OR 'profil*') IN DARE, HTA

Supplementary Material 3 - Excluded studies and reasons for exclusion.

Authors	DOI	Reason for exclusion
Kenny LC et al, 2008	10.1177/1933719108316908	Cross-sectional design.
Aris A et al, 2009	10.1016/j.placenta.2009.01.003	Non metabolomics research.
Kenny LC et al, 2009		Conference abstract. Duplicated data. Please check Kenny LC et al (10.1161/HYPERTENSIONAHA.110.157297)
Baker AM et al, 2010	10.1210/jc.2010-0996	Duplicated data. Please check Woodham PC et al, 2011 (10.1161/HYPERTENSIONAHA.111.179069)
Bahado-Singh RO et al, 2012	10.3109/14767058.2012.680254	Duplicated data. Please check Bahado-Singh RO et al, 2015 (10.1016/j.ajog.2015.06.044)
Austdal M et al, 2013	10.1016/j.preghy.2013.04.030	Cross-sectional design.
Bahado-Singh RO et al, 2013	10.1016/j.ajog.2012.11.003	Duplicated data. Please check Bahado-Singh RO et al, 2017 (10.1080/14767058.2016.1185411)
Senyavina NV et al, 2013	10.1007/s10517-013-2225-y	Cross-sectional design.
Bodnar et al, 2014	10.1097/EDE.0000000000000039	Imputation of data obtained in a small subset to the whole sample.
Austdal M et al, 2014	10.1371/journal.pone.0091923	Duplicated data. Please check Austdal M et al, 2015 (10.3390/ijms160921520)
Austdal M et al, 2015	10.1016/j.placenta.2015.10.019	Cross-sectional design.
Austdal M et al, 2015	10.3390/ijms160921520	Comparison of preeclampsia patients with women with normal blood pressure and gestational hypertension together.
Riley J et al, 2016		Conference abstract. Duplicated data. Please check Odibo A et al 2011 (10.1002/pd.2822)
Chen T et al, 2017	10.1016/j.bbrc.2017.02.032	Cross-sectional design.
Kelly RS et al, 2017	10.1007/s11306-016-1149-8	Randomized clinical trial.
Powell KL et al, 2018	10.1016/j.placenta.2018.04.005	Cross-sectional design.
Naase et al, 2018	10.1093/humrep/33.Supplement_1.1	Only mass spectrometry data.
Kawasaki K et al, 2019	10.1161/HYPERTENSIONAHA.118.12389	Cross-sectional design.
Liu Y et al, 2019	10.1111/1440-1681.13095	Cross-sectional design.
Sander KN et al, 2019	10.1007/s11306-019-1600-8	Cross-sectional design.
Chaudhry SH et al, 2019	10.1186/s12884-019-2219-5	Non metabolomics research.
Lewandowska M et al, 2019	10.3390/nu11051028	No specific data on preeclampsia.

Vieira MC et al, 2019	10.1177/1933719119834079	Randomized clinical trial. Only mass spectrometry data.
Odenkirk MT et al, 2020	10.1039/x0xx00000x	Cross-sectional design.
Ferranti et al, 2020	10.1155/2020/1515321	Mention of altered metabolic pathways, but not metabolites.
Kelly C et al, 2020	10.3390/nu12072048	Non metabolomics research.
Kyozuka H et al, 2020	10.1038/s41598-020-70974-3	No specific data on preeclampsia.
McBride N et al, 2020	10.1186/s12916-020-01819-z	Randomized clinical trial.
Jääskeläinen T et al, 2021	10.1007/s11306-020-01752-5	No specific data on preeclampsia compared to normotensive women.
Rodriguez et al, 2021	10.1016/j.arcmed.2021.01.012	Cross-sectional design.
McBride N et al, 2021	10.3390/ metabo11080530	Mention of accuracy measures of a group of metabolites, and of metabolites combined with other maternal risk factors, but do not mention specific metabolites.
Kivelä J et al, 2021	10.1210/clinem/dgab475	Randomized clinical trial.
Zhang-Rutledge K et al, 2021	10.1016/j.ajog.2020.12.267	Only mass spectrometry data.
Wahab et al, 2022	10.3390/ metabo12010013	No specific data on preeclampsia compared to normotensive women.
Herlambang H et al, 2022	10.1186/s12884-021-04313-3	Cross-sectional design.
Liu H, et al	10.1007/s12011-021-02988-5	Cross-sectional design.

Supplementary Material 4 - Predictive metabolites of hypertensive disorders of pregnancy summarized according to their superclass, chemical class, subclass, and metabolic pathways.

Predictive metabolites	Chemical class	Chemical subclass	Metabolic pathway or process	Involved with
		Organic acids and derivatives		
N1, N12-diacetylspermine	Carboximidic acids and derivatives	Carboximidic acids	NA	Late onset preeclampsia[1]
Acetamide	Carboximidic acids and derivatives	Carboximidic acids	NA	Preeclampsia [2]
Acetate	Carboxylic acids and derivatives	Carboxylic acids	Glycolysis, gluconeogenesis; pyruvate, propanoate, taurine and hypotaurine metabolism	Preeclampsia [2,3]
3-methylhistidine	Carboxylic acids and derivatives	Amino acids, peptides, and analogues	Beta-alanine, histidine and methylhistidine metabolism	Preeclampsia [3]
Methylhistidine	Carboxylic acids and derivatives	Amino acids, peptides, and analogues	Histidine Metabolism	Late onset preeclampsia [4]
Alanine	Carboxylic acids and derivatives	Amino acids, peptides, and analogues	Glutathione metabolism, glycine and serine metabolism, tryptophan metabolism, urea cycle, glutamate metabolism, glucose-alanine cycle	Preeclampsia [5], early onset [5] preeclampsia
Betaine	Carboxylic acids and derivatives	Amino acids, peptides, and analogues	Betaine metabolism, glycine and serine metabolism, methionine metabolism	Preeclampsia [2]
Asymmetric dimethylarginine	Carboxylic acids and derivatives	Amino acids, peptides, and analogues	NA	Preeclampsia [6], early onset [7] preeclampsia
L-arginine	Carboxylic acids and derivatives	Amino acids, peptides, and analogues	Urea cycle, arginine and proline metabolism, glycine and serine metabolism, transcription/translation	Preeclampsia [6], early onset [7] preeclampsia
L-homoarginine	Carboxylic acids and derivatives	Amino acids, peptides, and analogues	NA	Early onset [7] preeclampsia

Creatinine	Carboxylic acids and derivatives	Amino acids, peptides, and analogues	Arginine and proline metabolism	Preeclampsia [2,3]
Glutamine	Carboxylic acids and derivatives	Amino acids, peptides, and analogues	Pyrimidine metabolism, glutamate metabolism, purine metabolism, transcription/translation, urea cycle	Preeclampsia [2,3]
Glutamate	Carboxylic acids and derivatives	Amino acids, peptides, and analogues	Alanine metabolism, histidine metabolism, ammonia recycling, cysteine metabolism, arachidonic acid metabolism	Preeclampsia [5], early onset [5] preeclampsia
4-hydroxyglutamate	Carboxylic acids and derivatives	Amino acids, peptides, and analogues	Arginine and proline metabolism	Preeclampsia [8]
Glycine	Carboxylic acids and derivatives	Amino acids, peptides, and analogues	Ammonia recycling, porphyrin metabolism, glutathione metabolism, glycine and serine metabolism	Preeclampsia [2]
Glycylglycine	Carboxylic acids and derivatives	Amino acids, peptides, and analogues	NA	Late onset [9] preeclampsia
Isoleucine	Carboxylic acids and derivatives	Amino acids, peptides, and analogues	Valine, leucine and isoleucine biosynthesis and degradation	Preeclampsia [2,3]
Asparagine	Carboxylic acids and derivatives	Amino acids, peptides, and analogues	Alanine, aspartate and glutamate metabolism; protein digestion and absorption	Preeclampsia [10]
Methionine	Carboxylic acids and derivatives	Amino acids, peptides, and analogues	Betaine metabolism, glycine and serine metabolism; spermidine and spermine biosynthesis	Preeclampsia [2]
N,N-Dimethylglycine	Carboxylic acids and derivatives	Amino acids, peptides, and analogues	Glycine, serine, and threonine metabolism	Preeclampsia [10]
Ornithine	Carboxylic acids and derivatives	Amino acids, peptides, and analogues	Urea cycle, arginine and proline metabolism; glycine and serine	Preeclampsia [2]

			metabolism; Spermidine and Spermine Biosynthesis	
Phenylacetylglutamine	Carboxylic acids and derivatives	Amino acids, peptides, and analogues	Phenylacetate metabolism, phenylalanine metabolism	Preeclampsia [3]
Phenylalanine	Carboxylic acids and derivatives	Amino acids, peptides, and analogues	Phenylalanine, tyrosine and tryptophan biosynthesis; phenylalanine metabolismo;	Preeclampsia [5], early onset [5] preeclampsia
Proline	Carboxylic acids and derivatives	Amino acids, peptides, and analogues	Arginine and proline metabolism	Preeclampsia [2]
Tyrosine	Carboxylic acids and derivatives	Amino acids, peptides, and analogues	Tyrosine metabolism, phenylalanine and tyrosine metabolism, catecholamine biosynthesis, transcription/translation	Preeclampsia [3]
Valine	Carboxylic acids and derivatives	Amino acids, peptides, and analogues	Valine, leucine and isoleucine biosynthesis and degradation; propanoate metabolism	Preeclampsia [2]
Formate	Carboxylic acids and derivatives	Carboxylic acids	Folate metabolism, pterine biosynthesis	Preeclampsia [3]
Fumarate	Carboxylic acids and derivatives	Dicarboxylic acids and derivatives	Phenylalanine and tyrosine metabolism, arginine and proline metabolism, aspartate metabolism, urea cycle, mitochondrial electron transport chain	Preeclampsia [3]
Succinate	Carboxylic acids and derivatives	Dicarboxylic acids and derivatives	Citrate cycle, oxidative phosphorylation; alanine, aspartate and glutamate metabolism; pyruvate metabolism; cAMP signalling pathway; GABAergic synapse; glucagon signalling pathway	Preeclampsia [3]
Cis-aconitate	Carboxylic acids and derivatives	Tricarboxylic acids and derivatives	Citric acid cycle	Preeclampsia [3]

Citrate	Carboxylic acids and derivatives	Tricarboxylic acids and derivatives	Citric acid cycle, transfer of acetyl groups into mitochondria	Preeclampsia [3], early onset [11] preeclampsia, late onset [4] preeclampsia
2-hydroxybutyrate	Hydroxy acids and derivatives	Alpha hydroxy acids and derivatives	Propanoate metabolism	Early onset [11] preeclampsia
2-ketoglutarate	Keto acids and derivatives	Gamma-keto acids and derivatives	Citric acid cycle, gluconeogenesis, oxidation of branched-chain fatty acids, urea cycle, branched-chain amino acids degradation	Preeclampsia [3]
2-oxovaleric acid	Keto acids and derivatives	Short-chain keto acids and derivatives	NA	Late onset [12] preeclampsia
Acetoacetic acid	Keto acids and derivatives	Short-chain keto acids and derivatives	Ketone body metabolism, fatty acid biosynthesis, branched-chain amino acids degradation, tyrosine metabolism, phenylalanine and tyrosine metabolism	Late onset [12] preeclampsia
Oxo-methylbutanoic acid	Keto acids and derivatives	Short-chain keto acids and derivatives	Cell signaling; valine, leucine and isoleucine Degradation	Late onset [12] preeclampsia
Pyruvate	Keto acids and derivatives	Alpha keto acids and derivatives	Urea Cycle, glucose-alanine cycle, glycine and serine Metabolism, pyruvate metabolism, alanine metabolism	Late onset [13] preeclampsia
Urea	Organic carbonic acids and derivatives	Ureas	Urea cycle; D-arginine and D-ornithine metabolism; arginine and proline metabolism	Late onset [4] preeclampsia
Taurine	Organic sulfonic acids and derivatives	Organosulfonic acids and derivatives	Taurine and hypotaurine metabolism	Early onset [9] preeclampsia
Indoxyl sulphate	Organic sulfuric acids and derivatives	Arylsulfates	NA	Preeclampsia [3]
p-cresol sulphate	Organic sulfuric acids and derivatives	Arylsulfates	NA	Preeclampsia [3]

Lipids and lipid-likes molecules				
3-hydroxyisovalerate	Fatty acyls	Fatty acids and conjugates	Lipid transport, metabolism, and peroxidation; fatty acid metabolism	Early onset [11] preeclampsia
Adipic acid	Fatty acyls	Fatty acids and conjugates	Lipid transport, metabolism, and peroxidation; fatty acid metabolism	Late onset [12] preeclampsia
Docosapentaenoic acid	Fatty acyls	Fatty acids and conjugates	Lipid transport, metabolism, and peroxidation; fatty acid metabolism; cell signalling; alpha linolenic acid and linoleic acid metabolism	Preeclampsia [14]
Docosahexaenoic acid	Fatty acyls	Fatty acids and conjugates	Lipid transport, metabolism, and peroxidation; fatty acid metabolism; cell signalling; alpha linolenic acid and linoleic acid metabolism	Preeclampsia [14], Late onset [12] preeclampsia
Docosatriynoic acid	Fatty acyls	Fatty acids and conjugates	Lipid transport, metabolism, and peroxidation; fatty acid metabolism; cell signalling	Late onset [12] preeclampsia
Eicosatrienoic acid	Fatty acyls	Fatty acids and conjugates	Lipid transport, metabolism, and peroxidation; fatty acid metabolism; cell signalling	Preeclampsia [14]
Eicosatetraenoic acid	Fatty acyls	Fatty acids and conjugates	Lipid transport, metabolism, and peroxidation; fatty acid metabolism; cell signalling; alpha linolenic acid and linoleic acid metabolism.	Preeclampsia [14]
Eicosapentaenoic acid	Fatty acyls	Fatty acids and conjugates	Lipid transport, metabolism, and peroxidation; fatty acid metabolism; cell	Preeclampsia [14]

			signalling; alpha linolenic acid and linoleic acid metabolism.	
Methylglutaric acid	Fatty acyls	Fatty acid and conjugates	Lipid transport, metabolism, and peroxidation; fatty acid metabolism; cell signalling	Late onset [12] preeclampsia
Oleic acid	Fatty acyls	Fatty acids and conjugates	Fatty acid biosynthesis, biosynthesis of unsaturated fatty acids	Late onset [12] preeclampsia
Decanoylcarnitine	Fatty acyls	Fatty acid esters	Lipid transport and peroxidation; fatty acid metabolism; cell signaling	Early onset [15] preeclampsia, late onset [12] preeclampsia
Decenoylcarnitine	Fatty acyls	Fatty acid esters	NA	Early onset [15] preeclampsia, late onset preeclampsia
Lauroylcarnitine	Fatty acyls	Fatty acid esters	Lipid transport, metabolism, and peroxidation; fatty acid metabolism; cell signalling	Early onset [15] preeclampsia
Hexadecenoylcarnitine	Fatty acyls	Fatty acid esters	Lipid transport, metabolism, and peroxidation; fatty acid metabolism; cell signalling	Early onset [15] preeclampsia
Hexanoylcarnitine	Fatty acyls	Fatty acid esters	Lipid transport, metabolism, and peroxidation; fatty acid metabolism; cell signalling	Early onset [15] preeclampsia, late onset [15] preeclampsia
Hydroxyhexanoylcarnitine	Fatty acyls	Fatty acid esters	Lipid transport, metabolism, and peroxidation; fatty acid metabolism; cell signalling	Preeclampsia [5], early onset [5] preeclampsia
Isobutyrylcarnitine	Fatty acyls	Fatty acid esters	Lipid transport, metabolism, and peroxidation; fatty acid metabolism; cell signalling	Early onset [15] preeclampsia
Octanoylcarnitine	Fatty acyls	Fatty acid esters	Mitochondrial beta-oxidation of short chain saturated fatty acids	Early onset [15] preeclampsia, late onset [15] preeclampsia

Octenoylcarnitine	Fatty acyls	Fatty acid esters	Lipid transport, metabolism, and peroxidation; fatty acid metabolism; cell signalling	Early onset [15] preeclampsia, late onset [15] preeclampsia
Oleylcarnitine	Fatty acyls	Fatty acid esters	NA	Late onset [15] preeclampsia
Palmitoylcarnitine	Fatty acyls	Fatty acid esters	Fatty acid metabolism	Late onset [15] preeclampsia
Isoprostane 8-epi-prostaglandin F2α	Fatty acyls	Eicosanoids	Arachidonic acid metabolism	Preeclampsia [16]
(+/-) 5-iPF2α-VI	Fatty acyls	Eicosanoids	Lipid peroxidation	Preeclampsia [14]
(+/-) 5-iPF2α-VI-(d11)	Fatty acyls	Eicosanoids	Lipid peroxidation	Preeclampsia [14]
15-epilipoxin-A4	Fatty acyls	Eicosanoids	Lipid transport, metabolism, and peroxidation; fatty acid metabolism; cell signalling	Preeclampsia [17]
Alfa-linolenic acid	Fatty acyls	Lineolic acids and derivatives	Lipid transport, metabolism, and peroxidation; fatty acid metabolism; cell signalling; alpha linolenic acid and linoleic acid metabolism.	Preeclampsia [14]
Stearidonic acid	Fatty acyls	Lineolic acids and derivatives	Lipid transport, metabolism, and peroxidation; fatty acid metabolism; cell signalling; alpha linolenic acid and linoleic acid metabolism.	Preeclampsia [14]
Di-(octadecadienoyl)-sn-glycerol	Glycerophospholipids	Glycerophosphates	Lipid transport, metabolism, and peroxidation; fatty acid metabolism; cell signalling	Late onset [12] preeclampsia
Heptadecanoyl-2-hydroxy-sn-glycero-3-phosphocholine	Glycerophospholipids	Glycerophosphocholines	Lipid transport, metabolism, and peroxidation; fatty acid metabolism; cell signalling; phospholipid and glycerophospholipid metabolism.	Preeclampsia [18]
Dilinoleoyl-glycerol	Glycerophospholipids	Glycerophosphocholines	NA	Preeclampsia [18]

LysoPE C20:0	Glycerophospholipids	Glycerophosphoethanolamines	Lipid peroxidation and metabolism; glycerophospholipid metabolism; fatty acid metabolism cell signalling.	Preeclampsia [19]
Neomenthol-glucuronide	Prenol lipids	Terpene glycosides	Lipid peroxidation and metabolism; glycerophospholipid metabolism; fatty acid metabolism cell signalling.	Gestational hypertension [10]
Sphinganine 1-phosphate	Sphingolipids	Phosphosphingolipids	Sphingolipid metabolism, neuroactive ligand-receptor interaction	Late onset [12] preeclampsia
Sphingomyelin 16:0	Sphingolipids	Phosphosphingolipids	Lipid transport, metabolism, and peroxidation; fatty acid metabolism; cell signalling; apoptosis	Preeclampsia [20]
Sphingomyelin 18:0	Sphingolipids	Phosphosphingolipids	Phospholipid metabolism; lipid transport, metabolism, and peroxidation; fatty acid metabolism; cell signalling; apoptosis	Preeclampsia [20]
Sphingosine 1-phosphate	Sphingolipids	Phosphosphingolipids	Sphingolipid metabolism; lipid transport, metabolism, and peroxidation; fatty acid metabolism; cell signalling	Late onset [12] preeclampsia
Ceramide 14:0	Sphingolipids	Ceramides	NA	Preeclampsia [20]
Ceramide 24:0	Sphingolipids	Ceramides	NA	Preeclampsia [20]
Ceramide(d18:1/25:0)	Sphingolipids	Ceramides	NA	Preeclampsia [21]
Vitamin D	Steroids and steroids derivatives	Vitamin D and derivatives	Lipid transport, metabolism, and peroxidation; fatty acid metabolism; cell signalling	Preeclampsia [22], late onset [12] preeclampsia, gestational hypertension [23]
Bolasterone	Steroids and steroids derivatives	Androstan steroids	Lipid transport, metabolism, and peroxidation; fatty acid metabolism; cell signalling	Preeclampsia [10]
		Organoheterocyclic compounds		

Caffeine	Imidazopyrimidines	Purines and purine derivatives	Caffeine metabolism	Early onset [24] preeclampsia
Paraxanthine	Imidazopyrimidines	Purines and purine derivatives	Caffeine Metabolism	Early onset [24] preeclampsia
5-Hydroxytryptophan	Indoles and derivatives	Tryptamines and derivatives	Tryptophan metabolism	Late onset [12] preeclampsia
Serotonin	Indoles and derivatives	Tryptamines and derivatives	Tryptophan metabolism; nicotine action pathway	Late onset [4] preeclampsia
Gama-butyrolactone	Lactones	Gamma butyrolactones	NA	Late onset [12] preeclampsia
N-methyl-2-pyridone-5-carboxamide	Pyridines and derivatives	Pyridinecarboxylic acids and derivatives	Nicotinate and Nicotinamide Metabolism	Preeclampsia [3]
N-methylnicotinamide	Pyridines and derivatives	Pyridinecarboxylic acids and derivatives	NA	Preeclampsia [3]
Oxolan-3-one	Dihydrofurans	Furanones	NA	Late onset [12] preeclampsia
		Organic oxygen compounds		
4-deoxyerythronic acid	Organooxygen compounds	Carbohydrates and carbohydrate conjugates	NA	Preeclampsia [3]
Galactose	Organooxygen compounds	Carbohydrates and carbohydrate conjugates	Galactose metabolism, nucleotide sugars metabolismo, lactose degradation	Preeclampsia [3]
Glycerol	Organooxygen compounds	Carbohydrates and carbohydrate conjugates	Glycerolipid metabolism, galactose metabolism, glycerolipid metabolism	Early onset [11] preeclampsia
Lactose	Organooxygen compounds	Carbohydrates and carbohydrate conjugates	Galactose metabolism, Phosphotransferase system, carbohydrate digestion and absorption	Preeclampsia [3]
Sucrose	Organooxygen compounds	Carbohydrates and carbohydrate conjugates	Starch and sucrose metabolism, galactose metabolism	Preeclampsia [3]
Acetone	Organooxygen compounds	Carbonyl compounds	Ketone body metabolism	Early onset [11] preeclampsia, late onset [4] preeclampsia
		Organic nitrogen compounds		
Scyllo-inositol	Organonitrogen compounds	Alcohols and polyols	Inositol phosphate metabolism	Preeclampsia [3]

Propylene glycol	Organooxygen compounds	Alcohols and polyols	Pyruvate metabolism	Late onset [4] preeclampsia
Propane-1,3-diol	Organooxygen compounds	Alcohols and polyols	Glycerolipid metabolism	Preeclampsia [19]
Putrescine	Organonitrogen compounds	Amines	Methionine metabolism, spermidine and spermine biosynthesis	Late onset [4] preeclampsia
Trimethylamine	Organonitrogen compounds	Amines	Methane and carbon metabolisms.	Preeclampsia [2,10]
Carnitine	Organonitrogen compounds	Quaternary ammonium salts	Beta oxidation of very long chain and branched chain fatty acids, mitochondrial beta-oxidation of saturated fatty acids	Preeclampsia [3], late onset [4,13] preeclampsia
Linoleylcarnitine	Organonitrogen compounds	Quaternary ammonium salts	Lipid transport, metabolism, and peroxidation; fatty acid metabolism; cell signalling	Early onset [15] preeclampsia, late onset [15] preeclampsia
Stearoylcarnitine	Organonitrogen compounds	Quaternary ammonium salts	Mitochondrial beta-oxidation of long chain saturated fatty acids	Early onset [15] preeclampsia, late onset [15] preeclampsia
Tetradecenoylcarnitine	Organonitrogen compounds	Quaternary ammonium salts	Lipid transport, metabolism, and peroxidation; fatty acid metabolism; cell signalling	Early onset [15] preeclampsia, late onset [15] preeclampsia
		Benzenoids		
Bisphenol A	Benzene and substituted derivatives	Diphenylmethanes	Bisphenol degradation	Preeclampsia [25]
Hippurate	Benzene and substituted derivatives	Benzoic acids and derivatives	Phenylalanine metabolism	Preeclampsia [3]
4-OH-hippurate	Benzene and substituted derivatives	Benzoic acids and derivatives	NA	Preeclampsia [3]
Mono(2- ethyl-5-oxohexyl) phthalate	Benzene and substituted derivatives	Benzoic acids and derivatives	NA	Late onset [25] preeclampsia

Mono(2-ethyl-5-carboxypentyl) phthalate	Benzene and substituted derivatives	Benzoic acids and derivatives	NA	Late onset [25] preeclampsia
Mono(2-ethylhexyl) phthalate	Benzene and substituted derivatives	Benzoic acids and derivatives	NA	Preeclampsia [25]
Mono(3-carboxypropyl) phthalate	Benzene and substituted derivatives	Benzoic acids and derivatives	NA	Preeclampsia [25]
Mono-ethyl phthalate	Benzene and substituted derivatives	Benzoic acids and derivatives	NA	Preeclampsia [25], late onset [25] preeclampsia
Monoisobutyl phthalate	Benzene and substituted derivatives	Benzoic acids and derivatives	NA	Preeclampsia [25]
Mono-*n*-butyl phthalate	Benzene and substituted derivatives	Benzoic acids and derivatives	NA	Late onset [25] preeclampsia
Σ di(2-ethylhexyl) phthalate	Benzene and substituted derivatives	Benzoic acids and derivatives	NA	Preeclampsia [25], Late onset [25] preeclampsia
2,6-Di-tert-butyl-4-hydroxymethylphenol	Benzene and substituted derivatives	Phenylpropanes	NA	Gestational hypertension [10]
		Organohalogen compounds		
Perfluorohexane sulfonate	Alkyl halides	Alkyl fluorides	NA	Preeclampsia [26]
		Alkaloids and derivatives		
Trigonelline	Alkaloids derived from nicotinic acid	Pyridine alkaloids	Nicotinate and nicotinamide metabolism	Preeclampsia [3]
Pilocarpine	NA	NA	Biosynthesis of alkaloids derived from histidine and purine	Gestational hypertension [10]
		Phenylpropanoids and polyketides		
Cerasinone	Flavonoids	O-methylated flavonoids	NA	Preeclampsia [10]
		Organosulfur compounds		
Dimethyl sulfone	Sulfonyls	Sulfones	NA	Preeclampsia [2]

NA = not available

References

1 Gong S, Sovio U, Aye ILMH, *et al.* Placental polyamine metabolism differs by fetal sex, fetal growth restriction, and preeclampsia. *JCI Insight* 2018;**3**:1–15. doi:10.1172/jci.insight.120723

2 Kenny LC, Broadhurst DI, Hong W, *et al.* NMR Profiling in Early Pregnancy Reveals a Metabolomic Signature the Predicts the Subsequent Development Preeclampsia. *Reprod Sci* 2013;**20**:2641A. doi:10.1177/1933719113482088

3 Diaz SO, Barros AS, Goodfellow BJ, *et al.* Second trimester maternal urine for the diagnosis of trisomy 21 and prediction of poor pregnancy outcomes. *J Proteome Res* 2013;**12**:2946–57. doi:10.1021/pr4002355

4 Bahado-Singh R, Poon LC, Yilmaz A, *et al.* Integrated Proteomic and Metabolomic prediction of Term Preeclampsia. *Sci Rep* 2017;**7**:1–10. doi:10.1038/s41598-017-15882-9

5 Odibo AO, Goetzinger KR, Odibo L, *et al.* First-trimester prediction of preeclampsia using metabolomic biomarkers: a discovery phase study. *Prenat Diagn* 2011;**31**:990–4.

6 Rijvers CAH, Marzano S, Winkens B, *et al.* Early-pregnancy asymmetric dimethylarginine (ADMA) levels in women prone to develop recurrent hypertension. *Pregnancy Hypertens* 2013;**3**:118–23. doi:10.1016/j.preghy.2013.01.001

7 Khalil AA, Tsikas D, Akolekar R, *et al.* Asymmetric dimethylarginine, arginine and homoarginine at 11-13 weeks gestation and preeclampsia: A case-control study. *J Hum Hypertens* 2013;**27**:38–43. doi:10.1038/jhh.2011.109

8 Sovio U, McBride N, Wood AM, *et al.* 4-Hydroxyglutamate is a novel predictor of pre-eclampsia. *Int J Epidemiol* 2020;**49**:301–11. doi:10.1093/ije/dyz098

9 Kuc S, Koster MPH, Pennings JLA, *et al.* Metabolomics profiling for identification of novel potential markers in early prediction of preeclampsia. *PLoS One* 2014;**9**. doi:10.1371/journal.pone.0098540

10 Harville EW, Li Y-YY-Y, Pan K, *et al.* Untargeted analysis of first trimester serum to reveal biomarkers of pregnancy complications: a case-control discovery phase study. *Sci Rep* 2021;**11**:3468. doi:10.1038/s41598-021-82804-1

11 Bahado-Singh RO, Syngelaki A, Akolekar R, *et al.* Validation of metabolomic models for prediction of early-onset preeclampsia. *Am J Obstet Gynecol* 2015;**213**:530.e1-530.e10. doi:10.1016/j.ajog.2015.06.044

12 Kenny LC, Broadhurst DI, Dunn W, *et al.* Robust early pregnancy prediction of later preeclampsia using metabolomic biomarkers. *Hypertension* 2010;**56**:741–9. doi:10.1161/HYPERTENSIONAHA.110.157297

13 Bahado-Singh RO, Syngelaki A, Mandal R, *et al.* Metabolomic determination of pathogenesis of late-onset preeclampsia. *J Matern Neonatal Med* 2017;**30**:658–64. doi:10.1080/14767058.2016.1185411

14 Bilodeau JF, Qin Wei S, Larose J, *et al.* Plasma F2-isoprostane class VI isomers at 12-18 weeks of pregnancy are associated with later occurrence of preeclampsia. *Free Radic Biol Med* 2015;**85**:282–7. doi:10.1016/j.freeradbiomed.2015.05.012

15 Koster MPH, Vreeken RJ, Harms AC, *et al.* First-Trimester serum acylcarnitine levels to predict preeclampsia: A metabolomics approach. *Dis Markers* 2015;**2015**. doi:10.1155/2015/857108

16 Chappell L. A longitudinal study of biochemical variables in women at risk of preeclampsia. *Am J Obstet Gynecol* 2002;**187**:127–36. doi:10.1067/mob.2002.122969

17 Shanmugalingam R, Wang X, Motum P, *et al.* The 15-Epilipoxin-A4 Pathway with Prophylactic Aspirin in PreventingPreeclampsia: A Longitudinal Cohort Study. *J Clin Endocrinol Metab* 2020;**105**. doi:10.1210/clinem/dgaa642

18 Kenny LC, Thomas G, Poston L, *et al.* Prediction of preeclampsia risk in first time pregnant women: Metabolite biomarkers for a clinical test. *PLoS One* 2020;**15**:1–19. doi:10.1371/journal.pone.0244369

19 Lee SM, Kang Y, Lee EM, *et al.* Metabolomic biomarkers in midtrimester maternal plasma can accurately predict the development of preeclampsia. *Sci Rep* 2020;**10**:16142. doi:10.1038/s41598-020-72852-4

20 Dobierzewska A, Soman S, Illanes SE, *et al.* Plasma cross-gestational sphingolipidomic analyses reveal potential first trimester biomarkers of preeclampsia. *PLoS One* 2017;**12**:1–16. doi:10.1371/journal.pone.0175118

21 Huang Q, Hao S, You J, *et al.* Early-pregnancy prediction of risk for pre-eclampsia using maternal blood leptin/ceramide ratio: Discovery and confirmation. *BMJ Open* 2021;**11**. doi:10.1136/bmjopen-2021-050963

22 Woodham PC, Brittain JE, Baker AM, *et al.* Midgestation maternal serum 25-hydroxyvitamin D level and soluble fms-like tyrosine kinase 1/placental growth factor ratio as predictors of severe preeclampsia. *Hypertension* 2011;**58**:1120–5. doi:10.1161/HYPERTENSIONAHA.111.179069

23 Ates S, Sevket O, Ozcan P, *et al.* Vitamin D status in the first-trimester: Effects of vitamin D deficiency on pregnancy outcomes. *Afr Health Sci* 2016;**16**:36–43. doi:10.4314/ahs.v16i1.5

24 Eichelberger KY, Baker AM, Woodham PC, *et al.* Second-trimester maternal serum paraxanthine, CYP1A2 activity, and the risk of severe preeclampsia. *Obstet Gynecol* 2015;**126**:725–30. doi:10.1097/AOG.0000000000001041

25 Cantonwine DE, Meeker JD, Ferguson KK, *et al.* Urinary concentrations of bisphenol A and phthalate metabolites measured during pregnancy and risk of preeclampsia. *Environ Health Perspect* 2016;**124**:1651–5. doi:10.1289/EHP188

26 Rylander L, Lindh CH, Hansson SR, *et al.* Per- and Polyfluoroalkyl Substances in Early Pregnancy and Risk for Preeclampsia: A Case-Control Study in Southern Sweden. *Toxics* 2020;**8**. doi:10.3390/toxics8020043
