# Supplementary material for: Prediction of pregnancy-related hypertensive disorders using metabolomics: a systematic review
Source: BMJ Open. 2022 Apr 25;12(4):e054697. doi: 10.1136/bmjopen-2021-054697 (PMC9039389; doi:10.1136/bmjopen-2021-054697)
Supplement: Supplementary data [file bmjopen-2021-054697supp002.pdf]

**Studies**				
	**Patient selection**		**Index test**	
	Was a consecutive or random sample of patients enrolled?	Did the study avoid inappropriate exclusions?	Were the index test results interpreted without knowledge of the results of the reference standard?	If a threshold was used, was it pre-specified?
Chappell LC et al, 2002	Yes	No	Unclear	Yes
Powe et al, 2010	Yes	Yes	Unclear	Yes
Kenny LC et al, 2010	Yes	Yes	Yes	Yes
Odibo AO et al, 2011	Yes	No	Unclear	Yes
Woodham PC et al, 2011	Yes	Yes	Unclear	Yes
Rijvers CAH et al, 2013	Unclear	Unclear	Unclear	Yes
Khalil AA et al, 2013	Yes	Yes	Yes	Yes
Diaz SO et al, 2013	Yes	Unclear	Unclear	Yes
Kenny LC et al, 2013	Unclear	Unclear	Unclear	Yes
Kuc S et al, 2014	Yes	Yes	Unclear	Yes
Wetta LA et al, 2014	Yes	Yes	Unclear	Yes
Bahado-Singh RO et al, 2015	Yes	Yes	Unclear	Yes
Eichelberger KY et al, 2015	Yes	Yes	Yes	Yes
Koster MPH et al, 2015	Yes	Yes	Unclear	Yes
Bilodeau JF et al, 2015	Yes	Yes	Unclear	Yes
Ates S et al, 2016	Yes	Yes	Unclear	Yes
Cantonwine DE et al, 2016	Yes	Yes	Unclear	Yes
Kiely M et al, 2016	Yes	Yes	Yes	Yes
Bahado-Singh RO et al, 2017	Yes	Yes	Unclear	Yes
Bahado-Singh RO et al, 2017b	Yes	Yes	Unclear	Yes
Dobierzewska A et al, 2017	Yes	Yes	Unclear	Yes
Ye Y et al, 2017	Yes	No	Yes	Yes
Gong S et al, 2018	Yes	Yes	Unclear	Yes
Tamblyn JA et al, 2018	Yes	Yes	Yes	Yes
Sovio U et al, 2020	Yes	Yes	Unclear	Yes
Huo X et al, 2020	Yes	No	Unclear	Yes
Rylander L et al, 2020	Yes	Unclear	Unclear	Yes

Lee SM et al, 2020	Unclear	Unclear	Unclear	Yes
Kenny L et al, 2020	Yes	Yes	Yes	Yes
Shanmugalingam R et al, 2020	Yes	No	Unclear	Yes
Harville EW et al, 2021	Yes	Unclear	Unclear	Yes
Huang Q et al, 2021	Unclear	Yes	Unclear	Yes

Risk of bias				
Reference standard		Flow and timing		
Is the reference standard likely to correctly classify the target condition?	Were the reference standard results interpreted without knowledge of the results of the index test?	Was there an appropriate interval between index test and reference standard?	Did all patients receive the same reference standard?	Were all patients included in the analysis?
Yes	Yes	Yes	Yes	Yes
Yes	Unclear	Yes	Yes	Yes
Yes	Yes	Yes	Yes	Yes
Yes	Unclear	Yes	Yes	Yes
Yes	Unclear	Yes	Yes	Yes
Yes	Unclear	Yes	Yes	Yes
Yes	Unclear	Yes	Yes	Yes
Yes	Unclear	Unclear	Yes	Yes
Yes	Unclear	Yes	Yes	Yes
Yes	Unclear	Yes	Yes	Yes
Yes	Yes	Yes	Yes	Yes
Yes	Unclear	Yes	Yes	Yes
Yes	Unclear	Yes	Yes	Yes
Yes	Unclear	Yes	Yes	Yes
Yes	Unclear	Yes	Yes	Yes
Yes	Unclear	Yes	Yes	Yes
Yes	Unclear	Unclear	Yes	No
Yes	Yes	Yes	Yes	Yes
Yes	Unclear	Yes	Yes	Yes
Yes	Unclear	Yes	Yes	Yes
Yes	Unclear	Yes	Yes	Yes
Yes	Unclear	Yes	Yes	Yes
Yes	Unclear	Yes	Yes	Yes
Yes	Yes	Yes	Yes	Yes
Yes	Unclear	Yes	Yes	Yes
Yes	Unclear	Yes	Yes	Yes
Yes	Unclear	Yes	Yes	No

Yes	Unclear	Yes	Yes	Yes
Yes	Yes	Yes	Yes	Yes
Yes	Unclear	Yes	Yes	No
Unclear	Unclear	Yes	Yes	Yes
Yes	Unclear	Yes	Yes	Yes

**Applicability concerns**		
**Patient selection**	**Index test**	**Reference standard**
Are there concerns that the included patients do not match the review question?	Are there concerns that the index test, its conduct, or interpretation differ from the review question?	Are there concerns that the target condition as defined by the reference standard does not match the review
No	No	No
No	No	No
No	No	No
No	No	No
No	No	No
No	No	No
No	No	No
Yes	No	No
No	No	No
No	No	No
No	No	No
No	No	No
No	No	No
No	No	No
No	No	No
No	No	No
No	Yes	No
No	No	No
No	No	No
No	No	No
No	No	No
No	No	No
No	No	No
No	No	No
No	No	No
Yes	No	No
No	No	No

No	No	No
No	No	No
Yes	No	No
No	No	Yes
No	No	No
