# Supplementary material for: Prediction of pregnancy-related hypertensive disorders using metabolomics: a systematic review
Source: BMJ Open. 2022 Apr 25;12(4):e054697. doi: 10.1136/bmjopen-2021-054697 (PMC9039389; doi:10.1136/bmjopen-2021-054697)
Supplement: Author's manuscript [file bmjopen-2021-054697.draft_revisions.pdf]

BMJ Open

BMJ Open is committed to open peer review. As part of this commitment we make the peer review history of every article we publish publicly available.

When an article is published we post the peer reviewers' comments and the authors' responses online. We also post the versions of the paper that were used during peer review. These are the versions that the peer review comments apply to.

The versions of the paper that follow are the versions that were submitted during the peer review process. They are not the versions of record or the final published versions. They should not be cited or distributed as the published version of this manuscript.

BMJ Open is an open access journal and the full, final, typeset and author-corrected version of record of the manuscript is available on our site with no access controls, subscription charges or pay-per-view fees (http://bmjopen.bmj.com).

If you have any questions on BMJ Open's open peer review process please email info.bmjopen@bmj.com

Prediction of pregnancy-related hypertensive disorders using metabolomics: a systematic review

Journal:	*BMJ Open*
Manuscript ID	bmjopen-2021-054697
Article Type:	Original research
Date Submitted by the Author:	23-Jun-2021
Complete List of Authors:	Mayrink, Jussara; State University of Campinas Faculty of Medical Sciences, Obstetrics and Gynecology<br>Leite, Debora; State University of Campinas Faculty of Medical Sciences, Obstetrics and Gynecology<br>Nobrega, Guilherme; State University of Campinas Faculty of Medical Sciences, Department of Obstetrics and Gynecology<br>Costa, Maria Laura; State University of Campinas Faculty of Medical Sciences, Obstetrics and Gynecology<br>Cecatti, Jose; State University of Campinas Faculty of Medical Sciences, Obstetrics and Gynecology
Keywords:	Hypertension < CARDIOLOGY, Maternal medicine < OBSTETRICS, EPIDEMIOLOGY

SCHOLARONE™
Manuscripts

I, the Submitting Author has the right to grant and does grant on behalf of all authors of the Work (as defined in the below author licence), an exclusive licence and/or a non-exclusive licence for contributions from authors who are: i) UK Crown employees; ii) where BMJ has agreed a CC-BY licence shall apply, and/or iii) in accordance with the terms applicable for US Federal Government officers or employees acting as part of their official duties; on a worldwide, perpetual, irrevocable, royalty-free basis to BMJ Publishing Group Ltd ("BMJ") its licensees and where the relevant Journal is co-owned by BMJ to the co-owners of the Journal, to publish the Work in this journal and any other BMJ products and to exploit all rights, as set out in our licence.

The Submitting Author accepts and understands that any supply made under these terms is made by BMJ to the Submitting Author unless you are acting as an employee on behalf of your employer or a postgraduate student of an affiliated institution which is paying any applicable article publishing charge ("APC") for Open Access articles. Where the Submitting Author wishes to make the Work available on an Open Access basis (and intends to pay the relevant APC), the terms of reuse of such Open Access shall be governed by a Creative Commons licence – details of these licences and which Creative Commons licence will apply to this Work are set out in our licence referred to above.

Other than as permitted in any relevant BMJ Author's Self Archiving Policies, I confirm this Work has not been accepted for publication elsewhere, is not being considered for publication elsewhere and does not duplicate material already published. I confirm all authors consent to publication of this Work and authorise the granting of this licence.

For peer review only

SYSTEMATIC REVIEW

Prediction of pregnancy-related hypertensive disorders using metabolomics: a systematic review

Jussara Mayrink, MD, PhD [1,2] (0000-0001-5957-4365; jussaramayrink@gmail.com)
Debora Farias B. Leite, MD, PhD [1,3] (0000-0001-8839-3934; debora.leite@ufpe.br)
Guilherme Moraes Nobrega, BSc [1] (0000-0001-9406-4256; guigo.nobrega@hotmail.com)
Maria Laura Costa, MD, PhD [1] (0000-0001-8280-3234; mlaura@unicamp.br)
José Guilherme Cecatti, MD, PhD [1] (0000-0003-1285-8445; cecatti@unicamp.br)

[1]Department of Obstetrics and Gynecology, State University of Campinas, Faculty of Medical Sciences, Campinas, Sao Paulo, Brazil.

[2] Department of Gynecology and Obstetrics, Federal University of Minas Gerais, Belo Horizonte, Minas Gerais, Brazil.

[3]Department of Gynecology and Obstetrics, Federal University of Pernambuco, Recife, Pernambuco, Brazil.

Correspondence:
José Guilherme Cecatti
Rua Alexander Fleming, 101 Cidade Universitaria Zeferino Vaz.
13083-889. Campinas, Sao Paulo, Brazil.
Telephone: +55 19-35219482
Email: cecatti@unicamp.br

Word Count:
Abstract: 300 words
Main text: 5,048 words

For peer review only - http://bmjopen.bmj.com/site/about/guidelines.xhtml

Abstract

Objective: To determine metabolomics accuracy in predicting hypertensive disorders in pregnancy.

Design: systematic review on test accuracy.

Data sources and study eligibility criteria: An electronic literature search was performed in June, 2019 and April, 2021. Two researchers independently selected studies published from 1998 to 2021 on metabolomics' techniques applied to predict hypertensive disorders during pregnancy; subsequently, they extracted data and performed quality assessment. Discrepancies were dealt with by a third reviewer. The primary outcome was preeclampsia. Cohort or case-control studies were eligible when maternal samples were taken before the diagnosis of the hypertensive disorder.

Study appraisal and synthesis methods: Data on study design, maternal characteristics, methods of diagnosing hypertension in pregnancy, metabolomics details and metabolites, and accuracy measures were independently extracted by two authors.

Results: Among 4,597 initially identified studies on metabolomics, 57 were read in full text and 31 articles were included. Studies were excluded due to duplicated data, study design or lack of identification of metabolites. Metabolomics was applied mainly in the 2nd trimester; the liquid-chromatography coupled to mass spectrometry was the most common technique. Among the 121 different metabolites found, the majority were involved with ammonia recycling; amino acid metabolism; arachidonic acid metabolism; lipid transport, metabolism, and peroxidation; fatty acid metabolism; cell signalling; galactose metabolism; nucleotide sugars metabolism; lactose degradation; and glycerolipid metabolism. Only citrate was a common metabolite for prediction of early- and late-onset preeclampsia. Vitamin D was the only metabolite in common for preeclampsia and gestational hypertension prediction. The meta-analysis was not performed due to lack of appropriate standardized data.

Conclusions and Implications: Metabolite signature may contribute to further insights into the pathogenesis of preeclampsia and support screening tests. Nevertheless, it is mandatory to validate the method in larger studies with a heterogeneous population to ascertain its use in clinical practice.

PROSPERO registration: CRD42018097409.

For peer review only - http://bmjopen.bmj.com/site/about/guidelines.xhtml

Keywords: preeclampsia, systematic review, hypertension, metabolomics, metabolome, prediction.

Strengths and limitations of this study

- We have performed a comprehensive literature search from the beginning of metabolomic knowledge up to the present.
- we found that metabolites can contribute as a guide of pathways to be explored with the purpose to better treat the disease.
- As a limitation, considering that there was no clear uniformity regarding gestational age or platform for studying preeclampsia, a metanalysis could not be performed.

Original protocol: Mayrink J, Leite DFB, Costa ML, *et al*. Metabolomics for prediction of hypertension in pregnancy: a systematic review and meta-analysis protocol. *BMJ Open* 2020; 10:e040652. doi:10.1136/bmjopen-2020-040652.

For peer review only - http://bmjopen.bmj.com/site/about/guidelines.xhtml

INTRODUCTION

Hypertensive disorders have been reported in association to adverse outcomes in pregnancy since years before Christ. They are a heterogeneous group of conditions that include chronic hypertension, preeclampsia, gestational hypertension and preeclampsia superimposed on chronic hypertension.

Chronic hypertension is the condition of Systolic blood pressure ≥140 mmHg and/or Diastolic blood pressure ≥ 90 mmHg before twenty weeks of gestation or even before pregnancy. [1] Gestational Hypertension is the isolated elevation of pressure after twenty weeks of gestation. It is less damaging than preeclampsia but around 30% of these cases progress with preeclampsia. The most prevalent form - preeclampsia - is a serious potentially-life threatening condition to the mother and the baby, [1] and its high prevalence rate persists along the years, ranging from 3 to 10% of all pregnancies, according to the region studied. Unfortunately, treatment has been the same over decades and relays in the management of clinical features, such as new onset of hypertension and end-organ dysfunction, that characterizes preeclampsia. Outcomes mostly depend upon accurate diagnosis and decision on timing of childbirth since there are exiguous predictive tools available. As a result, the scientific race is on identifying the group of pregnant women at high-risk for the disease, who would be candidates for prophylactic and increased surveillance measures. The increased understanding on pathophysiology would also shed light into possible new therapeutic approaches.

Several biomarkers have been studied as predictive tools for preeclampsia, e.g. soluble fms-like tyrosine kinase-1, soluble endoglin, markers of apoptosis and inflammation, placental protein 13, C-reactive protein, and markers of placental hypoxia and distress. [2] However, none are sufficiently sensitive or specific to predict preeclampsia in advance.

Recently, novel technologies, such as metabolomics, have been applied to predict preeclampsia. Metabolomics is known as the newest member of the "omics" family. Metabolome - a collection of metabolites – is defined and used in research to pursue the phenotypic signature of a disease of interest. Metabolites are low-molecular-

For peer review only - http://bmjopen.bmj.com/site/about/guidelines.xhtml

weight chemicals (<1500 Da) resulting from changes in gene, protein expression [3] and environmental interferences. Metabolomics methods include hydrogen nuclear magnetic resonance spectroscopy (^{1}H-NMR) and gas- and liquid- chromatography-mass spectrometry (GC- and LC-MS, respectively). [3,4] Metabolomics has been applied to improve disease biomarkers and understand the pathogenesis of many conditions, such as cancer and diabetes. [5,6] One could argue its value in evaluating preeclampsia, which has a complex aetiology and is considered a multifactorial disease. Metabolomics, however, a high throughput technique, seems perfectly adequate, since it can simultaneously encompass a wide range of metabolic pathways. The objective of this systematic review was to determine the accuracy of metabolomics in predicting pregnancy-induced hypertensive disorders.

METHODS

This systematic review was conducted based on our previously published protocol [7] (CRD42018097409) and is reported in accordance with the Preferred Reported Items for Systematic Reviews and Meta-Analysis (PRISMA) statement. [8] Our research question was: "what is the performance of metabolomics for predicting gestational hypertensive disorders?"

Population and Interventions

Our domain of interest was any form of hypertensive disorder developed during pregnancy, in single or multiple gestations. Metabolomics was the intervention studied; then, metabolites that differentiate hypertensive from normotensive pregnant women should have been mentioned. Maternal samples should have been drawn during pregnancy and before diagnosis of pregnancy-induced hypertensive disorder. We excluded studies in which blood samples were collected after diagnosis of hypertension was established, or when preeclampsia and gestational hypertension were analysed together.

For peer review only - http://bmjopen.bmj.com/site/about/guidelines.xhtml

Comparison and Outcomes

We included studies in which women with any form of pregnancy-induced hypertension were compared to pregnant women without hypertension. Preeclampsia was the primary outcome, diagnosed by criteria defined by the authors. [9–13]

Secondary outcomes were: a) early-onset preeclampsia, characterized by delivery <34 weeks due to preeclampsia; b) late-onset preeclampsia, requiring delivery ≥34 weeks; c) gestational hypertension, when hypertension is the only clinical finding after 20 weeks gestation; d) preeclampsia superimposed on chronic hypertension; e) white coat hypertension, i.e., normal blood pressure recorded in a 24h ambulatory monitoring; f) masked hypertension, characterized as normal blood pressure in the office or clinic and altered under other circumstances; and g) transient gestational hypertension, higher blood pressure at levels during a certain period that is later normalized after repeated blood pressure readings. [9,14]

Search strategy

Two independent researchers (JM, DFBL) performed an electronic database search in PubMed, EMBASE, Scopus, Web of Knowledge, Latin American and Caribbean Health Sciences Literature (LILACS), Scientific Electronic Library Online (Scielo), Health Technology Assessment (HTA), and Database of Abstracts of Reviews of Effects (DARE), including studies published from 1998 to 2021.

The full EMBASE literature search was, as follows: (‘preeclampsia’ OR ‘pre-eclampsia’ OR ‘eclampsia’ OR ‘gestational hypertension’ OR ‘white coat hypertension’ OR ‘severe preeclampsia’ OR ‘late preeclampsia’ OR ‘early preeclampsia’ OR ‘pregnancy- induced hypertension’ OR ‘hypertensive syndromes of pregnancy’) AND (‘metabolomic*’ OR ‘metabonomic*’ OR ‘metabolit*’ OR ‘HNMR’ OR ‘proton NMR’ OR ‘proton nuclear magnetic resonance’ OR ‘liquid chromatogra*’ OR ‘gas chromatogra*’ OR ‘UPLC’ OR ‘HPLC’ OR ‘high pressure liquid chromatograph*’ OR ‘ultra-performance liquid chromatograph*’ OR ‘ultra performance liquid chromatograph*’ OR ‘lipidomic*’ OR ‘mass spectrometr*’) AND (‘screen*’ OR ‘predict*’ OR ‘profil*’). The grey literature

For peer review only

For peer review only - http://bmjopen.bmj.com/site/about/guidelines.xhtml

(conference abstracts) and the reference list of included studies were searched for additional articles for inclusion.

There were no language restrictions; only cohort and case control studies were included. Letters to the editor, editorials, comments, expert opinions, any type of review, experimental animal studies, case reports, intervention trials, and cross-sectional studies were all excluded. In case of duplicated data, the most recent or more complete published set was included.

Data extraction and quality assessment

Trained reviewers (JM, DFBL) proceeded studies' screening (using EndNote® software), data extraction and synthesis, and risk of bias evaluation (Quality Assessment of Diagnostic Accuracy Studies (QUADAS-2) [15]). Disagreements were solved by a third reviewer (MLC).

Summary of data and meta-analysis

For all included studies, we have extracted data on research characteristics and metabolomics details. If the study comprised training and validation datasets, we have extracted metabolomics data from the validation group of women. When the study has not definitively identified a given metabolite, we have extracted all possible metabolites. We have extracted data of metabolites combined with other biomarkers, when available. We have used the Human Metabolome Database [16], the Kyoto Encyclopaedia of Genes and Genomes [17] and the Lipid Maps [18] for matching each metabolite chemical class and subclass, and metabolic pathways and processes.

When available, data on accuracy measures on metabolomics performance in predicting hypertensive disorders of pregnancy were extracted. We planned to perform the estimation of likelihood ratios and hierarchical summary receiver operator characteristic curve, [19] and the assessment of heterogeneity and publication bias. [20]

Patient and public involvement

This is a systematic review and therefore patient and public involvement are not applicable.

For peer review only - http://bmjopen.bmj.com/site/about/guidelines.xhtml

RESULTS

Main findings

The literature search for this systematic review was performed in June 2019 with a further re-run in April 2021. **Figure 1** summarizes the flow chart of studies' selection and final inclusion. We excluded studies due to duplicated data, study design or lack of identification of metabolites **(Supplementary Material 1)**.

Overall, 31 studies were selected: 24 case control studies [21–44] and seven cohort studies [45–51] (**Table 1**). Studies from the same research project but evaluating different metabolites [40,43] or populations [22,26,27,31,34,36,37] were included. Most studies were developed in Europe [21,23,26,27,29,31,34,36,37,39,40,43,45–49] and North America; [30,32,33,38,41,42,44,51] there was only one research from Latin America, [24] two from Oceania [22,50] and another three from Asia. [25,28,35] Twelve studies have sampled maternal specimens in the 1st trimester, [29,30,32,33,39,40,43,45,47–50] 15 studies have sampled in the 2nd trimester, [21,22,25,27,28,31,34–38,41,42,46,51] three studies have sampled at least two times [23,24,44] in pregnancy, and one study included women in late 3rd trimester [26] (**Figure 2**). Three studies [22,31,36] have performed training and validation with different populations.

Studies have defined preeclampsia as hypertension developed in pregnancy associated with proteinuria [9,11–13] or end-organ disfunction. [10] Although not consistently, chronic hypertension or other illnesses, [22,25,27,31,34–38,42,52] and multiple pregnancies [23,25,28,30,31,34,35,37–39,42,49] were reasons for participants exclusion. When chronic hypertensive women were included, there were women taking aspirin as prophylaxis. [21,50] Then, there are no specific data on superimposed preeclampsia or multiple pregnancies. Similarly, there are no studies on white coat hypertension, masked hypertension, or transient gestational hypertension. Only three studies have brought data on gestational hypertension. [30,35,48] Small for gestational age (SGA) infants were fully excluded [29] or included only in preeclampsia group,

For peer review only - http://bmjopen.bmj.com/site/about/guidelines.xhtml

either because they were excluded from control groups, [21,32] or because this variable was not used as for matching cases and controls. [22,27,34]

In **Table 2**, characteristics of the included studies regarding metabolomics techniques and accuracy measures are shown according to the primary and secondary outcomes. Majority of studies have not presented data according to gestational age of delivery. The most common technique was the LC-MS, [22,24,25,27,29,31–36,38,40–45,48,50,52] followed by H-NMR [23,37,46,47,49] and GC-MS. [21,39] Bahado-Singh et al. [23] (H-NRM followed by LC-MS), Bilodeau et al. [51] (HPLC-MS/MS and GC-FID), Lee et al. [28] (GC-MS and LC-MS), and Harville et al. [30] (LC-MS and NMR) have used two complementary techniques. Most studies analysed blood samples, [21,22,24–26,28–43,45,47–51] two studies analysed urine [44,46] and one study analysed both, urine and blood [27] (**Figure 2**). There were no studies with maternal hair or amniotic fluid. All studies that used H-NMR [23,30,37,46,47,49] and four that applied LC-MS [22,26,30,36] were untargeted evaluations. Ten studies reported lipidomic analysis and/or amino acid analysis. [21,24,28,33,39,40,43,45,50,51] Six studies described vitamin D analysis, all of them using LC-MS [27,32,34,38,41,48]. Five studies reported environmental exposures, focusing on caffeine, [42] bisphenol A, [25] phthalates [44] and per-and polyfluoroalkyl substances (PFAS). [29,35] Two studies have looked for differences in preeclampsia prediction according to foetal sex. [26,44] There were five metabolites in common between preeclampsia and SGA prediction (sphinganine 1-phosphate, sphingosine 1-phosphate, tyrosine, vitamin D, N1, N12-diacetylspermine). [53]

For preeclampsia requiring delivery at any gestational age, the lowest area under the curve (AUC) was for isoprostane 8-epi-prostaglandin F2α (0.55), [21] and the highest (AUC 0.95) was for a model composed by cerasinone, bolasterone, and two unidentified metabolites, presented as retention time and mass (6.30_477.7721m/z, 5.86_313.1070m/z).[30] For early onset preeclampsia, the AUC of untargeted metabolomics in 1st trimester maternal blood varied from 0.673 (UPLC-MS/MS) [36] to 0.835 (H-NMR). [47] For late onset preeclampsia, the AUC of untargeted metabolomics

For peer review only - http://bmjopen.bmj.com/site/about/guidelines.xhtml

in 1st trimester maternal blood was 0.629 (H-NMR); [49] in 2nd trimester, 0.92 (UPLC-MS); [22] and in 3rd trimester, 0.761 (H-NMR and LC-MS/MS). [23]

When analysed together with non-metabolomics biomarkers (**Table 3**), the best performance was achieved by taurine combined with maternal prior risk and mean arterial blood pressure (MAP) (AUC 0.93 in 1st trimester for early onset preeclampsia); [40] followed by 3-hydroxyisovalerate, arginine, glycerol combined with uterine arteries pulsatility index (UtPI) (AUC 0.917 in 1st trimester for early onset preeclampsia) [47]; and vitamin D combined with vascular endothelial growth factor (VEGF) and soluble endoglin: placental growth factor ratio (sFLT-1/PlGF) (AUC 0.851 in 2st trimester for preeclampsia at any gestational age). [38]

All predictive metabolites of hypertensive disorders of pregnancy according to chemical class and subclass, and metabolic pathways possibly involved are presented as **Supplementary Material 2**. Ten metabolites could not be found in the metabolome databases (U4, U10, U14, U35, [46] hexadecenoyleicosatetraenoyl-Sn-glycerol; [22] SM C28:1, SM C30:1, LysoPC C19:0 [28], dibenzylamine [30], perfluoroheptanoic acid [35]). There were 121 different metabolites, from ten different super classes. Majority of metabolites corresponded to amino acids, peptides, and analogues (23 compounds); fatty acid esters (11 compounds); fatty acids and conjugates (10 compounds); and carbohydrates and carbohydrate conjugates (5 compounds). They are mainly involved with ammonia recycling; amino acid metabolism; arachidonic acid metabolism; lipid transport, metabolism, and peroxidation; fatty acid metabolism; cell signalling; galactose metabolism; nucleotide sugars metabolism; lactose degradation; and glycerolipid metabolism. There were 10 benzoic acids and derivatives, but only hippurate has a known pathway. Citrate was found in common for prediction of preeclampsia in any gestational age, [46] early [47] and late-onset [23] disease. Octenoylcarnitine, [43] octanoylcarnitine, [43] acetone, [23,47] linoleylcarnitine, [43] stearoylcarnitine, [43] tetradecenoylcarnitine [43] were predictive for both early and late preeclampsia. Vitamin D was the only metabolite involved simultaneously with preeclampsia, [38] late onset [22] preeclampsia, and gestational hypertension. [48]

For peer review only - http://bmjopen.bmj.com/site/about/guidelines.xhtml

Summary of included studies

The untargeted studies provided a great number of predictive metabolites. Kenny et al. showed a decrease of 13 metabolites (NMR) [37] and an increment of another 40 metabolites (LC-MS) [22] in blood analysis between 14-16 weeks of nulliparous pregnant women exclusively. In a validation phase with a list of 14 different metabolites, the odds ratio of developing preeclampsia, at any gestational age, was 23 (95% CI: 7-73), with AUC 0.92. [22] These same metabolites were validated in a different population, [31] and the dilinoleoyl-glycerol has achieved an AUC 0.70 (0.59-0.82).

Bahado-Singh et al. applied metabolomics to predict early- and late-onset cases. [23,47,49] Using maternal 1st trimester blood (11-13w), those authors reinforced the theory of the multiple phenotypes for preeclampsia. [54,55] 2-hydroxybutyrate was identified as predictor of early-onset preeclampsia, [47] whereas carnitine, pyruvate and acetone resulted in an AUC of 0.629 for late-onset disease. This latter AUC increased to 0.734 by combining metabolites and uterine Doppler velocimetry and maternal weight. [49] Finally, in a two-step evaluation in the 1st and 3rd trimester to predict term preeclampsia (≥37w), a combination of metabolomics and proteomics yielded an AUC of 0.817. [23]

Through an untargeted urinary metabolome analysis, Diaz et al. [46] showed decreased levels of acetate, formate, fumarate (all involved with the Krebs cycle), succinate, and isoleucine; and increased levels of 4-deoxyerythronic acid, a degradation product of 3-hydroxybutyrate [46] between 14-26w. Finally, still working with an untargeted approach, Sovio et al. demonstrated an increase of 4-hydroxyglutamate serum levels among women who developed early-onset preeclampsia, measured at 12w. [36] Its prediction performance was improved by addition of pregnancy-associated plasma protein A (PAPP-A) and PlGF. [36]

Regarding lipidomic or amino-acid studies, Odibo et al. adjusted four metabolites (hydroxyhexanoylcarnitine, phenylalanine, glutamate, alanine) for maternal variables (maternal body mass index, ethnicity, and diabetes) and the AUCs were similar with

For peer review only - http://bmjopen.bmj.com/site/about/guidelines.xhtml

the four-metabolite model (0.82) or when only the three amino acids were used for predicting preeclampsia (0.81). Additionally, the four metabolites have demonstrated equal AUCs for predicting preeclampsia at any gestational age (0.82) or in cases of early onset disease (0.84). [33]

In the 1st trimester, Shanmugalingam et al. [50] showed lower levels of 15-epilipoxin-A4, which is possibly involved with the aspirin mechanism of preeclampsia prophylaxis. Chappell et al. showed an increment in 8-epi-prostaglandin F2α from 20 to 24 weeks in the preeclampsia group. [21] Recently, Bilodeau et al. reinforced that F2-isoprostanes isomers from class VI were increased in the preeclampsia group. [51] The prostaglandins' metabolites are markers of lipid peroxidation, which pointed to an oxidative stress status, but presented modest accuracy. [21,51]

Rijvers et al. [45] and Khalil et al. [39] studied the metabolism of asymmetric dimethylarginine (ADMA), which is considered an important inhibitor of nitric oxide action. Rijvers et al. found an increase of L-arginine/ADMA ratio at 12w in the preeclampsia group, [45] and a consistent decrease in L-arginine between 16w and 20w in the preeclampsia compared to the gestational hypertension group. This can be clinically relevant since L-arginine supplementation is possible. [56] Khalil et al. found lower levels of L-arginine and L-homoarginine at 11-13w in the early-onset preeclampsia, and unchanged levels in the late-onset group, even after the excluding chronic hypertensive women. [39]

Koster et al. [43] showed that stearoylcarnitine was a common biomarker for both early-onset and late-onset preeclampsia. In a model composed of stearoylcarnitine, prior risk and MAP, the detection rate improved by 45% and 21%, for early and late-onset cases of preeclampsia, respectively. Dobierzewska et al. [24] have studied sphingolipids that have been related to trophoblast differentiation and invasion. In the ceramide profile, the Cer 14:0 was decreased in both 1st and 3rd trimesters of preeclampsia group, while Cer 16:0 was significantly increased in cross-gestational preeclampsia plasma samples between 1st and 2nd trimesters. Plasma levels of dihydro-sphingosine-1 decreased from the 1st to the 2nd trimester among the preeclampsia group, and SM 18:0 increased through gestation of these participants. [24] Lee SM et

For peer review only - http://bmjopen.bmj.com/site/about/guidelines.xhtml

al. proposed a model constituted by SM C28:1, SM C30:1, LysoPC C19:0, LysoPE C20:0 and propane-1,3-diol, achieving an AUC of 0.868 for identifying preeclampsia. [28]

Gong et al. [26] analysed serum samples in different moments of pregnancy, but it was at 36w when N1, N12 diacetylspermine were higher in women who later present preeclampsia. [26] This was the only study in common with our previous systematic review about SGA infant's prediction using metabolomics. [53]

Regarding targeted vitamin D analysis, all studies have applied LC-MS. [27,32,34,38,41,48] In general, they found lower serum vitamin D concentrations in women who later developed preeclampsia, both in blood [34] and urine, [27] suggesting that dysregulation of vitamin D metabolism occurs early in pregnancy. Indeed, Woodham et al. have pointed reduced odds of 38% of developing severe preeclampsia for a 10nmol/L increase in 25(OH)D level between 15-20w. [38] However, results did not reach statistical significance when a threshold was applied. In nested case-controls [32,41] or cohort [48] studies there were no differences between vitamin D status among preeclampsia group and controls. In the model proposed by Woodham et al. combining 25OHD, VEGF and sFLT-1/PlGF ratio, the AUC was 0.851, a higher value than either marker alone. [38] Vitamin D3 derivatives were found in the untargeted analysis of Kenny et al., [22] but upregulated in preeclampsia cases. [38]

Two studies have evaluated maternal exposure to bisphenol A and its metabolites. [25,44] The di(2-ethylhexyl) phthalate was increased in all trimesters. [44] Its hazard ratio for development of preeclampsia varied from 1.79 (95% CI 1.30-1.52), with 4-16 weeks, to 2.92 (95% CI 1.61-5.28) with 33-38w. The mono(2-ethyl-5-oxohexyl) phthalate, the mono(2-ethyl-5-carboxypentyl) phthalate and the Σdi(2-ethylhexyl) phthalate were associated to increased hazard ratios for preeclampsia when evaluated in urine [44] from both the 1st and the 2nd half of pregnancy. In maternal serum, [25] free bisphenol was higher in preeclamptic women compared to normotensive controls, even when superimposed preeclampsia cases were excluded. The cut-off of >4,4 µg/L was associated with increased odds of 16.46 (95% CI 5.42-49.95) of developing preeclampsia (adjusted for maternal age, parity, and body mass index). [25]

For peer review only - http://bmjopen.bmj.com/site/about/guidelines.xhtml

In another analysis of environmental factors, Eichelberger et al. found no differences in paraxanthine or caffeine levels, but the paraxanthine/caffeine ratio was lower in preeclampsia (0.23 versus; normotensive women, 0.37, *p* 0.02). [42] This represented a decreased risk of developing preeclampsia with increasing paraxanthine/caffeine ratio (odds ratio 0.53, 95%CI 0.31-0.90). Finally, regarding the perfluoroalkyl substances (PFASs), Rylander et al. [29] and Huo et al. [35] have found no differences between preeclamptic women and normotensive controls in blood samples from the 1st or 2nd trimester, respectively. However, levels of perfluorohexane sulfonate between 0.53-0.78ng/mL were associated to higher odds of preeclampsia (aOR 1.67, 95% CI1.02-2.74); [29] and of perfluoroheptanoic acid, to higher odds of gestational hypertension (aOR 1.38; 95%CI 1.01-1.87).

In the last study to evaluate gestational hypertension, Harville et al. [30] identified higher concentrations of 2,6-Di-tert-butyl-4-hydroxymethylphenol in blood samples between 6-14w. This metabolite is derived from 2,6-Di-tert-butyl-4-methylphenol, a synthetic phenolic antioxidant used widely in foods, polymers, and cosmetics to slow oxidation. It has been linked to induce cellular DNA damage.

Quality assessment and risk of bias

The synthesized data for all included studies is shown on **Figure 3**. Regarding the risk of bias, majority of studies were ranked as low for 'Patient Selection' and 'Flow and Timing' domains. On the contrary, most of studies were classified as unknown risk of bias in 'Index Test' and 'Reference Standard' domains due to the missing information if the diagnosis of preeclampsia (reference standard) and the metabolomics analysis (index test) were interpreted without the knowledge of each other. Regarding applicability, virtually all studies were ranked as low applicability concerns, with minor exceptions. [30,44,46,50,57] (Please, check the **Supplementary Material 3** for individual QUADAS-2 data).

Meta-analysis

We found that a third of included studies were designed with predictive purposes, [21–23,25,33,36,38,47,49] and we extracted accuracy measures when possible (**Tables**

For peer review only - http://bmjopen.bmj.com/site/about/guidelines.xhtml

2 and 3). The other studies have only presented statistical differences between groups. Unfortunately, there were discrepancies regarding type of maternal sample or gestational age at sampling, and metabolomics technique. Then, it was not possible to perform a meta-analysis.

DISCUSSION

Hypertensive disorders constitute one of the leading causes of maternal morbidity and mortality worldwide. Preeclampsia has driven attention from scientists with the purpose of better understand, prevent, treat, and deal with this health, social and economic problem. In this systematic review, we have found that amino acids and analogues were the most common chemical class associated to preeclampsia. Similarly, the main pathways involved in preeclampsia were lipid metabolism, mitochondrial beta-oxidation of short chain saturated fatty acids, and cell signalling (**Figure 4**). Our findings can improve disease understanding and contribute to future studies as a guide of pathways to be explored with the purpose to better treat the disease.

Amino acids and related compounds

Amino acids, peptides, and analogues were the most common chemical subclass. Taurine, glycine and glycylglycine were decreased in women who developed preeclampsia. [37,40] Taurine's function is associated with cytoprotection, involving the placentation process and the spiral artery remodelling. Nevertheless, a reduction in taurine activity may be associated with impaired placentation, which is correlated with the pathophysiology of preeclampsia. [40] Glycylglycine is strongly associated with homocysteine levels and participates in the metabolic pathway that antagonizes homocysteine: lower glycine levels promote higher homocysteine levels. Homocysteine is linked to cardiovascular risk and endothelial dysfunction.

Indeed, a reduction in arginine and homoarginine was observed among preeclampsia patients. [39] These are substrates for oxide nitric synthesis, a potent vasodilator with a major function in endothelial cells. ADMA is an inhibitor of the enzyme responsible for NO production; there is evidence that elevated ADMA levels are associated with

For peer review only - http://bmjopen.bmj.com/site/about/guidelines.xhtml

endothelial dysfunction, and consequently, preeclampsia. [45,58] NO is an important regulator of trophoblast implantation, differentiation, motility, invasion and apoptosis. [39] Decreased levels of arginine and homoarginine in blood samples of preeclampsia patients reinforces the involvement of NO molecule in the aetiology of the disorder. [39,47]

Carnitine is a non-essential amino acid strongly associated with lipid metabolism. Its function is to bind to fatty acid, form acyl-carnitine, and shuttle it to mitochondria, to integrate the mitochondrial oxidative metabolism. [49] The increment of carnitine might be associated to the oxidative stress observed in preeclampsia. [22,23,33,43,46,49]

Lipids

Maternal serum lipids are markedly elevated in healthy pregnancies, probably a hormone- induced increase. [59] Preeclampsia women may have even higher levels. [60] The lipidome – the group of lipid metabolites - contains key mediators of vascular tone (sphingosine), inflammation (prostaglandins), and insulin sensitivity (free fatty acids). [22] Lipids are involved in the aetiology of preeclampsia either indirectly, as a substrate, or directly, as a disease mediator. [60] Lipid peroxides may cause endothelial cell activation and reinforce the action of diabetes *mellitus* and essential hypertension as important risk factors to preeclampsia. [21]

In a healthy pregnancy, the pro-angiogenic biochemical scenario, sphingosine-1-phosphate and its receptors are up-regulated. Ceramide levels are also increased, reflecting the trophoblastic apoptosis process, observed because of normal syncytial fusion of villous trophoblasts. [60] On the other hand, in preeclampsia, low levels of sphingosine-1-phosphate (angiogenic) and high levels of ceramides (pro-apoptotic) were found in serum analyses of 3rd trimester preeclampsia cases. [24] The increase of sphingomyelin SM:18 might serve not only as atherogenic marker of preeclampsia development and progression, but also the marker of cardiovascular complications developed later in women who had before preeclamptic pregnancies. Recent studies have demonstrated that serum elevation of sphingomyelin SM:18 positively correlates

For peer review only - http://bmjopen.bmj.com/site/about/guidelines.xhtml

with parameters of insulin resistance and liver function in obese adults [61] and correlates with markers of NFkB activation and, thus, markers of intracellular inflammation. [61] As ceramide and their synthesizing enzymes (e.g. ceramide synthases) have been shown to be significantly up-regulated in the mice uterus during early gestation, [41,62] the role of Cer 14:0 in the normal human pregnancy and its relationship with preeclampsia etiopathogenesis needs to be further explored.

Preeclampsia has also been correlated to the increment of reactive oxygen species. In this sense, there is a consequent increment of oxidized cholesterol, resulting of phosphocoline exposition to reactive oxygen species. Increased levels of acylcarnitine reflect this fatty acid oxidation impairment. [62]

Glycerophosphocholines resulted from cell lysis, possibly correlated with apoptosis described in preeclampsia. For the propanoate pathway, late-onset preeclampsia showed higher concentrations of L- valine, 2-hydroxybutyric acid, lactic acid, acetone and propan-2-ol. [49] Decreased glycerol level has been regarded as an important biomarker of late-onset preeclampsia and this may be due to its conversion to triglycerides. [54,60]

Vitamin D

The included studies on vitamin D achieved contradictory results. [27,32,34,38,41,48] The heterogeneity of results may be attributable to the measurement of only serum 25(OH)D3 vitamin D, which is considered simplistic. [27] Interestingly, all included studies have considered low risk women and have presented data irrespective of gestational age of preeclampsia diagnosis. The active metabolite - 1,25-dihydroxyvitamin D - is thought to increase vascular endothelial growth factor transcription, [63] which is correlated to placenta functioning and adaptation. Besides, vitamin D deficiency may be associated with an increment of inflammatory cytokine levels, such as TNF-alpha, and to a tendency towards T-cell activity. [64] Thus, its deficiency could potentially contribute to the pathophysiology of preeclampsia: abnormal placental implantation, angiogenesis, excessive inflammation, immune dysfunction. [65] Accuracy performance of vitamin D was increased when combined

For peer review only - http://bmjopen.bmj.com/site/about/guidelines.xhtml

with angiogenic biomarkers (VEGF + sFLT-1/PlGF).[38] Additionally, all targeted studies showed lower levels of vitamin D metabolites, both in serum and urine, while untargeted LC-MS evaluation observed upregulated levels. Then, based on our results, we cannot rule out vitamin D role in predicting preeclampsia in high-risk population or early- or late-onset of disease. It seems there is a place for investigating its role in composing a multifactorial model of prediction.

Environmental exposures

Knowledge of the actual contribution of endocrine disrupting chemicals (EDC), such as BPA, phthalates, and others to preeclampsia is relevant, given that behavioural attitude can change maternal exposure to these risk factors. It is hypothesized that BPA can cross the placenta and induce degeneration and necrosis of placental cells, disturbing angiogenesis, [44] and possibly acting through estrogenic-related receptor *gama.* Phthalates can affect placental gene expression determining decreased placental growth, [61] and may also cause a pro-inflammatory response and increase the oxidative stress. [66] Although their role in preeclampsia remains elusive, [25,29,44,57] we cannot rule out their role before pregnancy: BPA, phthalates and PFASs are widely present in many products of daily life, and usually have half-lives of many years.

Another environmental factor that was pointed in our review was caffeine. Caffeine metabolism occurs in the cytochrome P450 1A2 system, and the primary metabolite of dietary caffeine (80% of caffeine by-product) is the paraxanthine. The activity of this cytochrome can be demonstrated by the ratio of paraxanthine/caffeine. During pregnancy, the primary pathway for all caffeine clearance happens in maternal cytochrome P450 1A2 system, as neither placenta nor the foetus demonstrates this cytochrome activity. [67] The decreased risk of severe preeclampsia with increasing paraxanthine/caffeine ratio suggests that faster caffeine metabolism may be associated with a lower risk of preeclampsia. [42] This is not exactly in accordance with other studies, which indicated a direct influence of caffeine levels on the risk of preeclampsia and failed to perform analysis of caffeine metabolism. [68] On the other hand, caffeine and its metabolites are antagonists of the adenosine A1 receptors, implicated in proximal tubular sodium reabsorption. [69] Although caffeine

For peer review only - http://bmjopen.bmj.com/site/about/guidelines.xhtml

consumption may be associated to elevated blood pressure in the short term (≤3 hours), it does not represent a cardiovascular risk factor in the long term (2 weeks) for non-pregnant adults. [70] Future studies should consider maternal smoking as potential bias since cigarette use induces the P450 1A2 system. [71]

Methodological quality

Although majority of included studies were ranked as low risk of bias and low applicability concerns, there is still place for improving data reporting in metabolomics. [72] Moving data from bench to bedside implies the clear description of reference standards and index tests, and timing of index test analysis. As preeclampsia has a complex aetiology and is a multifactorial disease, improving ‘Patient Selection’ should be the focus in the next studies. We could have better accuracy if we separate, for example, data from low- and high-risk women, or single versus multiple gestation.

Strengths and limitations

This systematic review is worthwhile as a guidance of metabolites and their performance as predictors of preeclampsia. We have applied a comprehensive search strategy in eight different electronic databases. Then, we presented possible biomarkers of preeclampsia that could ground future research in this area.

Unfortunately, there are limitations. The inclusion of case-control studies could add risk of bias, confounding and potential measurement error. However, we understand this is an important strategy to Biomarkers Selection and Performance Evaluation steps in metabolomics [4] in perinatal research. There was great heterogeneity among included studies. *Per se*, metabolomics is an overly complex process, influenced by sample collection, storage and preparation, analytical platform applied, and statistical tests performed. [4] Thus, results presented in the studies must consider all these variables. Exactly because of this complexity and authentic heterogeneity of metabolomics, the lack of standardization of preeclampsia concept (early and late-onset preeclampsia) and time of sample collection represented a limitation to our study. Thus, the results could not be correlated with this important variable.

For peer review only - http://bmjopen.bmj.com/site/about/guidelines.xhtml

CONCLUSION AND IMPLICATIONS FOR PRACTICE

We have presented a list of 121 different metabolites and their accuracy measures, when possible. They are mainly involved with ammonia recycling; amino acid and lipids metabolism; arachidonic acid metabolism; cell signalling; galactose, lactose, and nucleotide sugars metabolisms. Maternal blood seems to be the best specimen to predict early preeclampsia if sampled in the 1st trimester, and to predict late-onset preeclampsia in the 2nd trimester. Future metabolomics platforms should be as comprehensive as possible to evaluate the contributions of amino acids, peptides, fatty acid esters, and carbohydrates conjugates, as preeclampsia biomarkers.

Although individual metabolites have low predictive measures, they should be considered in multifactorial models of metabolites alone or in combination with other biomarkers. [23,31,36,38,40,43,47,49] Metabolite signature may contribute to further insights into the pathogenesis of preeclampsia and to design screening tests. In future, it is important to validate citrate and carnitines as common markers between early-onset and late-onset preeclampsia, considering the feasibility of universal screening. [23,46,47] Additionally, since preeclampsia and SGA are related to abnormal deep placentation, [73] it seems plausible to study both conditions simultaneously, at least considering the metabolites found in common (sphinganine 1-phosphate, sphingosine 1-phosphate, tyrosine, vitamin D, N1, N12-diacetylspermine). [53]

For peer review only

ACKNOWLEDGEMENTS

We are grateful to all members of the PRETERM-SAMBA study research group.

AUTHORS CONTRIBUTIONS

JM is the guarantor of this review. JM and DFBL elaborated the protocol, searched the literature, selected studies, extracted data, assessed risk of bias, and drafted the initial manuscript. MLC has helped in study inclusion, interpreting data, examining clinical applications of findings, and revising the manuscript. GN has helped in figures and tables elaboration and with the final version of the manuscript. JGC have supervised and approved all steps. All authors have read and agree with this submission.

FUNDING

This research is part of the PRETERM-SAMBA study, which has granted sponsor from Brazilian National Research Council (CNPq) (Award 401636/2013-5) and from the Bill and Melinda Gates Foundation (grant OPP1107597). Our sponsors have not intervened in any step of this study design, development, or submission.

COMPETING INTERESTS

None to declare.

PATIENT CONSENT FOR PUBLICATION

Not required.

ETHICS APPROVAL STATEMENT

This is a systematic review where ethical approval does not apply.

DATA SHARING STATEMENT

No data are available.

For peer review only

For peer review only - http://bmjopen.bmj.com/site/about/guidelines.xhtml

References

1 L. C. *Chesley's Hypertensive Disorders in Pregnancy*. 4th ed. Elsevier 2015.

2 Laganà AS, Favilli A, Triolo O, *et al.* Early serum markers of pre-eclampsia: are we stepping forward? *J Matern Fetal Neonatal Med* 2016;**29**:3019–23.

3 Nobakht M. Gh BF. Application of metabolomics to preeclampsia diagnosis. *Syst Biol Reprod Med* 2018;**64**:324–39. doi:10.1080/19396368.2018.1482968

4 Xia J, Broadhurst DI, Wilson M, *et al.* Translational biomarker discovery in clinical metabolomics: An introductory tutorial. *Metabolomics* 2013;**9**:280–99. doi:10.1007/s11306-012-0482-9

5 Guasch-Ferré M, Hruby A, Toledo E, *et al.* Metabolomics in prediabetes and diabetes: A systematic review and meta-analysis. *Diabetes Care* 2016;**39**:833–46. doi:10.2337/dc15-2251

6 Hoang G, Udupa S, Le A. *Application of metabolomics technologies toward cancer prognosis and therapy*. 1st ed. Elsevier Inc. 2019. doi:10.1016/bs.ircmb.2019.07.003

7 Mayrink J, Leite DFB, Costa ML, *et al.* Metabolomics for prediction of hypertension in pregnancy: A systematic review and meta-analysis protocol. *BMJ Open* 2020;**10**:1–5. doi:10.1136/bmjopen-2020-040652

8 Moher D, Liberati A, Tetzlaff J, *et al.* Preferred Reporting Items for Systematic Reviews and Meta-Analyses: the PRISMA statement. *Ann Intern Med* 2009;**151**:264–9. doi:10.1371/journal.pmed1000097

9 Tranquilli AL, Dekker G, Magee L, *et al.* The classification, diagnosis and management of the hypertensive disorders of pregnancy: A revised statement from the ISSHP. *Pregnancy Hypertens* 2014;**4**:97–104. doi:10.1016/j.preghy.2014.02.001

10 Brown MA, Hague WM, Higgins J, *et al.* The detection, investigation and management of hypertension in pregnancy: Executive summary. *Aust New Zeal J Obstet Gynaecol* 2000;**40**:133–8. doi:10.1111/j.1479-828X.2000.tb01136.x

11 ACOG Committee on Practice Bulletins--Obstetrics. ACOG practice bulletin. Diagnosis and management of preeclampsia and eclampsia. Number 33, January 2002. *Obstet Gynecol* 2002;**99**:159–67. doi:10.1097/00006250-200201000-00028

12 ACOG. Hypertension in Pregnancy. *Obstet Gynecol* 2013;**122**:1122–31. doi:10.1097/01.AOG.0000437382.03963.88

13 Report of the National High Blood Pressure Education Program Working Group on High Blood Pressure in Pregnancy. *Am J Obstet Gynecol* 2000;**183**:S1–22. doi:10.1067/mob.2000.107928

14 Geraldo J, Ramos L, Sass N, *et al.* Preeclampsia: Definitions of Hypertensive States during Pregnancy Pathophysiological Foundations. *Rev Bras Ginecol Obs* 2017;**39**:496–512.https://pubmed.ncbi.nlm.nih.gov/28793357/

15 Whiting PF, Rutjes AWS, Westwood ME, *et al.* Research and Reporting Methods Accuracy Studies. *Ann Intern Med* 2011;**155**:529–36. doi:10.7326/0003-4819-155-8-201110180-00009

For peer review only

For peer review only - http://bmjopen.bmj.com/site/about/guidelines.xhtml

16 Wishart DS, Feunang YD, Marcu A, *et al.* HMDB 4.0: The human metabolome database for 2018. *Nucleic Acids Res* 2018;**46**:D608–17. doi:10.1093/nar/gkx1089

17 Kanehisa M, Goto S. Kyoto Encyclopedia of Genes and Genomes. *Nucleic Acids Res* 2000;**1**:27–30.https://www.genome.jp/kegg/ (accessed 20 Jun 2021).

18 Fahy E, Sud M, Cotter D, *et al.* LIPID MAPS online tools for lipid research. *Nucleic Acids Res* 2007;**35**:606–12. doi:10.1093/nar/gkm324

19 Rutter CM, Gatsonis CA. A hierarchical regression approach to meta-analysis of diagnostic test accuracy evaluations. *Stat Med* 2001;**20**:2865–84. doi:10.1002/sim.942

20 Deeks JJ, Macaskill P, Irwig L. The performance of tests of publication bias and other sample size effects in systematic reviews of diagnostic test accuracy was assessed. *J Clin Epidemiol* 2005;**58**:882–93. doi:10.1016/j.jclinepi.2005.01.016

21 Chappell L. A longitudinal study of biochemical variables in women at risk of preeclampsia. *Am J Obstet Gynecol* 2002;**187**:127–36. doi:10.1067/mob.2002.122969

22 Kenny LC, Broadhurst DI, Dunn W, *et al.* Robust early pregnancy prediction of later preeclampsia using metabolomic biomarkers. *Hypertension* 2010;**56**:741–9. doi:10.1161/HYPERTENSIONAHA.110.157297

23 Bahado-Singh R, Poon LC, Yilmaz A, *et al.* Integrated Proteomic and Metabolomic prediction of Term Preeclampsia. *Sci Rep* 2017;**7**:1–10. doi:10.1038/s41598-017-15882-9

24 Dobierzewska A, Soman S, Illanes SE, *et al.* Plasma cross-gestational sphingolipidomic analyses reveal potential first trimester biomarkers of preeclampsia. *PLoS One* 2017;**12**:1–16. doi:10.1371/journal.pone.0175118

25 Ye Y, Zhou Q, Feng L, *et al.* Maternal serum bisphenol A levels and risk of pre-eclampsia: A nested case-control study. *Eur J Public Health* 2017;**27**:1102–7. doi:10.1093/eurpub/ckx148

26 Gong S, Sovio U, Aye IL, *et al.* Placental polyamine metabolism differs by fetal sex, fetal growth restriction, and preeclampsia. *JCI insight* 2018;**3**:1–15. doi:10.1172/jci.insight.120723

27 Tamblyn JA, Jenkinson C, Larner DP, *et al.* Serum and urine vitamin D metabolite analysis in early preeclampsia. *Endocr Connect* 2018;**7**:199–210. doi:10.1530/EC-17-0308

28 Lee SM, Kang Y, Lee EM, *et al.* Metabolomic biomarkers in midtrimester maternal plasma can accurately predict the development of preeclampsia. *Sci Rep* 2020;**10**:16142. doi:10.1038/s41598-020-72852-4

29 Rylander L, Lindh CH, Hansson SR, *et al.* Per- and Polyfluoroalkyl Substances in Early Pregnancy and Risk for Preeclampsia: A Case-Control Study in Southern Sweden. *Toxics* 2020;**8**. doi:10.3390/toxics8020043

30 Harville EW, Li Y-YY-Y, Pan K, *et al.* Untargeted analysis of first trimester serum to reveal biomarkers of pregnancy complications: a case-control discovery phase study. *Sci Rep* 2021;**11**:3468. doi:10.1038/s41598-021-82804-1

For peer review only - http://bmjopen.bmj.com/site/about/guidelines.xhtml

31 Kenny LC, Thomas G, Poston L, *et al.* Prediction of preeclampsia risk in first time pregnant women: Metabolite biomarkers for a clinical test. *PLoS One* 2020;**15**:1–19. doi:10.1371/journal.pone.0244369

32 Powe CE, Seely EW, Rana S, *et al.* First trimester vitamin D, vitamin D binding protein, and subsequent preeclampsia. *Hypertension* 2010;**56**:758–63. doi:10.1161/HYPERTENSIONAHA.110.158238

33 Odibo AO, Goetzinger KR, Odibo L, *et al.* First-trimester prediction of preeclampsia using metabolomic biomarkers: a discovery phase study. *Prenat Diagn* 2011;**31**:990–4.

34 Kiely ME, Zhang JY, Kinsella M, *et al.* Vitamin D status is associated with uteroplacental dysfunction indicated by pre-eclampsia and small-for-gestational-age birth in a large prospective pregnancy cohort in Ireland with low Vitamin D status. *Am J Clin Nutr* 2016;**104**:354–61. doi:10.3945/ajcn.116.130419

35 Huo X, Huang R, Gan Y, *et al.* Perfluoroalkyl substances in early pregnancy and risk of hypertensivedisorders of pregnancy: A prospective cohort study. *Environ Int* 2020;**138**. doi:10.1016/j.envint.2020.105656

36 Sovio U, McBride N, Wood AM, *et al.* 4-Hydroxyglutamate is a novel predictor of pre-eclampsia. *Int J Epidemiol* 2020;**49**:301–11. doi:10.1093/ije/dyz098

37 Kenny LC, Broadhurst DI, Hong W, *et al.* NMR Profiling in Early Pregnancy Reveals a Metabolomic Signature the Predicts the Subsequent Development Preeclampsia. *Reprod Sci* 2013;**20**:2641A. doi:10.1177/1933719113482088

38 Woodham PC, Brittain JE, Baker AM, *et al.* Midgestation maternal serum 25-hydroxyvitamin D level and soluble fms-like tyrosine kinase 1/placental growth factor ratio as predictors of severe preeclampsia. *Hypertension* 2011;**58**:1120–5. doi:10.1161/HYPERTENSIONAHA.111.179069

39 Khalil AA, Tsikas D, Akolekar R, *et al.* Asymmetric dimethylarginine, arginine and homoarginine at 11-13 weeks gestation and preeclampsia: A case-control study. *J Hum Hypertens* 2013;**27**:38–43. doi:10.1038/jhh.2011.109

40 Kuc S, Koster MPH, Pennings JLA, *et al.* Metabolomics profiling for identification of novel potential markers in early prediction of preeclampsia. *PLoS One* 2014;**9**. doi:10.1371/journal.pone.0098540

41 Wetta LA, Biggio JR, Cliver S, *et al.* Is midtrimester vitamin D status associated with spontaneous preterm birth and preeclampsia? *Am J Perinatol* 2014;**31**:541–6. doi:10.1055/s-0033-1356483

42 Eichelberger KY, Baker AM, Woodham PC, *et al.* Second-trimester maternal serum paraxanthine, CYP1A2 activity, and the risk of severe preeclampsia. *Obstet Gynecol* 2015;**126**:725–30. doi:10.1097/AOG.0000000000001041

43 Koster MPH, Vreeken RJ, Harms AC, *et al.* First-Trimester serum acylcarnitine levels to predict preeclampsia: A metabolomics approach. *Dis Markers* 2015;**2015**. doi:10.1155/2015/857108

44 Cantonwine DE, Meeker JD, Ferguson KK, *et al.* Urinary concentrations of bisphenol A and phthalate metabolites measured during pregnancy and risk of preeclampsia. *Environ Health Perspect* 2016;**124**:1651–5. doi:10.1289/EHP188

For peer review only

For peer review only - http://bmjopen.bmj.com/site/about/guidelines.xhtml

45 Rijvers CAH, Marzano S, Winkens B, *et al.* Early-pregnancy asymmetric dimethylarginine (ADMA) levels in women prone to develop recurrent hypertension. *Pregnancy Hypertens* 2013;**3**:118–23. doi:10.1016/j.preghy.2013.01.001

46 Diaz SO, Barros AS, Goodfellow BJ, *et al.* Second trimester maternal urine for the diagnosis of trisomy 21 and prediction of poor pregnancy outcomes. *J Proteome Res* 2013;**12**:2946–57. doi:10.1021/pr4002355

47 Bahado-Singh RO, Syngelaki A, Akolekar R, *et al.* Validation of metabolomic models for prediction of early-onset preeclampsia. *Am J Obstet Gynecol* 2015;**213**:530.e1-530.e10. doi:10.1016/j.ajog.2015.06.044

48 Ates S, Sevket O, Ozcan P, *et al.* Vitamin D status in the first-trimester: Effects of vitamin D deficiency on pregnancy outcomes. *Afr Health Sci* 2016;**16**:36–43. doi:10.4314/ahs.v16i1.5

49 Bahado-Singh RO, Syngelaki A, Mandal R, *et al.* Metabolomic determination of pathogenesis of late-onset preeclampsia. *J Matern Neonatal Med* 2017;**30**:658–64. doi:10.1080/14767058.2016.1185411

50 Shanmugalingam R, Wang X, Motum P, *et al.* The 15-Epilipoxin-A4 Pathway with Prophylactic Aspirin in PreventingPreeclampsia: A Longitudinal Cohort Study. *J Clin Endocrinol Metab* 2020;**105**. doi:10.1210/clinem/dgaa642

51 Bilodeau JF, Qin Wei S, Larose J, *et al.* Plasma F2-isoprostane class VI isomers at 12-18 weeks of pregnancy are associated with later occurrence of preeclampsia. *Free Radic Biol Med* 2015;**85**:282–7. doi:10.1016/j.freeradbiomed.2015.05.012

52 Gong S, Sovio U, Aye ILMH, *et al.* Placental polyamine metabolism differs by fetal sex, fetal growth restriction, and preeclampsia. *JCI Insight* 2018;**3**:1–15. doi:10.1172/jci.insight.120723

53 Leite DFB, Morillon A-C, Junior EF de M, *et al.* Examining the predictive accuracy of metabolomics for small for gestational age babies: a systematic review. *BMJ Open* 2019;**9**:e031238. doi:10.1136/ bmjopen-2019-031238

54 Bahado-Singh RO, Akolekar R, Mandal R, *et al.* First-trimester metabolomic detection of late-onset preeclampsia. *Am J Obstet Gynecol* 2013;**208**:58.e1-58.e7. doi:10.1016/j.ajog.2012.11.003

55 Nelson DB, Ziadie MS, McIntire DD, *et al.* Placental pathology suggesting that preeclampsia is more than one disease. *Am J Obstet Gynecol* 2014;**210**:66.e1-66.e7. doi:10.1016/j.ajog.2013.09.010

56 Khalaf D, Krüger M, Wehland M, *et al.* The effects of oral l-arginine and l-citrulline supplementation on blood pressure. *Nutrients* 2019;**11**:1–14. doi:10.3390/nu11071679

57 Huo X, Huang R, Gan Y, *et al.* Perfluoroalkyl substances in early pregnancy and risk of hypertensive disorders of pregnancy: A prospective cohort study. *Environ Int* 2020;**138**:1–7. doi:10.1016/j.envint.2020.105656

58 Levine RJ, Lam C, Qian C, *et al.* Soluble Endoglin and Other Circulating Antiangiogenic Factors in Preeclampsia. *N Engl J Med* 2006;**355**:992–1005.

59 Wang C, Kong L, Yang Y, *et al.* Recommended reference values for serum lipids during

For peer review only

For peer review only - http://bmjopen.bmj.com/site/about/guidelines.xhtml

early and middle pregnancy: A retrospective study from China 11 Medical and Health Sciences 1114 Paediatrics and Reproductive Medicine. *Lipids Health Dis* 2018;**17**:1–16. doi:10.1186/s12944-018-0885-3

60 Wojcik-Baszko D, Charkiewicz K, Laudanski P. Role of dyslipidemia in preeclampsia—A review of lipidomic analysis of blood, placenta, syncytiotrophoblast microvesicles and umbilical cord artery from women with preeclampsia. *Prostaglandins Other Lipid Mediat* 2018;**139**:19–23. doi:10.1016/j.prostaglandins.2018.09.006

61 Aris A, Benali S, Ouellet A, *et al.* Potential Biomarkers of Preeclampsia: Inverse Correlation between Hydrogen Peroxide and Nitric Oxide Early in Maternal Circulation and at Term in Placenta of Women with Preeclampsia. *Placenta* 2009;**30**:342–7. doi:10.1016/j.placenta.2009.01.003

62 Anand S, Young SA, Esplin MS, *et al.* Detection and confirmation of serum lipid biomarkers for preeclampsia using direct infusion mass spectrometry. *J Lipid Res* 2016;**57**:687–96. doi:10.1194/jlr.P064451

63 Cardus A, Panizo S, Encinas M, *et al.* 1,25-Dihydroxyvitamin D3 regulates VEGF production through a vitamin D response element in the VEGF promoter. *Atherosclerosis* 2009;**204**:85–9. doi:10.1016/j.atherosclerosis.2008.08.020

64 Baker AM, Haeri S, Camargo CA, *et al.* A nested case-control study of midgestation vitamin D deficiency and risk of severe preeclampsia. *J Clin Endocrinol Metab* 2010;**95**:5105–9. doi:10.1210/jc.2010-0996

65 Westwood M, Al-Saghir K, Finn-Sell S, *et al.* Vitamin D attenuates sphingosine-1-phosphate (S1P)-mediated inhibition of extravillous trophoblast migration. *Placenta* 2017;**60**:1–8. doi:10.1016/j.placenta.2017.09.009

66 Werner EF, Braun JM, Yolton K, *et al.* The association between maternal urinary phthalate concentrations and blood pressure in pregnancy: The HOME Study. *Environ Heal A Glob Access Sci Source* 2015;**14**:1–9. doi:10.1186/s12940-015-0062-3

67 Grosso LM, Bracken MB. Caffeine metabolism, genetics, and perinatal outcomes: A review of exposure assessment considerations during pregnancy. *Ann Epidemiol* 2005;**15**:460–6. doi:10.1016/j.annepidem.2004.12.011

68 Bakker R, Steegers EAP, Raat H, *et al.* Maternal caffeine intake, blood pressure, and the risk of hypertensive complications during pregnancy. the generation R study. *Am J Hypertens* 2011;**24**:421–8. doi:10.1038/ajh.2010.242

69 Guessous I, Eap CB, Bochud M. Blood Pressure in Relation to Coffee and Caffeine Consumption. *Curr Hypertens Rep* 2014;**16**:1–9. doi:10.1007/s11906-014-0468-2

70 Mesas AE, Leon-Muñoz LM, Rodriguez-Artalejo F, *et al.* The effect of coffee on blood pressure and cardiovascular disease in hypertensive individuals: A systematic review and meta-analysis. *Am J Clin Nutr* 2011;**94**:1113–26. doi:10.3945/ajcn.111.016667

71 Dobrinas M, Cornuz J, Eap CB. Pharmacogenetics of CYP1A2 activity and inducibility in smokers and exsmokers. *Pharmacogenet Genomics* 2013;**23**:286–92. doi:10.1097/FPC.0b013e3283602e75

72 Goodacre R, Broadhurst D, Smilde AK, *et al.* Proposed minimum reporting standards for data analysis in metabolomics. *Metabolomics* 2007;**3**:231–41. doi:10.1007/s11306-007-

For peer review only - http://bmjopen.bmj.com/site/about/guidelines.xhtml

0081-3

73 Brosens I, Pijnenborg R, Vercruysse L, *et al.* The ‘great obstetrical syndromes’ are associated with disorders of deep placentation. *Am J Obs Gynecol* 2011;**204**:193–201. doi:10.1016/j.ajog.2010.08.009.THE

Legends

Figures

Figure 1. Flow chart of study selection.

Figure 2. Characterization of study selection regarding biological samples, sampling moment and metabolomic analysis techniques applied

Figure 3. Assessment of risk of bias (left) and applicability concerns (right) of included studies.

Figure 4. Preeclampsia related metabolites, chemical classes and biological processes.

Tables

Table 1. Characteristics of included studies.

Table 2. Metabolites and their accuracy measures for predicting preeclampsia according to gestational age of required delivery.

Table 3 – Accuracy measures of metabolites and other pregnancy-related biomarkers.

Supplementary Material

Supplementary material 1 - Excluded studies and reasons for exclusion.

Supplementary material 2 - Predictive metabolites of hypertensive disorders of pregnancy summarized according to their chemical class, subclass, and metabolic pathways.

Supplementary material 3 – Individual QUADAS-2 data for all included studies.

For peer review only

Table 1. Characteristics of included studies.

Authors, year	Country of data collection	Study design	Classification of hypertensive syndrome	Comments
Chappell LC et al, 2002 [1]	United Kingdom	Case-control	ISSHP, 1988	17 women with preeclampsia; 21 without preeclampsia. Chronic hypertensive women included.
Powe CE et al, 2010 [2]	United States of America	Nested case-control	ICD codes	39 preeclampsia, 131 controls. Gestational diabetes excluded.
Kenny LC et al, 2010 [3]	New Zealand; Australia	Nested case-control	ASSHP, 2000	Discovery phase (New Zealand): 60 women with preeclampsia, 60 matched controls (age, ethnicity, body mass index) with uneventful pregnancies. Validation phase (Australia): 39 women with preeclampsia, 40 healthy matched pregnancies (SCOPE study).
Odibo AO et al, 2011 [4]	United States of America	Nested case-control	ACOG, 2002	41 women with preeclampsia, 41 normotensive controls. Pregestational diabetes included.
Woodham PC et al, 2011 [5]	United States of America	Nested case-control	ACOG, 2002	41 women with severe preeclampsia, 123 normotensive uncomplicated pregnancies at term. Chronic illness and multiple pregnancies excluded.
Rijvers CAH et al, 2013 [6]	Netherlands	Retrospective cohort	NHBP, 2000	06 women with preeclampsia, 11 with gestational hypertension, 18 normotensive women
Khalil AA et al, 2013 [7]	United Kingdom	Case-control	ISSHP, 2001	25 women with early preeclampsia, 50 cases of late preeclampsia, 300 normotensive controls (matching 1 case: 4 controls). Only singleton pregnancies; chronic hypertensive women included.
Diaz SO et al, 2013 [8]	Portugal	Prospective cohort	ACOG, 2002	09 women with preterm preeclampsia (31-37weeks); 84 women with healthy term pregnancies
Kenny LC et al, 2013 [9]	New Zealand; Australia	Nested case-control	ASSHP, 2000	49 women with preeclampsia and 49 normotensive nulliparous women
Kuc S et al, 2014 [10]	Netherlands	Nested case-control	ISSHP, 2001	68 early-onset preeclampsia, 99 late-onset preeclampsia, 500 controls
Wetta LA et al, 2014 [11]	United States of America	Nested case-control	ACOG, 2002	89 women with preterm preeclampsia; 177 normotensive and term pregnancies
Bahado-Singh RO et al,	United Kingdom	Prospective cohort	ISSHP, 2001	50 cases of early preeclampsia, 108 normotensive controls.

For peer review only

2015 [12]				Singleton pregnancies delivered at term; birthweight adequate for gestational age. HELLP syndrome cases excluded.
Eichelberger KY et al, 2015 [13]	United States of America	Nested case-control	ACOG, 2002	25 women with severe preeclampsia; 87 normotensive, healthy pregnancies delivered at term. Chronic hypertensive women and multiple pregnancies excluded.
Koster MPH et al, 2015 [14]	Netherlands	Nested case-control	ISSHP, 2001	68 early-onset preeclampsia, 99 late-onset preeclampsia, 500 controls
Bilodeau JF et al, 2015 [15]	Canada	Prospective cohort	CHS, 1997	33 preeclampsia, 60 controls (MIROS study)
Ates S et al, 2016 [16]	Turkey	Prospective cohort	ACOG, 2002	04 women with preeclampsia; 11 women with gestational hypertension; 214 normotensive women
Cantonwine DE et al, 2016 [17]	United States of America	Nested case-control	ACOG, 2002	50 women with preeclampsia, 431 without preeclampsia. Chronic hypertensive women included.
Kiely ME et al, 2016 [18]	Ireland	Nested case-control	ASSHP, 2000	68 preeclampsia, 1528 controls. SGA infants excluded from controls. (SCOPE Study)
Bahado-Singh RO et al, 2017 [19]	United Kingdom	Prospective cohort	ISSHP, 2001	59 late-onset preeclampsia cases, 115 normotensive controls. Singleton pregnancies delivered at term; birthweight adequate for gestational age. HELLP syndrome cases excluded.
Bahado-Singh RO et al, 2017b [20]	United Kingdom	Nested case-control	ISSHP, 2001	35 term preeclampsia cases (≥37w), 65 normotensive controls. Singleton pregnancies, no major malformations; controls with birthweight adequate for gestational age.
Dobierzewska A et al, 2017 [21]	Chile	Case-control	ACOG, 2002	07 women with preeclampsia; 07 normotensive women
Ye Y et al, 2017 [22]	China	Nested case-control	ACOG, 2013	74 women with preeclampsia, 99 normotensive term pregnancies as controls. Only singleton pregnancies; chronic illness excluded in control group.
Gong S et al, 2018 [23]	United Kingdom	Case-control	ACOG, 2002	134 term preeclampsia, 259 normotensive controls. Nulliparous women, singleton pregnancies (POP Study).
Tamblyn JA et al, 2018	Ireland	Case-control	ASSHP, 2000	25 women with preeclampsia, 25 normotensive women (SCOPE

For peer review only - http://bmjopen.bmj.com/site/about/guidelines.xhtml

[24]				Study).
Sovio U et al, 2020 [25]	United Kingdom	Case-cohort	ISSHP, 2001; ACOG, 2013	Training and test with POP Study: 194 women with preeclampsia, 323 controls. Validation within BiB Study: 95 women with preeclampsia, 953 normotensive controls.
Huo X et al, 2020 [26]	Shanghai, China	Case-Cohort	ACOG, 2013	64 gestational hypertension, 71 preeclampsia. Singleton gestation only; chronic hypertensive women excluded.
Rylander L et al, 2020 [27]	Sweden	Case-control	ICD codes	296 preeclampsia, 580 controls. Excluded pregnancies with SGA infants.
Lee SM et al, 2020 [28]	South Korea	Case-control	ACOG, 2013	33 preeclampsia, 66 controls. Singleton pregnancies, congenital malformations excluded.
Kenny L et al, 2020 [29]	Ireland and England	Nested case-control	ISSHP, 2018	97 preeclampsia (23 preterm preeclampsia and 74 term preeclampsia); 335 controls. Nulliparous women, singleton pregnancies. Chronic illness and fetal malformations excluded (SCOPE study)
Shanmugalingam R et al, 2020 [30]	Australia	Prospective cohort	ISSHP, 2018	21 preeclampsia, 103 controls. High risk women.
Harville EW et al, 2021 [31]	United States of America	Case-control	Not mentioned	18 preeclampsia, 109 controls. Only singleton pregnancies.

ACOG: American College of Obstetricians and Gynecologists; CHS, Canadian Hypertensive Society; ASSHP: Australasian Society of the Study of Hypertension in Pregnancy; ICD, International Classification of Diseases; BiB Study: Born in Bradford Study; ISSHP: International Society for the Study of Hypertension in Pregnancy; POP Study: Pregnancy Outcome Prediction; SCOPE Study: Screening for Pregnancy Endpoints; MIROS study: Maternal and Infant Research on Oxidative Stress; PTB: preterm birth; SGA, small for gestational age.

For peer review only

For peer review only - http://bmjopen.bmj.com/site/about/guidelines.xhtml

1 Table 2. Metabolites and their accuracy measures for predicting preeclampsia according to gestational age of required delivery.

**Preeclampsia at any gestational age**					
**Authors, year**	**Metabolomics methods**	**Maternal biological specimen; gestational age at sampling**	**Increased metabolites**	**Decreased metabolites**	**S/ Sp/ AUC (95% CI)**
Chappell LC et al, 2002 [1]	GC-MS	Blood; 20 weeks	Isoprostane 8-epi-prostaglandin F2α		-/ -/ 0.62 (0.44-0.81)
		Blood; 24 weeks	Isoprostane 8-epi-prostaglandin F2α		-/ -/ 0.55 (0.35-0.75)
Powe CE et al, 2010 [2]	Targeted LC-MS/MS	Blood (serum); 11 (±3w)		25(OH)vitamin D [a]	-/ -/ -
Odibo AO et al, 2011 [4]	Targeted LC-MS/MS	Blood (serum); 11-14w	Hydroxyhexanoylcarnitine, phenylalanine, glutamate, alanine		-/ -/ 0.82 (0.80–0.85)
Woodham PC et al, 2011 [5]	Targeted LC-MS/MS	Blood (serum); 15-20w		25(OH)vitamin D	-/ -/ 0.745
Diaz SO et al, 2013 [8]	Untargeted ^{1}H-NMR	Urine; 14-26w	*N*-methyl-2-pyridone-5-carboxamide, 3-methylhistidine, 4-deoxyerythronic acid, *cis*-aconitate, citrate, Glutamine, hippurate, indoxyl sulphate, *N*-methylnicotinamide, sucrose, U35	2-ketoglutarate, 4-OH-hippurate, acetate, carnitine, creatinine, formate, fumarate, galactose, isoleucine, lactose, phenylacetylglutamine, *p*-cresol sulphate, *scyllo*-ino, succinate, trigonelline, tyrosine, U14, U4, U10	-/ -/ -
Kenny LC et al, 2013 [9]	Untargeted H-NMR	Blood (serum); 15 w		Acetamide, Glutamine, Trimethylamine, creatinine, dimethyl sulfone, valine, isoleucine, ornithine, glycine, methionine, betaine, acetate, proline	-/-/-
Rijvers CAH et al, 2013 [6]	Targeted UPLC-MS/MS	Blood (plasma); 12w	L-arginine/ Asymmetric dimethylarginine ratio		-/ -/ -
		Blood (plasma); 16-20w		L-arginine	-/ -/ -
Wetta LA et al, 2014 [11]	Targeted LC-MS/MS	Blood (serum); 15-21w		25(OH)vitamin D [a]	-/ -/ -

For peer review only - http://bmjopen.bmj.com/site/about/guidelines.xhtml

Bilodeau JF et al, 2015 [15]	Targeted HPLC-MS/MS	Blood (plasma); 12-18w	(+/-) 5-iPF2α-VI, (+/-) 5-iPF2α-VI-(d11)		-/ -/ 0.67
	GC-FID	Blood (plasma); 12-18w		Alfa-linolenic acid, stearidonic acid, eicosatrienoic acid, eicosatetraenoic acid, eicosapentaenoic acid, docosapentaenoic acid, docosahexaenoic acid	-/ -/ -
Ates S et al, 2016 [16]	Targeted LC-MS/MS	Blood (serum); 11-14 w		Vitamin D (<10mg/dL)	0.50/0.54
Cantonwine DE et al, 2016 [17]	Targeted HPLC-MS	Urine; 4-16w	Total bisphenol A, Σ di(2-ethylhexyl) phthalate, mono-ethyl phthalate	%mono(2-ethylhexyl) phthalate	-/ -/ -
		Urine; 22-29w	mono(2-ethylhexyl) phthalate, Σ di(2-ethylhexyl) phthalate, mono(3-carboxypropyl) phthalate	monoisobutyl phthalate	-/ -/ -
		Urine; 33-38w	mono(2-ethylhexyl) phthalate, Σ di(2-ethylhexyl) phthalate, mono(3-carboxypropyl) phthalate		-/ -/ -
Kiely ME et al, 2016 [18]	Targeted LC-MS/MS	Blood (serum); 14-16w		25(OH)D3, 3-epi-25(OH)D3, and 25(OH)D2 [a]	-/ -/ -
Dobierzewska A et al, 2017 [21]	Targeted HPLC-MS	Blood (plasma); 11-14w		Sphingomyelin 16:0, sphingomyelin 18:0, ceramide 14:0	-/ -/ -
		Blood (plasma); 32-36w		Ceramide 14:0, ceramide 24:0	-/ -/ -
Ye Y et al, 2017 [22]	Targeted LC-MS	Blood (serum); 16-20w	Free bisphenol A		-/ -/ 0.73 (0.65-0.81)
Tamblyn JA et al, 2018 [24]	Targeted LC-MS/MS	Urine; 14-16w		25(OH) vitamin D3; 24,25(OH)2 vitamin D3[a]	-/ -/ -
Huo X et al, 2020 [26]	HPLC/MS-MS	Blood (serum); 13-17w	Perfluorooctanate; perfluorooctane sulfonate; perfluorodecanoic acid; perfluoroundecanoic acid;		-/ -/ -

For peer review only - http://bmjopen.bmj.com/site/about/guidelines.xhtml

			perfluorononanoic acid; perfluorohexanesulfonate; perfluoroheptanoic acid; perfluorobutane sulfonate; perfluorododecanoic acid; perfluorooctane sulfonamide[a]		
Rylander L et al, 2020 [27]	Targeted LC-MS/MS	Blood (serum); 12-14w	Perfluorohexane sulfonate (0.53–0.78 ng/mL)		0.38/ 0.67/ -
Lee SM et al, 2020 [28]	GC-TOF MS	Blood (plasma); 16-24w		Propane-1,3-diol	0.75/ 0.83/ 0.868 (0.844-0.891)
	LC-Orbitrap MS	Blood (plasma); 16-24w	LysoPE C20:0; SM C30:1	LysoPC C19:0; SM C28:1	
Kenny L et al, 2020 [29]	Targeted LC-MS/MS	Blood (plasma); 14-16w	Dilinoleoyl-glycerol (DLG)		0.39/ 0.90/0.70 (0.59-0.82)
Shanmugalingam R et al, 2020 [30]	Targeted LC-MS/MS	Blood (plasma); 12w		15-epilipoxin-A4	
Harville EW et al, 2021 [31]	Untargeted UPLC-HR-MS	Blood (serum); 6-14w	Cerasinone; bolasterone, 6.30_477.7721m/z, 5.86_313.1070m/z[b]		-/ -/ 0.95
	NMR	Blood (serum); 6-14w		Asparagine, N,N-Dimethylglycine, Trimethylamine	-/ -/ -
**Early onset preeclampsia (<34w)**					
Odibo AO et al, 2011 [4]	Targeted LC-MS/MS	Blood (serum); 11-14w	Hydroxyhexanoylcarnitine, phenylalanine, glutamate, alanine		-/ -/ 0.84
Khalil AA et al, 2013 [7]	Targeted GC-MS; GC-MS/MS	Blood (plasma); 11-13w	asymmetric dimethylarginine/ L-arginine ratio; asymmetric dimethylarginine/ L-homoarginine ratio	L-arginine, L-homoarginine	-/ -/ -
Kuc S et al, 2014 [10]	Targeted UPLC-MS/MS	Blood (serum); 8-13w		Taurine, asparagine	-/ -/ -
Bahado-Singh RO et	Untargeted	Blood (serum); 11-13 w	2-hydroxybutyrate, 3-hydroxyisovalerate,	Acetone, glycerol	0.75/ 0.74/ 0.835

For peer review only - http://bmjopen.bmj.com/site/about/guidelines.xhtml

al, 2015 [12]	^{1}H-NMR		citrate		(0.769-0.941)
Eichelberger KY et al, 2015 [13]	LC-MS	Blood (serum); 16-19w		Paraxanthine/caffeine ratio	-/ -/ -
Koster MPH et al, 2015 [14]	Targeted UPLC-MS/MS	Blood (serum); 8-13w	Hexanoylcarnitine, octenoylcarnitine, octanoylcarnitine, decenoylcarnitine, decanoylcarnitine, dodecanoylcarnitine, lauroylcarnitine, tetradecenoylcarnitine, hexadecenoylcarnitine	Isobutyrylcarnitine, linoleylcarnitine, stearoylcarnitine	-/ -/ -
Sovio U et al, 2020 [25]	Untargeted UPLC-MS/MS	Blood (serum); 12w<br>Blood (serum); 20w<br>Blood (serum);28w	4-hydroxyglutamate		-/-/0.673<br>-/-/0.731<br>-/-/0.765
**Late onset preeclampsia (≥34w)**					
Kenny LC et al, 2010 [3]	Untargeted UPLC-MS	Blood (plasma); 14-16w	Monosaccharide(s), decanoylcarnitine, oleic acid, docosahexaenoic acid and/or docosatriynoic acid, gama-butyrolactone and/or oxolan-3-one, 2-oxovaleric acid and/or oxo-methylbutanoic acid, acetoacetic acid, hexadecenoyleicosatetraenoyl-Sn-glycerol, Di-(octadecadienoyl)-sn-glycerol, sphingosine 1-phosphate, sphinganine 1-phosphate, vitamin D3 derivatives	5-Hydroxytryptophan, methylglutaric acid and/or adipic acid	-/ -/ 0.92[c]
Kuc S et al, 2014 [10]	Targeted UPLC-MS/MS	Blood (serum); 8-13w		Glycylglycine	-/ -/ -
Koster MPH et al, 2015 [14]	Targeted UPLC-MS/MS	Blood (serum); 8-13w	Hexanoylcarnitine, octenoylcarnitine, octanoylcarnitine, decenoylcarnitine,	Linoleylcarnitine, palmitoylcarnitine, oleylcarnitine, stearoylcarnitine	-/ -/ -

For peer review only

For peer review only - http://bmjopen.bmj.com/site/about/guidelines.xhtml

			decanoylcarnitine, dodecanoylcarnitine, tetradecenoylcarnitine		
Cantonwine DE et al, 2016 [17]	Targeted HPLC-MS	Urine; 4-16w	mono(2- ethyl-5-oxohexyl) phthalate, mono(2-ethyl-5-carboxypentyl) phthalate, Σ di(2-ethylhexyl) phthalate, mono-ethyl phthalate		-/ -/ -
		Urine; 33-38w	mono(2- ethyl-5-oxohexyl) phthalate, mono(2-ethyl-5-carboxypentyl) phthalate, Σ di(2-ethylhexyl) phthalate, Mono-*n*-butyl phthalate		-/ -/ -
Bahado-Singh RO et al, 2017 [19]	Untargeted ^{1}H-NMR	Blood (serum); 11-13 w	Carnitine, pyruvate, acetone	-	0.30/ 0.80/ 0.629 (0.490-0.767)
Bahado-Singh RO et al, 2017b [20]	Untargeted ^{1}H-NMR, targeted LC-MS/MS	Blood (serum); 11-13w	-	Putrescine (LC-MS); urea, carnitine (H-NMR)	0.72/ 0.57/ 0.701
		Blood (serum); 32-33w	Methylhistidine, propylene glycol (H-NMR), citrate, hexose[d]	Serotonin (LC-MS)	0.74/ 0.72/ 0.761
Gong S et al, 2018 [23]	Untargeted LC-MS/MS	Blood (serum); 36w	N1, N12-diacetylspermine[e]	-	-
**Gestational hypertension**					
Ates S et al, 2016 [16]	Targeted LC-MS/MS	Blood (serum); 11-14 w		Vitamin D (<10mg/dL)	0.45/0.54
Huo X et al, 2020 [26]	HPLC/ MS-MS	Blood (serum); 13-17w	Perfluoroheptanoic acid[f]		-/ -/ -
Harville EW et al, 2021 [31]	Untargeted UPLC-HR-MS	Blood (serum); 6-14w	Neomenthol-glucuronide, dibenzylamine, pilocarpine, 2,6-Di-*tert*-butyl-4-hydroxymethylphenol, 0.59_746.6045n, 8.66_762.1452m/z, 12.74_412.2842m/z		-/ - / 0.95[b]

AUC: area under the curve; GC-MS: gas-chromatography coupled to mass spectrometry; GC-FID: gas-chromatography coupled with a flame ionization detector; ^{1}H-NMR: hydrogen nuclear magnetic resonance; HPLC-MS: High Performance Liquid Chromatography- mass spectrometry; LC-MS: liquid- chromatography coupled to mass

For peer review only - http://bmjopen.bmj.com/site/about/guidelines.xhtml

spectrometry; GC-TOF MS: gas chromatography time-of-flight mass spectrometry; LC-Orbitrap MS: liquid chromatography Orbitrap mass spectrometry. S, sensitivity; Sp, specificity; UPLC-MS: ultra- Performance Liquid Chromatography- mass spectrometry; 95% CI, 95% confidence interval.

[a]No statistical difference between preeclampsia and normotensive controls. [b]AUC for all metabolites model; some of them were not identified, thus they are presented as retention time and mass; it is unclear which metabolite was up or downregulated in preeclampsia cases. [c]Accuracy measures for the validation phase. [d]Unclear type of platform that has found this metabolite, or if it is increased or decreased in preeclampsia. [e]Higher preeclampsia risk with increasing N1, N12-diacetylspermine concentrations. [f] No statistical difference between preeclampsia normotensive controls.

For peer review only

For peer review only - http://bmjopen.bmj.com/site/about/guidelines.xhtml

1 Table 3 – Accuracy measures of metabolites and other pregnancy-related biomarkers.

Maternal specimen	Trimester of pregnancy	Metabolites	Additional variables considered in prediction models	AUC of metabolites + additional variables model (95% CI)
**Preeclampsia at any gestational age**				
Blood	1st	Dilinoleoyl-glycerol heptadecanoyl-2-hydroxy-sn-glycero-3-phosphocholine	PlGF	0.78 (0.69-0.88) [29]
Blood	2nd	25OHD	VEGF + sFLT-1/PlGF	0.851 [5]
Blood	3rd	4-hydroxyglutamate	s-Flt-1/PlGF	0.834 [25]
**Early onset preeclampsia (delivery <34w)**				
Blood	1st	Taurine	Prior risk+ MAP	0.93 [10]
		Stearoylcarnitine	Prior risk+ MAP	0.747 [14]
		3-hydroxyisovalerate, arginine, glycerol	UtPI	0.917 [12]
**Late onset preeclampsia (delivery ≥34w)**				
Blood	1st	Pyruvate, carnitine, glycerol	UtPI	0.722 [19]
Blood	1st	Stearoylcarnitine	Prior risk+ MAP	0.828 [14]
Blood	1st and 3rd	Urea, SM C18:1 (1st), Hexose, Citrate (3rd)	Maternal BMI (12w) + MAP (32w)	0.805 [20]

2 AUC: area under the curve; BMI: body mass index; MAP, mean arterial pressure; UtPI: uterine arteries pulsatility index; CI: confidence interval.

3

For peer review only

For peer review only - http://bmjopen.bmj.com/site/about/guidelines.xhtml

References

1 Chappell L. A longitudinal study of biochemical variables in women at risk of preeclampsia. *Am J Obstet Gynecol* 2002;**187**:127–36. doi:10.1067/mob.2002.122969

2 Powe CE, Seely EW, Rana S, *et al.* First trimester vitamin D, vitamin D binding protein, and subsequent preeclampsia. *Hypertension* 2010;**56**:758–63. doi:10.1161/HYPERTENSIONAHA.110.158238

3 Kenny LC, Broadhurst DI, Dunn W, *et al.* Robust early pregnancy prediction of later preeclampsia using metabolomic biomarkers. *Hypertension* 2010;**56**:741–9. doi:10.1161/HYPERTENSIONAHA.110.157297

4 Odibo1 AO, Goetzinger KR, Odibo L, *et al.* First-trimester prediction of preeclampsia using metabolomic biomarkers: a discovery phase study. *Prenat Diagn* 2011;**31**:990–4.

5 Woodham PC, Brittain JE, Baker AM, *et al.* Midgestation maternal serum 25-hydroxyvitamin D level and soluble fms-like tyrosine kinase 1/placental growth factor ratio as predictors of severe preeclampsia. *Hypertension* 2011;**58**:1120–5. doi:10.1161/HYPERTENSIONAHA.111.179069

6 Rijvers CAH, Marzano S, Winkens B, *et al.* Early-pregnancy asymmetric dimethylarginine (ADMA) levels in women prone to develop recurrent hypertension. *Pregnancy Hypertens* 2013;**3**:118–23. doi:10.1016/j.preghy.2013.01.001

7 Khalil AA, Tsikas D, Akolekar R, *et al.* Asymmetric dimethylarginine, arginine and homoarginine at 11-13 weeks gestation and preeclampsia: A case-control study. *J Hum Hypertens* 2013;**27**:38–43. doi:10.1038/jhh.2011.109

8 Diaz SO, Barros AS, Goodfellow BJ, *et al.* Second trimester maternal urine for the diagnosis of trisomy 21 and prediction of poor pregnancy outcomes. *J Proteome Res* 2013;**12**:2946–57. doi:10.1021/pr4002355

9 Kenny LC, Broadhurst DI, Hong W, *et al.* NMR Profiling in Early Pregnancy Reveals a Metabolomic Signature the Predicts the Subsequent Development Preeclampsia. *Reprod Sci* 2013;**20**:2641A. doi:10.1177/1933719113482088

10 Kuc S, Koster MPH, Pennings JLA, *et al.* Metabolomics profiling for identification of novel potential markers in early prediction of preeclampsia. *PLoS One* 2014;**9**. doi:10.1371/journal.pone.0098540

11 Wetta LA, Biggio JR, Cliver S, *et al.* Is midtrimester vitamin D status associated with spontaneous preterm birth and preeclampsia? *Am J Perinatol* 2014;**31**:541–6. doi:10.1055/s-0033-1356483

For peer review only - http://bmjopen.bmj.com/site/about/guidelines.xhtml

12 Bahado-Singh RO, Syngelaki A, Akolekar R, *et al.* Validation of metabolomic models for prediction of early-onset preeclampsia. *Am J Obstet Gynecol* 2015;**213**:530.e1-530.e10. doi:10.1016/j.ajog.2015.06.044

13 Eichelberger KY, Baker AM, Woodham PC, *et al.* Second-trimester maternal serum paraxanthine, CYP1A2 activity, and the risk of severe preeclampsia. *Obstet Gynecol* 2015;**126**:725–30. doi:10.1097/AOG.0000000000001041

14 Koster MPH, Vreeken RJ, Harms AC, *et al.* First-Trimester serum acylcarnitine levels to predict preeclampsia: A metabolomics approach. *Dis Markers* 2015;**2015**. doi:10.1155/2015/857108

15 Bilodeau JF, Qin Wei S, Larose J, *et al.* Plasma F2-isoprostane class VI isomers at 12-18 weeks of pregnancy are associated with later occurrence of preeclampsia. *Free Radic Biol Med* 2015;**85**:282–7. doi:10.1016/j.freeradbiomed.2015.05.012

16 Ates S, Sevket O, Ozcan P, *et al.* Vitamin D status in the first-trimester: Effects of vitamin D deficiency on pregnancy outcomes. *Afr Health Sci* 2016;**16**:36–43. doi:10.4314/ahs.v16i1.5

17 Cantonwine DE, Meeker JD, Ferguson KK, *et al.* Urinary concentrations of bisphenol A and phthalate metabolites measured during pregnancy and risk of preeclampsia. *Environ Health Perspect* 2016;**124**:1651–5. doi:10.1289/EHP188

18 Kiely ME, Zhang JY, Kinsella M, *et al.* Vitamin D status is associated with uteroplacental dysfunction indicated by pre-eclampsia and small-for-gestational-age birth in a large prospective pregnancy cohort in Ireland with low Vitamin D status. *Am J Clin Nutr* 2016;**104**:354–61. doi:10.3945/ajcn.116.130419

19 Bahado-Singh RO, Syngelaki A, Mandal R, *et al.* Metabolomic determination of pathogenesis of late-onset preeclampsia. *J Matern Neonatal Med* 2017;**30**:658–64. doi:10.1080/14767058.2016.1185411

20 Bahado-Singh R, Poon LC, Yilmaz A, *et al.* Integrated Proteomic and Metabolomic prediction of Term Preeclampsia. *Sci Rep* 2017;**7**:1–10. doi:10.1038/s41598-017-15882-9

21 Dobierzewska A, Soman S, Illanes SE, *et al.* Plasma cross-gestational sphingolipidomic analyses reveal potential first trimester biomarkers of preeclampsia. *PLoS One* 2017;**12**:1–16. doi:10.1371/journal.pone.0175118

22 Ye Y, Zhou Q, Feng L, *et al.* Maternal serum bisphenol A levels and risk of pre-eclampsia: A nested case-control study. *Eur J Public Health* 2017;**27**:1102–7. doi:10.1093/eurpub/ckx148

23 Gong S, Sovio U, Aye ILMH, *et al.* Placental polyamine metabolism differs by fetal sex, fetal growth restriction, and preeclampsia. *JCI Insight* 2018;**3**:1–15.

For peer review only - http://bmjopen.bmj.com/site/about/guidelines.xhtml

doi:10.1172/jci.insight.120723

24 Tamblyn JA, Jenkinson C, Larner DP, *et al.* Serum and urine vitamin D metabolite analysis in early preeclampsia. *Endocr Connect* 2018;**7**:199–210. doi:10.1530/EC-17-0308

25 Sovio U, McBride N, Wood AM, *et al.* 4-Hydroxyglutamate is a novel predictor of pre-eclampsia. *Int J Epidemiol* 2020;**49**:301–11. doi:10.1093/ije/dyz098

26 Huo X, Huang R, Gan Y, *et al.* Perfluoroalkyl substances in early pregnancy and risk of hypertensivedisorders of pregnancy: A prospective cohort study. *Environ Int* 2020;**138**. doi:10.1016/j.envint.2020.105656

27 Rylander L, Lindh CH, Hansson SR, *et al.* Per- and Polyfluoroalkyl Substances in Early Pregnancy and Risk for Preeclampsia: A Case-Control Study in Southern Sweden. *Toxics* 2020;**8**. doi:10.3390/toxics8020043

28 Lee SM, Kang Y, Lee EM, *et al.* Metabolomic biomarkers in midtrimester maternal plasma can accurately predict the development of preeclampsia. *Sci Rep* 2020;**10**:16142. doi:10.1038/s41598-020-72852-4

29 Kenny LC, Thomas G, Poston L, *et al.* Prediction of preeclampsia risk in first time pregnant women: Metabolite biomarkers for a clinical test. *PLoS One* 2020;**15**:1–19. doi:10.1371/journal.pone.0244369

30 Shanmugalingam R, Wang X, Motum P, *et al.* The 15-Epilipoxin-A4 Pathway with Prophylactic Aspirin in PreventingPreeclampsia: A Longitudinal Cohort Study. *J Clin Endocrinol Metab* 2020;**105**. doi:10.1210/clinem/dgaa642

31 Harville EW, Li Y-YY-Y, Pan K, *et al.* Untargeted analysis of first trimester serum to reveal biomarkers of pregnancy complications: a case-control discovery phase study. *Sci Rep* 2021;**11**:3468. doi:10.1038/s41598-021-82804-1

For peer review only

For peer review only - http://bmjopen.bmj.com/site/about/guidelines.xhtml

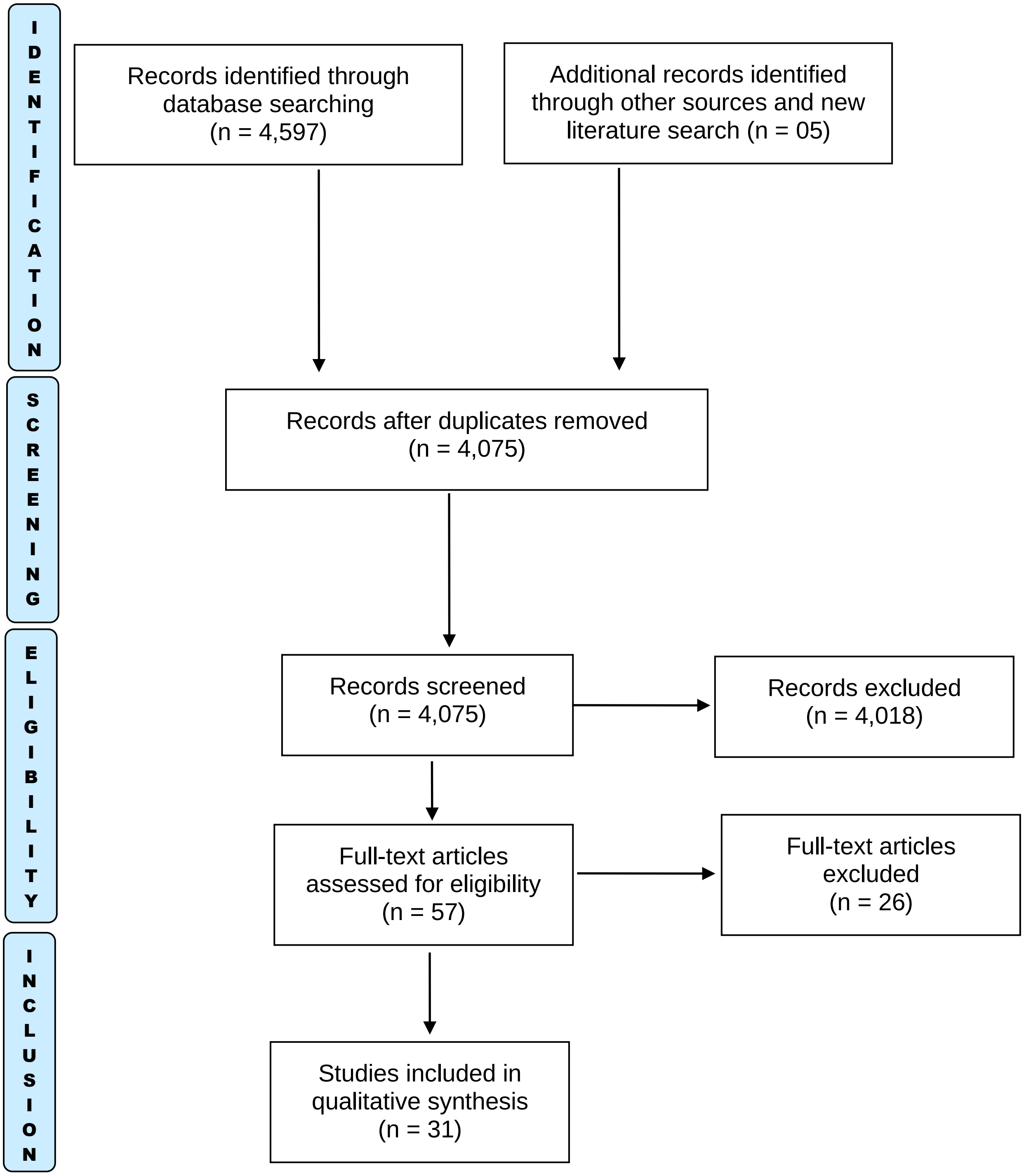

From: Moher D, Liberati A, Tetzlaff J, Altman DG, The PRISMA Group (2009). *P*referred *R*eporting *I*tems for *S*ystematic Reviews and *M*eta-*A*nalyses: The PRISMA Statement. PLoS Med 6(7): e1000097. doi:10.1371/journal.pmed1000097

For more information, visit www.prisma-statement.org.

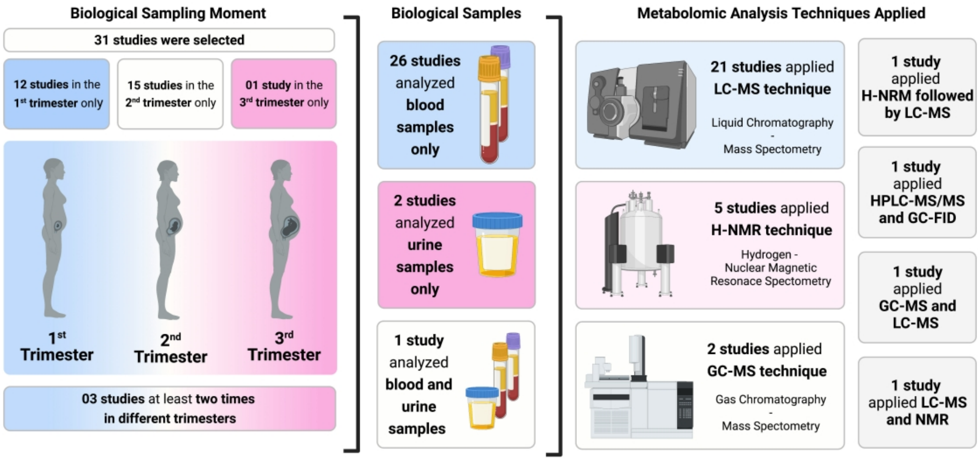

Figure 2

419x199mm (300 x 300 DPI)

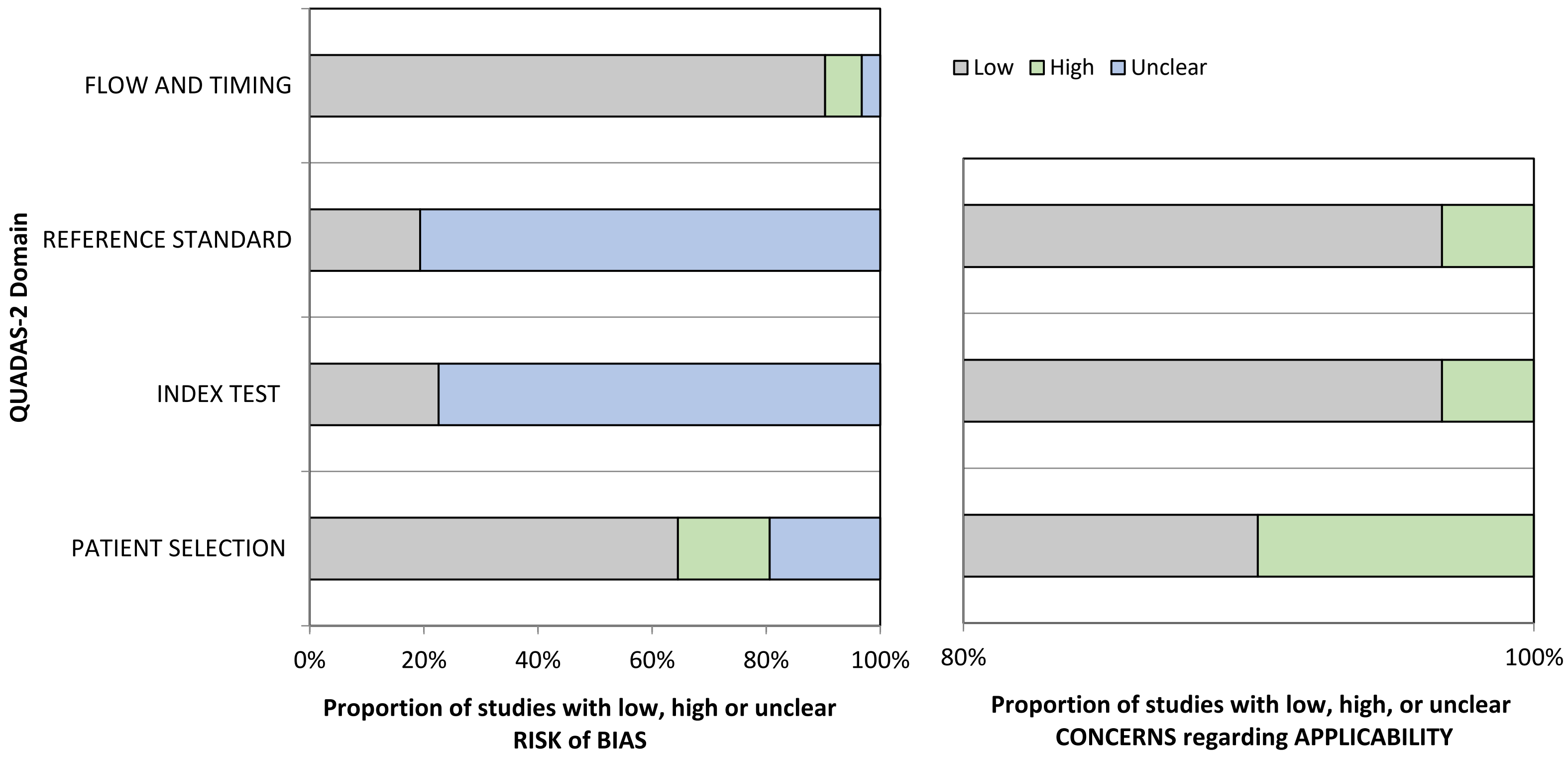
Low
High
Unclear
QUADAS-2 Domain
FLOW AND TIMING
REFERENCE STANDARD
INDEX TEST
PATIENT SELECTION
0%
20%
40%
60%
80%
100%
Proportion of studies with low, high or unclear RISK of BIAS
80%
100%
Proportion of studies with low, high, or unclear CONCERNS regarding APPLICABILITY

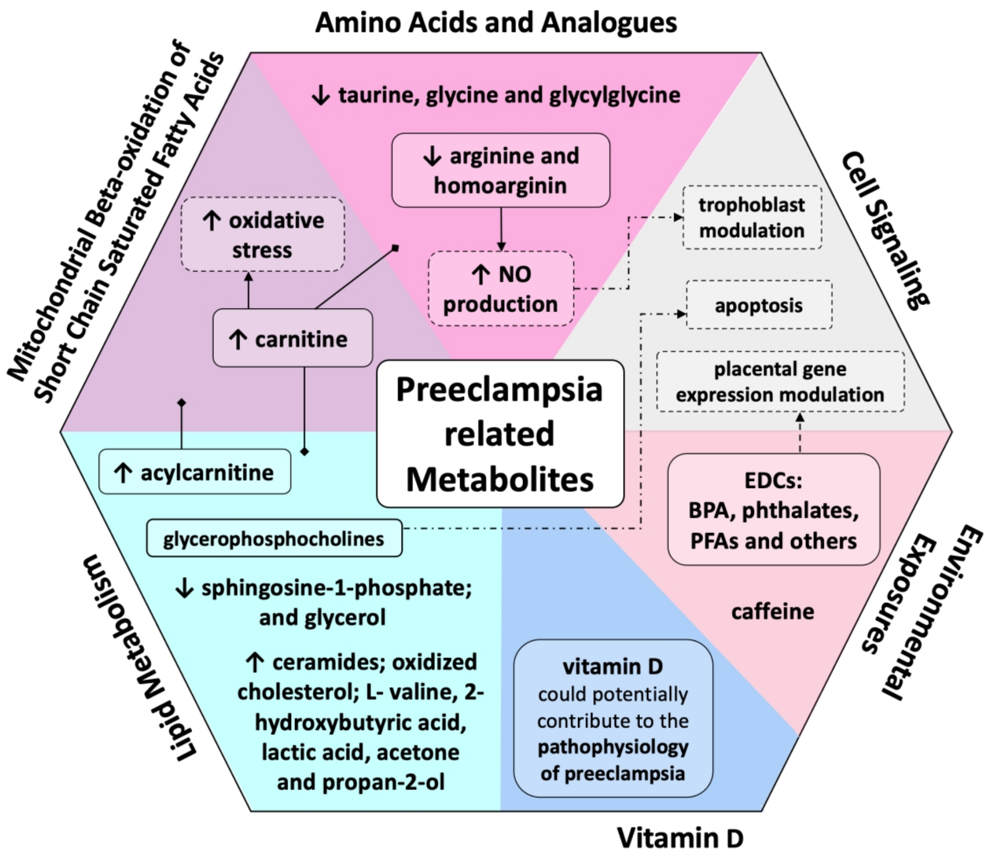

Figure 4

355x313mm (144 x 144 DPI)

Prediction of pregnancy-related hypertensive disorders using metabolomics: a systematic review

Jussara Mayrink, Debora Farias Batista Leite, Maria Laura Costa, Guilherme M Nobrega, José Guilherme Cecatti

Supplementary material 1 – Excluded studies and reasons for exclusion.

Authors	DOI	Reason for exclusion
Kenny LC et al, 2008	10.1177/1933719108316908	Cross-sectional design.
Aris A et al, 2009	10.1016/j.placenta.2009.01.003	Non metabolomics research.
Kenny LC et al, 2009		Conference abstract. Duplicated data. Please check Kenny LC et al (10.1161/HYPERTENSIONAHA.110.157297)
Baker AM et al, 2010	10.1210/jc.2010-0996	Duplicated data. Please check Woodham PC et al, 2011 (10.1161/HYPERTENSIONAHA.111.179069)
Bahado-Singh RO et al, 2012	10.3109/14767058.2012.680254	Duplicated data. Please check Bahado-Singh RO et al, 2015 (10.1016/j.ajog.2015.06.044)
Austdal M et al, 2013	10.1016/j.preghy.2013.04.030	Cross-sectional design.
Bahado-Singh RO et al, 2013	10.1016/j.ajog.2012.11.003	Duplicated data. Please check Bahado-Singh RO et al, 2017 (10.1080/14767058.2016.1185411)
Senyavina NV et al, 2013	10.1007/s10517-013-2225-y	Cross-sectional design.
Bodnar et al, 2014	10.1097/EDE.0000000000000039	Imputation of data obtained in a small subset to the whole sample.
Austdal M et al, 2014	10.1371/journal.pone.0091923	Duplicated data. Please check Austdal M et al, 2015 (10.3390/ijms160921520)
Austdal M et al, 2015	10.1016/j.placenta.2015.10.019	Cross-sectional design.
Austdal M et al, 2015	10.3390/ijms160921520	Comparison of preeclampsia patients with women with normal blood pressure and gestational hypertension together.
Riley J et al, 2016		Conference abstract. Duplicated data. Please check Odibo A et al 2011 (10.1002/pd.2822)
Chen T et al, 2017	10.1016/j.bbrc.2017.02.032	Cross-sectional design.
Kelly RS et al, 2017	10.1007/s11306-016-1149-8	Randomized clinical trial.
Powell KL et al, 2018	10.1016/j.placenta.2018.04.005	Cross-sectional design.
Kawasaki K et al, 2019	10.1161/HYPERTENSIONAHA.118.12389	Cross-sectional design.
Liu Y et al, 2019	10.1111/1440-1681.13095	Cross-sectional design.
Sander KN et al, 2019	10.1007/s11306-019-1600-8	Cross-sectional design.

For peer review only

Chaudhry SH et al, 2019	10.1186/s12884-019-2219-5	Non metabolomics research.
Lewandowska M et al, 2019	10.3390/nu11051028	No specific data on preeclampsia.
Ferranti et al, 2020	10.1155/2020/1515321	Mention of altered metabolic pathways, but not metabolites.
Kelly C et al, 2020	10.3390/nu12072048	Non metabolomics research.
Kyozuka H et al, 2020	10.1038/s41598-020-70974-3	No specific data on preeclampsia.
McBride N et al, 2020	10.1186/s12916-020-01819-z	Randomized clinical trial.
Jääskeläinen T et al, 2021	10.1007/s11306-020-01752-5	No specific data on preeclampsia compared to normotensive women.

For peer review only

Supplementary Material 2. Predictive metabolites of hypertensive disorders of pregnancy summarized according to their chemical class, subclass, and metabolic pathways.

Predictive metabolites	Chemical class	Chemical subclass	Metabolic pathway or process	Involved with
		Organic acids and derivatives		
N1, N12-diacetylspermine	Carboximidic acids and derivatives	Carboximidic acids	NA	Late onset preeclampsia[1]
Acetamide	Carboximidic acids and derivatives	Carboximidic acids	NA	Preeclampsia [2]
Acetate	Carboxylic acids and derivatives	Carboxylic acids	Glycolysis, gluconeogenesis; pyruvate, propanoate, taurine and hypotaurine metabolism	Preeclampsia [2,3]
3-methylhistidine	Carboxylic acids and derivatives	Amino acids, peptides, and analogues	Beta-alanine, histidine and methylhistidine metabolism	Preeclampsia [3]
Methylhistidine	Carboxylic acids and derivatives	Amino acids, peptides, and analogues	Histidine Metabolism	Late onset preeclampsia [4]
Alanine	Carboxylic acids and derivatives	Amino acids, peptides, and analogues	Glutathione metabolism, glycine and serine metabolism, tryptophan metabolism, urea cycle, glutamate metabolism, glucose-alanine cycle	Preeclampsia [5], early onset [5] preeclampsia
Betaine	Carboxylic acids and derivatives	Amino acids, peptides, and analogues	Betaine metabolism, glycine and serine metabolism, methionine metabolism	Preeclampsia [2]
Asymmetric dimethylarginine	Carboxylic acids and derivatives	Amino acids, peptides, and analogues	NA	Preeclampsia [6], early onset [7] preeclampsia
L-arginine	Carboxylic acids and derivatives	Amino acids, peptides, and analogues	Urea cycle, arginine and proline metabolism, glycine and serine metabolism, transcription/translation	Preeclampsia [6], early onset [7] preeclampsia
L-homoarginine	Carboxylic acids and derivatives	Amino acids, peptides, and analogues	NA	Early onset [7] preeclampsia

For peer review only

Creatinine	Carboxylic acids and derivatives	Amino acids, peptides, and analogues	Arginine and proline metabolism	Preeclampsia [2,3]
Glutamine	Carboxylic acids and derivatives	Amino acids, peptides, and analogues	Pyrimidine metabolism, glutamate metabolism, purine metabolism, transcription/translation, urea cycle	Preeclampsia [2,3]
Glutamate	Carboxylic acids and derivatives	Amino acids, peptides, and analogues	Alanine metabolism, histidine metabolism, ammonia recycling, cysteine metabolism, arachidonic acid metabolism	Preeclampsia [5], early onset [5] preeclampsia
4-hydroxyglutamate	Carboxylic acids and derivatives	Amino acids, peptides, and analogues	Arginine and proline metabolism	Preeclampsia [8]
Glycine	Carboxylic acids and derivatives	Amino acids, peptides, and analogues	Ammonia recycling, porphyrin metabolism, glutathione metabolism, glycine and serine metabolism	Preeclampsia [2]
Glycylglycine	Carboxylic acids and derivatives	Amino acids, peptides, and analogues	NA	Late onset [9] preeclampsia
Isoleucine	Carboxylic acids and derivatives	Amino acids, peptides, and analogues	Valine, leucine and isoleucine biosynthesis and degradation	Preeclampsia [2,3]
Asparagine	Carboxylic acids and derivatives	Amino acids, peptides, and analogues	Alanine, aspartate and glutamate metabolism; protein digestion and absorption	Preeclampsia [10]
Methionine	Carboxylic acids and derivatives	Amino acids, peptides, and analogues	Betaine metabolism, glycine and serine metabolism; spermidine and spermine biosynthesis	Preeclampsia [2]
N,N-Dimethylglycine	Carboxylic acids and derivatives	Amino acids, peptides, and analogues	Glycine, serine, and threonine metabolism	Preeclampsia [10]
Ornithine	Carboxylic acids and derivatives	Amino acids, peptides, and analogues	Urea cycle, arginine and proline metabolism; glycine and serine	Preeclampsia [2]

For peer review only

			metabolism; Spermidine and Spermine Biosynthesis	
Phenylacetylglutamine	Carboxylic acids and derivatives	Amino acids, peptides, and analogues	Phenylacetate metabolism, phenylalanine metabolism	Preeclampsia [3]
Phenylalanine	Carboxylic acids and derivatives	Amino acids, peptides, and analogues	Phenylalanine, tyrosine and tryptophan biosynthesis; phenylalanine metabolismo;	Preeclampsia [5], early onset [5] preeclampsia
Proline	Carboxylic acids and derivatives	Amino acids, peptides, and analogues	Arginine and proline metabolism	Preeclampsia [2]
Tyrosine	Carboxylic acids and derivatives	Amino acids, peptides, and analogues	Tyrosine metabolism, phenylalanine and tyrosine metabolism, catecholamine biosynthesis, transcription/translation	Preeclampsia [3]
Valine	Carboxylic acids and derivatives	Amino acids, peptides, and analogues	Valine, leucine and isoleucine biosynthesis and degradation; propanoate metabolism	Preeclampsia [2]
Formate	Carboxylic acids and derivatives	Carboxylic acids	Folate metabolism, pterine biosynthesis	Preeclampsia [3]
Fumarate	Carboxylic acids and derivatives	Dicarboxylic acids and derivatives	Phenylalanine and tyrosine metabolism, arginine and proline metabolism, aspartate metabolism, urea cycle, mitochondrial electron transport chain	Preeclampsia [3]
Succinate	Carboxylic acids and derivatives	Dicarboxylic acids and derivatives	Citrate cycle, oxidative phosphorylation; alanine, aspartate and glutamate metabolism; pyruvate metabolism; cAMP signalling pathway; GABAergic synapse; glucagon signalling pathway	Preeclampsia [3]
Cis-aconitate	Carboxylic acids and derivatives	Tricarboxylic acids and derivatives	Citric acid cycle	Preeclampsia [3]

For peer review only

Citrate	Carboxylic acids and derivatives	Tricarboxylic acids and derivatives	Citric acid cycle, transfer of acetyl groups into mitochondria	Preeclampsia [3], early onset [11] preeclampsia, late onset [4] preeclampsia
2-hydroxybutyrate	Hydroxy acids and derivatives	Alpha hydroxy acids and derivatives	Propanoate metabolism	Early onset [11] preeclampsia
2-ketoglutarate	Keto acids and derivatives	Gamma-keto acids and derivatives	Citric acid cycle, gluconeogenesis, oxidation of branched-chain fatty acids, urea cycle, branched-chain amino acids degradation	Preeclampsia [3]
2-oxovaleric acid	Keto acids and derivatives	Short-chain keto acids and derivatives	NA	Late onset [12] preeclampsia
Acetoacetic acid	Keto acids and derivatives	Short-chain keto acids and derivatives	Ketone body metabolism, fatty acid biosynthesis, branched-chain amino acids degradation, tyrosine metabolism, phenylalanine and tyrosine metabolism	Late onset [12] preeclampsia
Oxo-methylbutanoic acid	Keto acids and derivatives	Short-chain keto acids and derivatives	Cell signaling; valine, leucine and isoleucine Degradation	Late onset [12] preeclampsia
Pyruvate	Keto acids and derivatives	Alpha keto acids and derivatives	Urea Cycle, glucose-alanine cycle, glycine and serine Metabolism, pyruvate metabolism, alanine metabolism	Late onset [13] preeclampsia
Urea	Organic carbonic acids and derivatives	Ureas	Urea cycle; D-arginine and D-ornithine metabolism; arginine and proline metabolism	Late onset [4] preeclampsia
Taurine	Organic sulfonic acids and derivatives	Organosulfonic acids and derivatives	Taurine and hypotaurine metabolism	Early onset [9] preeclampsia
Indoxyl sulphate	Organic sulfuric acids and derivatives	Arylsulfates	NA	Preeclampsia [3]
p-cresol sulphate	Organic sulfuric acids and derivatives	Arylsulfates	NA	Preeclampsia [3]

For peer review only - http://bmjopen.bmj.com/site/about/guidelines.xhtml

Lipids and lipid-likes molecules				
Ceramide 14:0	Ceramides	N-acylsphingosines (ceramides)	NA	Preeclampsia [14]
Ceramide 24:0	Ceramides	N-acylsphingosines (ceramides)	NA	Preeclampsia [14]
3-hydroxyisovalerate	Fatty acyls	Fatty acids and conjugates	Lipid transport, metabolism, and peroxidation; fatty acid metabolism	Early onset [11] preeclampsia
Adipic acid	Fatty acyls	Fatty acids and conjugates	Lipid transport, metabolism, and peroxidation; fatty acid metabolism	Late onset [12] preeclampsia
Docosapentaenoic acid	Fatty acyls	Fatty acids and conjugates	Lipid transport, metabolism, and peroxidation; fatty acid metabolism; cell signalling; alpha linolenic acid and linoleic acid metabolism	Preeclampsia [15]
Docosahexaenoic acid	Fatty acyls	Fatty acids and conjugates	Lipid transport, metabolism, and peroxidation; fatty acid metabolism; cell signalling; alpha linolenic acid and linoleic acid metabolism	Preeclampsia [15], Late onset [12] preeclampsia
Docosatriynoic acid	Fatty acyls	Fatty acids and conjugates	Lipid transport, metabolism, and peroxidation; fatty acid metabolism; cell signalling	Late onset [12] preeclampsia
Eicosatrienoic acid	Fatty acyls	Fatty acids and conjugates	Lipid transport, metabolism, and peroxidation; fatty acid metabolism; cell signalling	Preeclampsia [15]
Eicosatetraenoic acid	Fatty acyls	Fatty acids and conjugates	Lipid transport, metabolism, and peroxidation; fatty acid metabolism; cell signalling; alpha linolenic acid and linoleic acid metabolism.	Preeclampsia [15]
Eicosapentaenoic acid	Fatty acyls	Fatty acids and conjugates	Lipid transport, metabolism, and peroxidation; fatty acid metabolism; cell	Preeclampsia [15]

For peer review only

			signalling; alpha linolenic acid and linoleic acid metabolism.	
Methylglutaric acid	Fatty acyls	Fatty acid and conjugates	Lipid transport, metabolism, and peroxidation; fatty acid metabolism; cell signalling	Late onset [12] preeclampsia
Oleic acid	Fatty acyls	Fatty acids and conjugates	Fatty acid biosynthesis, biosynthesis of unsaturated fatty acids	Late onset [12] preeclampsia
Decanoylcarnitine	Fatty acyls	Fatty acid esters	Lipid transport and peroxidation; fatty acid metabolism; cell signaling	Early onset [16] preeclampsia, late onset [12] preeclampsia
Decenoylcarnitine	Fatty acyls	Fatty acid esters	NA	Early onset [16] preeclampsia, late onset preeclampsia
Lauroylcarnitine	Fatty acyls	Fatty acid esters	Lipid transport, metabolism, and peroxidation; fatty acid metabolism; cell signalling	Early onset [16] preeclampsia
Hexadecenoylcarnitine	Fatty acyls	Fatty acid esters	Lipid transport, metabolism, and peroxidation; fatty acid metabolism; cell signalling	Early onset [16] preeclampsia
Hexanoylcarnitine	Fatty acyls	Fatty acid esters	Lipid transport, metabolism, and peroxidation; fatty acid metabolism; cell signalling	Early onset [16] preeclampsia, late onset [16] preeclampsia
Hydroxyhexanoylcarnitine	Fatty acyls	Fatty acid esters	Lipid transport, metabolism, and peroxidation; fatty acid metabolism; cell signalling	Preeclampsia [5], early onset [5] preeclampsia
Isobutyrylcarnitine	Fatty acyls	Fatty acid esters	Lipid transport, metabolism, and peroxidation; fatty acid metabolism; cell signalling	Early onset [16] preeclampsia
Octanoylcarnitine	Fatty acyls	Fatty acid esters	Mitochondrial beta-oxidation of short chain saturated fatty acids	Early onset [16] preeclampsia, late onset [16] preeclampsia

For peer review only

Octenoylcarnitine	Fatty acyls	Fatty acid esters	Lipid transport, metabolism, and peroxidation; fatty acid metabolism; cell signalling	Early onset [16] preeclampsia, late onset [16] preeclampsia
Oleylcarnitine	Fatty acyls	Fatty acid esters	NA	Late onset [16] preeclampsia
Palmitoylcarnitine	Fatty acyls	Fatty acid esters	Fatty acid metabolism	Late onset [16] preeclampsia
Isoprostane 8-epi-prostaglandin F2α	Fatty acyls	Eicosanoids	Arachidonic acid metabolism	Preeclampsia [17]
(+/-) 5-iPF2α-VI	Fatty acyls	Eicosanoids	Lipid peroxidation	Preeclampsia [15]
(+/-) 5-iPF2α-VI-(d11)	Fatty acyls	Eicosanoids	Lipid peroxidation	Preeclampsia [15]
15-epilipoxin-A4	Fatty acyls	Eicosanoids	Lipid transport, metabolism, and peroxidation; fatty acid metabolism; cell signalling	Preeclampsia [18]
Alfa-linolenic acid	Fatty acyls	Lineolic acids and derivatives	Lipid transport, metabolism, and peroxidation; fatty acid metabolism; cell signalling; alpha linolenic acid and linoleic acid metabolism.	Preeclampsia [15]
Stearidonic acid	Fatty acyls	Lineolic acids and derivatives	Lipid transport, metabolism, and peroxidation; fatty acid metabolism; cell signalling; alpha linolenic acid and linoleic acid metabolism.	Preeclampsia [15]
Di-(octadecadienoyl)-sn-glycerol	Glycerophospholipids	Glycerophosphates	Lipid transport, metabolism, and peroxidation; fatty acid metabolism; cell signalling	Late onset [12] preeclampsia
Heptadecanoyl-2-hydroxy-sn-glycero-3-phosphocholine	Glycerophospholipids	Glycerophosphocholines	Lipid transport, metabolism, and peroxidation; fatty acid metabolism; cell signalling; phospholipid and glycerophospholipid metabolism.	Preeclampsia [19]
Dilinoleoyl-glycerol	Glycerophospholipids	Glycerophosphocholines	NA	Preeclampsia [19]

For peer review only

LysoPE C20:0	Glycerophospholipids	Glycerophosphoethanolamines	Lipid peroxidation and metabolism; glycerophospholipid metabolism; fatty acid metabolism cell signalling.	Preeclampsia [20]
Neomenthol-glucuronide	Prenol lipids	Terpene glycosides	Lipid peroxidation and metabolism; glycerophospholipid metabolism; fatty acid metabolism cell signalling.	Gestational hypertension [10]
Sphinganine 1-phosphate	Sphingolipids	Phosphosphingolipids	Sphingolipid metabolism, neuroactive ligand-receptor interaction	Late onset [12] preeclampsia
Sphingomyelin 16:0	Sphingolipids	Phosphosphingolipids	Lipid transport, metabolism, and peroxidation; fatty acid metabolism; cell signalling; apoptosis	Preeclampsia [14]
Sphingomyelin 18:0	Sphingolipids	Phosphosphingolipids	Phospholipid metabolism; lipid transport, metabolism, and peroxidation; fatty acid metabolism; cell signalling; apoptosis	Preeclampsia [14]
Sphingosine 1-phosphate	Sphingolipids	Phosphosphingolipids	Sphingolipid metabolism; lipid transport, metabolism, and peroxidation; fatty acid metabolism; cell signalling	Late onset [12] preeclampsia
Vitamin D	Steroids and steroids derivatives	Vitamin D and derivatives	Lipid transport, metabolism, and peroxidation; fatty acid metabolism; cell signalling	Preeclampsia [21], late onset [12] preeclampsia, gestational hypertension [22]
Bolasterone	Steroids and steroids derivatives	Androstan steroids	Lipid transport, metabolism, and peroxidation; fatty acid metabolism; cell signalling	Preeclampsia [10]
		Organoheterocyclic compounds		
Caffeine	Imidazopyrimidines	Purines and purine derivatives	Caffeine metabolism	Early onset [23] preeclampsia
Paraxanthine	Imidazopyrimidines	Purines and purine derivatives	Caffeine Metabolism	Early onset [23] preeclampsia
5-Hydroxytryptophan	Indoles and derivatives	Tryptamines and derivatives	Tryptophan metabolism	Late onset [12] preeclampsia

For peer review only - http://bmjopen.bmj.com/site/about/guidelines.xhtml

Serotonin	Indoles and derivatives	Tryptamines and derivatives	Tryptophan metabolism; nicotine action pathway	Late onset [4] preeclampsia
Gama-butyrolactone	Lactones	Gamma butyrolactones	NA	Late onset [12] preeclampsia
N-methyl-2-pyridone-5-carboxamide	Pyridines and derivatives	Pyridinecarboxylic acids and derivatives	Nicotinate and Nicotinamide Metabolism	Preeclampsia [3]
N-methylnicotinamide	Pyridines and derivatives	Pyridinecarboxylic acids and derivatives	NA	Preeclampsia [3]
Oxolan-3-one	Dihydrofurans	Furanones	NA	Late onset [12] preeclampsia
		Organic oxygen compounds		
4-deoxyerythronic acid	Organooxygen compounds	Carbohydrates and carbohydrate conjugates	NA	Preeclampsia [3]
Galactose	Organooxygen compounds	Carbohydrates and carbohydrate conjugates	Galactose metabolism, nucleotide sugars metabolismo, lactose degradation	Preeclampsia [3]
Glycerol	Organooxygen compounds	Carbohydrates and carbohydrate conjugates	Glycerolipid metabolism, galactose metabolism, glycerolipid metabolism	Early onset [11] preeclampsia
Lactose	Organooxygen compounds	Carbohydrates and carbohydrate conjugates	Galactose metabolism, Phosphotransferase system, carbohydrate digestion and absorption	Preeclampsia [3]
Sucrose	Organooxygen compounds	Carbohydrates and carbohydrate conjugates	Starch and sucrose metabolism, galactose metabolism	Preeclampsia [3]
Acetone	Organooxygen compounds	Carbonyl compounds	Ketone body metabolism	Early onset [11] preeclampsia, late onset [4] preeclampsia
		Organic nitrogen compounds		
Scyllo-inositol	Organonitrogen compounds	Alcohols and polyols	Inositol phosphate metabolism	Preeclampsia [3]
Propylene glycol	Organooxygen compounds	Alcohols and polyols	Pyruvate metabolism	Late onset [4] preeclampsia

For peer review only

Propane-1,3-diol	Organooxygen compounds	Alcohols and polyols	Glycerolipid metabolism	Preeclampsia [20]
Putrescine	Organonitrogen compounds	Amines	Methionine metabolism, spermidine and spermine biosynthesis	Late onset [4] preeclampsia
Trimethylamine	Organonitrogen compounds	Amines	Methane and carbon metabolisms.	Preeclampsia [2,10]
Carnitine	Organonitrogen compounds	Quaternary ammonium salts	Beta oxidation of very long chain and branched chain fatty acids, mitochondrial beta-oxidation of saturated fatty acids	Preeclampsia [3], late onset [4,13] preeclampsia
Linoleylcarnitine	Organonitrogen compounds	Quaternary ammonium salts	Lipid transport, metabolism, and peroxidation; fatty acid metabolism; cell signalling	Early onset [16] preeclampsia, late onset [16] preeclampsia
Stearoylcarnitine	Organonitrogen compounds	Quaternary ammonium salts	Mitochondrial beta-oxidation of long chain saturated fatty acids	Early onset [16] preeclampsia, late onset [16] preeclampsia
Tetradecenoylcarnitine	Organonitrogen compounds	Quaternary ammonium salts	Lipid transport, metabolism, and peroxidation; fatty acid metabolism; cell signalling	Early onset [16] preeclampsia, late onset [16] preeclampsia
		Benzenoids		
Bisphenol A	Benzene and substituted derivatives	Diphenylmethanes	Bisphenol degradation	Preeclampsia [24]
Hippurate	Benzene and substituted derivatives	Benzoic acids and derivatives	Phenylalanine metabolism	Preeclampsia [3]
4-OH-hippurate	Benzene and substituted derivatives	Benzoic acids and derivatives	NA	Preeclampsia [3]
Mono(2- ethyl-5-oxohexyl) phthalate	Benzene and substituted derivatives	Benzoic acids and derivatives	NA	Late onset [24] preeclampsia
Mono(2-ethyl-5-carboxypentyl) phthalate	Benzene and substituted derivatives	Benzoic acids and derivatives	NA	Late onset [24] preeclampsia

For peer review only

Mono(2-ethylhexyl) phthalate	Benzene and substituted derivatives	Benzoic acids and derivatives	NA	Preeclampsia [24]
Mono(3-carboxypropyl) phthalate	Benzene and substituted derivatives	Benzoic acids and derivatives	NA	Preeclampsia [24]
Mono-ethyl phthalate	Benzene and substituted derivatives	Benzoic acids and derivatives	NA	Preeclampsia [24], late onset [24] preeclampsia
Monoisobutyl phthalate	Benzene and substituted derivatives	Benzoic acids and derivatives	NA	Preeclampsia [24]
Mono-*n*-butyl phthalate	Benzene and substituted derivatives	Benzoic acids and derivatives	NA	Late onset [24] preeclampsia
Σ di(2-ethylhexyl) phthalate	Benzene and substituted derivatives	Benzoic acids and derivatives	NA	Preeclampsia [24], Late onset [24] preeclampsia
2,6-Di-tert-butyl-4-hydroxymethylphenol	Benzene and substituted derivatives	Phenylpropanes	NA	Gestational hypertension [10]
		Organohalogen compounds		
Perfluorohexane sulfonate	Alkyl halides	Alkyl fluorides	NA	Preeclampsia [25]
		Alkaloids and derivatives		
Trigonelline	Alkaloids derived from nicotinic acid	Pyridine alkaloids	Nicotinate and nicotinamide metabolism	Preeclampsia [3]
Pilocarpine	NA	NA	Biosynthesis of alkaloids derived from histidine and purine	Gestational hypertension [10]
		Phenylpropanoids and polyketides		
Cerasinone	Flavonoids	O-methylated flavonoids	NA	Preeclampsia [10]
		Organosulfur compounds		
Dimethyl sulfone	Sulfonyls	Sulfones	NA	Preeclampsia [2]

NA = not available

For peer review only

References

1 Gong S, Sovio U, Aye ILMH, *et al.* Placental polyamine metabolism differs by fetal sex, fetal growth restriction, and preeclampsia. *JCI Insight* 2018;**3**:1–15. doi:10.1172/jci.insight.120723

2 Kenny LC, Broadhurst DI, Hong W, *et al.* NMR Profiling in Early Pregnancy Reveals a Metabolomic Signature the Predicts the Subsequent Development Preeclampsia. *Reprod Sci* 2013;**20**:2641A. doi:10.1177/1933719113482088

3 Diaz SO, Barros AS, Goodfellow BJ, *et al.* Second trimester maternal urine for the diagnosis of trisomy 21 and prediction of poor pregnancy outcomes. *J Proteome Res* 2013;**12**:2946–57. doi:10.1021/pr4002355

4 Bahado-Singh R, Poon LC, Yilmaz A, *et al.* Integrated Proteomic and Metabolomic prediction of Term Preeclampsia. *Sci Rep* 2017;**7**:1–10. doi:10.1038/s41598-017-15882-9

5 Odibo1 AO, Goetzinger KR, Odibo L, *et al.* First-trimester prediction of preeclampsia using metabolomic biomarkers: a discovery phase study. *Prenat Diagn* 2011;**31**:990–4.

6 Rijvers CAH, Marzano S, Winkens B, *et al.* Early-pregnancy asymmetric dimethylarginine (ADMA) levels in women prone to develop recurrent hypertension. *Pregnancy Hypertens* 2013;**3**:118–23. doi:10.1016/j.preghy.2013.01.001

7 Khalil AA, Tsikas D, Akolekar R, *et al.* Asymmetric dimethylarginine, arginine and homoarginine at 11-13 weeks gestation and preeclampsia: A case-control study. *J Hum Hypertens* 2013;**27**:38–43. doi:10.1038/jhh.2011.109

8 Sovio U, McBride N, Wood AM, *et al.* 4-Hydroxyglutamate is a novel predictor of pre-eclampsia. *Int J Epidemiol* 2020;**49**:301–11. doi:10.1093/ije/dyz098

9 Kuc S, Koster MPH, Pennings JLA, *et al.* Metabolomics profiling for identification of novel potential markers in early prediction of preeclampsia. *PLoS One* 2014;**9**. doi:10.1371/journal.pone.0098540

10 Harville EW, Li Y-YY-Y, Pan K, *et al.* Untargeted analysis of first trimester serum to reveal biomarkers of pregnancy complications: a case-control discovery phase study. *Sci Rep* 2021;**11**:3468. doi:10.1038/s41598-021-82804-1

11 Bahado-Singh RO, Syngelaki A, Akolekar R, *et al.* Validation of metabolomic models for prediction of early-onset preeclampsia. *Am J Obstet Gynecol* 2015;**213**:530.e1-530.e10. doi:10.1016/j.ajog.2015.06.044

12 Kenny LC, Broadhurst DI, Dunn W, *et al.* Robust early pregnancy prediction of later preeclampsia using metabolomic biomarkers. *Hypertension* 2010;**56**:741–9. doi:10.1161/HYPERTENSIONAHA.110.157297

13 Bahado-Singh RO, Syngelaki A, Mandal R, *et al.* Metabolomic determination of pathogenesis of late-onset preeclampsia. *J Matern Neonatal Med* 2017;**30**:658–64. doi:10.1080/14767058.2016.1185411

14 Dobierzewska A, Soman S, Illanes SE, *et al.* Plasma cross-gestational sphingolipidomic analyses reveal potential first trimester biomarkers of preeclampsia. *PLoS One* 2017;**12**:1–16. doi:10.1371/journal.pone.0175118

For peer review only

15 Bilodeau JF, Qin Wei S, Larose J, *et al.* Plasma F2-isoprostane class VI isomers at 12-18 weeks of pregnancy are associated with later occurrence of preeclampsia. *Free Radic Biol Med* 2015;**85**:282–7. doi:10.1016/j.freeradbiomed.2015.05.012

16 Koster MPH, Vreeken RJ, Harms AC, *et al.* First-Trimester serum acylcarnitine levels to predict preeclampsia: A metabolomics approach. *Dis Markers* 2015;**2015**. doi:10.1155/2015/857108

17 Chappell L. A longitudinal study of biochemical variables in women at risk of preeclampsia. *Am J Obstet Gynecol* 2002;**187**:127–36. doi:10.1067/mob.2002.122969

18 Shanmugalingam R, Wang X, Motum P, *et al.* The 15-Epilipoxin-A4 Pathway with Prophylactic Aspirin in PreventingPreeclampsia: A Longitudinal Cohort Study. *J Clin Endocrinol Metab* 2020;**105**. doi:10.1210/clinem/dgaa642

19 Kenny LC, Thomas G, Poston L, *et al.* Prediction of preeclampsia risk in first time pregnant women: Metabolite biomarkers for a clinical test. *PLoS One* 2020;**15**:1–19. doi:10.1371/journal.pone.0244369

20 Lee SM, Kang Y, Lee EM, *et al.* Metabolomic biomarkers in midtrimester maternal plasma can accurately predict the development of preeclampsia. *Sci Rep* 2020;**10**:16142. doi:10.1038/s41598-020-72852-4

21 Woodham PC, Brittain JE, Baker AM, *et al.* Midgestation maternal serum 25-hydroxyvitamin D level and soluble fms-like tyrosine kinase 1/placental growth factor ratio as predictors of severe preeclampsia. *Hypertension* 2011;**58**:1120–5. doi:10.1161/HYPERTENSIONAHA.111.179069

22 Ates S, Sevket O, Ozcan P, *et al.* Vitamin D status in the first-trimester: Effects of vitamin D deficiency on pregnancy outcomes. *Afr Health Sci* 2016;**16**:36–43. doi:10.4314/ahs.v16i1.5

23 Eichelberger KY, Baker AM, Woodham PC, *et al.* Second-trimester maternal serum paraxanthine, CYP1A2 activity, and the risk of severe preeclampsia. *Obstet Gynecol* 2015;**126**:725–30. doi:10.1097/AOG.0000000000001041

24 Cantonwine DE, Meeker JD, Ferguson KK, *et al.* Urinary concentrations of bisphenol A and phthalate metabolites measured during pregnancy and risk of preeclampsia. *Environ Health Perspect* 2016;**124**:1651–5. doi:10.1289/EHP188

25 Rylander L, Lindh CH, Hansson SR, *et al.* Per- and Polyfluoroalkyl Substances in Early Pregnancy and Risk for Preeclampsia: A Case-Control Study in Southern Sweden. *Toxics* 2020;**8**. doi:10.3390/toxics8020043

For peer review only

Studies	Patient selection		Index test	
	Was a consecutive or random sample of patients enrolled?	Did the study avoid inappropriate exclusions?	Were the index test results interpreted without knowledge of the results of the reference standard?	If a threshold was used, was it pre-specified?
Chappell LC et al, 2002	Yes	No	Unclear	Yes
Powe et al, 2010	Yes	Yes	Unclear	Yes
Kenny LC et al, 2010	Yes	Yes	Yes	Yes
Odibo AO et al, 2011	Yes	No	Unclear	Yes
Woodham PC et al, 2011	Yes	Yes	Unclear	Yes
Rijvers CAH et al, 2013	Unclear	Unclear	Unclear	Yes
Khalil AA et al, 2013	Yes	Yes	Yes	Yes
Diaz SO et al, 2013	Yes	Unclear	Unclear	Yes
Kenny LC et al, 2013	Unclear	Unclear	Unclear	Yes
Kuc S et al, 2014	Yes	Yes	Unclear	Yes
Wetta LA et al, 2014	Yes	Yes	Unclear	Yes
Bahado-Singh RO et al, 2015	Yes	Yes	Unclear	Yes
Eichelberger KY et al, 2015	Yes	Yes	Yes	Yes
Koster MPH et al, 2015	Yes	Yes	Unclear	Yes
Bilodeau JF et al, 2015	Yes	Yes	Unclear	Yes
Ates S et al, 2016	Yes	Yes	Unclear	Yes
Cantonwine DE et al, 2016	Yes	Yes	Unclear	Yes
Kiely M et al, 2016	Yes	Yes	Yes	Yes
Bahado-Singh RO et al, 2017	Yes	Yes	Unclear	Yes
Bahado-Singh RO et al, 2017b	Yes	Yes	Unclear	Yes
Dobierzewska A et al, 2017	Yes	Yes	Unclear	Yes
Ye Y et al, 2017	Yes	No	Yes	Yes
Gong S et al, 2018	Yes	Yes	Unclear	Yes
Tamblyn JA et al, 2018	Yes	Yes	Yes	Yes
Sovio U et al, 2020	Yes	Yes	Unclear	Yes
Huo X et al, 2020	Yes	No	Unclear	Yes
Rylander L et al, 2020	Yes	Unclear	Unclear	Yes

For peer review only

Lee SM et al, 2020	Unclear	Unclear	Unclear	Yes
Kenny L et al, 2020	Yes	Yes	Yes	Yes
Shanmugalingam R et al, 2020	Yes	No	Unclear	Yes
Harville EW et al, 2021	Yes	Unclear	Unclear	Yes

For peer review only

Risk of bias				
Reference standard			Flow and timing	
Is the reference standard likely to correctly classify the target condition?	Were the reference standard results interpreted without knowledge of the results of the index test?	Was there an appropriate interval between index test and reference standard?	Did all patients receive the same reference standard?	Were all patients included in the analysis?
Yes	Yes	Yes	Yes	Yes
Yes	Unclear	Yes	Yes	Yes
Yes	Yes	Yes	Yes	Yes
Yes	Unclear	Yes	Yes	Yes
Yes	Unclear	Yes	Yes	Yes
Yes	Unclear	Yes	Yes	Yes
Yes	Unclear	Yes	Yes	Yes
Yes	Unclear	Unclear	Yes	Yes
Yes	Unclear	Yes	Yes	Yes
Yes	Unclear	Yes	Yes	Yes
Yes	Yes	Yes	Yes	Yes
Yes	Unclear	Yes	Yes	Yes
Yes	Unclear	Yes	Yes	Yes
Yes	Unclear	Yes	Yes	Yes
Yes	Unclear	Yes	Yes	Yes
Yes	Unclear	Yes	Yes	Yes
Yes	Unclear	Unclear	Yes	No
Yes	Yes	Yes	Yes	Yes
Yes	Unclear	Yes	Yes	Yes
Yes	Unclear	Yes	Yes	Yes
Yes	Unclear	Yes	Yes	Yes
Yes	Unclear	Yes	Yes	Yes
Yes	Unclear	Yes	Yes	Yes
Yes	Yes	Yes	Yes	Yes
Yes	Unclear	Yes	Yes	Yes
Yes	Unclear	Yes	Yes	Yes
Yes	Unclear	Yes	Yes	No

For peer review only

Yes	Unclear	Yes	Yes	Yes
Yes	Yes	Yes	Yes	Yes
Yes	Unclear	Yes	Yes	No
Unclear	Unclear	Yes	Yes	Yes

For peer review only

Applicability concerns		
**Patient selection**	**Index test**	**Reference standard**
Are there concerns that the included patients do not match the review question?	Are there concerns that the index test, its conduct, or interpretation differ from the review question?	Are there concerns that the target condition as defined by the reference standard does not match the review
No	No	No
No	No	No
No	No	No
No	No	No
No	No	No
No	No	No
No	No	No
Yes	No	No
No	No	No
No	No	No
No	No	No
No	No	No
No	No	No
No	No	No
No	No	No
No	No	No
No	Yes	No
No	No	No
No	No	No
No	No	No
No	No	No
No	No	No
No	No	No
No	No	No
No	No	No
Yes	No	No
No	No	No

For peer review only

No	No	No
No	No	No
Yes	No	No
No	No	Yes

For peer review only

PRISMA 2009 Checklist

Prediction of pregnancy-related hypertensive disorders using metabolomics: a systematic review

Jussara Mayrink, Debora Farias Batista Leite, Maria Laura Costa, Guilherme M Nobrega, José Guilherme Cecatti

Section/topic	#	Checklist item	Reported on page #
**TITLE**			
Title	1	Identify the report as a systematic review, meta-analysis, or both.	1
**ABSTRACT**			
Structured summary	2	Provide a structured summary including, as applicable: background; objectives; data sources; study eligibility criteria, participants, and interventions; study appraisal and synthesis methods; results; limitations; conclusions and implications of key findings; systematic review registration number.	2
**INTRODUCTION**			
Rationale	3	Describe the rationale for the review in the context of what is already known.	4
Objectives	4	Provide an explicit statement of questions being addressed with reference to participants, interventions, comparisons, outcomes, and study design (PICOS).	5
**METHODS**			
Protocol and registration	5	Indicate if a review protocol exists, if and where it can be accessed (e.g., Web address), and, if available, provide registration information including registration number.	5
Eligibility criteria	6	Specify study characteristics (e.g., PICOS, length of follow-up) and report characteristics (e.g., years considered, language, publication status) used as criteria for eligibility, giving rationale.	6
Information sources	7	Describe all information sources (e.g., databases with dates of coverage, contact with study authors to identify additional studies) in the search and date last searched.	6
Search	8	Present full electronic search strategy for at least one database, including any limits used, such that it could be repeated.	6
Study selection	9	State the process for selecting studies (i.e., screening, eligibility, included in systematic review, and, if applicable, included in the meta-analysis).	7
Data collection process	10	Describe method of data extraction from reports (e.g., piloted forms, independently, in duplicate) and any processes for obtaining and confirming data from investigators.	7
Data items	11	List and define all variables for which data were sought (e.g., PICOS, funding sources) and any assumptions and simplifications made.	6
Risk of bias in individual studies	12	Describe methods used for assessing risk of bias of individual studies (including specification of whether this was done at the study or outcome level), and how this information is to be used in any data synthesis.	7

For peer review only

PRISMA 2009 Checklist

Prediction of pregnancy-related hypertensive disorders using metabolomics: a systematic review

Jussara Mayrink, Debora Farias Batista Leite, Maria Laura Costa, Guilherme M Nobrega, José Guilherme Cecatti

Summary measures	13	State the principal summary measures (e.g., risk ratio, difference in means).	
Synthesis of results	14	Describe the methods of handling data and combining results of studies, if done, including measures of consistency (e.g., I^2) for each meta-analysis.	

Section/topic	#	Checklist item	Reported on page #
Risk of bias across studies	15	Specify any assessment of risk of bias that may affect the cumulative evidence (e.g., publication bias, selective reporting within studies).	
Additional analyses	16	Describe methods of additional analyses (e.g., sensitivity or subgroup analyses, meta-regression), if done, indicating which were pre-specified.	
**RESULTS**			
Study selection	17	Give numbers of studies screened, assessed for eligibility, and included in the review, with reasons for exclusions at each stage, ideally with a flow diagram.	8
Study characteristics	18	For each study, present characteristics for which data were extracted (e.g., study size, PICOS, follow-up period) and provide the citations.	10-14
Risk of bias within studies	19	Present data on risk of bias of each study and, if available, any outcome level assessment (see item 12).	
Results of individual studies	20	For all outcomes considered (benefits or harms), present, for each study: (a) simple summary data for each intervention group (b) effect estimates and confidence intervals, ideally with a forest plot.	10-14
Synthesis of results	21	Present results of each meta-analysis done, including confidence intervals and measures of consistency.	
Risk of bias across studies	22	Present results of any assessment of risk of bias across studies (see Item 15).	
Additional analysis	23	Give results of additional analyses, if done (e.g., sensitivity or subgroup analyses, meta-regression [see Item 16]).	
**DISCUSSION**			
Summary of evidence	24	Summarize the main findings including the strength of evidence for each main outcome; consider their relevance to key groups (e.g., healthcare providers, users, and policy makers).	15-18
Limitations	25	Discuss limitations at study and outcome level (e.g., risk of bias), and at review-level (e.g., incomplete retrieval of identified research, reporting bias).	19
Conclusions	26	Provide a general interpretation of the results in the context of other evidence, and implications for future research.	19, 20
**FUNDING**			

For peer review only - http://bmjopen.bmj.com/site/about/guidelines.xhtml

PRISMA 2009 Checklist

Prediction of pregnancy-related hypertensive disorders using metabolomics: a systematic review

Jussara Mayrink, Debora Farias Batista Leite, Maria Laura Costa, Guilherme M Nobrega, José Guilherme Cecatti

Funding	27	Describe sources of funding for the systematic review and other support (e.g., supply of data); role of funders for the systematic review.	27

From: Moher D, Liberati A, Tetzlaff J, Altman DG, The PRISMA Group (2009). Preferred Reporting Items for Systematic Reviews and Meta-Analyses: The PRISMA Statement. PLoS Med 6(7): e1000097. doi:10.1371/journal.pmed1000097

For more information, visit: www.prisma-statement.org.

For peer review only

For peer review only - http://bmjopen.bmj.com/site/about/guidelines.xhtml

BMJ Open

Prediction of pregnancy-related hypertensive disorders using metabolomics: a systematic review

Journal:	*BMJ Open*
Manuscript ID	bmjopen-2021-054697.R1
Article Type:	Original research
Date Submitted by the Author:	08-Mar-2022
Complete List of Authors:	Mayrink, Jussara; State University of Campinas Faculty of Medical Sciences, Obstetrics and Gynecology<br>Leite, Debora; State University of Campinas Faculty of Medical Sciences, Obstetrics and Gynecology<br>Nobrega, Guilherme; State University of Campinas Faculty of Medical Sciences, Department of Obstetrics and Gynecology<br>Costa, Maria Laura; State University of Campinas Faculty of Medical Sciences, Obstetrics and Gynecology<br>Cecatti, Jose; State University of Campinas Faculty of Medical Sciences, Obstetrics and Gynecology
**Primary Subject Heading**:	Obstetrics and gynaecology
Secondary Subject Heading:	Diagnostics
Keywords:	Hypertension < CARDIOLOGY, Maternal medicine < OBSTETRICS, EPIDEMIOLOGY

SCHOLARONE™
Manuscripts

I, the Submitting Author has the right to grant and does grant on behalf of all authors of the Work (as defined in the below author licence), an exclusive licence and/or a non-exclusive licence for contributions from authors who are: i) UK Crown employees; ii) where BMJ has agreed a CC-BY licence shall apply, and/or iii) in accordance with the terms applicable for US Federal Government officers or employees acting as part of their official duties; on a worldwide, perpetual, irrevocable, royalty-free basis to BMJ Publishing Group Ltd ("BMJ") its licensees and where the relevant Journal is co-owned by BMJ to the co-owners of the Journal, to publish the Work in this journal and any other BMJ products and to exploit all rights, as set out in our licence.

The Submitting Author accepts and understands that any supply made under these terms is made by BMJ to the Submitting Author unless you are acting as an employee on behalf of your employer or a postgraduate student of an affiliated institution which is paying any applicable article publishing charge ("APC") for Open Access articles. Where the Submitting Author wishes to make the Work available on an Open Access basis (and intends to pay the relevant APC), the terms of reuse of such Open Access shall be governed by a Creative Commons licence – details of these licences and which Creative Commons licence will apply to this Work are set out in our licence referred to above.

Other than as permitted in any relevant BMJ Author's Self Archiving Policies, I confirm this Work has not been accepted for publication elsewhere, is not being considered for publication elsewhere and does not duplicate material already published. I confirm all authors consent to publication of this Work and authorise the granting of this licence.

For peer review only

SYSTEMATIC REVIEW

Prediction of pregnancy-related hypertensive disorders using metabolomics: a systematic review

Jussara Mayrink, MD, PhD [1,2] (0000-0001-5957-4365; jussaramayrink@gmail.com)
Debora Farias B. Leite, MD, PhD [1,3] (0000-0001-8839-3934; debora.leite@ufpe.br)
Guilherme Moraes Nobrega, BSc [1] (0000-0001-9406-4256; guigo.nobrega@hotmail.com)
Maria Laura Costa, MD, PhD [1] (0000-0001-8280-3234; mlaura@unicamp.br)
José Guilherme Cecatti, MD, PhD [1] (0000-0003-1285-8445; cecatti@unicamp.br)

[1]Department of Obstetrics and Gynecology, University of Campinas, School of Medicine, Brazil
[2]Federal University of Minas Gerais, Belo Horizonte, Minas Gerais, Brazil.
[3]Department of Gynecology and Obstetrics, Federal University of Pernambuco, Recife, Pernambuco, Brazil.

Correspondence:
José Guilherme Cecatti
Rua Alexander Fleming, 101 Cidade Universitaria Zeferino Vaz.
13083-889. Campinas, Sao Paulo, Brazil.
Telephone: +55 19-35219482
Email: cecatti@unicamp.br

Word Count:
Abstract: 300 words
Main text: 5,144 words

For peer review only - http://bmjopen.bmj.com/site/about/guidelines.xhtml
For peer review only

Abstract

Objective: To determine metabolomics accuracy in predicting hypertensive disorders in pregnancy.

Design: systematic review on test accuracy.

Data sources and study eligibility criteria: An electronic literature search was performed in June, 2019 and February, 2022. Two researchers independently selected studies published since 1998 to 2022 on metabolomics' techniques applied to predict the condition; subsequently, they extracted data and performed quality assessment. Discrepancies were dealt by a third reviewer. The primary outcome was preeclampsia. Cohort or case-control studies were eligible when maternal samples were taken before the diagnosis of the hypertensive disorder.

Study appraisal and synthesis methods: Data on study design, maternal characteristics, how hypertension was diagnosed, metabolomics details and metabolites, and accuracy were independently extracted by two authors.

Results: Among 4,613 initially identified studies on metabolomics, 68 were read in full text and 32 articles were included. Studies were excluded due to duplicated data, study design or lack of identification of metabolites. Metabolomics was applied mainly in the 2nd trimester; the liquid-chromatography coupled to mass spectrometry was the most common technique. Among the 122 different metabolites found, there were 23 amino acids and 21 fatty acids. Most of metabolites were involved with ammonia recycling; amino acid metabolism; arachidonic acid metabolism; lipid transport, metabolism, and peroxidation; fatty acid metabolism; cell signalling; galactose metabolism; nucleotide sugars metabolism; lactose degradation; and glycerolipid metabolism. Only citrate was a common metabolite for prediction of early- and late-onset preeclampsia. Vitamin D was the only metabolite in common for preeclampsia and gestational hypertension prediction. The meta-analysis was not performed due to lack of appropriate standardized data.

Conclusions and Implications: Metabolite signature may contribute to further insights into the pathogenesis of preeclampsia and support screening tests. Nevertheless, it is mandatory to validate the method in larger studies with a heterogeneous population to ascertain its use in clinical practice.

PROSPERO registration: CRD42018097409.

For peer review only - http://bmjopen.bmj.com/site/about/guidelines.xhtml

Keywords: preeclampsia, systematic review, hypertension, metabolomics, metabolome, prediction.

Strengths and limitations of this study

- This systematic review present possible biomarkers of preeclampsia that could ground future research in this area.
- There was great heterogeneity among included studies due to complexity of metabolomics procedures, which are influenced by sample collection, storage and preparation, analytical platform applied, and statistical tests performed.
- Our findings must be analysed with caution due to the lack of standardization of preeclampsia definition (early and late-onset preeclampsia) and time of sample collection in pregnancy.

Original protocol: Mayrink J, Leite DFB, Costa ML, *et al*. Metabolomics for prediction of hypertension in pregnancy: a systematic review and meta-analysis protocol. *BMJ Open* 2020;10:e040652. doi:10.1136/bmjopen-2020-040652.

For peer review only - http://bmjopen.bmj.com/site/about/guidelines.xhtml

INTRODUCTION

Hypertensive disorders have been reported in association to adverse outcomes in pregnancy since years before Christ. They are a heterogeneous group of conditions that include chronic hypertension, preeclampsia, gestational hypertension and preeclampsia superimposed on chronic hypertension.[1]

Chronic hypertension is the condition of Systolic blood pressure ≥140 mmHg and/or Diastolic blood pressure ≥ 90 mmHg before twenty weeks of gestation or even before pregnancy. [2] Gestational Hypertension is the isolated elevation of pressure after twenty weeks of gestation. It is less damaging than preeclampsia but around 30% of these cases progress with preeclampsia.[3] The most prevalent form - preeclampsia - is a serious potentially-life threatening condition to the mother and the baby, [2] and its high prevalence rate persists along the years, ranging from 3 to 10% of all pregnancies, according to the region studied. Unfortunately, treatment has been the same over decades and relies on the management of clinical features, such as new onset of hypertension and end-organ dysfunction, that characterizes preeclampsia. Outcomes mostly depend upon accurate diagnosis and decision on timing of childbirth since there are exiguous predictive tools available. [3] As a result, the scientific race is to identify a group of pregnant women at high-risk for the disease, who would be candidates for prophylactic and increased surveillance measures. The increased understanding on pathophysiology would also shed light into possible new therapeutic approaches.

Several biomarkers have been studied as predictive tools for preeclampsia, e.g. soluble fms-like tyrosine kinase-1, soluble endoglin, markers of apoptosis and inflammation, placental protein 13, C-reactive protein, and markers of placental hypoxia and distress. [4] However, none are sufficiently sensitive or specific to predict preeclampsia in advance.

Recently, novel technologies, such as metabolomics, have been applied to predict preeclampsia. Metabolomics is known as the newest member of the “omics” family. Metabolome - a collection of metabolites – is defined and used in research to pursue

For peer review only - http://bmjopen.bmj.com/site/about/guidelines.xhtml

the phenotypic signature of a disease of interest. Metabolites are low-molecular-weight chemicals (<1500 Da) resulting from changes in gene, protein expression [5] and environmental interferences. Metabolomics methods include hydrogen nuclear magnetic resonance spectroscopy (^{1}H-NMR) and gas- and liquid- chromatography-mass spectrometry (GC- and LC-MS, respectively). [5,6] Metabolomics has been applied to improve disease biomarkers and understand the pathogenesis of many conditions, such as cancer and diabetes. [7,8] One could argue its value in evaluating preeclampsia, which has a complex aetiology and is considered a multifactorial disease. Metabolomics, however, a high throughput technique, seems perfectly adequate, since it can simultaneously encompass a wide range of metabolic pathways. The objective of this systematic review was to determine the accuracy of metabolomics in predicting pregnancy-induced hypertensive disorders.

METHODS

This systematic review was conducted based on our previously published protocol [9] (CRD42018097409) and is reported in accordance with the Preferred Reported Items for Systematic Reviews and Meta-Analysis (PRISMA) statement; [10] a PRISMA checklist is provided as a Supplementary Material 1. Our research question was: "what is the performance of metabolomics for predicting gestational hypertensive disorders?"

Population and Interventions

Our domain of interest was any form of hypertensive disorder developed during pregnancy, in single or multiple gestations. Metabolomics was the intervention studied; then, metabolites that differentiate hypertensive from normotensive pregnant women should have been mentioned. Maternal samples should have been drawn during pregnancy and before diagnosis of pregnancy-induced hypertensive disorder. We excluded studies in which blood samples were collected after the diagnosis of hypertension was established, or when preeclampsia and gestational hypertension were analysed as the same outcome.

For peer review only - http://bmjopen.bmj.com/site/about/guidelines.xhtml

Comparison and Outcomes

We included studies in which women with any form of pregnancy-induced hypertension were compared to pregnant women without hypertension. Preeclampsia was the primary outcome, diagnosed by criteria defined by the authors. [11–15]

Secondary outcomes were: a) early-onset preeclampsia, characterized by delivery <34 weeks due to preeclampsia; [3] b) late-onset preeclampsia, requiring delivery ≥34 weeks; [3] c) gestational hypertension, when hypertension is the only clinical finding after 20 weeks gestation; d) preeclampsia superimposed on chronic hypertension; e) white coat hypertension, i.e., normal blood pressure recorded in a 24h ambulatory monitoring; f) masked hypertension, characterized as normal blood pressure in the office or clinic and altered under other circumstances; and g) transient gestational hypertension, higher blood pressure at levels during a certain period that is later normalized after repeated blood pressure readings. [11,16]

Search strategy

Two independent researchers (JM, DFBL) performed an electronic database search in PubMed, EMBASE, Scopus, Web of Knowledge, Latin American and Caribbean Health Sciences Literature (LILACS), Scientific Electronic Library Online (SciELO), Health Technology Assessment (HTA), and Database of Abstracts of Reviews of Effects (DARE), including studies published from 1998 to 2022. The full search strategies are provided as Supplementary Material 2. We have applied no filters, except for SCOPUS (we have used the Title, Abstract and Keyword filter). The grey literature (conference abstracts) and the reference list of included studies were searched for additional articles for inclusion.

There were no language restrictions; only cohort and case control studies were included. Letters to the editor, editorials, comments, expert opinions, any type of review, experimental animal studies, case reports, intervention trials, and cross-sectional studies were all excluded. We intend to show the capacity of metabolomics

For peer review only

to predict hypertensive disorders without interference of any intervention. Thus, it is absolutely necessary to guarantee the temporal relation between timing of sample (before) and outcome (after). This is the reason for including just cohort and case control studies.

Data extraction and quality assessment

Trained reviewers (JM, DFBL) proceeded studies' screening (using EndNote® software), data extraction and synthesis, and risk of bias evaluation (Quality Assessment of Diagnostic Accuracy Studies (QUADAS-2) [17]). Disagreements were solved by a third reviewer (MLC).

Summary of data and meta-analysis

For all included studies, we have extracted data on research characteristics and metabolomics details. If the study comprised training and validation datasets, we have extracted metabolomics data from the validation group of women. When the study has not definitively identified a given metabolite, we have extracted all possible metabolites. We have extracted data of metabolites combined with other biomarkers, when available. We have used the Human Metabolome Database [18], the Kyoto Encyclopaedia of Genes and Genomes [19] and the Lipid Maps [20] for matching each metabolite chemical class and subclass, and metabolic pathways and processes.

When available, data on accuracy measures on metabolomics performance in predicting hypertensive disorders of pregnancy were extracted. We planned to perform the estimation of likelihood ratios and hierarchical summary receiver operator characteristic curve, [21] and the assessment of heterogeneity and publication bias. [22]

RESULTS

Main findings

The literature search for this systematic review was performed in June 2019 with a further re-run in February 2022. **Figure 1** summarizes the flow chart of studies'

For peer review only - http://bmjopen.bmj.com/site/about/guidelines.xhtml

selection and final inclusion. We excluded studies (Supplementary Material 3) due to study design (25 studies) or lack of identification of metabolites (05 studies). Regarding duplicated data, only the most recent and complete study was included (06 studies). .

Overall, 32 studies were selected: 25 case control studies [23–47] and seven cohort studies [48–54] (**Table 1**). Studies from the same research project but evaluating different metabolites [43,46] or populations [24,28,29,33,36,38,39] were included. Most studies were developed in Europe [23,25,28,29,31,33,36,38,39,42,43,46,48–52] and North America; [32,34,35,40,41,44,45,47,54] there was only one research from Latin America, [26] two from Oceania [24,53] and another three from Asia. [27,30,37] Twelve studies have sampled maternal specimens in the 1st trimester, [31,32,34,35,42,43,46,48,50–53] 15 studies have sampled in the 2nd trimester, [23,24,27,29,30,33,36–39,41,44,45,49,54] four studies have sampled at least two times [25,26,40,47] in pregnancy, and one study included women in late 3rd trimester [28] (**Figure 2**). Four studies [24,33,38,40] have performed training and validation with different populations.

Studies have defined preeclampsia as hypertension developed in pregnancy associated with proteinuria [11,13–15] or end-organ dysfunction. [12] Although not consistently, chronic hypertension or other illnesses, [24,27,29,33,36–39,41,45,55] and multiple pregnancies [25,27,30,32,33,36,37,39,41,42,45,52] were reasons for participants exclusion. When chronic hypertensive women were included, there were women taking aspirin as prophylaxis. [23,53] Then, there are no specific data on superimposed preeclampsia or multiple pregnancies. Similarly, there are no studies on white coat hypertension, masked hypertension, or transient gestational hypertension. Only three studies have brought data on gestational hypertension. [32,37,51] Small for gestational age (SGA) infants were fully excluded [31] or included only in preeclampsia group, either because they were excluded from control groups, [23,34] or because this variable was not used as for matching cases and controls. [24,29,36]

In **Table 2**, characteristics of the included studies regarding metabolomics techniques and accuracy measures are shown according to the primary and secondary outcomes. Majority of studies have not presented data according to gestational age of delivery.

For peer review only - http://bmjopen.bmj.com/site/about/guidelines.xhtml

The most common technique was the LC-MS, [24,26,27,29,31,33–38,40,41,43–48,51,53,55] followed by H-NMR [25,39,49,50,52] and GC-MS. [23,42] Bahado-Singh et al. [25] (H-NMR followed by LC-MS), Bilodeau et al. [54] (HPLC-MS/MS and GC-FID), Lee et al. [30] (GC-MS and LC-MS), and Harville et al. [32] (LC-MS and NMR) have used two complementary techniques. Most studies analysed blood samples, [23,24,26–28,30–46,48,50–54] two studies analysed urine [47,49] and one study analysed both, urine and blood [29] (**Figure 2**). There were no studies analysing maternal hair or amniotic fluid. All studies that used H-NMR [25,32,39,49,50,52] and four that applied LC-MS [24,28,32,38] were untargeted evaluations. Eleven studies reported lipidomic analysis and/or amino acid analysis. [23,26,30,35,40,42,43,46,48,53,54] Six studies described vitamin D analysis, all of them using LC-MS.[29,34,36,41,44,51] Five studies reported environmental exposures, focusing on caffeine, [45] bisphenol A, [27] phthalates [47] and per-and polyfluoroalkyl substances (PFAS). [31,37] Two studies have looked for differences in preeclampsia prediction according to foetal sex. [28,47] There were five metabolites in common between preeclampsia and SGA prediction (sphinganine 1-phosphate, sphingosine 1-phosphate, tyrosine, vitamin D, N1, N12-diacetylspermine). [56]

For preeclampsia requiring delivery at any gestational age, the lowest area under the curve (AUC) was for isoprostane 8-epi-prostaglandin F2α (0.55), [23] and the highest (AUC 0.95) was for a model composed by cerasinone, bolasterone, and two unidentified metabolites, presented as retention time and mass (6.30_477.7721m/z, 5.86_313.1070m/z).[32] For early onset preeclampsia, the AUC of untargeted metabolomics in 1st trimester maternal blood varied from 0.673 (UPLC-MS/MS) [38] to 0.835 (H-NMR). [50] For late onset preeclampsia, the AUC of untargeted metabolomics in 1st trimester maternal blood was 0.629 (H-NMR); [52] in 2nd trimester, 0.92 (UPLC-MS); [24] and in 3rd trimester, 0.761 (H-NMR and LC-MS/MS). [25]

When analysed together with non-metabolomics biomarkers (**Table 3**), the best performance was achieved by taurine combined with maternal prior risk and mean arterial blood pressure (MAP) (AUC 0.93 in 1st trimester for early onset preeclampsia); [43] followed by 3-hydroxyisovalerate, arginine, glycerol combined with uterine

For peer review only - http://bmjopen.bmj.com/site/about/guidelines.xhtml

arteries pulsatility index (UtPI) (AUC 0.917 in 1st trimester for early onset preeclampsia) [50]; and leptin/ceramide(d18:1/25:0) ratio in in 1st and 2nd trimester (AUC 0.876 and 0.892, respectively) for preeclampsia at any gestational age. [40]

All predictive metabolites of hypertensive disorders of pregnancy according to chemical class and subclass, and metabolic pathways possibly involved are presented as Supplementary Material 4. Ten metabolites could not be found in the metabolome databases (U4, U10, U14, U35, [49] hexadecenoyleicosatetraenoyl-Sn-glycerol; [24] SM C28:1, SM C30:1, LysoPC C19:0 [30], dibenzylamine [32], perfluoroheptanoic acid [37]). There were 121 different metabolites, from ten different super classes. Majority of metabolites corresponded to amino acids, peptides, and analogues (23 compounds); fatty acid esters (11 compounds); fatty acids and conjugates (10 compounds); and carbohydrates and carbohydrate conjugates (5 compounds). They are mainly involved with ammonia recycling; amino acid metabolism; arachidonic acid metabolism; lipid transport, metabolism, and peroxidation; fatty acid metabolism; cell signalling; galactose metabolism; nucleotide sugars metabolism; lactose degradation; and glycerolipid metabolism. There were 10 benzoic acids and derivatives, but only hippurate has a known pathway. Citrate was found in common for prediction of preeclampsia in any gestational age, [49] early [50] and late-onset [25] disease. Octenoylcarnitine, [46] octanoylcarnitine, [46] acetone, [25,50] linoleylcarnitine, [46] stearoylcarnitine, [46] tetradecenoylcarnitine [46] were predictive for both early and late preeclampsia. Vitamin D was the only metabolite involved simultaneously with preeclampsia, [41] late onset [24] preeclampsia, and gestational hypertension. [51]

Summary of included studies

The untargeted studies provided a great number of predictive metabolites. Kenny et al. showed a decrease of 13 metabolites (NMR) [39] and an increment of another 40 metabolites (LC-MS) [24] in blood analysis between 14-16 weeks of nulliparous pregnant women exclusively. In a validation phase with a list of 14 different metabolites, the odds ratio of developing preeclampsia, at any gestational age, was 23 (95% CI: 7-73), with AUC 0.92. [24] These same metabolites were validated in a

For peer review only - http://bmjopen.bmj.com/site/about/guidelines.xhtml

different population, [33] and the dilinoleoyl-glycerol has achieved an AUC 0.70 (0.59-0.82).

Bahado-Singh et al. applied metabolomics to predict early- and late-onset cases. [25,50,52] Using maternal 1st trimester blood (11-13w), those authors reinforced the theory of the multiple phenotypes for preeclampsia. [57,58] 2-hydroxybutyrate was identified as predictor of early-onset preeclampsia, [50] whereas carnitine, pyruvate and acetone resulted in an AUC of 0.629 for late-onset disease. This latter AUC increased to 0.734 by combining metabolites and uterine Doppler velocimetry and maternal weight. [52] Finally, in a two-step evaluation in the 1st and 3rd trimester to predict term preeclampsia (≥37w), a combination of metabolomics and proteomics yielded an AUC of 0.817. [25]

Through an untargeted urinary metabolome analysis, Diaz et al. [49] showed decreased levels of acetate, formate, fumarate (all involved with the Krebs cycle), succinate, and isoleucine; and increased levels of 4-deoxyerythronic acid, a degradation product of 3-hydroxybutyrate [49] between 14-26w. Finally, still working with an untargeted approach, Sovio et al. demonstrated an increase of 4-hydroxyglutamate serum levels among women who developed early-onset preeclampsia, measured at 12w. [38] Its prediction performance was improved by addition of pregnancy-associated plasma protein A (PAPP-A) and PlGF. [38]

Regarding lipidomic or amino-acid studies, Odibo et al. adjusted four metabolites (hydroxyhexanoylcarnitine, phenylalanine, glutamate, alanine) for maternal variables (maternal body mass index, ethnicity, and diabetes) and the AUCs were similar with the four-metabolite model (0.82) or when only the three amino acids were used for predicting preeclampsia (0.81). Additionally, the four metabolites have demonstrated equal AUCs for predicting preeclampsia at any gestational age (0.82) or in cases of early onset disease (0.84). [35]In the 1st trimester, Shanmugalingam et al. [53] showed lower levels of 15-epilipoxin-A4, which is possibly involved with the aspirin mechanism of preeclampsia prophylaxis. Chappell et al. showed an increment in 8-epi-prostaglandin F2α from 20 to 24 weeks in the preeclampsia group. [23] Recently, Bilodeau et al. reinforced that F2-isoprostanes isomers from class VI were increased in

For peer review only - http://bmjopen.bmj.com/site/about/guidelines.xhtml

the preeclampsia group. [54] The prostaglandins' metabolites are markers of lipid peroxidation, which pointed to an oxidative stress status, but presented modest accuracy. [23,54]

Rijvers et al. [48] and Khalil et al. [42] studied the metabolism of asymmetric dimethylarginine (ADMA), which is considered an important inhibitor of nitric oxide action. Rijvers et al. found an increase of L-arginine/ADMA ratio at 12w in the preeclampsia group, [48] and a consistent decrease in L-arginine between 16w and 20w in the preeclampsia compared to the gestational hypertension group. This can be clinically relevant since L-arginine supplementation is possible. [59] Khalil et al. found lower levels of L-arginine and L-homoarginine at 11-13w in the early-onset preeclampsia, and unchanged levels in the late-onset group, even after the excluding chronic hypertensive women. [42]

Koster et al. [46] showed that stearoylcarnitine was a common biomarker for both early-onset and late-onset preeclampsia. In a model composed of stearoylcarnitine, prior risk and MAP, the detection rate improved by 45% and 21%, for early and late-onset cases of preeclampsia, respectively. Dobierzewska et al. [26] have studied sphingolipids that have been related to trophoblast differentiation and invasion. In the ceramide profile, the Cer 14:0 was decreased in both 1st and 3rd trimesters of preeclampsia group, while Cer 16:0 was significantly increased in cross-gestational preeclampsia plasma samples between 1st and 2nd trimesters. Plasma levels of dihydro-sphingosine-1 decreased from the 1st to the 2nd trimester among the preeclampsia group, and SM 18:0 increased through gestation of these participants. [26] Still studying ceramides, Huang et al [40] have found decreasing levels of Ceramide(d18:1/25:0) from 5-29 weeks, which increased the Leptin/ Ceramide(d18:1/25:0) ratio. Lee SM et al. proposed a model constituted by SM C28:1, SM C30:1, LysoPC C19:0, LysoPE C20:0 and propane-1,3-diol, achieving an AUC of 0.868 for identifying preeclampsia. [30]

Gong et al. [28] analysed serum samples in different moments of pregnancy, but it was at 36w when N1, N12 diacetylspermine were higher in women who later present preeclampsia, especially in the case of a female fetus. [28] This was the only study in

For peer review only - http://bmjopen.bmj.com/site/about/guidelines.xhtml

common with our previous systematic review about SGA infant's prediction using metabolomics. [56]

Regarding targeted vitamin D analysis, all studies have applied LC-MS. [29,34,36,41,44,51] In general, they found lower serum vitamin D concentrations in women who later developed preeclampsia, both in blood [36] and urine, [29] suggesting that dysregulation of vitamin D metabolism occurs early in pregnancy. Indeed, Woodham et al. have pointed reduced odds of 38% of developing severe preeclampsia for a 10nmol/L increase in 25(OH)D level between 15-20w. [41] However, results did not reach statistical significance when a threshold was applied. In nested case-controls [34,44] or cohort [51] studies there were no differences between vitamin D status among preeclampsia group and controls. In the model proposed by Woodham et al. combining 25OHD, VEGF and sFLT-1/PlGF ratio, the AUC was 0.851, a higher value than either marker alone. [41] Vitamin D3 derivatives were found in the untargeted analysis of Kenny et al., [24] but upregulated in preeclampsia cases. [41]

Two studies have evaluated maternal exposure to bisphenol A and its metabolites. [27,47] The di(2-ethylhexyl) phthalate was increased in all trimesters. [47] Its hazard ratio for development of preeclampsia varied from 1.79 (95% CI 1.30-1.52), with 4-16 weeks, to 2.92 (95% CI 1.61-5.28) with 33-38w. The mono(2-ethyl-5-oxohexyl) phthalate, the mono(2-ethyl-5-carboxypentyl) phthalate and the Σdi(2-ethylhexyl) phthalate were associated to increased hazard ratios for preeclampsia when evaluated in urine [47] from both the 1st and the 2nd half of pregnancy. When the findings were analysed according to fetal sex, exposure to bisphenol and di(2-ethylhexyl) phthalate was only associated to preeclampsia in pregnancies of female fetuses.[47] In maternal serum, [27] free bisphenol was higher in preeclamptic women compared to normotensive controls, even when superimposed preeclampsia cases were excluded. The cut-off of >4.4 μg/L was associated with increased odds of 16.46 (95% CI 5.42-49.95) of developing preeclampsia (adjusted for maternal age, parity, and body mass index). [27]

In another analysis of environmental factors, Eichelberger et al. found no differences in paraxanthine or caffeine levels, but the paraxanthine/caffeine ratio was lower in

For peer review only - http://bmjopen.bmj.com/site/about/guidelines.xhtml

preeclampsia (0.23 versus; normotensive women, 0.37, *p* 0.02). [45] This represented a decreased risk of developing preeclampsia with increasing paraxanthine/caffeine ratio (odds ratio 0.53, 95%CI 0.31-0.90). Finally, regarding the perfluoroalkyl substances (PFASs), Rylander et al. [31] and Huo et al. [37] have found no differences between preeclamptic women and normotensive controls in blood samples from the 1st or 2nd trimester, respectively. However, levels of perfluorohexane sulfonate between 0.53-0.78ng/mL were associated to higher odds of preeclampsia (aOR 1.67, 95% CI1.02-2.74); [31] and of perfluoroheptanoic acid, to higher odds of gestational hypertension (aOR 1.38; 95%CI 1.01-1.87).

In the last study to evaluate gestational hypertension, Harville et al. [32] identified higher concentrations of 2,6-Di-tert-butyl-4-hydroxymethylphenol in blood samples between 6-14w. This metabolite is derived from 2,6-Di-tert-butyl-4-methylphenol, a synthetic phenolic antioxidant used widely in foods, polymers, and cosmetics to slow oxidation. It has been linked to induce cellular DNA damage.

Quality assessment and risk of bias

The synthesized data for all included studies is shown on **Figure 3**. Regarding the risk of bias, majority of studies were ranked as low for 'Patient Selection' and 'Flow and Timing' domains. On the contrary, most studies were classified as unknown risk of bias in 'Index Test' and 'Reference Standard' domains due to the missing information if the diagnosis of preeclampsia (reference standard) and the metabolomics analysis (index test) were interpreted without the knowledge of each other. Regarding applicability, virtually all studies were ranked as low applicability concerns, with minor exceptions. [32,47,49,53,60] (Please, check the Supplementary Material 5 for individual QUADAS-2 data).

Meta-analysis

We found that a third of included studies were designed with predictive purposes, [23–25,27,35,38,41,50,52] and we extracted accuracy measures when possible (**Tables 2 and 3**). The other studies have only presented statistical differences between groups. Unfortunately, there were discrepancies regarding type of maternal sample or

For peer review only
For peer review only - http://bmjopen.bmj.com/site/about/guidelines.xhtml

gestational age at sampling, and metabolomics technique. Then, it was not possible to perform a meta-analysis.

DISCUSSION

Hypertensive disorders constitute one of the leading causes of maternal morbidity and mortality worldwide. In this systematic review, we have found that amino acids and fatty acids were the most common chemical subclasses associated to preeclampsia. Similarly, the main pathways involved in preeclampsia were lipid metabolism, mitochondrial beta-oxidation of short chain saturated fatty acids, and cell signalling (**Figure 4**). Our findings can improve disease understanding and contribute to future studies as a guide of pathways to be explored with the purpose to better treat the disease.

Amino acids and related compounds

Amino acids, peptides, and analogues were the most common chemical subclass. Taurine, glycine and glycylglycine were decreased in women who developed preeclampsia. [39,43] Taurine's function is associated with cytoprotection, involving the placentation process and the spiral artery remodelling. Nevertheless, a reduction in taurine activity may be associated with impaired placentation, which is correlated with the pathophysiology of preeclampsia. [43] Glycylglycine is strongly associated with homocysteine levels and participates in the metabolic pathway that antagonizes homocysteine: lower glycine levels promote higher homocysteine levels. Homocysteine is linked to cardiovascular risk and endothelial dysfunction.

Indeed, a reduction in arginine and homoarginine was observed among preeclampsia patients. [42] These are substrates for oxide nitric synthesis, a potent vasodilator with a major function in endothelial cells. ADMA is an inhibitor of the enzyme responsible for NO production; there is evidence that elevated ADMA levels are associated with endothelial dysfunction, and consequently, preeclampsia. [48,61] NO is an important regulator of trophoblast implantation, differentiation, motility, invasion and apoptosis. [42] Decreased levels of arginine and homoarginine in blood samples of preeclampsia

For peer review only - http://bmjopen.bmj.com/site/about/guidelines.xhtml

patients reinforces the involvement of NO molecule in the aetiology of the disorder. [42,50]

Carnitine is a non-essential amino acid strongly associated with lipid metabolism. Its function is to bind to fatty acid, form acyl-carnitine, and shuttle it to mitochondria, to integrate the mitochondrial oxidative metabolism. [52] The increment of carnitine might be associated to the oxidative stress observed in preeclampsia. [24,25,35,46,49,52]

Lipids

Maternal serum lipids are markedly elevated in healthy pregnancies, probably a hormone- induced increase. [62] Preeclamptic women may have even higher levels. [63] The lipidome – the group of lipid metabolites - contains key mediators of vascular tone (sphingosine), inflammation (prostaglandins), and insulin sensitivity (free fatty acids). [24] Lipids are involved in the aetiology of preeclampsia either indirectly, as a substrate, or directly, as a disease mediator. [63] Lipid peroxides may cause endothelial cell activation and reinforce the action of diabetes *mellitus* and essential hypertension as important risk factors to preeclampsia. [23]

In a healthy pregnancy, the pro-angiogenic biochemical scenario, sphingosine-1-phosphate and its receptors are up-regulated. Ceramide levels are also increased, reflecting the trophoblastic apoptosis process, observed because of normal syncytial fusion of villous trophoblasts. [63] On the other hand, in preeclampsia, low levels of sphingosine-1-phosphate (angiogenic) and high levels of ceramides (pro-apoptotic) were found in serum analyses of 3rd trimester preeclampsia cases. [26] The increase of sphingomyelin SM:18 might serve not only as atherogenic marker of preeclampsia development and progression, but also the marker of cardiovascular complications developed later in women who had previous preeclamptic pregnancies. Recent studies have demonstrated that serum elevation of sphingomyelin SM:18 positively correlates with parameters of insulin resistance and liver function in obese adults [64] and correlates with markers of NFkB activation and, thus, markers of intracellular inflammation. [64] The ceramides might be biomarkers for cardiovascular disease in

For peer review only - http://bmjopen.bmj.com/site/about/guidelines.xhtml

later life, which points attention to its role in hypertension in pregnancy. [65] As ceramide and their synthesizing enzymes (e.g. ceramide synthases) have been shown to be significantly up-regulated in the mouse uterus during early gestation, [44,66] the role of ceramides in the normal human pregnancy and its relationship with preeclampsia etiopathogenesis needs to be further explored.

Preeclampsia has also been correlated to the increment of reactive oxygen species. In this sense, there is a consequent increment of oxidized cholesterol, resulting of phosphocholine exposition to reactive oxygen species. Increased levels of acylcarnitine reflect this fatty acid oxidation impairment. [66]

Glycerophosphocholines resulted from cell lysis, possibly correlated with apoptosis described in preeclampsia. For the propanoate pathway, late-onset preeclampsia showed higher concentrations of L- valine, 2-hydroxybutyric acid, lactic acid, acetone and propan-2-ol. [52] Decreased glycerol level has been regarded as an important biomarker of late-onset preeclampsia and this may be due to its conversion to triglycerides. [57,63]

Vitamin D

The included studies on vitamin D achieved contradictory results. [29,34,36,41,44,51] The heterogeneity of results may be attributable to the measurement of only serum 25(OH)D3 vitamin D, which is considered simplistic. [29] Interestingly, all included studies have considered low risk women and have presented data irrespective of gestational age of preeclampsia diagnosis. The active metabolite - 1,25-dihydroxyvitamin D - is thought to increase vascular endothelial growth factor transcription, [67] which is correlated to placenta functioning and adaptation. Besides, vitamin D deficiency may be associated with an increment of inflammatory cytokine levels, such as TNF-alpha, and to a tendency towards T-cell activity. [68] Thus, its deficiency could potentially contribute to the pathophysiology of preeclampsia: abnormal placental implantation, angiogenesis, excessive inflammation, immune dysfunction. [69] Accuracy performance of vitamin D was increased when combined with angiogenic biomarkers (VEGF + sFLT-1/PlGF).[41] Additionally, all targeted studies

For peer review only - http://bmjopen.bmj.com/site/about/guidelines.xhtml

showed lower levels of vitamin D metabolites, both in serum and urine, while untargeted LC-MS evaluation observed upregulated levels. Then, based on our results, we cannot rule out vitamin D role in predicting preeclampsia in high-risk population or early- or late-onset of disease. It seems there is a place for investigating its role in composing a multifactorial model of prediction.

Environmental exposures

Knowledge of the actual contribution of endocrine disrupting chemicals (EDC), such as BPA, phthalates, and others to preeclampsia is relevant, given that behavioural attitude can change maternal exposure to these risk factors. It is hypothesized that BPA can cross the placenta and induce degeneration and necrosis of placental cells, disturbing angiogenesis, [47] and possibly acting through estrogenic-related receptor *gama.* Phthalates can affect placental gene expression determining decreased placental growth, [64] and may also cause a pro-inflammatory response and increase the oxidative stress. [70] Although their role in preeclampsia remains elusive, [27,31,47,60] we cannot rule out their role before pregnancy: BPA, phthalates and PFASs are widely present in many products of daily life, and usually have half-lives of many years.

Another environmental factor that was pointed in our review was caffeine. Caffeine metabolism occurs in the cytochrome P450 1A2 system, and the primary metabolite of dietary caffeine (80% of caffeine by-product) is the paraxanthine. The activity of this cytochrome can be demonstrated by the ratio of paraxanthine/caffeine. During pregnancy, the primary pathway for all caffeine clearance happens in maternal cytochrome P450 1A2 system, as neither placenta nor the foetus demonstrates this cytochrome activity. [71] The decreased risk of severe preeclampsia with increasing paraxanthine/caffeine ratio suggests that faster caffeine metabolism may be associated with a lower risk of preeclampsia. [45] This is not exactly in accordance with other studies, which indicated a direct influence of caffeine levels on the risk of preeclampsia and failed to perform analysis of caffeine metabolism. [72] On the other hand, caffeine and its metabolites are antagonists of the adenosine A1 receptors, implicated in proximal tubular sodium reabsorption. [73] Although caffeine consumption may be associated to elevated blood pressure in the short term (≤3

For peer review only - http://bmjopen.bmj.com/site/about/guidelines.xhtml

hours), it does not represent a cardiovascular risk factor in the long term (2 weeks) for non-pregnant adults. [74] Future studies should consider daily intake of caffeine, which was not explored in the included study, and maternal smoking as potential bias since cigarette use induces the P450 1A2 system. [75]

Methodological quality

Although majority of included studies were ranked as low risk of bias and low applicability concerns, there is still place for improving data reporting in metabolomics. [76] Moving data from bench to bedside implies the clear description of reference standards and index tests, and timing of index test analysis. As preeclampsia has a complex aetiology and is a multifactorial disease, improving 'Patient Selection' should be the focus in the next studies. We could have better accuracy if we separate, for example, data from low- and high-risk women, or single versus multiple gestation.

Strengths and limitations

This systematic review is worthwhile as a guidance of metabolites and their performance as predictors of preeclampsia. We have applied a systematic review strategy in eight different electronic databases. Then, we presented possible biomarkers of preeclampsia that could ground future research in this area.

Unfortunately, there are limitations. The inclusion of case-control studies could add risk of bias, confounding and potential measurement error. However, we understand this is an important strategy to Biomarkers Selection and Performance Evaluation steps in metabolomics [6] in perinatal research. There was great heterogeneity among included studies. *Per se*, metabolomics is an overly complex process, influenced by sample collection, storage and preparation, analytical platform applied, statistical tests performed, time of day collection, fasting or non-fasting state prior the samples. [6] Thus, results presented in the studies must consider all these variables. Exactly because of this complexity and authentic heterogeneity of metabolomics, the lack of standardization of preeclampsia concept (early and late-onset preeclampsia) and time of sample collection represented a limitation to our study. Thus, the results could not be correlated with this important variable. Additionally, since only two studies

For peer review only - http://bmjopen.bmj.com/site/about/guidelines.xhtml

evaluated preeclampsia prediction regarding fetal sex, our findings should be interpreted with caution.

CONCLUSION AND IMPLICATIONS FOR PRACTICE

We have presented a list of 122 different metabolites and their accuracy measures, when possible. They are mainly involved with ammonia recycling; amino acid and lipids metabolism; arachidonic acid metabolism; cell signalling; galactose, lactose, and nucleotide sugars metabolisms. Maternal blood seems to be the best specimen to predict early preeclampsia if sampled in the 1st trimester, and to predict late-onset preeclampsia in the 2nd trimester. Future metabolomics platforms should be as comprehensive as possible to evaluate the contributions of amino acids, peptides, fatty acid esters, and carbohydrates conjugates, as preeclampsia biomarkers.

Although individual metabolites have low predictive measures, they should be considered in multifactorial models of metabolites alone or in combination with other biomarkers. [25,33,38,41,43,46,50,52] Metabolite signature may contribute to further insights into the pathogenesis of preeclampsia and to design screening tests, which may contribute to early recognizing high risk women to hypertensive disorders In future, it is important to validate citrate and carnitines as common markers between early-onset and late-onset preeclampsia, considering the feasibility of universal screening. [25,49,50] Additionally, since preeclampsia and SGA are related to abnormal deep placentation, [77] it seems plausible to study both conditions simultaneously, at least considering the metabolites found in common (sphinganine 1-phosphate, sphingosine 1-phosphate, tyrosine, vitamin D, N1, N12-diacetylspermine). [56]

References

1 Mol BWJ, Roberts CT, Thangaratinam S, *et al.* Pre-eclampsia. *Lancet* 2016;**387**:999–1011. doi:10.1016/S0140-6736(15)00070-7

2 L. C. *Chesley's Hypertensive Disorders in Pregnancy*. 4th ed. Elsevier 2015.

3 Roberts JM, Rich-Edwards JW, McElrath TF, *et al.* Subtypes of Preeclampsia: Recognition and Determining Clinical Usefulness. *Hypertension* 2021;:1430–41.

For peer review only - http://bmjopen.bmj.com/site/about/guidelines.xhtml

doi:10.1161/HYPERTENSIONAHA.120.14781

4 Laganà AS, Favilli A, Triolo O, *et al.* Early serum markers of pre-eclampsia: are we stepping forward? *J Matern Fetal Neonatal Med* 2016;**29**:3019–23.

5 Nobakht M. Gh BF. Application of metabolomics to preeclampsia diagnosis. *Syst Biol Reprod Med* 2018;**64**:324–39. doi:10.1080/19396368.2018.1482968

6 Xia J, Broadhurst DI, Wilson M, *et al.* Translational biomarker discovery in clinical metabolomics: An introductory tutorial. *Metabolomics* 2013;**9**:280–99. doi:10.1007/s11306-012-0482-9

7 Guasch-Ferré M, Hruby A, Toledo E, *et al.* Metabolomics in prediabetes and diabetes: A systematic review and meta-analysis. *Diabetes Care* 2016;**39**:833–46. doi:10.2337/dc15-2251

8 Hoang G, Udupa S, Le A. *Application of metabolomics technologies toward cancer prognosis and therapy*. 1st ed. Elsevier Inc. 2019. doi:10.1016/bs.ircmb.2019.07.003

9 Mayrink J, Leite DFB, Costa ML, *et al.* Metabolomics for prediction of hypertension in pregnancy: A systematic review and meta-analysis protocol. *BMJ Open* 2020;**10**:1–5. doi:10.1136/bmjopen-2020-040652

10 Moher D, Liberati A, Tetzlaff J, *et al.* Preferred Reporting Items for Systematic Reviews and Meta-Analyses: the PRISMA statement. *Ann Intern Med* 2009;**151**:264–9. doi:10.1371/journal.pmed1000097

11 Tranquilli AL, Dekker G, Magee L, *et al.* The classification, diagnosis and management of the hypertensive disorders of pregnancy: A revised statement from the ISSHP. *Pregnancy Hypertens* 2014;**4**:97–104. doi:10.1016/j.preghy.2014.02.001

12 Brown MA, Hague WM, Higgins J, *et al.* The detection, investigation and management of hypertension in pregnancy: Executive summary. *Aust New Zeal J Obstet Gynaecol* 2000;**40**:133–8. doi:10.1111/j.1479-828X.2000.tb01136.x

13 ACOG Committee on Practice Bulletins--Obstetrics. ACOG practice bulletin. Diagnosis and management of preeclampsia and eclampsia. Number 33, January 2002. *Obstet Gynecol* 2002;**99**:159–67. doi:10.1097/00006250-200201000-00028

14 ACOG. Hypertension in Pregnancy. *Obstet Gynecol* 2013;**122**:1122–31. doi:10.1097/01.AOG.0000437382.03963.88

15 Report of the National High Blood Pressure Education Program Working Group on High Blood Pressure in Pregnancy. *Am J Obstet Gynecol* 2000;**183**:S1–22. doi:10.1067/mob.2000.107928

16 Geraldo J, Ramos L, Sass N, *et al.* Preeclampsia: Definitions of Hypertensive States during Pregnancy Pathophysiological Foundations. *Rev Bras Ginecol Obs* 2017;**39**:496–512.https://pubmed.ncbi.nlm.nih.gov/28793357/

17 Whiting PF, Rutjes AWS, Westwood ME, *et al.* QUADAS-2: A Revised Tool for the Quality Assessment of Diagnostic Accuracy Studies. *Ann Intern Med* 2011;**155**:529–36. doi:10.7326/0003-4819-155-8-201110180-00009

18 Wishart DS, Feunang YD, Marcu A, *et al.* HMDB 4.0: The human metabolome database

For peer review only

For peer review only - http://bmjopen.bmj.com/site/about/guidelines.xhtml

for 2018. *Nucleic Acids Res* 2018;**46**:D608–17. doi:10.1093/nar/gkx1089

19 Kanehisa M, Goto S. Kyoto Encyclopedia of Genes and Genomes. *Nucleic Acids Res* 2000;**1**:27–30.https://www.genome.jp/kegg/ (accessed 24 Feb 2022).

20 Fahy E, Sud M, Cotter D, *et al.* LIPID MAPS online tools for lipid research. *Nucleic Acids Res* 2007;**35**:606–12. doi:10.1093/nar/gkm324

21 Rutter CM, Gatsonis CA. A hierarchical regression approach to meta-analysis of diagnostic test accuracy evaluations. *Stat Med* 2001;**20**:2865–84. doi:10.1002/sim.942

22 Deeks JJ, Macaskill P, Irwig L. The performance of tests of publication bias and other sample size effects in systematic reviews of diagnostic test accuracy was assessed. *J Clin Epidemiol* 2005;**58**:882–93. doi:10.1016/j.jclinepi.2005.01.016

23 Chappell L. A longitudinal study of biochemical variables in women at risk of preeclampsia. *Am J Obstet Gynecol* 2002;**187**:127–36. doi:10.1067/mob.2002.122969

24 Kenny LC, Broadhurst DI, Dunn W, *et al.* Robust early pregnancy prediction of later preeclampsia using metabolomic biomarkers. *Hypertension* 2010;**56**:741–9. doi:10.1161/HYPERTENSIONAHA.110.157297

25 Bahado-Singh R, Poon LC, Yilmaz A, *et al.* Integrated Proteomic and Metabolomic prediction of Term Preeclampsia. *Sci Rep* 2017;**7**:1–10. doi:10.1038/s41598-017-15882-9

26 Dobierzewska A, Soman S, Illanes SE, *et al.* Plasma cross-gestational sphingolipidomic analyses reveal potential first trimester biomarkers of preeclampsia. *PLoS One* 2017;**12**:1–16. doi:10.1371/journal.pone.0175118

27 Ye Y, Zhou Q, Feng L, *et al.* Maternal serum bisphenol A levels and risk of pre-eclampsia: A nested case-control study. *Eur J Public Health* 2017;**27**:1102–7. doi:10.1093/eurpub/ckx148

28 Gong S, Sovio U, Aye IL, *et al.* Placental polyamine metabolism differs by fetal sex, fetal growth restriction, and preeclampsia. *JCI insight* 2018;**3**:1–15. doi:10.1172/jci.insight.120723

29 Tamblyn JA, Jenkinson C, Larner DP, *et al.* Serum and urine vitamin D metabolite analysis in early preeclampsia. *Endocr Connect* 2018;**7**:199–210. doi:10.1530/EC-17-0308

30 Lee SM, Kang Y, Lee EM, *et al.* Metabolomic biomarkers in midtrimester maternal plasma can accurately predict the development of preeclampsia. *Sci Rep* 2020;**10**:16142. doi:10.1038/s41598-020-72852-4

31 Rylander L, Lindh CH, Hansson SR, *et al.* Per- and Polyfluoroalkyl Substances in Early Pregnancy and Risk for Preeclampsia: A Case-Control Study in Southern Sweden. *Toxics* 2020;**8**. doi:10.3390/toxics8020043

32 Harville EW, Li Y-YY-Y, Pan K, *et al.* Untargeted analysis of first trimester serum to reveal biomarkers of pregnancy complications: a case-control discovery phase study. *Sci Rep* 2021;**11**:3468. doi:10.1038/s41598-021-82804-1

33 Kenny LC, Thomas G, Poston L, *et al.* Prediction of preeclampsia risk in first time

For peer review only

For peer review only - http://bmjopen.bmj.com/site/about/guidelines.xhtml

pregnant women: Metabolite biomarkers for a clinical test. *PLoS One* 2020;**15**:1–19. doi:10.1371/journal.pone.0244369

34 Powe CE, Seely EW, Rana S, *et al.* First trimester vitamin D, vitamin D binding protein, and subsequent preeclampsia. *Hypertension* 2010;**56**:758–63. doi:10.1161/HYPERTENSIONAHA.110.158238

35 Odibo AO, Goetzinger KR, Odibo L, *et al.* First-trimester prediction of preeclampsia using metabolomic biomarkers: a discovery phase study. *Prenat Diagn* 2011;**31**:990–4.

36 Kiely ME, Zhang JY, Kinsella M, *et al.* Vitamin D status is associated with uteroplacental dysfunction indicated by pre-eclampsia and small-for-gestational-age birth in a large prospective pregnancy cohort in Ireland with low Vitamin D status. *Am J Clin Nutr* 2016;**104**:354–61. doi:10.3945/ajcn.116.130419

37 Huo X, Huang R, Gan Y, *et al.* Perfluoroalkyl substances in early pregnancy and risk of hypertensivedisorders of pregnancy: A prospective cohort study. *Environ Int* 2020;**138**. doi:10.1016/j.envint.2020.105656

38 Sovio U, McBride N, Wood AM, *et al.* 4-Hydroxyglutamate is a novel predictor of pre-eclampsia. *Int J Epidemiol* 2020;**49**:301–11. doi:10.1093/ije/dyz098

39 Kenny LC, Broadhurst DI, Hong W, *et al.* NMR Profiling in Early Pregnancy Reveals a Metabolomic Signature the Predicts the Subsequent Development Preeclampsia. *Reprod Sci* 2013;**20**:2641A. doi:10.1177/1933719113482088

40 Huang Q, Hao S, You J, *et al.* Early-pregnancy prediction of risk for pre-eclampsia using maternal blood leptin/ceramide ratio: Discovery and confirmation. *BMJ Open* 2021;**11**. doi:10.1136/bmjopen-2021-050963

41 Woodham PC, Brittain JE, Baker AM, *et al.* Midgestation maternal serum 25-hydroxyvitamin D level and soluble fms-like tyrosine kinase 1/placental growth factor ratio as predictors of severe preeclampsia. *Hypertension* 2011;**58**:1120–5. doi:10.1161/HYPERTENSIONAHA.111.179069

42 Khalil AA, Tsikas D, Akolekar R, *et al.* Asymmetric dimethylarginine, arginine and homoarginine at 11-13 weeks gestation and preeclampsia: A case-control study. *J Hum Hypertens* 2013;**27**:38–43. doi:10.1038/jhh.2011.109

43 Kuc S, Koster MPH, Pennings JLA, *et al.* Metabolomics profiling for identification of novel potential markers in early prediction of preeclampsia. *PLoS One* 2014;**9**. doi:10.1371/journal.pone.0098540

44 Wetta LA, Biggio JR, Cliver S, *et al.* Is midtrimester vitamin D status associated with spontaneous preterm birth and preeclampsia? *Am J Perinatol* 2014;**31**:541–6. doi:10.1055/s-0033-1356483

45 Eichelberger KY, Baker AM, Woodham PC, *et al.* Second-trimester maternal serum paraxanthine, CYP1A2 activity, and the risk of severe preeclampsia. *Obstet Gynecol* 2015;**126**:725–30. doi:10.1097/AOG.0000000000001041

46 Koster MPH, Vreeken RJ, Harms AC, *et al.* First-Trimester serum acylcarnitine levels to predict preeclampsia: A metabolomics approach. *Dis Markers* 2015;**2015**. doi:10.1155/2015/857108

For peer review only

For peer review only - http://bmjopen.bmj.com/site/about/guidelines.xhtml

47 Cantonwine DE, Meeker JD, Ferguson KK, *et al.* Urinary concentrations of bisphenol A and phthalate metabolites measured during pregnancy and risk of preeclampsia. *Environ Health Perspect* 2016;**124**:1651–5. doi:10.1289/EHP188

48 Rijvers CAH, Marzano S, Winkens B, *et al.* Early-pregnancy asymmetric dimethylarginine (ADMA) levels in women prone to develop recurrent hypertension. *Pregnancy Hypertens* 2013;**3**:118–23. doi:10.1016/j.preghy.2013.01.001

49 Diaz SO, Barros AS, Goodfellow BJ, *et al.* Second trimester maternal urine for the diagnosis of trisomy 21 and prediction of poor pregnancy outcomes. *J Proteome Res* 2013;**12**:2946–57. doi:10.1021/pr4002355

50 Bahado-Singh RO, Syngelaki A, Akolekar R, *et al.* Validation of metabolomic models for prediction of early-onset preeclampsia. *Am J Obstet Gynecol* 2015;**213**:530.e1-530.e10. doi:10.1016/j.ajog.2015.06.044

51 Ates S, Sevket O, Ozcan P, *et al.* Vitamin D status in the first-trimester: Effects of vitamin D deficiency on pregnancy outcomes. *Afr Health Sci* 2016;**16**:36–43. doi:10.4314/ahs.v16i1.5

52 Bahado-Singh RO, Syngelaki A, Mandal R, *et al.* Metabolomic determination of pathogenesis of late-onset preeclampsia. *J Matern Neonatal Med* 2017;**30**:658–64. doi:10.1080/14767058.2016.1185411

53 Shanmugalingam R, Wang X, Motum P, *et al.* The 15-Epilipoxin-A4 Pathway with Prophylactic Aspirin in PreventingPreeclampsia: A Longitudinal Cohort Study. *J Clin Endocrinol Metab* 2020;**105**. doi:10.1210/clinem/dgaa642

54 Bilodeau JF, Qin Wei S, Larose J, *et al.* Plasma F2-isoprostane class VI isomers at 12-18 weeks of pregnancy are associated with later occurrence of preeclampsia. *Free Radic Biol Med* 2015;**85**:282–7. doi:10.1016/j.freeradbiomed.2015.05.012

55 Gong S, Sovio U, Aye ILMH, *et al.* Placental polyamine metabolism differs by fetal sex, fetal growth restriction, and preeclampsia. *JCI Insight* 2018;**3**:1–15. doi:10.1172/jci.insight.120723

56 Leite DFB, Morillon A-C, Junior EF de M, *et al.* Examining the predictive accuracy of metabolomics for small for gestational age babies: a systematic review. *BMJ Open* 2019;**9**:e031238. doi:10.1136/ bmjopen-2019-031238

57 Bahado-Singh RO, Akolekar R, Mandal R, *et al.* First-trimester metabolomic detection of late-onset preeclampsia. *Am J Obstet Gynecol* 2013;**208**:58.e1-58.e7. doi:10.1016/j.ajog.2012.11.003

58 Nelson DB, Ziadie MS, McIntire DD, *et al.* Placental pathology suggesting that preeclampsia is more than one disease. *Am J Obstet Gynecol* 2014;**210**:66.e1-66.e7. doi:10.1016/j.ajog.2013.09.010

59 Khalaf D, Krüger M, Wehland M, *et al.* The effects of oral l-arginine and l-citrulline supplementation on blood pressure. *Nutrients* 2019;**11**:1–14. doi:10.3390/nu11071679

60 Huo X, Huang R, Gan Y, *et al.* Perfluoroalkyl substances in early pregnancy and risk of hypertensive disorders of pregnancy: A prospective cohort study. *Environ Int* 2020;**138**:1–7. doi:10.1016/j.envint.2020.105656

For peer review only - http://bmjopen.bmj.com/site/about/guidelines.xhtml

61 Levine RJ, Lam C, Qian C, *et al.* Soluble Endoglin and Other Circulating Antiangiogenic Factors in Preeclampsia. *N Engl J Med* 2006;**355**:992–1005.

62 Wang C, Kong L, Yang Y, *et al.* Recommended reference values for serum lipids during early and middle pregnancy: A retrospective study from China 11 Medical and Health Sciences 1114 Paediatrics and Reproductive Medicine. *Lipids Health Dis* 2018;**17**:1–16. doi:10.1186/s12944-018-0885-3

63 Wojcik-Baszko D, Charkiewicz K, Laudanski P. Role of dyslipidemia in preeclampsia—A review of lipidomic analysis of blood, placenta, syncytiotrophoblast microvesicles and umbilical cord artery from women with preeclampsia. *Prostaglandins Other Lipid Mediat* 2018;**139**:19–23. doi:10.1016/j.prostaglandins.2018.09.006

64 Aris A, Benali S, Ouellet A, *et al.* Potential Biomarkers of Preeclampsia: Inverse Correlation between Hydrogen Peroxide and Nitric Oxide Early in Maternal Circulation and at Term in Placenta of Women with Preeclampsia. *Placenta* 2009;**30**:342–7. doi:10.1016/j.placenta.2009.01.003

65 de Carvalho LP, Tan SH, Ow GS, *et al.* Plasma Ceramides as Prognostic Biomarkers and Their Arterial and Myocardial Tissue Correlates in Acute Myocardial Infarction. *JACC Basic to Transl Sci* 2018;**3**:163–75. doi:10.1016/j.jacbts.2017.12.005

66 Anand S, Young SA, Esplin MS, *et al.* Detection and confirmation of serum lipid biomarkers for preeclampsia using direct infusion mass spectrometry. *J Lipid Res* 2016;**57**:687–96. doi:10.1194/jlr.P064451

67 Cardus A, Panizo S, Encinas M, *et al.* 1,25-Dihydroxyvitamin D3 regulates VEGF production through a vitamin D response element in the VEGF promoter. *Atherosclerosis* 2009;**204**:85–9. doi:10.1016/j.atherosclerosis.2008.08.020

68 Baker AM, Haeri S, Camargo CA, *et al.* A nested case-control study of midgestation vitamin D deficiency and risk of severe preeclampsia. *J Clin Endocrinol Metab* 2010;**95**:5105–9. doi:10.1210/jc.2010-0996

69 Westwood M, Al-Saghir K, Finn-Sell S, *et al.* Vitamin D attenuates sphingosine-1-phosphate (S1P)-mediated inhibition of extravillous trophoblast migration. *Placenta* 2017;**60**:1–8. doi:10.1016/j.placenta.2017.09.009

70 Werner EF, Braun JM, Yolton K, *et al.* The association between maternal urinary phthalate concentrations and blood pressure in pregnancy: The HOME Study. *Environ Heal A Glob Access Sci Source* 2015;**14**:1–9. doi:10.1186/s12940-015-0062-3

71 Grosso LM, Bracken MB. Caffeine metabolism, genetics, and perinatal outcomes: A review of exposure assessment considerations during pregnancy. *Ann Epidemiol* 2005;**15**:460–6. doi:10.1016/j.annepidem.2004.12.011

72 Bakker R, Steegers EAP, Raat H, *et al.* Maternal caffeine intake, blood pressure, and the risk of hypertensive complications during pregnancy. the generation R study. *Am J Hypertens* 2011;**24**:421–8. doi:10.1038/ajh.2010.242

73 Guessous I, Eap CB, Bochud M. Blood Pressure in Relation to Coffee and Caffeine Consumption. *Curr Hypertens Rep* 2014;**16**:1–9. doi:10.1007/s11906-014-0468-2

74 Mesas AE, Leon-Muñoz LM, Rodriguez-Artalejo F, *et al.* The effect of coffee on blood pressure and cardiovascular disease in hypertensive individuals: A systematic review

For peer review only - http://bmjopen.bmj.com/site/about/guidelines.xhtml

and meta-analysis. *Am J Clin Nutr* 2011;**94**:1113–26. doi:10.3945/ajcn.111.016667

75 Dobrinas M, Cornuz J, Eap CB. Pharmacogenetics of CYP1A2 activity and inducibility in smokers and exsmokers. *Pharmacogenet Genomics* 2013;**23**:286–92. doi:10.1097/FPC.0b013e3283602e75

76 Goodacre R, Broadhurst D, Smilde AK, *et al.* Proposed minimum reporting standards for data analysis in metabolomics. *Metabolomics* 2007;**3**:231–41. doi:10.1007/s11306-007-0081-3

77 Brosens I, Pijnenborg R, Vercruysse L, *et al.* The 'great obstetrical syndromes' are associated with disorders of deep placentation. *Am J Obs Gynecol* 2011;**204**:193–201. doi:10.1016/j.ajog.2010.08.009.THE

For peer review only

For peer review only - http://bmjopen.bmj.com/site/about/guidelines.xhtml

Legends

Figures

Figure 1. Flow chart of study identification, screening, eligibility and inclusion.

Figure 2. Characterization of study selection regarding biological samples, sampling moment, and metabolomic analysis techniques applied

Figure 3. Assessment of risk of bias (left) and applicability concerns (right) of included studies.

Figure 4. Preeclampsia related metabolites, chemical classes, and biological processes.

Tables

Table 1. Characteristics of included studies.

Table 2. Metabolites and their accuracy measures for predicting preeclampsia according to gestational age of required delivery.

Table 3 – Accuracy measures of metabolites and other pregnancy-related biomarkers.

Supplementary Material

Supplementary material 1 Preferred Reported Items for Systematic Reviews and Meta-Analysis (PRISMA) statement: a PRISMA checklist

Supplementary material 2 – Full search strategy for each database.

Supplementary material 3- Excluded studies and reasons for exclusion.

Supplementary material 4 - Predictive metabolites of hypertensive disorders of pregnancy summarized according to their superclass, chemical class, subclass, and metabolic pathways.

Supplementary material 5 – Individual QUADAS-2 data for all included studies.

ACKNOWLEDGEMENTS

We are grateful to all members of the PRETERM-SAMBA study research group.

For peer review only - http://bmjopen.bmj.com/site/about/guidelines.xhtml

AUTHORS CONTRIBUTIONS

JM is the guarantor of this review. JM and DFBL elaborated the protocol, searched the literature, selected studies, extracted data, assessed risk of bias, and drafted the initial manuscript. MLC has helped in study inclusion, interpreting data, examining clinical applications of findings, and revising the manuscript. GN has helped in figures and tables elaboration and with the final version of the manuscript. JGC have supervised and approved all steps. All authors have read and agree with this submission.

FUNDING

DFBL (process number 88881.134512/2016-01) has awarded scholarship from Brazilian Federal Agency for Support and Evaluation of Graduate Education (CAPES). This research is part of the PRETERM-SAMBA study, which has granted sponsor from Brazilian National Research Council (CNPq) (Award 401636/2013-5) and from the Bill and Melinda Gates Foundation (grant OPP1107597). This specific analysis received no specific grant from any sectors. Our sponsors have not intervened in any step of this study design, development, or submission.

COMPETING INTERESTS

None to declare.

PATIENT CONSENT FOR PUBLICATION

Not required.

PROVENANCE AND PEER REVIEW

Not commissioned; externally peer reviewed.

DATA AVAILABILITY STATEMENT

No data are available.

For peer review only

1 **Table 1. Characteristics of included studies.**

2

Authors, year	Country of data collection	Study design	Classification of hypertensive syndrome	Comments
Chappell LC et al, 2002 [1]	United Kingdom	Case-control	ISSHP, 1988	17 women with preeclampsia; 21 without preeclampsia. Chronic hypertensive women included.
Powe CE et al, 2010 [2]	United States of America	Nested case-control	ICD codes	39 preeclampsia, 131 controls. Gestational diabetes excluded.
Kenny LC et al, 2010 [3]	New Zealand; Australia	Nested case-control	ASSHP, 2000	Discovery phase (New Zealand): 60 women with preeclampsia, 60 matched controls (age, ethnicity, body mass index) with uneventful pregnancies. Validation phase (Australia): 39 women with preeclampsia, 40 healthy matched pregnancies (SCOPE study).
Odibo AO et al, 2011 [4]	United States of America	Nested case-control	ACOG, 2002	41 women with preeclampsia, 41 normotensive controls. Pregestational diabetes included.
Woodham PC et al, 2011 [5]	United States of America	Nested case-control	ACOG, 2002	41 women with severe preeclampsia, 123 normotensive uncomplicated pregnancies at term. Chronic illness and multiple pregnancies excluded.
Rijvers CAH et al, 2013 [6]	Netherlands	Retrospective cohort	NHBP, 2000	06 women with preeclampsia, 11 with gestational hypertension, 18 normotensive women
Khalil AA et al, 2013 [7]	United Kingdom	Case-control	ISSHP, 2001	25 women with early preeclampsia, 50 cases of late preeclampsia, 300 normotensive controls (matching 1 case: 4 controls). Only singleton pregnancies; chronic hypertensive women included.
Diaz SO et al, 2013 [8]	Portugal	Prospective cohort	ACOG, 2002	09 women with preterm preeclampsia (31-37weeks); 84 women with healthy term pregnancies

For peer review only - http://bmjopen.bmj.com/site/about/guidelines.xhtml

Kenny LC et al, 2013 [9]	New Zealand; Australia	Nested case-control	ASSHP, 2000	49 women with preeclampsia and 49 normotensive nulliparous women. (SCOPE study).
Kuc S et al, 2014 [10]	Netherlands	Nested case-control	ISSHP, 2001	68 early-onset preeclampsia, 99 late-onset preeclampsia, 500 controls
Wetta LA et al, 2014 [11]	United States of America	Nested case-control	ACOG, 2002	89 women with preterm preeclampsia; 177 normotensive and term pregnancies
Bahado-Singh RO et al, 2015 [12]	United Kingdom	Prospective cohort	ISSHP, 2001	50 cases of early preeclampsia, 108 normotensive controls. Singleton pregnancies delivered at term; birthweight adequate for gestational age. HELLP syndrome cases excluded.
Eichelberger KY et al, 2015 [13]	United States of America	Nested case-control	ACOG, 2002	25 women with severe preeclampsia; 87 normotensive, healthy pregnancies delivered at term. Chronic hypertensive women and multiple pregnancies excluded.
Koster MPH et al, 2015 [14]	Netherlands	Nested case-control	ISSHP, 2001	68 early-onset preeclampsia, 99 late-onset preeclampsia, 500 controls
Bilodeau JF et al, 2015 [15]	Canada	Prospective cohort	CHS, 1997	33 preeclampsia, 60 controls (MIROS study)
Ates S et al, 2016 [16]	Turkey	Prospective cohort	ACOG, 2002	04 women with preeclampsia; 11 women with gestational hypertension; 214 normotensive women
Cantonwine DE et al, 2016 [17]	United States of America	Nested case-control	ACOG, 2002	50 women with preeclampsia, 431 without preeclampsia. Chronic hypertensive women included.
Kiely ME et al, 2016 [18]	Ireland	Nested case-control	ASSHP, 2000	68 preeclampsia, 1528 controls. SGA infants excluded from controls. (SCOPE Study)
Bahado-Singh RO et al,	United Kingdom	Prospective cohort	ISSHP, 2001	59 late-onset preeclampsia cases, 115 normotensive controls. Singleton

For peer review only - http://bmjopen.bmj.com/site/about/guidelines.xhtml

2017 [19]				pregnancies delivered at term; birthweight adequate for gestational age. HELLP syndrome cases excluded.
Bahado-Singh RO et al, 2017b [20]	United Kingdom	Nested case-control	ISSHP, 2001	35 term preeclampsia cases (≥37w), 65 normotensive controls. Singleton pregnancies, no major malformations; controls with birthweight adequate for gestational age.
Dobierzewska A et al, 2017 [21]	Chile	Case-control	ACOG, 2002	07 women with preeclampsia; 07 normotensive women
Ye Y et al, 2017 [22]	China	Nested case-control	ACOG, 2013	74 women with preeclampsia, 99 normotensive term pregnancies as controls. Only singleton pregnancies; chronic illness excluded in control group.
Gong S et al, 2018 [23]	United Kingdom	Case-control	ACOG, 2002	134 term preeclampsia, 259 normotensive controls. Nulliparous women, singleton pregnancies (POP Study).
Tamblyn JA et al, 2018 [24]	Ireland	Case-control	ASSHP, 2000	25 women with preeclampsia, 25 normotensive women (SCOPE Study).
Sovio U et al, 2020 [25]	United Kingdom	Case-cohort	ISSHP, 2001; ACOG, 2013	Training and test with POP Study: 194 women with preeclampsia, 323 controls. Validation within BiB Study: 95 women with preeclampsia, 953 normotensive controls.
Huo X et al, 2020 [26]	Shanghai, China	Case-Cohort	ACOG, 2013	64 gestational hypertension, 71 preeclampsia. Singleton gestation only; chronic hypertensive women excluded.
Rylander L et al, 2020 [27]	Sweden	Case-control	ICD codes	296 preeclampsia, 580 controls. Excluded pregnancies with SGA infants.
Lee SM et al, 2020 [28]	South Korea	Case-control	ACOG, 2013	33 preeclampsia, 66 controls. Singleton pregnancies, congenital

For peer review only - http://bmjopen.bmj.com/site/about/guidelines.xhtml

				malformations excluded.
Kenny L et al, 2020 [29]	Ireland and England	Nested case-control	ISSHP, 2018	97 preeclampsia (23 preterm preeclampsia and 74 term preeclampsia); 335 controls. Nulliparous women, singleton pregnancies. Chronic illness and fetal malformations excluded (SCOPE study)
Shanmugalingam R et al, 2020 [30]	Australia	Prospective cohort	ISSHP, 2018	21 preeclampsia, 103 controls. High risk women.
Harville EW et al, 2021 [31]	United States of America	Case-control	Not mentioned	18 preeclampsia, 109 controls. Only singleton pregnancies.
Huang Q et al, 2021 [32]	United States of America	Case-control	ACOG, 2013	20 preeclampsia, 20 controls (validation phase).

ACOG: American College of Obstetricians and Gynecologists; CHS, Canadian Hypertensive Society; ASSHP: Australasian Society of the Study of Hypertension in Pregnancy; ICD, International Classification of Diseases; BiB Study: Born in Bradford Study; ISSHP: International Society for the Study of Hypertension in Pregnancy; POP Study: Pregnancy Outcome Prediction; SCOPE Study: Screening for Pregnancy Endpoints; MIROS study: Maternal and Infant Research on Oxidative Stress; PTB: preterm birth; SGA, small for gestational age.

For peer review only - http://bmjopen.bmj.com/site/about/guidelines.xhtml

Table 2. Metabolites and their accuracy measures for predicting preeclampsia according to gestational age of required delivery.

**Preeclampsia at any gestational age**					
**Authors, year**	**Metabolomics methods**	**Maternal biological specimen; gestational age at sampling**	**Increased metabolites**	**Decreased metabolites**	**S/ Sp/ AUC (95% CI)**
Chappell LC et al, 2002 [1]	GC-MS	Blood; 20w	Isoprostane 8-epi-prostaglandin F2α		-/ -/ 0.62 (0.44-0.81)
		Blood; 24w	Isoprostane 8-epi-prostaglandin F2α		-/ -/ 0.55 (0.35-0.75)
Powe CE et al, 2010 [2]	Targeted LC-MS/MS	Blood (serum); 11w (±3w)		25(OH)vitamin D [a]	-/ -/ -
Odibo AO et al, 2011 [4]	Targeted LC-MS/MS	Blood (serum); 11-14w	Hydroxyhexanoylcarnitine, phenylalanine, glutamate, alanine		-/ -/ 0.82 (0.80–0.85)
Woodham PC et al, 2011 [5]	Targeted LC-MS/MS	Blood (serum); 15-20w		25(OH)vitamin D	-/ -/ 0.745
Diaz SO et al, 2013 [8]	Untargeted ^{1}H-NMR	Urine; 14-26w	*N*-methyl-2-pyridone-5-carboxamide, 3-methylhistidine, 4-deoxyerythronic acid, *cis*-aconitate, citrate, Glutamine, hippurate, indoxyl sulphate, *N*-methylnicotinamide, sucrose, U35	2-ketoglutarate, 4-OH-hippurate, acetate, carnitine, creatinine, formate, fumarate, galactose, isoleucine, lactose, phenylacetylglutamine, *p*-cresol sulphate, *scyllo*-ino, succinate, trigonelline, tyrosine, U14, U4, U10	-/ -/ -
Kenny LC et al, 2013 [9]	Untargeted H-NMR	Blood (serum); 15w		Acetamide, Glutamine, Trimethylamine, creatinine, dimethyl sulfone, valine,	-/-/-

For peer review only

For peer review only - http://bmjopen.bmj.com/site/about/guidelines.xhtml

				isoleucine, ornithine, glycine, methionine, betaine, acetate, proline	
Rijvers CAH et al, 2013 [6]	Targeted UPLC-MS/MS	Blood (plasma); 12w	L-arginine/ Asymmetric dimethylarginine ratio		-/ -/ -
		Blood (plasma); 16-20w		L-arginine	-/ -/ -
Wetta LA et al, 2014 [11]	Targeted LC-MS/MS	Blood (serum); 15-21w		25(OH)vitamin D [a]	-/ -/ -
Bilodeau JF et al, 2015 [15]	Targeted HPLC-MS/MS	Blood (plasma); 12-18w	(+/-) 5-iPF2α-VI, (+/-) 5-iPF2α-VI-(d11)		-/ -/ 0.67
	GC-FID	Blood (plasma); 12-18w		Alfa-linolenic acid, stearidonic acid, eicosatrienoic acid, eicosatetraenoic acid, eicosapentaenoic acid, docosapentaenoic acid, docosahexaenoic acid	-/ -/ -
Ates S et al, 2016 [16]	Targeted LC-MS/MS	Blood (serum); 11-14w		Vitamin D (<10mg/dL)	0.50/0.54
Cantonwine DE et al, 2016 [17]	Targeted HPLC-MS	Urine; 4-16w	Total bisphenol A, Σ di(2-ethylhexyl) phthalate, mono-ethyl phthalate	%mono(2-ethylhexyl) phthalate	-/ -/ -
		Urine; 22-29w	mono(2-ethylhexyl) phthalate, Σ di(2-ethylhexyl) phthalate, mono(3-carboxypropyl) phthalate	monoisobutyl phthalate	-/ -/ -
		Urine; 33-38w	mono(2-ethylhexyl) phthalate, Σ di(2-ethylhexyl) phthalate, mono(3-		-/ -/ -

For peer review only

			carboxypropyl) phthalate		
Kiely ME et al, 2016 [18]	Targeted LC-MS/MS	Blood (serum); 14-16w		25(OH)D3, 3-epi-25(OH)D3, and 25(OH)D2 [a]	-/ -/ -
Dobierzewska A et al, 2017 [21]	Targeted HPLC-MS	Blood (plasma); 11-14w		Sphingomyelin 16:0, sphingomyelin 18:0, ceramide 14:0	-/ -/ -
		Blood (plasma); 32-36w		Ceramide 14:0, ceramide 24:0	-/ -/ -
Ye Y et al, 2017 [22]	Targeted LC-MS	Blood (serum); 16-20w	Free bisphenol A		-/ -/ 0.73 (0.65-0.81)
Tamblyn JA et al, 2018 [24]	Targeted LC-MS/MS	Urine; 14-16w		25(OH) vitamin D3; 24,25(OH)2 vitamin D3[a]	-/ -/ -
Huo X et al, 2020 [26]	HPLC/MS-MS	Blood (serum); 13-17w	Perfluorooctanate; perfluorooctane sulfonate; perfluorodecanoic acid; perfluoroundecanoic acid; perfluorononanoic acid; perfluorohexanesulfonate; perfluoroheptanoic acid; perfluorobutane sulfonate; perfluorododecanoic acid; perfluorooctane sulfonamide[a]		-/ -/ -
Rylander L et al, 2020 [27]	Targeted LC-MS/MS	Blood (serum); 12-14w	Perfluorohexane sulfonate (0.53–0.78 ng/mL)		0.38/ 0.67/ -
Lee SM et al, 2020 [28]	GC-TOF MS	Blood (plasma); 16-24w		Propane-1,3-diol	0.75/ 0.83/ 0.868 (0.844-0.891)
	LC-Orbitrap	Blood (plasma);	LysoPE C20:0; SM C30:1	LysoPC C19:0; SM C28:1	

For peer review only

	MS	16-24w			
Kenny L et al, 2020 [29]	Targeted LC-MS/MS	Blood (plasma); 14-16w	Dilinoleoyl-glycerol (DLG)		0.39/ 0.90/0.70 (0.59-0.82)
Shanmugalingam R et al, 2020 [30]	Targeted LC-MS/MS	Blood (plasma); 12w		15-epilipoxin-A4	
Harville EW et al, 2021 [31]	Untargeted UPLC-HR-MS	Blood (serum); 6-14w	Cerasinone; bolasterone, 6.30_477.7721m/z, 5.86_313.1070m/z[b]		-/ -/ 0.95
	NMR	Blood (serum); 6-14w		Asparagine, N,N-Dimethylglycine, Trimethylamine	-/ -/ -
Huang Q et al, 2021 [32]	LC-MS	Blood (serum); 5-15w		Cer(d18:1/25:0)	-/-/0.868
		Blood (serum); 16-29w		Cer(d18:1/25:0)	-/-/0.747
**Early onset preeclampsia (<34w)**					
Odibo AO et al, 2011 [4]	Targeted LC-MS/MS	Blood (serum); 11-14w	Hydroxyhexanoylcarnitine, phenylalanine, glutamate, alanine		-/ -/ 0.84
Khalil AA et al, 2013 [7]	Targeted GC-MS; GC-MS/MS	Blood (plasma); 11-13w	asymmetric dimethylarginine/ L-arginine ratio; asymmetric dimethylarginine/ L-homoarginine ratio	L-arginine, L-homoarginine	-/ -/ -
Kuc S et al, 2014 [10]	Targeted UPLC-MS/MS	Blood (serum); 8-13w		Taurine, asparagine	-/ -/ -
Bahado-Singh RO et al, 2015 [12]	Untargeted ^{1}H-NMR	Blood (serum); 11-13w	2-hydroxybutyrate, 3-hydroxyisovalerate, citrate	Acetone, glycerol	0.75/ 0.74/ 0.835 (0.769-0.941)

For peer review only

For peer review only - http://bmjopen.bmj.com/site/about/guidelines.xhtml

Eichelberger KY et al, 2015 [13]	LC-MS	Blood (serum); 16-19w		Paraxanthine/caffeine ratio	-/ -/ -
Koster MPH et al, 2015 [14]	Targeted UPLC-MS/MS	Blood (serum); 8-13w	Hexanoylcarnitine, octenoylcarnitine, octanoylcarnitine, decenoylcarnitine, decanoylcarnitine, dodecanoylcarnitine, lauroylcarnitine, tetradecenoylcarnitine, hexadecenoylcarnitine	Isobutyrylcarnitine, linoleylcarnitine, stearoylcarnitine	-/ -/ -
Sovio U et al, 2020 [25]	Untargeted UPLC-MS/MS	Blood (serum); 12w<br>Blood (serum); 20w<br>Blood (serum); 28w	4-hydroxyglutamate		-/-/0.673<br>-/-/0.731<br>-/-/0.765
**Late onset preeclampsia (≥34w)**					
Kenny LC et al, 2010 [3]	Untargeted UPLC-MS	Blood (plasma); 14-16w	Monosaccharide(s), decanoylcarnitine, oleic acid, docosahexaenoic acid and/or docosatriynoic acid, gama-butyrolactone and/or oxolan-3-one, 2-oxovaleric acid and/or oxo-methylbutanoic acid, acetoacetic acid, hexadecenoyleicosatetraenoyl-Sn-glycerol, Di-(octadecadienoyl)-sn-glycerol, sphingosine 1-phosphate, sphinganine 1-phosphate, vitamin D3 derivatives	5-Hydroxytryptophan, methylglutaric acid and/or adipic acid	-/ -/ 0.92[c]

For peer review only - http://bmjopen.bmj.com/site/about/guidelines.xhtml

Kuc S et al, 2014 [10]	Targeted UPLC-MS/MS	Blood (serum); 8-13w		Glycylglycine	-/ -/ -
Koster MPH et al, 2015 [14]	Targeted UPLC-MS/MS	Blood (serum); 8-13w	Hexanoylcarnitine, octenoylcarnitine, octanoylcarnitine, decenoylcarnitine, decanoylcarnitine, dodecanoylcarnitine, tetradecenoylcarnitine	Linoleylcarnitine, palmitoylcarnitine, oleylcarnitine, stearoylcarnitine	-/ -/ -
Cantonwine DE et al, 2016 [17]	Targeted HPLC-MS	Urine; 4-16w	mono(2- ethyl-5-oxohexyl) phthalate, mono(2-ethyl-5-carboxypentyl) phthalate, Σ di(2-ethylhexyl) phthalate, mono-ethyl phthalate		-/ -/ -
		Urine; 33-38w	mono(2- ethyl-5-oxohexyl) phthalate, mono(2-ethyl-5-carboxypentyl) phthalate, Σ di(2-ethylhexyl) phthalate, Mono-*n*-butyl phthalate		-/ -/ -
Bahado-Singh RO et al, 2017 [19]	Untargeted ^{1}H-NMR	Blood (serum); 11-13w	Carnitine, pyruvate, acetone	-	0.30/ 0.80/ 0.629 (0.490-0.767)
Bahado-Singh RO et al, 2017b [20]	Untargeted ^{1}H-NMR, targeted LC-MS/MS	Blood (serum); 11-13w	-	Putrescine (LC-MS); urea, carnitine (H-NMR)	0.72/ 0.57/ 0.701
		Blood (serum); 32-33w	Methylhistidine, propylene glycol (H-NMR), citrate, hexose[d]	Serotonin (LC-MS)	0.74/ 0.72/ 0.761
Gong S et al, 2018	Untargeted	Blood (serum); 36w	N1, N12-diacetylspermine[e]	-	-

For peer review only - http://bmjopen.bmj.com/site/about/guidelines.xhtml

[23]	LC-MS/MS				
**Gestational hypertension**					
Ates S et al, 2016 [16]	Targeted LC-MS/MS	Blood (serum); 11-14w		Vitamin D (<10mg/dL)	0.45/0.54
Huo X et al, 2020 [26]	HPLC/ MS-MS	Blood (serum); 13-17w	Perfluoroheptanoic acid[f]		-/ -/ -
Harville EW et al, 2021 [31]	Untargeted UPLC-HR-MS	Blood (serum); 6-14w	Neomenthol-glucuronide, dibenzylamine, pilocarpine, 2,6-Di-*tert*-butyl-4-hydroxymethylphenol, 0.59_746.6045n, 8.66_762.1452m/z, 12.74_412.2842m/z		-/ - / 0.95[b]

AUC: area under the curve; w: weeks; GC-MS: gas-chromatography coupled to mass spectrometry; GC-FID: gas-chromatography coupled with a flame ionization detector; ^{1}H-NMR: hydrogen nuclear magnetic resonance; HPLC-MS: High Performance Liquid Chromatography- mass spectrometry; LC-MS: liquid- chromatography coupled to mass spectrometry; GC-TOF MS: gas chromatography time-of-flight mass spectrometry; LC-Orbitrap MS: liquid chromatography Orbitrap mass spectrometry. S, sensitivity; Sp, specificity; UPLC-MS: ultra- Performance Liquid Chromatography- mass spectrometry; 95% CI, 95% confidence interval.

[a]No statistical difference between preeclampsia and normotensive controls. [b]AUC for all metabolites model; some of them were not identified, thus they are presented as retention time and mass; it is unclear which metabolite was up or downregulated in preeclampsia cases. [c]Accuracy measures for the validation phase. [d]Unclear type of platform that has found this metabolite, or if it is increased or decreased in preeclampsia. [e]Higher preeclampsia risk with increasing N1, N12-diacetylspermine concentrations. [f] No statistical difference between preeclampsia normotensive controls.

For peer review only - http://bmjopen.bmj.com/site/about/guidelines.xhtml

Table 3 – Accuracy measures of metabolites and other pregnancy-related biomarkers.

Maternal specimen	Trimester of pregnancy	Metabolites	Additional variables considered in prediction models	AUC of metabolites + additional variables model (95% CI)
**Preeclampsia at any gestational age**				
Blood	1st	Dilinoleoyl-glycerol heptadecanoyl-2-hydroxy-sn-glycero-3-phosphocholine	PlGF	0.78 (0.69-0.88) [29]
		Ceramide(d18:1/25:0)[a]	Leptin	0.876 [32]
Blood	2nd	25OHD	VEGF + sFLT-1/PlGF	0.851 [5]
		Ceramide(d18:1/25:0)[a]	Leptin	0.892 [32]
Blood	3rd	4-hydroxyglutamate	s-Flt-1/PlGF	0.834 [25]
**Early onset preeclampsia (delivery <34w)**				
Blood	1st	Taurine	Prior risk+ MAP	0.93 [10]
		Stearoylcarnitine	Prior risk+ MAP	0.747 [14]
		3-hydroxyisovalerate, arginine, glycerol	UtPI	0.917 [12]
**Late onset preeclampsia (delivery ≥34w)**				
Blood	1st	Pyruvate, carnitine, glycerol	UtPI	0.722 [19]
Blood	1st	Stearoylcarnitine	Prior risk+ MAP	0.828 [14]
Blood	1st and 3rd	Urea, SM C18:1 (1st), Hexose, Citrate (3rd)	Maternal BMI (12w) + MAP (32w)	0.805 [20]

AUC: area under the curve; BMI: body mass index; MAP, mean arterial pressure; UtPI: uterine arteries pulsatility index; CI: confidence interval.

[a]Increased leptin/Ceramide(d18:1/25:0) ratio in preeclamptic women.

For peer review only

References

1 Chappell L. A longitudinal study of biochemical variables in women at risk of preeclampsia. *Am J Obstet Gynecol* 2002;**187**:127–36. doi:10.1067/mob.2002.122969

2 Powe CE, Seely EW, Rana S, *et al.* First trimester vitamin D, vitamin D binding protein, and subsequent preeclampsia. *Hypertension* 2010;**56**:758–63. doi:10.1161/HYPERTENSIONAHA.110.158238

3 Kenny LC, Broadhurst DI, Dunn W, *et al.* Robust early pregnancy prediction of later preeclampsia using metabolomic biomarkers. *Hypertension* 2010;**56**:741–9. doi:10.1161/HYPERTENSIONAHA.110.157297

4 Odibo AO, Goetzinger KR, Odibo L, *et al.* First-trimester prediction of preeclampsia using metabolomic biomarkers: a discovery phase study. *Prenat Diagn* 2011;**31**:990–4.

5 Woodham PC, Brittain JE, Baker AM, *et al.* Midgestation maternal serum 25-hydroxyvitamin D level and soluble fms-like tyrosine kinase 1/placental growth factor ratio as predictors of severe preeclampsia. *Hypertension* 2011;**58**:1120–5. doi:10.1161/HYPERTENSIONAHA.111.179069

6 Rijvers CAH, Marzano S, Winkens B, *et al.* Early-pregnancy asymmetric dimethylarginine (ADMA) levels in women prone to develop recurrent hypertension. *Pregnancy Hypertens* 2013;**3**:118–23. doi:10.1016/j.preghy.2013.01.001

7 Khalil AA, Tsikas D, Akolekar R, *et al.* Asymmetric dimethylarginine, arginine and homoarginine at 11-13 weeks gestation and preeclampsia: A case-control study. *J Hum Hypertens* 2013;**27**:38–43. doi:10.1038/jhh.2011.109

8 Diaz SO, Barros AS, Goodfellow BJ, *et al.* Second trimester maternal urine for the diagnosis of trisomy 21 and prediction of poor pregnancy outcomes. *J Proteome Res* 2013;**12**:2946–57. doi:10.1021/pr4002355

9 Kenny LC, Broadhurst DI, Hong W, *et al.* NMR Profiling in Early Pregnancy Reveals a Metabolomic Signature the Predicts the Subsequent Development Preeclampsia. *Reprod Sci* 2013;**20**:2641A. doi:10.1177/1933719113482088

10 Kuc S, Koster MPH, Pennings JLA, *et al.* Metabolomics profiling for identification of novel potential markers in early prediction of preeclampsia. *PLoS One* 2014;**9**. doi:10.1371/journal.pone.0098540

11 Wetta LA, Biggio JR, Cliver S, *et al.* Is midtrimester vitamin D status associated with spontaneous preterm birth and preeclampsia? *Am J Perinatol* 2014;**31**:541–6. doi:10.1055/s-0033-1356483

For peer review only - http://bmjopen.bmj.com/site/about/guidelines.xhtml

12 Bahado-Singh RO, Syngelaki A, Akolekar R, *et al.* Validation of metabolomic models for prediction of early-onset preeclampsia. *Am J Obstet Gynecol* 2015;**213**:530.e1-530.e10. doi:10.1016/j.ajog.2015.06.044

13 Eichelberger KY, Baker AM, Woodham PC, *et al.* Second-trimester maternal serum paraxanthine, CYP1A2 activity, and the risk of severe preeclampsia. *Obstet Gynecol* 2015;**126**:725–30. doi:10.1097/AOG.0000000000001041

14 Koster MPH, Vreeken RJ, Harms AC, *et al.* First-Trimester serum acylcarnitine levels to predict preeclampsia: A metabolomics approach. *Dis Markers* 2015;**2015**. doi:10.1155/2015/857108

15 Bilodeau JF, Qin Wei S, Larose J, *et al.* Plasma F2-isoprostane class VI isomers at 12-18 weeks of pregnancy are associated with later occurrence of preeclampsia. *Free Radic Biol Med* 2015;**85**:282–7. doi:10.1016/j.freeradbiomed.2015.05.012

16 Ates S, Sevket O, Ozcan P, *et al.* Vitamin D status in the first-trimester: Effects of vitamin D deficiency on pregnancy outcomes. *Afr Health Sci* 2016;**16**:36–43. doi:10.4314/ahs.v16i1.5

17 Cantonwine DE, Meeker JD, Ferguson KK, *et al.* Urinary concentrations of bisphenol A and phthalate metabolites measured during pregnancy and risk of preeclampsia. *Environ Health Perspect* 2016;**124**:1651–5. doi:10.1289/EHP188

18 Kiely ME, Zhang JY, Kinsella M, *et al.* Vitamin D status is associated with uteroplacental dysfunction indicated by pre-eclampsia and small-for-gestational-age birth in a large prospective pregnancy cohort in Ireland with low Vitamin D status. *Am J Clin Nutr* 2016;**104**:354–61. doi:10.3945/ajcn.116.130419

19 Bahado-Singh RO, Syngelaki A, Mandal R, *et al.* Metabolomic determination of pathogenesis of late-onset preeclampsia. *J Matern Neonatal Med* 2017;**30**:658–64. doi:10.1080/14767058.2016.1185411

20 Bahado-Singh R, Poon LC, Yilmaz A, *et al.* Integrated Proteomic and Metabolomic prediction of Term Preeclampsia. *Sci Rep* 2017;**7**:1–10. doi:10.1038/s41598-017-15882-9

21 Dobierzewska A, Soman S, Illanes SE, *et al.* Plasma cross-gestational sphingolipidomic analyses reveal potential first trimester biomarkers of preeclampsia. *PLoS One* 2017;**12**:1–16. doi:10.1371/journal.pone.0175118

22 Ye Y, Zhou Q, Feng L, *et al.* Maternal serum bisphenol A levels and risk of pre-eclampsia: A nested case-control study. *Eur J Public Health* 2017;**27**:1102–7. doi:10.1093/eurpub/ckx148

23 Gong S, Sovio U, Aye ILMH, *et al.* Placental polyamine metabolism differs by fetal sex, fetal growth restriction, and preeclampsia. *JCI Insight* 2018;**3**:1–15.

For peer review only

For peer review only - http://bmjopen.bmj.com/site/about/guidelines.xhtml

doi:10.1172/jci.insight.120723

24 Tamblyn JA, Jenkinson C, Larner DP, *et al.* Serum and urine vitamin D metabolite analysis in early preeclampsia. *Endocr Connect* 2018;**7**:199–210. doi:10.1530/EC-17-0308

25 Sovio U, McBride N, Wood AM, *et al.* 4-Hydroxyglutamate is a novel predictor of pre-eclampsia. *Int J Epidemiol* 2020;**49**:301–11. doi:10.1093/ije/dyz098

26 Huo X, Huang R, Gan Y, *et al.* Perfluoroalkyl substances in early pregnancy and risk of hypertensivedisorders of pregnancy: A prospective cohort study. *Environ Int* 2020;**138**. doi:10.1016/j.envint.2020.105656

27 Rylander L, Lindh CH, Hansson SR, *et al.* Per- and Polyfluoroalkyl Substances in Early Pregnancy and Risk for Preeclampsia: A Case-Control Study in Southern Sweden. *Toxics* 2020;**8**. doi:10.3390/toxics8020043

28 Lee SM, Kang Y, Lee EM, *et al.* Metabolomic biomarkers in midtrimester maternal plasma can accurately predict the development of preeclampsia. *Sci Rep* 2020;**10**:16142. doi:10.1038/s41598-020-72852-4

29 Kenny LC, Thomas G, Poston L, *et al.* Prediction of preeclampsia risk in first time pregnant women: Metabolite biomarkers for a clinical test. *PLoS One* 2020;**15**:1–19. doi:10.1371/journal.pone.0244369

30 Shanmugalingam R, Wang X, Motum P, *et al.* The 15-Epilipoxin-A4 Pathway with Prophylactic Aspirin in PreventingPreeclampsia: A Longitudinal Cohort Study. *J Clin Endocrinol Metab* 2020;**105**. doi:10.1210/clinem/dgaa642

31 Harville EW, Li Y-YY-Y, Pan K, *et al.* Untargeted analysis of first trimester serum to reveal biomarkers of pregnancy complications: a case-control discovery phase study. *Sci Rep* 2021;**11**:3468. doi:10.1038/s41598-021-82804-1

32 Huang Q, Hao S, You J, *et al.* Early-pregnancy prediction of risk for pre-eclampsia using maternal blood leptin/ceramide ratio: Discovery and confirmation. *BMJ Open* 2021;**11**. doi:10.1136/bmjopen-2021-050963

For peer review only

For peer review only - http://bmjopen.bmj.com/site/about/guidelines.xhtml

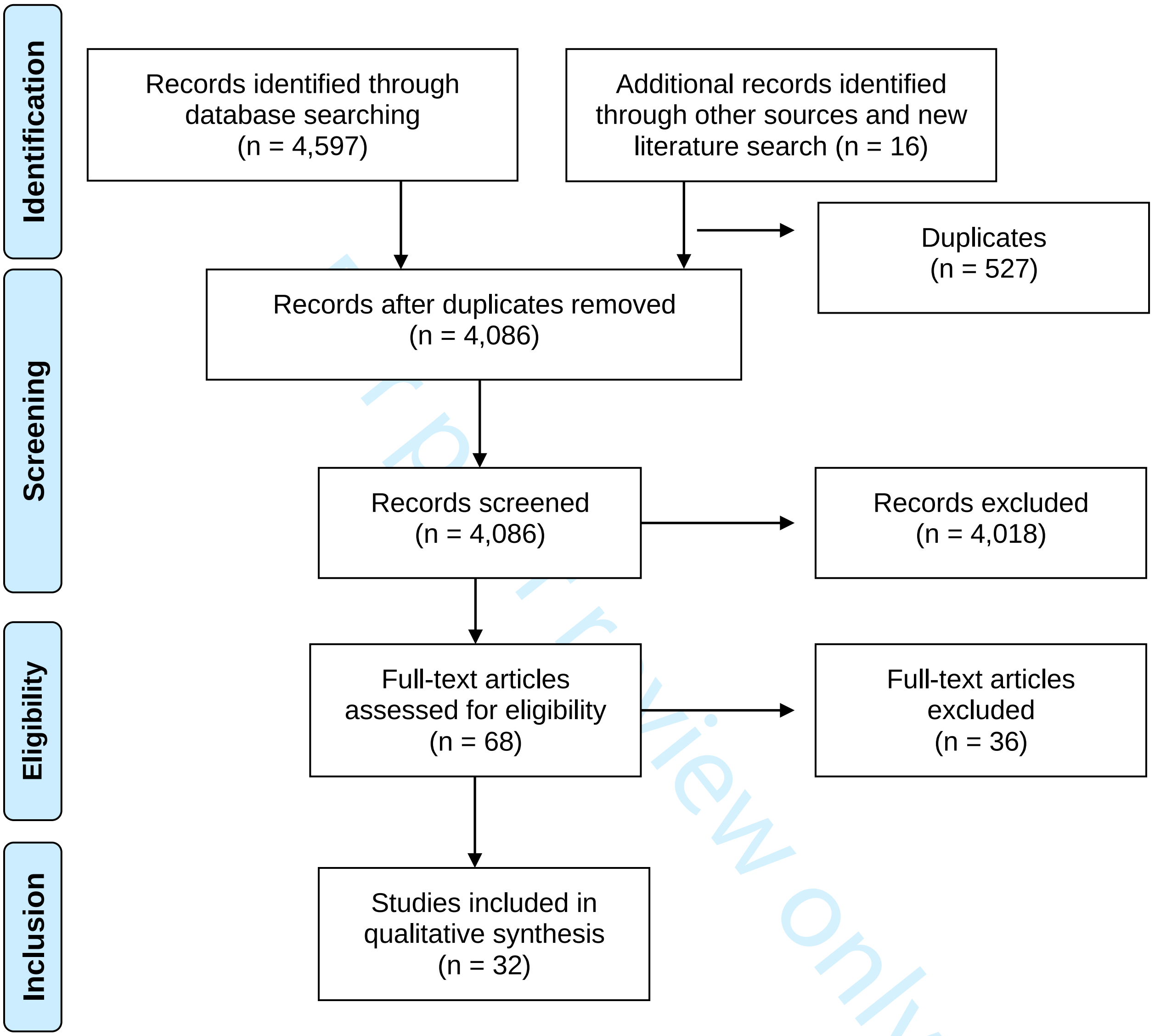

From: Moher D, Liberati A, Tetzlaff J, Altman DG, The PRISMA Group (2009). *P*referred *R*eporting *I*tems for *S*ystematic Reviews and *M*eta-*A*nalyses: The PRISMA Statement. PLoS Med 6(7): e1000097. doi:10.1371/journal.pmed1000097

For more information, visit www.prisma-statement.org.

For peer review only - http://bmjopen.bmj.com/site/about/guidelines.xhtml

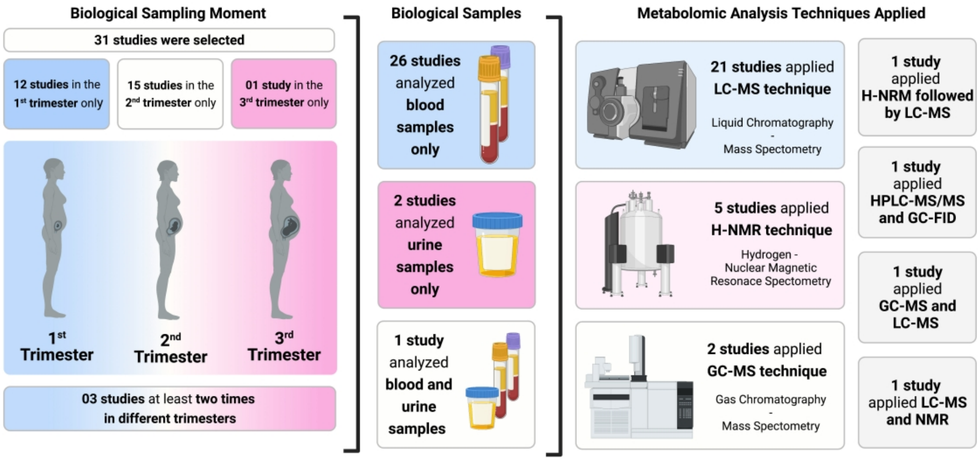

Figure 2

419x199mm (300 x 300 DPI)

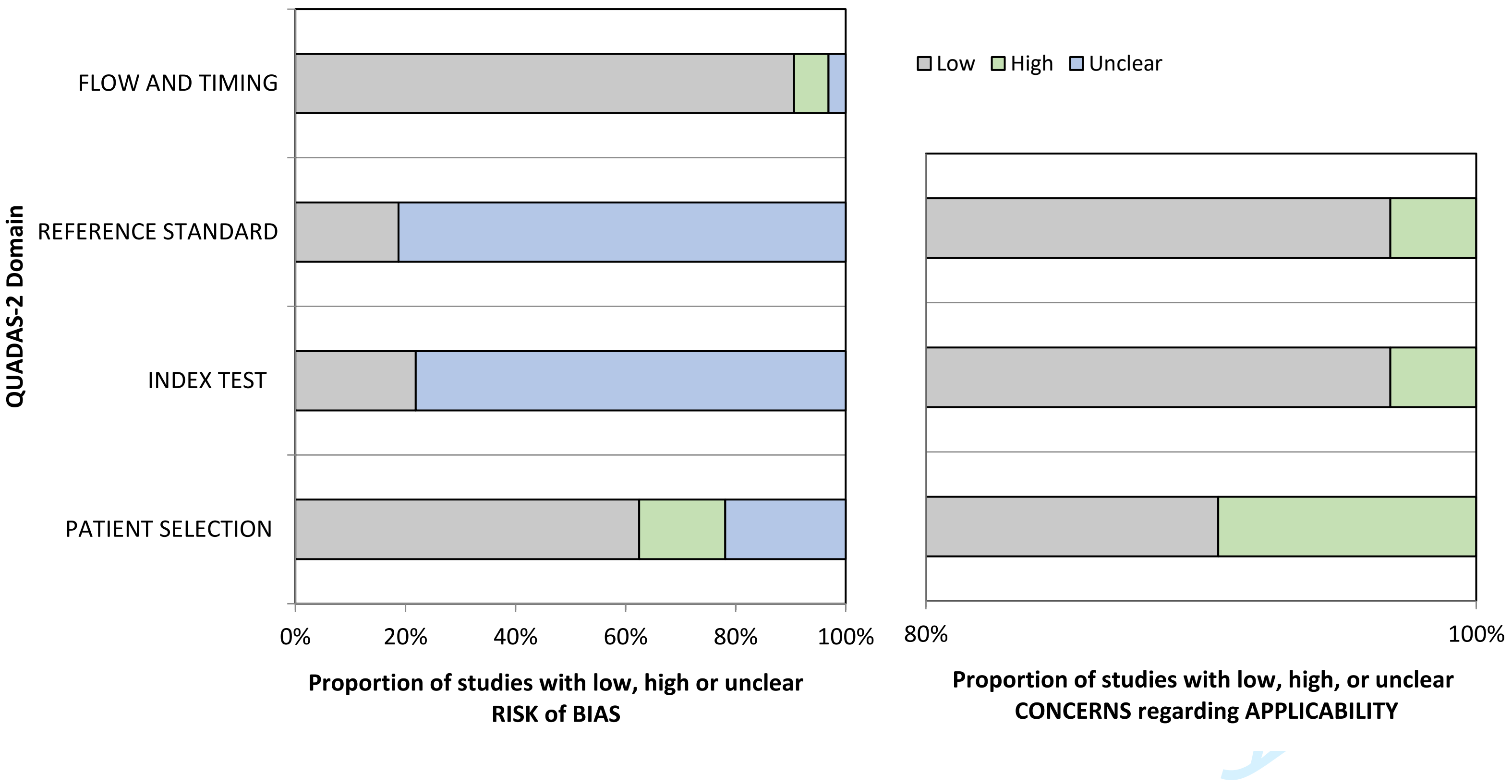
Low
High
Unclear
QUADAS-2 Domain
FLOW AND TIMING
REFERENCE STANDARD
INDEX TEST
PATIENT SELECTION
0%
20%
40%
60%
80%
100%
Proportion of studies with low, high or unclear RISK of BIAS
80%
100%
Proportion of studies with low, high, or unclear CONCERNS regarding APPLICABILITY

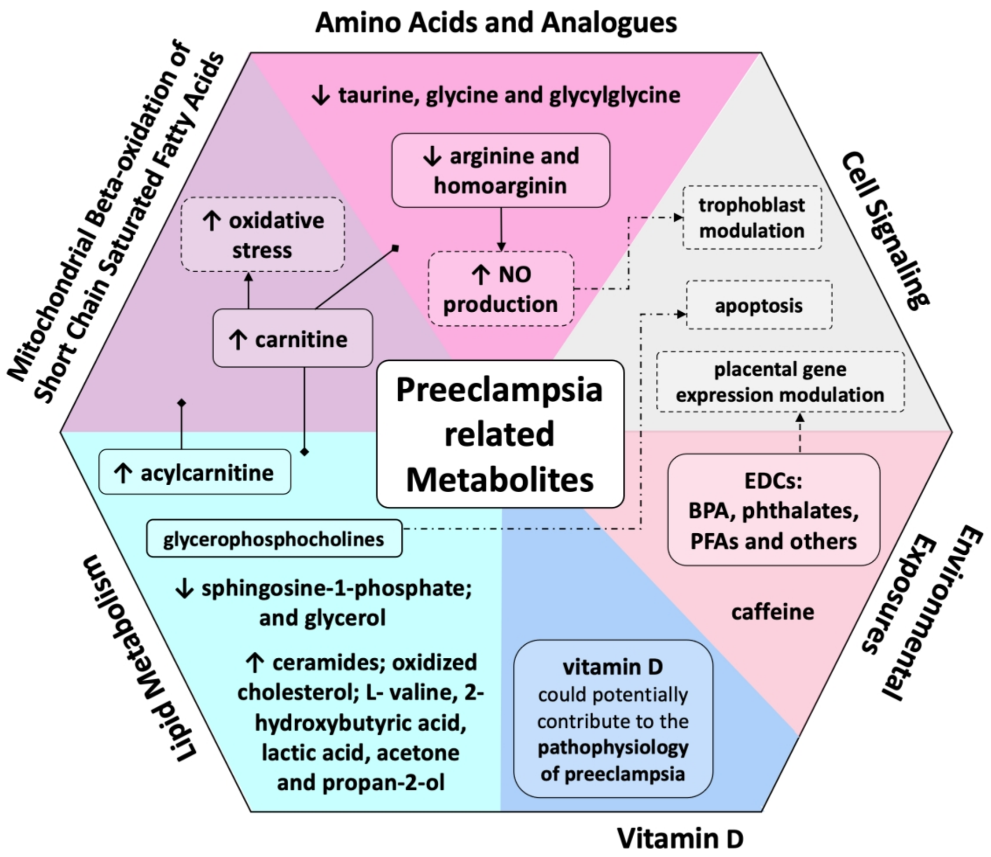

Figure 4

355x313mm (144 x 144 DPI)

Prediction of pregnancy-related hypertensive disorders using metabolomics: a systematic review

Jussara Mayrink, Debora Farias B. Leite, Guilherme M Nóbrega, Maria Laura Costa, José Guilherme Cecatti

Supplementary material 1 – PRISMA checklist

Section/topic	#	Checklist item	Section where the item was reported
**Title**			
Title	1	Identify the report as a systematic review, meta-analysis, or both.	Page 1/title
**ABSTRACT**			
Structured summary	2	Provide a structured summary including, as applicable: background; objectives; data sources; study eligibility criteria, participants, and interventions; study appraisal and synthesis methods; results; limitations; conclusions and implications of key findings; systematic review registration number.	Page 2/abstract
**INTRODUCTION**			
Rationale	3	Describe the rationale for the review in the context of what is already known.	Page 4
Objectives	4	Provide an explicit statement of questions being addressed with reference to participants, interventions, comparisons, outcomes, and study design (PICOS).	Page 5; Lines 11-12
**METHODS**			
Protocol and registration	5	Indicate if a review protocol exists, if and where it can be accessed (e.g., Web address), and, if available, provide registration information including registration number.	Page 3, Line 3<br>Page 5, Line 15
Eligibility criteria	6	Specify study characteristics (e.g., PICOS, length of follow-up) and report characteristics (e.g., years considered, language, publication status) used as criteria for eligibility, giving rationale.	Page 5, Lines 20-28<br>Page 6, Lines 1-13<br>Page 7, Lines 6-13
Information sources	7	Describe all information sources (e.g., databases with dates of coverage, contact with study authors to identify additional studies) in the search and date last searched.	Page 6, Lines 14-23<br>Page 8, Lines 10-11
Search	8	Present full electronic search strategy for at least one database, including any limits used, such that it could be repeated.	Supplementary Material 2
Study selection	9	State the process for selecting studies (i.e., screening, eligibility, included in systematic review, and, if applicable, included in the meta-analysis).	Page 7, Lines 16-27<br>Page 8, Lines 1-7
Data collection process	10	Describe method of data extraction from reports (e.g., piloted forms, independently, in duplicate) and any processes for obtaining and confirming data from investigators.	Page 7, Lines 16-27<br>Page 8, Lines 1-7
Data items	11	List and define all variables for which data were sought (e.g., PICOS, funding sources) and any assumptions and simplifications made.	Page 7, Lines 16-27<br>Page 8, Lines 1-7
Risk of bias in individual studies	12	Describe methods used for assessing risk of bias of individual studies (including specification of whether this was done at the study or outcome level), and how this information is to be used in any data synthesis.	Page 7, Lines 16-20
Summary measures	13	State the principal summary measures (e.g., risk ratio, difference in means).	Page 8, Lines 3-7
Synthesis of results	14	Describe the methods of handling data and combining results of studies, if done, including measures of	NA

For peer review only

Section/topic	#	Checklist item	Section where the item was reported
		consistency (e.g., I^2) for each meta-analysis.	
Risk of bias across studies	15	Specify any assessment of risk of bias that may affect the cumulative evidence (e.g., publication bias, selective reporting within studies).	NA
Additional analyses	16	Describe methods of additional analyses (e.g., sensitivity or subgroup analyses, meta-regression), if done, indicating which were pre-specified.	NA
RESULTS			
Study selection	17	Give numbers of studies screened, assessed for eligibility, and included in the review, with reasons for exclusions at each stage, ideally with a flow diagram.	Page 8, Lines 11-27
Study characteristics	18	For each study, present characteristics for which data were extracted (e.g., study size, PICOS, follow-up period) and provide the citations.	Page 11, Line 11 – Page 15, Line 5
Risk of bias within studies	19	Present data on risk of bias of each study and, if available, any outcome level assessment (see item 12).	Page 15, Lines 6-15
Results of individual studies	20	For all outcomes considered (benefits or harms), present, for each study: (a) simple summary data for each intervention group (b) effect estimates and confidence intervals, ideally with a forest plot.	NA
Synthesis of results	21	Present results of each meta-analysis done, including confidence intervals and measures of consistency.	NA
Risk of bias across studies	22	Present results of any assessment of risk of bias across studies (see Item 15).	NA
Additional analysis	23	Give results of additional analyses, if done (e.g., sensitivity or subgroup analyses, meta-regression [see Item 16]).	NA
**DISCUSSION**			
Summary of evidence	24	Summarize the main findings including the strength of evidence for each main outcome; consider their relevance to key groups (e.g., healthcare providers, users, and policy makers).	Page 16, Line 6 – Page 19, Line 30
Limitations	25	Discuss limitations at study and outcome level (e.g., risk of bias), and at review-level (e.g., incomplete retrieval of identified research, reporting bias).	Page 20, Lines 15-28
Conclusions	26	Provide a general interpretation of the results in the context of other evidence, and implications for future research.	Page 21, Lines 1-20
**FUNDING**			
Funding	27	Describe sources of funding for the systematic review and other support (e.g., supply of data); role of funders for the systematic review.	Page 29, Lines 8-15

For peer review only

Supplementary material 2 – Full search strategy for each database.

PUBMED

(((((((((((preeclampsia) OR (pre-eclampsia)) OR (eclampsia)) OR (gestational hypertension)) OR (white coat hypertension)) OR (severe preeclampsia)) OR (late preeclampsia)) OR (early preeclampsia)) OR (pregnancy-induced hypertension)) OR (hypertensive syndromes of pregnancy)) AND (((((((((((((((metabolomic*) OR (metabonomic*)) OR (metabolit*)) OR (HNMR)) OR (proton NMR)) OR (proton nuclear magnetic resonance)) OR (liquid chromatogra*)) OR (gas chromatogra*)) OR (UPLC)) OR (HPLC)) OR (high pressure liquid chromatograph*)) OR (ultra-performance liquid chromatograph*)) OR (ultra performance liquid chromatograph*)) OR (lipidomic*)) OR (mass spectrometr*))) AND (((screen*) OR (predict*)) OR (profil*)))

EMBASE

('preeclampsia' OR 'pre-eclampsia' OR 'eclampsia' OR 'gestational hypertension' OR 'white coat hypertension' OR 'severe preeclampsia' OR 'late preeclampsia' OR 'early preeclampsia' OR 'pregnancy-induced hypertension' OR 'hypertensive syndromes of pregnancy') AND ('metabolomic*' OR 'metabonomic*' OR 'metabolit*' OR 'HNMR' OR 'proton NMR' OR 'proton nuclear magnetic resonance' OR 'liquid chromatogra*' OR 'gas chromatogra*' OR 'UPLC' OR 'HPLC' OR 'high pressure liquid chromatograph*' OR 'ultra-performance liquid chromatograph*' OR 'ultra performance liquid chromatograph*' OR 'lipidomic*' OR 'mass spectrometr*') AND ('screen*' OR 'predict*' OR 'profil*').

SCOPUS

((TITLE-ABS-KEY (screen*) OR TITLE-ABS-KEY (predict*) OR TITLE-ABS-KEY (profil*))) AND ((TITLE-ABS-KEY (preeclampsia) OR TITLE-ABS-KEY (pre-eclampsia) OR TITLE-ABS-KEY (eclampsia) OR TITLE-ABS-KEY (gestational AND hypertension) OR TITLE-ABS-KEY (white AND coat AND hypertension) OR TITLE-ABS-KEY (severe AND preeclampsia) OR TITLE-ABS-KEY (late AND preeclampsia) OR TITLE-ABS-KEY (early AND preeclampsia) OR TITLE-ABS-KEY (pregnancy- AND induced AND hypertension) OR TITLE-ABS-KEY (hypertensive AND syndromes AND of AND pregnancy))) AND ((TITLE-ABS-KEY (metabolomic) OR TITLE-ABS-KEY (metabonomic*) OR TITLE-ABS-KEY (metabolit*) OR TITLE-ABS-KEY (hnmr) OR TITLE-ABS-KEY (proton AND nmr) OR TITLE-ABS-KEY (proton AND nuclear AND magnetic AND resonance) OR TITLE-ABS-KEY (liquid AND chromatogra*) OR TITLE-ABS-KEY (gas AND chromatogra*) OR TITLE-ABS-KEY (uplc) OR TITLE-ABS-KEY (hplc) OR TITLE-ABS-KEY (high AND pressure AND liquid AND chromatograph*) OR TITLE-ABS-KEY (ultra-performance AND liquid AND chromatograph*) OR TITLE-ABS-KEY (ultra AND performance AND liquid AND chromatograph*) OR TITLE-ABS-KEY (lipidomic*) OR TITLE-ABS-KEY (mass AND spectrometr*)))

Web of Knowledge

#1

For peer review only

((((((((ALL=(preeclampsia)) OR ALL=(pre-eclampsia)) OR ALL=(eclampsia)) OR ALL=(gestational hypertension)) OR ALL=(white coat hypertension)) OR ALL=(severe preeclampsia)) OR ALL=(late preeclampsia)) OR ALL=(early preeclampsia)) OR ALL=(pregnancy- induced hypertension)) OR ALL=(hypertensive syndromes of pregnancy)

#2

(((((((((((((ALL=(metabolomic*)) OR ALL=(metabonomic*)) OR ALL=(metabolit*)) OR ALL=(HNMR)) OR ALL=(proton NMR)) OR ALL=(proton nuclear magnetic resonance)) OR ALL=(liquid chromatogra*)) OR ALL=(gas chromatogra*)) OR ALL=(UPLC)) OR ALL=(HPLC)) OR ALL=(high pressure liquid chromatograph*)) OR ALL=(ultra-performance liquid chromatograph*)) OR ALL=(ultra performance liquid chromatograph*)) OR ALL=(lipidomic*)) OR ALL=(mass spectrometr*)

#3

((ALL=(screen*)) OR ALL=(predict*)) OR ALL=(profil*)

#4

#1 AND #2 AND #3

LILACS

('preeclampsia' OR 'pre-eclampsia' OR 'eclampsia' OR 'gestational hypertension' OR 'white coat hypertension' OR 'severe preeclampsia' OR 'late preeclampsia' OR 'early preeclampsia' OR 'pregnancy-induced hypertension' OR 'hypertensive syndromes of pregnancy') AND ('metabolomic*' OR 'metabonomic*' OR 'metabolit*' OR 'HNMR' OR 'proton NMR' OR 'proton nuclear magnetic resonance' OR 'liquid chromatogra*' OR 'gas chromatogra*' OR 'UPLC' OR 'HPLC' OR 'high pressure liquid chromatograph*' OR 'ultra-performance liquid chromatograph*' OR 'ultra performance liquid chromatograph*' OR 'lipidomic*' OR 'mass spectrometr*') AND ('screen*' OR 'predict*' OR 'profil*')

SciELO

('preeclampsia' OR 'pre-eclampsia' OR 'eclampsia' OR 'gestational hypertension' OR 'white coat hypertension' OR 'severe preeclampsia' OR 'late preeclampsia' OR 'early preeclampsia' OR 'pregnancy-induced hypertension' OR 'hypertensive syndromes of pregnancy') AND ('metabolomic*' OR 'metabonomic*' OR 'metabolit*' OR 'HNMR' OR 'proton NMR' OR 'proton nuclear magnetic resonance' OR 'liquid chromatogra*' OR 'gas chromatogra*' OR 'UPLC' OR 'HPLC' OR 'high pressure liquid chromatograph*' OR 'ultra-performance liquid chromatograph*' OR 'ultra performance liquid chromatograph*' OR 'lipidomic*' OR 'mass spectrometr*') AND ('screen*' OR 'predict*' OR 'profil*')

DARE and HTA

('preeclampsia' OR 'pre-eclampsia' OR 'eclampsia' OR 'gestational hypertension' OR 'white coat hypertension' OR 'severe preeclampsia' OR 'late preeclampsia' OR 'early preeclampsia' OR 'pregnancy-induced hypertension' OR 'hypertensive syndromes of pregnancy') AND ('metabolomic*' OR 'metabonomic*' OR 'metabolit*' OR 'HNMR' OR 'proton NMR' OR 'proton nuclear magnetic resonance' OR 'liquid chromatogra*' OR 'gas chromatogra*' OR 'UPLC' OR 'HPLC' OR 'high pressure liquid

For peer review only

chromatograph*' OR 'ultra-performance liquid chromatograph*' OR 'ultra performance liquid chromatograph*' OR 'lipidomic*' OR 'mass spectrometr*') AND ('screen*' OR 'predict*' OR 'profil*') IN DARE, HTA

For peer review only

Supplementary Material 3 - Excluded studies and reasons for exclusion.

Authors	DOI	Reason for exclusion
Kenny LC et al, 2008	10.1177/1933719108316908	Cross-sectional design.
Aris A et al, 2009	10.1016/j.placenta.2009.01.003	Non metabolomics research.
Kenny LC et al, 2009		Conference abstract. Duplicated data. Please check Kenny LC et al (10.1161/HYPERTENSIONAHA.110.157297)
Baker AM et al, 2010	10.1210/jc.2010-0996	Duplicated data. Please check Woodham PC et al, 2011 (10.1161/HYPERTENSIONAHA.111.179069)
Bahado-Singh RO et al, 2012	10.3109/14767058.2012.680254	Duplicated data. Please check Bahado-Singh RO et al, 2015 (10.1016/j.ajog.2015.06.044)
Austdal M et al, 2013	10.1016/j.preghy.2013.04.030	Cross-sectional design.
Bahado-Singh RO et al, 2013	10.1016/j.ajog.2012.11.003	Duplicated data. Please check Bahado-Singh RO et al, 2017 (10.1080/14767058.2016.1185411)
Senyavina NV et al, 2013	10.1007/s10517-013-2225-y	Cross-sectional design.
Bodnar et al, 2014	10.1097/EDE.0000000000000039	Imputation of data obtained in a small subset to the whole sample.
Austdal M et al, 2014	10.1371/journal.pone.0091923	Duplicated data. Please check Austdal M et al, 2015 (10.3390/ijms160921520)
Austdal M et al, 2015	10.1016/j.placenta.2015.10.019	Cross-sectional design.
Austdal M et al, 2015	10.3390/ijms160921520	Comparison of preeclampsia patients with women with normal blood pressure and gestational hypertension together.
Riley J et al, 2016		Conference abstract. Duplicated data. Please check Odibo A et al 2011 (10.1002/pd.2822)
Chen T et al, 2017	10.1016/j.bbrc.2017.02.032	Cross-sectional design.
Kelly RS et al, 2017	10.1007/s11306-016-1149-8	Randomized clinical trial.
Powell KL et al, 2018	10.1016/j.placenta.2018.04.005	Cross-sectional design.
Naase et al, 2018	10.1093/humrep/33.Supplement_1.1	Only mass spectrometry data.
Kawasaki K et al, 2019	10.1161/HYPERTENSIONAHA.118.12389	Cross-sectional design.
Liu Y et al, 2019	10.1111/1440-1681.13095	Cross-sectional design.
Sander KN et al, 2019	10.1007/s11306-019-1600-8	Cross-sectional design.
Chaudhry SH et al, 2019	10.1186/s12884-019-2219-5	Non metabolomics research.
Lewandowska M et al, 2019	10.3390/nu11051028	No specific data on preeclampsia.

For peer review only

Vieira MC et al, 2019	10.1177/1933719119834079	Randomized clinical trial. Only mass spectrometry data.
Odenkirk MT et al, 2020	10.1039/x0xx00000x	Cross-sectional design.
Ferranti et al, 2020	10.1155/2020/1515321	Mention of altered metabolic pathways, but not metabolites.
Kelly C et al, 2020	10.3390/nu12072048	Non metabolomics research.
Kyozuka H et al, 2020	10.1038/s41598-020-70974-3	No specific data on preeclampsia.
McBride N et al, 2020	10.1186/s12916-020-01819-z	Randomized clinical trial.
Jääskeläinen T et al, 2021	10.1007/s11306-020-01752-5	No specific data on preeclampsia compared to normotensive women.
Rodriguez et al, 2021	10.1016/j.arcmed.2021.01.012	Cross-sectional design.
McBride N et al, 2021	10.3390/ metabo11080530	Mention of accuracy measures of a group of metabolites, and of metabolites combined with other maternal risk factors, but do not mention specific metabolites.
Kivelä J et al, 2021	10.1210/clinem/dgab475	Randomized clinical trial.
Zhang-Rutledge K et al, 2021	10.1016/j.ajog.2020.12.267	Only mass spectrometry data.
Wahab et al, 2022	10.3390/ metabo12010013	No specific data on preeclampsia compared to normotensive women.
Herlambang H et al, 2022	10.1186/s12884-021-04313-3	Cross-sectional design.
Liu H, et al	10.1007/s12011-021-02988-5	Cross-sectional design.

For peer review only

Supplementary Material 4 - Predictive metabolites of hypertensive disorders of pregnancy summarized according to their superclass, chemical class, subclass, and metabolic pathways.

Predictive metabolites	Chemical class	Chemical subclass	Metabolic pathway or process	Involved with
		Organic acids and derivatives		
N1, N12-diacetylspermine	Carboximidic acids and derivatives	Carboximidic acids	NA	Late onset preeclampsia[1]
Acetamide	Carboximidic acids and derivatives	Carboximidic acids	NA	Preeclampsia [2]
Acetate	Carboxylic acids and derivatives	Carboxylic acids	Glycolysis, gluconeogenesis; pyruvate, propanoate, taurine and hypotaurine metabolism	Preeclampsia [2,3]
3-methylhistidine	Carboxylic acids and derivatives	Amino acids, peptides, and analogues	Beta-alanine, histidine and methylhistidine metabolism	Preeclampsia [3]
Methylhistidine	Carboxylic acids and derivatives	Amino acids, peptides, and analogues	Histidine Metabolism	Late onset preeclampsia [4]
Alanine	Carboxylic acids and derivatives	Amino acids, peptides, and analogues	Glutathione metabolism, glycine and serine metabolism, tryptophan metabolism, urea cycle, glutamate metabolism, glucose-alanine cycle	Preeclampsia [5], early onset [5] preeclampsia
Betaine	Carboxylic acids and derivatives	Amino acids, peptides, and analogues	Betaine metabolism, glycine and serine metabolism, methionine metabolism	Preeclampsia [2]
Asymmetric dimethylarginine	Carboxylic acids and derivatives	Amino acids, peptides, and analogues	NA	Preeclampsia [6], early onset [7] preeclampsia
L-arginine	Carboxylic acids and derivatives	Amino acids, peptides, and analogues	Urea cycle, arginine and proline metabolism, glycine and serine metabolism, transcription/translation	Preeclampsia [6], early onset [7] preeclampsia
L-homoarginine	Carboxylic acids and derivatives	Amino acids, peptides, and analogues	NA	Early onset [7] preeclampsia

For peer review only - http://bmjopen.bmj.com/site/about/guidelines.xhtml

Creatinine	Carboxylic acids and derivatives	Amino acids, peptides, and analogues	Arginine and proline metabolism	Preeclampsia [2,3]
Glutamine	Carboxylic acids and derivatives	Amino acids, peptides, and analogues	Pyrimidine metabolism, glutamate metabolism, purine metabolism, transcription/translation, urea cycle	Preeclampsia [2,3]
Glutamate	Carboxylic acids and derivatives	Amino acids, peptides, and analogues	Alanine metabolism, histidine metabolism, ammonia recycling, cysteine metabolism, arachidonic acid metabolism	Preeclampsia [5], early onset [5] preeclampsia
4-hydroxyglutamate	Carboxylic acids and derivatives	Amino acids, peptides, and analogues	Arginine and proline metabolism	Preeclampsia [8]
Glycine	Carboxylic acids and derivatives	Amino acids, peptides, and analogues	Ammonia recycling, porphyrin metabolism, glutathione metabolism, glycine and serine metabolism	Preeclampsia [2]
Glycylglycine	Carboxylic acids and derivatives	Amino acids, peptides, and analogues	NA	Late onset [9] preeclampsia
Isoleucine	Carboxylic acids and derivatives	Amino acids, peptides, and analogues	Valine, leucine and isoleucine biosynthesis and degradation	Preeclampsia [2,3]
Asparagine	Carboxylic acids and derivatives	Amino acids, peptides, and analogues	Alanine, aspartate and glutamate metabolism; protein digestion and absorption	Preeclampsia [10]
Methionine	Carboxylic acids and derivatives	Amino acids, peptides, and analogues	Betaine metabolism, glycine and serine metabolism; spermidine and spermine biosynthesis	Preeclampsia [2]
N,N-Dimethylglycine	Carboxylic acids and derivatives	Amino acids, peptides, and analogues	Glycine, serine, and threonine metabolism	Preeclampsia [10]
Ornithine	Carboxylic acids and derivatives	Amino acids, peptides, and analogues	Urea cycle, arginine and proline metabolism; glycine and serine metabolism; Spermidine and Spermine	Preeclampsia [2]

For peer review only - http://bmjopen.bmj.com/site/about/guidelines.xhtml

			Biosynthesis	
Phenylacetylglutamine	Carboxylic acids and derivatives	Amino acids, peptides, and analogues	Phenylacetate metabolism, phenylalanine metabolism	Preeclampsia [3]
Phenylalanine	Carboxylic acids and derivatives	Amino acids, peptides, and analogues	Phenylalanine, tyrosine and tryptophan biosynthesis; phenylalanine metabolismo;	Preeclampsia [5], early onset [5] preeclampsia
Proline	Carboxylic acids and derivatives	Amino acids, peptides, and analogues	Arginine and proline metabolism	Preeclampsia [2]
Tyrosine	Carboxylic acids and derivatives	Amino acids, peptides, and analogues	Tyrosine metabolism, phenylalanine and tyrosine metabolism, catecholamine biosynthesis, transcription/translation	Preeclampsia [3]
Valine	Carboxylic acids and derivatives	Amino acids, peptides, and analogues	Valine, leucine and isoleucine biosynthesis and degradation; propanoate metabolism	Preeclampsia [2]
Formate	Carboxylic acids and derivatives	Carboxylic acids	Folate metabolism, pterine biosynthesis	Preeclampsia [3]
Fumarate	Carboxylic acids and derivatives	Dicarboxylic acids and derivatives	Phenylalanine and tyrosine metabolism, arginine and proline metabolism, aspartate metabolism, urea cycle, mitochondrial electron transport chain	Preeclampsia [3]
Succinate	Carboxylic acids and derivatives	Dicarboxylic acids and derivatives	Citrate cycle, oxidative phosphorylation; alanine, aspartate and glutamate metabolism; pyruvate metabolism; cAMP signalling pathway; GABAergic synapse; glucagon signalling pathway	Preeclampsia [3]
Cis-aconitate	Carboxylic acids and derivatives	Tricarboxylic acids and derivatives	Citric acid cycle	Preeclampsia [3]
Citrate	Carboxylic acids and derivatives	Tricarboxylic acids and derivatives	Citric acid cycle, transfer of acetyl groups into mitochondria	Preeclampsia [3], early onset [11] preeclampsia, late onset [4] preeclampsia

For peer review only - http://bmjopen.bmj.com/site/about/guidelines.xhtml

For peer review only

2-hydroxybutyrate	Hydroxy acids and derivatives	Alpha hydroxy acids and derivatives	Propanoate metabolism	Early onset [11] preeclampsia
2-ketoglutarate	Keto acids and derivatives	Gamma-keto acids and derivatives	Citric acid cycle, gluconeogenesis, oxidation of branched-chain fatty acids, urea cycle, branched-chain amino acids degradation	Preeclampsia [3]
2-oxovaleric acid	Keto acids and derivatives	Short-chain keto acids and derivatives	NA	Late onset [12] preeclampsia
Acetoacetic acid	Keto acids and derivatives	Short-chain keto acids and derivatives	Ketone body metabolism, fatty acid biosynthesis, branched-chain amino acids degradation, tyrosine metabolism, phenylalanine and tyrosine metabolism	Late onset [12] preeclampsia
Oxo-methylbutanoic acid	Keto acids and derivatives	Short-chain keto acids and derivatives	Cell signaling; valine, leucine and isoleucine Degradation	Late onset [12] preeclampsia
Pyruvate	Keto acids and derivatives	Alpha keto acids and derivatives	Urea Cycle, glucose-alanine cycle, glycine and serine Metabolism, pyruvate metabolism, alanine metabolism	Late onset [13] preeclampsia
Urea	Organic carbonic acids and derivatives	Ureas	Urea cycle; D-arginine and D-ornithine metabolism; arginine and proline metabolism	Late onset [4] preeclampsia
Taurine	Organic sulfonic acids and derivatives	Organosulfonic acids and derivatives	Taurine and hypotaurine metabolism	Early onset [9] preeclampsia
Indoxyl sulphate	Organic sulfuric acids and derivatives	Arylsulfates	NA	Preeclampsia [3]
p-cresol sulphate	Organic sulfuric acids and derivatives	Arylsulfates	NA	Preeclampsia [3]
		Lipids and lipid-likes molecules		
~~Ceramide 14:0~~	~~Ceramides~~	~~N-acylsphingosines (ceramides)~~	~~NA~~	~~Preeclampsia [14]~~

For peer review only - http://bmjopen.bmj.com/site/about/guidelines.xhtml

Ceramide 24:0	Ceramides	N-acylsphingosines (ceramides)	NA	Preeclampsia [14]
3-hydroxyisovalerate	Fatty acyls	Fatty acids and conjugates	Lipid transport, metabolism, and peroxidation; fatty acid metabolism	Early onset [11] preeclampsia
Adipic acid	Fatty acyls	Fatty acids and conjugates	Lipid transport, metabolism, and peroxidation; fatty acid metabolism	Late onset [12] preeclampsia
Docosapentaenoic acid	Fatty acyls	Fatty acids and conjugates	Lipid transport, metabolism, and peroxidation; fatty acid metabolism; cell signalling; alpha linolenic acid and linoleic acid metabolism	Preeclampsia [14]
Docosahexaenoic acid	Fatty acyls	Fatty acids and conjugates	Lipid transport, metabolism, and peroxidation; fatty acid metabolism; cell signalling; alpha linolenic acid and linoleic acid metabolism	Preeclampsia [14], Late onset [12] preeclampsia
Docosatriynoic acid	Fatty acyls	Fatty acids and conjugates	Lipid transport, metabolism, and peroxidation; fatty acid metabolism; cell signalling	Late onset [12] preeclampsia
Eicosatrienoic acid	Fatty acyls	Fatty acids and conjugates	Lipid transport, metabolism, and peroxidation; fatty acid metabolism; cell signalling	Preeclampsia [14]
Eicosatetraenoic acid	Fatty acyls	Fatty acids and conjugates	Lipid transport, metabolism, and peroxidation; fatty acid metabolism; cell signalling; alpha linolenic acid and linoleic acid metabolism.	Preeclampsia [14]
Eicosapentaenoic acid	Fatty acyls	Fatty acids and conjugates	Lipid transport, metabolism, and peroxidation; fatty acid metabolism; cell signalling; alpha linolenic acid and linoleic acid metabolism.	Preeclampsia [14]

For peer review only

Methylglutaric acid	Fatty acyls	Fatty acid and conjugates	Lipid transport, metabolism, and peroxidation; fatty acid metabolism; cell signalling	Late onset [12] preeclampsia
Oleic acid	Fatty acyls	Fatty acids and conjugates	Fatty acid biosynthesis, biosynthesis of unsaturated fatty acids	Late onset [12] preeclampsia
Decanoylcarnitine	Fatty acyls	Fatty acid esters	Lipid transport and peroxidation; fatty acid metabolism; cell signaling	Early onset [15] preeclampsia, late onset [12] preeclampsia
Decenoylcarnitine	Fatty acyls	Fatty acid esters	NA	Early onset [15] preeclampsia, late onset preeclampsia
Lauroylcarnitine	Fatty acyls	Fatty acid esters	Lipid transport, metabolism, and peroxidation; fatty acid metabolism; cell signalling	Early onset [15] preeclampsia
Hexadecenoylcarnitine	Fatty acyls	Fatty acid esters	Lipid transport, metabolism, and peroxidation; fatty acid metabolism; cell signalling	Early onset [15] preeclampsia
Hexanoylcarnitine	Fatty acyls	Fatty acid esters	Lipid transport, metabolism, and peroxidation; fatty acid metabolism; cell signalling	Early onset [15] preeclampsia, late onset [15] preeclampsia
Hydroxyhexanoylcarnitine	Fatty acyls	Fatty acid esters	Lipid transport, metabolism, and peroxidation; fatty acid metabolism; cell signalling	Preeclampsia [5], early onset [5] preeclampsia
Isobutyrylcarnitine	Fatty acyls	Fatty acid esters	Lipid transport, metabolism, and peroxidation; fatty acid metabolism; cell signalling	Early onset [15] preeclampsia
Octanoylcarnitine	Fatty acyls	Fatty acid esters	Mitochondrial beta-oxidation of short chain saturated fatty acids	Early onset [15] preeclampsia, late onset [15] preeclampsia
Octenoylcarnitine	Fatty acyls	Fatty acid esters	Lipid transport, metabolism, and peroxidation; fatty acid metabolism; cell	Early onset [15] preeclampsia, late onset [15] preeclampsia

For peer review only

			signalling	
Oleylcarnitine	Fatty acyls	Fatty acid esters	NA	Late onset [15] preeclampsia
Palmitoylcarnitine	Fatty acyls	Fatty acid esters	Fatty acid metabolism	Late onset [15] preeclampsia
Isoprostane 8-epi-prostaglandin F2α	Fatty acyls	Eicosanoids	Arachidonic acid metabolism	Preeclampsia [16]
(+/-) 5-iPF2α-VI	Fatty acyls	Eicosanoids	Lipid peroxidation	Preeclampsia [14]
(+/-) 5-iPF2α-VI-(d11)	Fatty acyls	Eicosanoids	Lipid peroxidation	Preeclampsia [14]
15-epilipoxin-A4	Fatty acyls	Eicosanoids	Lipid transport, metabolism, and peroxidation; fatty acid metabolism; cell signalling	Preeclampsia [17]
Alfa-linolenic acid	Fatty acyls	Lineolic acids and derivatives	Lipid transport, metabolism, and peroxidation; fatty acid metabolism; cell signalling; alpha linolenic acid and linoleic acid metabolism.	Preeclampsia [14]
Stearidonic acid	Fatty acyls	Lineolic acids and derivatives	Lipid transport, metabolism, and peroxidation; fatty acid metabolism; cell signalling; alpha linolenic acid and linoleic acid metabolism.	Preeclampsia [14]
Di-(octadecadienoyl)-sn-glycerol	Glycerophospholipids	Glycerophosphates	Lipid transport, metabolism, and peroxidation; fatty acid metabolism; cell signalling	Late onset [12] preeclampsia
Heptadecanoyl-2-hydroxy-sn-glycero-3-phosphocholine	Glycerophospholipids	Glycerophosphocholines	Lipid transport, metabolism, and peroxidation; fatty acid metabolism; cell signalling; phospholipid and glycerophospholipid metabolism.	Preeclampsia [18]
Dilinoleoyl-glycerol	Glycerophospholipids	Glycerophosphocholines	NA	Preeclampsia [18]
LysoPE C20:0	Glycerophospholipids	Glycerophosphoethanolamines	Lipid peroxidation and metabolism;	Preeclampsia [19]

For peer review only

			glycerophospholipid metabolism; fatty acid metabolism cell signalling.	
Neomenthol-glucuronide	Prenol lipids	Terpene glycosides	Lipid peroxidation and metabolism; glycerophospholipid metabolism; fatty acid metabolism cell signalling.	Gestational hypertension [10]
Sphinganine 1-phosphate	Sphingolipids	Phosphosphingolipids	Sphingolipid metabolism, neuroactive ligand-receptor interaction	Late onset [12] preeclampsia
Sphingomyelin 16:0	Sphingolipids	Phosphosphingolipids	Lipid transport, metabolism, and peroxidation; fatty acid metabolism; cell signalling; apoptosis	Preeclampsia [20]
Sphingomyelin 18:0	Sphingolipids	Phosphosphingolipids	Phospholipid metabolism; lipid transport, metabolism, and peroxidation; fatty acid metabolism; cell signalling; apoptosis	Preeclampsia [20]
Sphingosine 1-phosphate	Sphingolipids	Phosphosphingolipids	Sphingolipid metabolism; lipid transport, metabolism, and peroxidation; fatty acid metabolism; cell signalling	Late onset [12] preeclampsia
Ceramide 14:0	Sphingolipids	Ceramides	NA	Preeclampsia [20]
Ceramide 24:0	Sphingolipids	Ceramides	NA	Preeclampsia [20]
Ceramide(d18:1/25:0)	Sphingolipids	Ceramides	NA	Preeclampsia [21]
Vitamin D	Steroids and steroids derivatives	Vitamin D and derivatives	Lipid transport, metabolism, and peroxidation; fatty acid metabolism; cell signalling	Preeclampsia [22], late onset [12] preeclampsia, gestational hypertension [23]
Bolasterone	Steroids and steroids derivatives	Androstan steroids	Lipid transport, metabolism, and peroxidation; fatty acid metabolism; cell signalling	Preeclampsia [10]
		Organoheterocyclic compounds		
Caffeine	Imidazopyrimidines	Purines and purine derivatives	Caffeine metabolism	Early onset [24] preeclampsia
Paraxanthine	Imidazopyrimidines	Purines and purine derivatives	Caffeine Metabolism	Early onset [24] preeclampsia

For peer review only - http://bmjopen.bmj.com/site/about/guidelines.xhtml

5-Hydroxytryptophan	Indoles and derivatives	Tryptamines and derivatives	Tryptophan metabolism	Late onset [12] preeclampsia
Serotonin	Indoles and derivatives	Tryptamines and derivatives	Tryptophan metabolism; nicotine action pathway	Late onset [4] preeclampsia
Gama-butyrolactone	Lactones	Gamma butyrolactones	NA	Late onset [12] preeclampsia
N-methyl-2-pyridone-5-carboxamide	Pyridines and derivatives	Pyridinecarboxylic acids and derivatives	Nicotinate and Nicotinamide Metabolism	Preeclampsia [3]
N-methylnicotinamide	Pyridines and derivatives	Pyridinecarboxylic acids and derivatives	NA	Preeclampsia [3]
Oxolan-3-one	Dihydrofurans	Furanones	NA	Late onset [12] preeclampsia
		Organic oxygen compounds		
4-deoxyerythronic acid	Organooxygen compounds	Carbohydrates and carbohydrate conjugates	NA	Preeclampsia [3]
Galactose	Organooxygen compounds	Carbohydrates and carbohydrate conjugates	Galactose metabolism, nucleotide sugars metabolismo, lactose degradation	Preeclampsia [3]
Glycerol	Organooxygen compounds	Carbohydrates and carbohydrate conjugates	Glycerolipid metabolism, galactose metabolism, glycerolipid metabolism	Early onset [11] preeclampsia
Lactose	Organooxygen compounds	Carbohydrates and carbohydrate conjugates	Galactose metabolism, Phosphotransferase system, carbohydrate digestion and absorption	Preeclampsia [3]
Sucrose	Organooxygen compounds	Carbohydrates and carbohydrate conjugates	Starch and sucrose metabolism, galactose metabolism	Preeclampsia [3]
Acetone	Organooxygen compounds	Carbonyl compounds	Ketone body metabolism	Early onset [11] preeclampsia, late onset [4] preeclampsia
		Organic nitrogen compounds		
Scyllo-inositol	Organonitrogen compounds	Alcohols and polyols	Inositol phosphate metabolism	Preeclampsia [3]
Propylene glycol	Organooxygen compounds	Alcohols and polyols	Pyruvate metabolism	Late onset [4] preeclampsia

For peer review only - http://bmjopen.bmj.com/site/about/guidelines.xhtml

Propane-1,3-diol	Organooxygen compounds	Alcohols and polyols	Glycerolipid metabolism	Preeclampsia [19]
Putrescine	Organonitrogen compounds	Amines	Methionine metabolism, spermidine and spermine biosynthesis	Late onset [4] preeclampsia
Trimethylamine	Organonitrogen compounds	Amines	Methane and carbon metabolisms.	Preeclampsia [2,10]
Carnitine	Organonitrogen compounds	Quaternary ammonium salts	Beta oxidation of very long chain and branched chain fatty acids, mitochondrial beta-oxidation of saturated fatty acids	Preeclampsia [3], late onset [4,13] preeclampsia
Linoleylcarnitine	Organonitrogen compounds	Quaternary ammonium salts	Lipid transport, metabolism, and peroxidation; fatty acid metabolism; cell signalling	Early onset [15] preeclampsia, late onset [15] preeclampsia
Stearoylcarnitine	Organonitrogen compounds	Quaternary ammonium salts	Mitochondrial beta-oxidation of long chain saturated fatty acids	Early onset [15] preeclampsia, late onset [15] preeclampsia
Tetradecenoylcarnitine	Organonitrogen compounds	Quaternary ammonium salts	Lipid transport, metabolism, and peroxidation; fatty acid metabolism; cell signalling	Early onset [15] preeclampsia, late onset [15] preeclampsia
		Benzenoids		
Bisphenol A	Benzene and substituted derivatives	Diphenylmethanes	Bisphenol degradation	Preeclampsia [25]
Hippurate	Benzene and substituted derivatives	Benzoic acids and derivatives	Phenylalanine metabolism	Preeclampsia [3]
4-OH-hippurate	Benzene and substituted derivatives	Benzoic acids and derivatives	NA	Preeclampsia [3]
Mono(2- ethyl-5-oxohexyl) phthalate	Benzene and substituted derivatives	Benzoic acids and derivatives	NA	Late onset [25] preeclampsia
Mono(2-ethyl-5-carboxypentyl) phthalate	Benzene and substituted derivatives	Benzoic acids and derivatives	NA	Late onset [25] preeclampsia

For peer review only

Mono(2-ethylhexyl) phthalate	Benzene and substituted derivatives	Benzoic acids and derivatives	NA	Preeclampsia [25]
Mono(3-carboxypropyl) phthalate	Benzene and substituted derivatives	Benzoic acids and derivatives	NA	Preeclampsia [25]
Mono-ethyl phthalate	Benzene and substituted derivatives	Benzoic acids and derivatives	NA	Preeclampsia [25], late onset [25] preeclampsia
Monoisobutyl phthalate	Benzene and substituted derivatives	Benzoic acids and derivatives	NA	Preeclampsia [25]
Mono-*n*-butyl phthalate	Benzene and substituted derivatives	Benzoic acids and derivatives	NA	Late onset [25] preeclampsia
Σ di(2-ethylhexyl) phthalate	Benzene and substituted derivatives	Benzoic acids and derivatives	NA	Preeclampsia [25], Late onset [25] preeclampsia
2,6-Di-tert-butyl-4-hydroxymethylphenol	Benzene and substituted derivatives	Phenylpropanes	NA	Gestational hypertension [10]
		Organohalogen compounds		
Perfluorohexane sulfonate	Alkyl halides	Alkyl fluorides	NA	Preeclampsia [26]
		Alkaloids and derivatives		
Trigonelline	Alkaloids derived from nicotinic acid	Pyridine alkaloids	Nicotinate and nicotinamide metabolism	Preeclampsia [3]
Pilocarpine	NA	NA	Biosynthesis of alkaloids derived from histidine and purine	Gestational hypertension [10]
		Phenylpropanoids and polyketides		
Cerasinone	Flavonoids	O-methylated flavonoids	NA	Preeclampsia [10]
		Organosulfur compounds		
Dimethyl sulfone	Sulfonyls	Sulfones	NA	Preeclampsia [2]

NA = not available

For peer review only

References

1 Gong S, Sovio U, Aye ILMH, *et al.* Placental polyamine metabolism differs by fetal sex, fetal growth restriction, and preeclampsia. *JCI Insight* 2018;**3**:1–15. doi:10.1172/jci.insight.120723

2 Kenny LC, Broadhurst DI, Hong W, *et al.* NMR Profiling in Early Pregnancy Reveals a Metabolomic Signature the Predicts the Subsequent Development Preeclampsia. *Reprod Sci* 2013;**20**:2641A. doi:10.1177/1933719113482088

3 Diaz SO, Barros AS, Goodfellow BJ, *et al.* Second trimester maternal urine for the diagnosis of trisomy 21 and prediction of poor pregnancy outcomes. *J Proteome Res* 2013;**12**:2946–57. doi:10.1021/pr4002355

4 Bahado-Singh R, Poon LC, Yilmaz A, *et al.* Integrated Proteomic and Metabolomic prediction of Term Preeclampsia. *Sci Rep* 2017;**7**:1–10. doi:10.1038/s41598-017-15882-9

5 Odibo AO, Goetzinger KR, Odibo L, *et al.* First-trimester prediction of preeclampsia using metabolomic biomarkers: a discovery phase study. *Prenat Diagn* 2011;**31**:990–4.

6 Rijvers CAH, Marzano S, Winkens B, *et al.* Early-pregnancy asymmetric dimethylarginine (ADMA) levels in women prone to develop recurrent hypertension. *Pregnancy Hypertens* 2013;**3**:118–23. doi:10.1016/j.preghy.2013.01.001

7 Khalil AA, Tsikas D, Akolekar R, *et al.* Asymmetric dimethylarginine, arginine and homoarginine at 11-13 weeks gestation and preeclampsia: A case-control study. *J Hum Hypertens* 2013;**27**:38–43. doi:10.1038/jhh.2011.109

8 Sovio U, McBride N, Wood AM, *et al.* 4-Hydroxyglutamate is a novel predictor of pre-eclampsia. *Int J Epidemiol* 2020;**49**:301–11. doi:10.1093/ije/dyz098

9 Kuc S, Koster MPH, Pennings JLA, *et al.* Metabolomics profiling for identification of novel potential markers in early prediction of preeclampsia. *PLoS One* 2014;**9**. doi:10.1371/journal.pone.0098540

10 Harville EW, Li Y-YY-Y, Pan K, *et al.* Untargeted analysis of first trimester serum to reveal biomarkers of pregnancy complications: a case-control discovery phase study. *Sci Rep* 2021;**11**:3468. doi:10.1038/s41598-021-82804-1

11 Bahado-Singh RO, Syngelaki A, Akolekar R, *et al.* Validation of metabolomic models for prediction of early-onset preeclampsia. *Am J Obstet Gynecol* 2015;**213**:530.e1-530.e10. doi:10.1016/j.ajog.2015.06.044

12 Kenny LC, Broadhurst DI, Dunn W, *et al.* Robust early pregnancy prediction of later preeclampsia using metabolomic biomarkers. *Hypertension* 2010;**56**:741–9. doi:10.1161/HYPERTENSIONAHA.110.157297

13 Bahado-Singh RO, Syngelaki A, Mandal R, *et al.* Metabolomic determination of pathogenesis of late-onset preeclampsia. *J Matern Neonatal Med* 2017;**30**:658–64. doi:10.1080/14767058.2016.1185411

14 Bilodeau JF, Qin Wei S, Larose J, *et al.* Plasma F2-isoprostane class VI isomers at 12-18 weeks of pregnancy are associated with later occurrence of preeclampsia. *Free Radic Biol Med* 2015;**85**:282–7. doi:10.1016/j.freeradbiomed.2015.05.012

For peer review only

15 Koster MPH, Vreeken RJ, Harms AC, *et al.* First-Trimester serum acylcarnitine levels to predict preeclampsia: A metabolomics approach. *Dis Markers* 2015;**2015**. doi:10.1155/2015/857108

16 Chappell L. A longitudinal study of biochemical variables in women at risk of preeclampsia. *Am J Obstet Gynecol* 2002;**187**:127–36. doi:10.1067/mob.2002.122969

17 Shanmugalingam R, Wang X, Motum P, *et al.* The 15-Epilipoxin-A4 Pathway with Prophylactic Aspirin in PreventingPreeclampsia: A Longitudinal Cohort Study. *J Clin Endocrinol Metab* 2020;**105**. doi:10.1210/clinem/dgaa642

18 Kenny LC, Thomas G, Poston L, *et al.* Prediction of preeclampsia risk in first time pregnant women: Metabolite biomarkers for a clinical test. *PLoS One* 2020;**15**:1–19. doi:10.1371/journal.pone.0244369

19 Lee SM, Kang Y, Lee EM, *et al.* Metabolomic biomarkers in midtrimester maternal plasma can accurately predict the development of preeclampsia. *Sci Rep* 2020;**10**:16142. doi:10.1038/s41598-020-72852-4

20 Dobierzewska A, Soman S, Illanes SE, *et al.* Plasma cross-gestational sphingolipidomic analyses reveal potential first trimester biomarkers of preeclampsia. *PLoS One* 2017;**12**:1–16. doi:10.1371/journal.pone.0175118

21 Huang Q, Hao S, You J, *et al.* Early-pregnancy prediction of risk for pre-eclampsia using maternal blood leptin/ceramide ratio: Discovery and confirmation. *BMJ Open* 2021;**11**. doi:10.1136/bmjopen-2021-050963

22 Woodham PC, Brittain JE, Baker AM, *et al.* Midgestation maternal serum 25-hydroxyvitamin D level and soluble fms-like tyrosine kinase 1/placental growth factor ratio as predictors of severe preeclampsia. *Hypertension* 2011;**58**:1120–5. doi:10.1161/HYPERTENSIONAHA.111.179069

23 Ates S, Sevket O, Ozcan P, *et al.* Vitamin D status in the first-trimester: Effects of vitamin D deficiency on pregnancy outcomes. *Afr Health Sci* 2016;**16**:36–43. doi:10.4314/ahs.v16i1.5

24 Eichelberger KY, Baker AM, Woodham PC, *et al.* Second-trimester maternal serum paraxanthine, CYP1A2 activity, and the risk of severe preeclampsia. *Obstet Gynecol* 2015;**126**:725–30. doi:10.1097/AOG.0000000000001041

25 Cantonwine DE, Meeker JD, Ferguson KK, *et al.* Urinary concentrations of bisphenol A and phthalate metabolites measured during pregnancy and risk of preeclampsia. *Environ Health Perspect* 2016;**124**:1651–5. doi:10.1289/EHP188

26 Rylander L, Lindh CH, Hansson SR, *et al.* Per- and Polyfluoroalkyl Substances in Early Pregnancy and Risk for Preeclampsia: A Case-Control Study in Southern Sweden. *Toxics* 2020;**8**. doi:10.3390/toxics8020043

For peer review only

Studies	Patient selection		Index test	
	Was a consecutive or random sample of patients enrolled?	Did the study avoid inappropriate exclusions?	Were the index test results interpreted without knowledge of the results of the reference standard?	If a threshold was used, was it pre-specified?
Chappell LC et al, 2002	Yes	No	Unclear	Yes
Powe et al, 2010	Yes	Yes	Unclear	Yes
Kenny LC et al, 2010	Yes	Yes	Yes	Yes
Odibo AO et al, 2011	Yes	No	Unclear	Yes
Woodham PC et al, 2011	Yes	Yes	Unclear	Yes
Rijvers CAH et al, 2013	Unclear	Unclear	Unclear	Yes
Khalil AA et al, 2013	Yes	Yes	Yes	Yes
Diaz SO et al, 2013	Yes	Unclear	Unclear	Yes
Kenny LC et al, 2013	Unclear	Unclear	Unclear	Yes
Kuc S et al, 2014	Yes	Yes	Unclear	Yes
Wetta LA et al, 2014	Yes	Yes	Unclear	Yes
Bahado-Singh RO et al, 2015	Yes	Yes	Unclear	Yes
Eichelberger KY et al, 2015	Yes	Yes	Yes	Yes
Koster MPH et al, 2015	Yes	Yes	Unclear	Yes
Bilodeau JF et al, 2015	Yes	Yes	Unclear	Yes
Ates S et al, 2016	Yes	Yes	Unclear	Yes
Cantonwine DE et al, 2016	Yes	Yes	Unclear	Yes
Kiely M et al, 2016	Yes	Yes	Yes	Yes
Bahado-Singh RO et al, 2017	Yes	Yes	Unclear	Yes
Bahado-Singh RO et al, 2017b	Yes	Yes	Unclear	Yes
Dobierzewska A et al, 2017	Yes	Yes	Unclear	Yes
Ye Y et al, 2017	Yes	No	Yes	Yes
Gong S et al, 2018	Yes	Yes	Unclear	Yes
Tamblyn JA et al, 2018	Yes	Yes	Yes	Yes
Sovio U et al, 2020	Yes	Yes	Unclear	Yes
Huo X et al, 2020	Yes	No	Unclear	Yes
Rylander L et al, 2020	Yes	Unclear	Unclear	Yes

For peer review only

Lee SM et al, 2020	Unclear	Unclear	Unclear	Yes
Kenny L et al, 2020	Yes	Yes	Yes	Yes
Shanmugalingam R et al, 2020	Yes	No	Unclear	Yes
Harville EW et al, 2021	Yes	Unclear	Unclear	Yes
Huang Q et al, 2021	Unclear	Yes	Unclear	Yes

For peer review only

Risk of bias				
Reference standard		Flow and timing		
Is the reference standard likely to correctly classify the target condition?	Were the reference standard results interpreted without knowledge of the results of the index test?	Was there an appropriate interval between index test and reference standard?	Did all patients receive the same reference standard?	Were all patients included in the analysis?
Yes	Yes	Yes	Yes	Yes
Yes	Unclear	Yes	Yes	Yes
Yes	Yes	Yes	Yes	Yes
Yes	Unclear	Yes	Yes	Yes
Yes	Unclear	Yes	Yes	Yes
Yes	Unclear	Yes	Yes	Yes
Yes	Unclear	Yes	Yes	Yes
Yes	Unclear	Unclear	Yes	Yes
Yes	Unclear	Yes	Yes	Yes
Yes	Unclear	Yes	Yes	Yes
Yes	Yes	Yes	Yes	Yes
Yes	Unclear	Yes	Yes	Yes
Yes	Unclear	Yes	Yes	Yes
Yes	Unclear	Yes	Yes	Yes
Yes	Unclear	Yes	Yes	Yes
Yes	Unclear	Yes	Yes	Yes
Yes	Unclear	Unclear	Yes	No
Yes	Yes	Yes	Yes	Yes
Yes	Unclear	Yes	Yes	Yes
Yes	Unclear	Yes	Yes	Yes
Yes	Unclear	Yes	Yes	Yes
Yes	Unclear	Yes	Yes	Yes
Yes	Unclear	Yes	Yes	Yes
Yes	Yes	Yes	Yes	Yes
Yes	Unclear	Yes	Yes	Yes
Yes	Unclear	Yes	Yes	Yes
Yes	Unclear	Yes	Yes	No

For peer review only

Yes	Unclear	Yes	Yes	Yes
Yes	Yes	Yes	Yes	Yes
Yes	Unclear	Yes	Yes	No
Unclear	Unclear	Yes	Yes	Yes
Yes	Unclear	Yes	Yes	Yes

For peer review only

Applicability concerns		
**Patient selection**	**Index test**	**Reference standard**
Are there concerns that the included patients do not match the review question?	Are there concerns that the index test, its conduct, or interpretation differ from the review question?	Are there concerns that the target condition as defined by the reference standard does not match the review
No	No	No
No	No	No
No	No	No
No	No	No
No	No	No
No	No	No
No	No	No
Yes	No	No
No	No	No
No	No	No
No	No	No
No	No	No
No	No	No
No	No	No
No	No	No
No	No	No
No	Yes	No
No	No	No
No	No	No
No	No	No
No	No	No
No	No	No
No	No	No
No	No	No
No	No	No
Yes	No	No
No	No	No

For peer review only

No	No	No
No	No	No
Yes	No	No
No	No	Yes
No	No	No

For peer review only

BMJ Open

Prediction of pregnancy-related hypertensive disorders using metabolomics: a systematic review

Journal:	*BMJ Open*
Manuscript ID	bmjopen-2021-054697.R2
Article Type:	Original research
Date Submitted by the Author:	21-Mar-2022
Complete List of Authors:	Mayrink, Jussara; State University of Campinas Faculty of Medical Sciences, Obstetrics and Gynecology<br>Leite, Debora; State University of Campinas Faculty of Medical Sciences, Obstetrics and Gynecology<br>Nobrega, Guilherme; State University of Campinas Faculty of Medical Sciences, Department of Obstetrics and Gynecology<br>Costa, Maria Laura; State University of Campinas Faculty of Medical Sciences, Obstetrics and Gynecology<br>Cecatti, Jose; State University of Campinas Faculty of Medical Sciences, Obstetrics and Gynecology
**Primary Subject Heading**:	Obstetrics and gynaecology
Secondary Subject Heading:	Diagnostics
Keywords:	Hypertension < CARDIOLOGY, Maternal medicine < OBSTETRICS, EPIDEMIOLOGY

SCHOLARONE™
Manuscripts

I, the Submitting Author has the right to grant and does grant on behalf of all authors of the Work (as defined in the below author licence), an exclusive licence and/or a non-exclusive licence for contributions from authors who are: i) UK Crown employees; ii) where BMJ has agreed a CC-BY licence shall apply, and/or iii) in accordance with the terms applicable for US Federal Government officers or employees acting as part of their official duties; on a worldwide, perpetual, irrevocable, royalty-free basis to BMJ Publishing Group Ltd ("BMJ") its licensees and where the relevant Journal is co-owned by BMJ to the co-owners of the Journal, to publish the Work in this journal and any other BMJ products and to exploit all rights, as set out in our licence.

The Submitting Author accepts and understands that any supply made under these terms is made by BMJ to the Submitting Author unless you are acting as an employee on behalf of your employer or a postgraduate student of an affiliated institution which is paying any applicable article publishing charge ("APC") for Open Access articles. Where the Submitting Author wishes to make the Work available on an Open Access basis (and intends to pay the relevant APC), the terms of reuse of such Open Access shall be governed by a Creative Commons licence – details of these licences and which Creative Commons licence will apply to this Work are set out in our licence referred to above.

Other than as permitted in any relevant BMJ Author's Self Archiving Policies, I confirm this Work has not been accepted for publication elsewhere, is not being considered for publication elsewhere and does not duplicate material already published. I confirm all authors consent to publication of this Work and authorise the granting of this licence.

For peer review only

SYSTEMATIC REVIEW

Prediction of pregnancy-related hypertensive disorders using metabolomics: a systematic review

Jussara Mayrink, MD, PhD [1,2] (0000-0001-5957-4365; jussaramayrink@gmail.com)

Debora Farias B. Leite, MD, PhD [1,3] (0000-0001-8839-3934; debora.leite@ufpe.br)

Guilherme Moraes Nobrega, BSc [1] (0000-0001-9406-4256; guigo.nobrega@hotmail.com)

Maria Laura Costa, MD, PhD [1] **(**0000-0001-8280-3234; mlaura@unicamp.br)

José Guilherme Cecatti, MD, PhD [1] (0000-0003-1285-8445; cecatti@unicamp.br)

[1]Department of Obstetrics and Gynecology, University of Campinas, School of Medicine, Brazil

[2]Federal University of Minas Gerais, Belo Horizonte, Minas Gerais, Brazil.

[3]Department of Gynecology and Obstetrics, Federal University of Pernambuco, Recife, Pernambuco, Brazil.

Correspondence:

José Guilherme Cecatti

Rua Alexander Fleming, 101 Cidade Universitaria Zeferino Vaz.

13083-889. Campinas, Sao Paulo, Brazil.

Telephone: +55 19-35219482

Email: cecatti@unicamp.br

Word Count:

Abstract: 300 words

Main text: 5,144 words

For peer review only - http://bmjopen.bmj.com/site/about/guidelines.xhtml

Abstract

Objective: To determine the accuracy of metabolomics in predicting hypertensive disorders in pregnancy.

Design: Systematic Review of observational studies.

Data sources and study eligibility criteria: An electronic literature search was performed in June, 2019 and February, 2022. Two researchers independently selected studies published between 1998 and 2022 on metabolomics techniques applied to predict the condition; subsequently, they extracted data and performed quality assessment. Discrepancies were dealt with a third reviewer. The primary outcome was preeclampsia. Cohort or case-control studies were eligible when maternal samples were taken before the diagnosis of the hypertensive disorder.

Study appraisal and synthesis methods: Data on study design, maternal characteristics, how hypertension was diagnosed, metabolomics details and metabolites, and accuracy were independently extracted by two authors.

Results: Among 4,613 initially identified studies on metabolomics, 68 were read in full text and 32 articles were included. Studies were excluded due to duplicated data, study design or lack of identification of metabolites. Metabolomics was applied mainly in the 2nd trimester; the liquid-chromatography coupled to mass spectrometry was the most common technique. Among the 122 different metabolites found, there were 23 amino acids and 21 fatty acids. Most of metabolites were involved with ammonia recycling; amino acid metabolism; arachidonic acid metabolism; lipid transport, metabolism, and peroxidation; fatty acid metabolism; cell signalling; galactose metabolism; nucleotide sugars metabolism; lactose degradation; and glycerolipid metabolism. Only citrate was a common metabolite for prediction of early- and late-onset preeclampsia. Vitamin D was the only metabolite in common for preeclampsia and gestational hypertension prediction. Meta-analysis was not performed due to lack of appropriate standardized data.

Conclusions and Implications: Metabolite signature may contribute to further insights into the pathogenesis of preeclampsia and support screening tests. Nevertheless, it is mandatory to validate the method in larger studies with a heterogeneous population to ascertain its use in clinical practice.

PROSPERO registration: CRD42018097409.

For peer review only - http://bmjopen.bmj.com/site/about/guidelines.xhtml

Keywords: preeclampsia, systematic review, hypertension, metabolomics, metabolome, prediction.

Strengths and limitations of this study

- This Systematic Review present possible biomarkers of preeclampsia that could provide a basis for further research in this area.
- There was great heterogeneity among included studies due to complexity of metabolomics procedures, which are influenced by sample collection, storage and preparation, analytical platform applied, and statistical tests performed.
- Our findings must be treated with caution due to the lack of standardization of preeclampsia definition (early and late-onset preeclampsia) and timing of sample collection in pregnancy.

Original protocol: Mayrink J, Leite DFB, Costa ML, *et al*. Metabolomics for prediction of hypertension in pregnancy: a systematic review and meta-analysis protocol. *BMJ Open* 2020;10:e040652. doi:10.1136/bmjopen-2020-040652.

For peer review only

INTRODUCTION

Hypertensive disorders have been reported as associated with adverse outcomes in pregnancy since years before Christ. They are a heterogeneous group of conditions that include chronic hypertension, preeclampsia, gestational hypertension and preeclampsia superimposed on chronic hypertension.[1]

Chronic hypertension is the condition of systolic blood pressure ≥140 mmHg and/or diastolic blood pressure ≥ 90 mmHg before twenty weeks of gestation or even before pregnancy. [2] Gestational hypertension is the isolated elevation of pressure after twenty weeks of gestation. It is less damaging than preeclampsia but around 30% of these cases progress to pre-eclampsia.[3] The most prevalent form – pre-eclampsia - is a serious and potentially-life threatening condition to the mother and the baby, [2] and its high prevalence rate persists over the years, ranging from 3 to 10% of all pregnancies, according to the country studied. Unfortunately, treatment has been the same over decades and relies on the management of clinical features, such as new onset of hypertension and end-organ dysfunction, that characterizes preeclampsia. Outcomes mostly depend upon accurate diagnosis and decision on timing of childbirth since there are few predictive tools available. [3] As a result, one of the most urgent priorities is to identify those women at high-risk for the disease, who would be candidates for prophylactic and increased surveillance measures. The increased understanding on pathophysiology would also shed light into possible new therapeutic approaches.

Several biomarkers have been studied as predictive tools for preeclampsia, e.g. soluble fms-like tyrosine kinase-1, soluble endoglin, markers of apoptosis and inflammation, placental protein 13, C-reactive protein, and markers of placental hypoxia and distress. [4] However, none are sufficiently sensitive or specific to predict preeclampsia in advance.

Recently, novel technologies, such as metabolomics, have been applied to predict preeclampsia. Metabolomics is known as the newest member of the “omics” family. Metabolome - a collection of metabolites – is defined and used in research to pursue

For peer review only - http://bmjopen.bmj.com/site/about/guidelines.xhtml

the phenotypic signature of a disease of interest. Metabolites are low-molecular-weight chemicals (<1500 Da) resulting from changes in gene, protein expression [5] and environmental interferences. Metabolomics methods include hydrogen nuclear magnetic resonance spectroscopy (^{1}H-NMR) and gas- and liquid- chromatography-mass spectrometry (GC- and LC-MS, respectively). [5,6] Metabolomics has been applied to improve disease biomarkers and understand the pathogenesis of many conditions, such as cancer and diabetes. [7,8] One could argue its value in evaluating preeclampsia, which has a complex aetiology and is considered a multifactorial disease. Metabolomics, however, a high throughput technique, seems perfectly adequate, since it can simultaneously encompass a wide range of metabolic pathways. The objective of this systematic review was to determine the accuracy of metabolomics in predicting pregnancy-induced hypertensive disorders.

METHODS

This systematic review was conducted based on our previously published protocol [9] (CRD42018097409) and is reported in accordance with the Preferred Reported Items for Systematic Reviews and Meta-Analysis (PRISMA) statement; [10] a PRISMA checklist is provided as a Supplementary Material 1. Our research question was: "what is the performance of metabolomics for predicting gestational hypertensive disorders?"

Population and Interventions

Our domain of interest was any form of hypertensive disorder developed during pregnancy, in relation to either single or multiple gestations. Metabolomics was the intervention studied. Papers eligible for inclusion in our review should mention metabolites potentially differentiating hypertensive from normotensive pregnant women. Maternal samples should have been drawn during pregnancy and before diagnosis of pregnancy-induced hypertensive disorder. We excluded studies in which blood samples were collected after the diagnosis of hypertension was established, or when preeclampsia and gestational hypertension were analysed as the same outcome.

For peer review only - http://bmjopen.bmj.com/site/about/guidelines.xhtml

Comparison and Outcomes

We included studies in which women with any form of pregnancy-induced hypertension were compared to pregnant women without hypertension. Preeclampsia was the primary outcome, diagnosed by criteria defined by the authors. [11–15]

Secondary outcomes were: a) early-onset preeclampsia, characterized by delivery <34 weeks due to preeclampsia; [3] b) late-onset preeclampsia, requiring delivery ≥34 weeks; [3] c) gestational hypertension, when hypertension is the only clinical finding after 20 weeks gestation; d) preeclampsia superimposed on chronic hypertension; e) white coat hypertension, i.e., normal blood pressure recorded in a 24h ambulatory monitoring; f) masked hypertension, characterized as normal blood pressure in the office or clinic and altered under other circumstances; and g) transient gestational hypertension, higher blood pressure at levels during a certain period that is later normalized after repeated blood pressure readings. [11,16]

Search strategy

Two independent researchers (JM, DFBL) performed an electronic database search in PubMed, EMBASE, Scopus, Web of Knowledge, Latin American and Caribbean Health Sciences Literature (LILACS), Scientific Electronic Library Online (SciELO), Health Technology Assessment (HTA), and Database of Abstracts of Reviews of Effects (DARE), including studies published from 1998 to 2022. The full search strategies are provided as Supplementary Material 2. We have applied no filters, except for SCOPUS (we have used the Title, Abstract and Keyword filter). The grey literature (conference abstracts) and the reference list of included studies were searched for additional articles for inclusion.

There were no language restrictions; only cohort and case control studies were included. Letters to the editor, editorials, comments, expert opinions, any type of review, experimental animal studies, case reports, intervention trials, and cross-sectional studies were all excluded. We intend to show the capacity of metabolomics to predict hypertensive disorders without interference of any intervention. Thus, it is absolutely necessary to guarantee the temporal relation between timing of sample

For peer review only

(before) and outcome (after). This is the reason for including just cohort and case control studies.

Data extraction and quality assessment

Trained reviewers (JM, DFBL) proceeded studies' screening (using EndNote® software), data extraction and synthesis, and risk of bias evaluation (Quality Assessment of Diagnostic Accuracy Studies (QUADAS-2) [17]). Disagreements were solved by a third reviewer (MLC).

Summary of data and meta-analysis

For all included studies, we have extracted data on research characteristics and metabolomics details. If the study comprised training and validation datasets, we have extracted metabolomics data from the validation group of women. When the study has not definitively identified a given metabolite, we have extracted all possible metabolites. We have extracted data of metabolites combined with other biomarkers, when available. We have used the Human Metabolome Database [18], the Kyoto Encyclopaedia of Genes and Genomes [19] and the Lipid Maps [20] for matching each metabolite chemical class and subclass, and metabolic pathways and processes.

When available, data on accuracy measures on metabolomics performance in predicting hypertensive disorders of pregnancy were extracted. We planned to perform the estimation of likelihood ratios and hierarchical summary receiver operator characteristic curve, [21] and the assessment of heterogeneity and publication bias. [22]

Patient and public involvement

It was not appropriate or possible to involve patients or the public in the design, or conduct, or reporting, or dissemination plans of our research

For peer review only

For peer review only - http://bmjopen.bmj.com/site/about/guidelines.xhtml

RESULTS

Main findings

The literature search for this systematic review was performed in June 2019 with a further re-run in February 2022. **Figure 1** summarizes the flow chart of studies' selection and final inclusion. We excluded studies (Supplementary Material 3) due to study design (25 studies) or lack of identification of metabolites (05 studies). Regarding duplicated data, only the most recent and complete study was included (06 studies). .

Overall, 32 studies were selected: 25 case control studies [23–47] and seven cohort studies [48–54] (**Table 1**). Studies from the same research project but evaluating different metabolites [43,46] or populations [24,28,29,33,36,38,39] were included. Most studies were developed in Europe [23,25,28,29,31,33,36,38,39,42,43,46,48–52] and North America; [32,34,35,40,41,44,45,47,54] there was only one research from Latin America, [26] two from Oceania [24,53] and another three from Asia. [27,30,37] Twelve studies have sampled maternal specimens in the 1st trimester, [31,32,34,35,42,43,46,48,50–53] 15 studies have sampled in the 2nd trimester, [23,24,27,29,30,33,36–39,41,44,45,49,54] four studies have sampled at least two times [25,26,40,47] in pregnancy, and one study included women in late 3rd trimester [28] (**Figure 2**). Four studies [24,33,38,40] have performed training and validation with different populations.

Studies have defined preeclampsia as hypertension developed in pregnancy associated with proteinuria [11,13–15] or end-organ dysfunction. [12] Although not consistently, chronic hypertension or other illnesses, [24,27,29,33,36–39,41,45,55] and multiple pregnancies [25,27,30,32,33,36,37,39,41,42,45,52] were reasons for participants exclusion. When chronic hypertensive women were included, there were women taking aspirin as prophylaxis. [23,53] Then, there are no specific data on superimposed preeclampsia or multiple pregnancies. Similarly, there are no studies on white coat hypertension, masked hypertension, or transient gestational hypertension. Only three studies have brought data on gestational hypertension. [32,37,51] Small for gestational age (SGA) infants were fully excluded [31] or included only in preeclampsia group,

For peer review only - http://bmjopen.bmj.com/site/about/guidelines.xhtml

either because they were excluded from control groups, [23,34] or because this variable was not used as for matching cases and controls. [24,29,36]

In **Table 2**, characteristics of the included studies regarding metabolomics techniques and accuracy measures are shown according to the primary and secondary outcomes. The majority of studies did not present data according to gestational age of delivery. The most common technique was LC-MS, [24,26,27,29,31,33–38,40,41,43–48,51,53,55] followed by H-NMR [25,39,49,50,52] and GC-MS. [23,42] Bahado-Singh et al. [25] (H-NMR followed by LC-MS), Bilodeau et al. [54] (HPLC-MS/MS and GC-FID), Lee et al. [30] (GC-MS and LC-MS), and Harville et al. [32] (LC-MS and NMR) have used two complementary techniques. Most studies analysed blood samples, [23,24,26–28,30–46,48,50–54] two studies analysed urine [47,49] and one study analysed both, urine and blood [29] (**Figure 2**). There were no studies carrying out analyses on maternal hair or amniotic fluid. All studies that used H-NMR [25,32,39,49,50,52] and four that applied LC-MS [24,28,32,38] were untargeted evaluations. Eleven studies reported lipidomic analysis and/or amino acid analysis. [23,26,30,35,40,42,43,46,48,53,54] Six studies described vitamin D analysis, all of them using LC-MS.[29,34,36,41,44,51] Five studies reported environmental exposures, focusing on caffeine, [45] bisphenol A, [27] phthalates [47] and per-and polyfluoroalkyl substances (PFAS). [31,37] Two studies have looked for differences in preeclampsia prediction according to foetal sex. [28,47] There were five metabolites in common between preeclampsia and SGA prediction (sphinganine 1-phosphate, sphingosine 1-phosphate, tyrosine, vitamin D, N1, N12-diacetylspermine). [56]

For preeclampsia requiring delivery at any gestational age, the lowest area under the curve (AUC) was for isoprostane 8-epi-prostaglandin F2α (0.55), [23] and the highest (AUC 0.95) was for a model composed by cerasinone, bolasterone, and two unidentified metabolites, presented as retention time and mass (6.30_477.7721m/z, 5.86_313.1070m/z).[32] For early onset preeclampsia, the AUC of untargeted metabolomics in 1st trimester maternal blood varied from 0.673 (UPLC-MS/MS) [38] to 0.835 (H-NMR). [50] For late onset preeclampsia, the AUC of untargeted metabolomics

For peer review only - http://bmjopen.bmj.com/site/about/guidelines.xhtml

in 1st trimester maternal blood was 0.629 (H-NMR); [52] in 2nd trimester, 0.92 (UPLC-MS); [24] and in 3rd trimester, 0.761 (H-NMR and LC-MS/MS). [25]

When analysed together with non-metabolomics biomarkers (**Table 3**), the best performance was achieved by taurine combined with maternal prior risk and mean arterial blood pressure (MAP) (AUC 0.93 in 1st trimester for early onset preeclampsia); [43] followed by 3-hydroxyisovalerate, arginine, glycerol combined with uterine arteries pulsatility index (UtPI) (AUC 0.917 in 1st trimester for early onset preeclampsia) [50]; and leptin/ceramide(d18:1/25:0) ratio in in 1st and 2nd trimester (AUC 0.876 and 0.892, respectively) for preeclampsia at any gestational age. [40]

All predictive metabolites of hypertensive disorders of pregnancy according to chemical class and subclass, and metabolic pathways possibly involved are presented as Supplementary Material 4. Ten metabolites could not be found in the metabolome databases (U4, U10, U14, U35, [49] hexadecenoyleicosatetraenoyl-Sn-glycerol; [24] SM C28:1, SM C30:1, LysoPC C19:0 [30], dibenzylamine [32], perfluoroheptanoic acid [37]). There were 121 different metabolites, from ten different super classes. The majority of metabolites corresponded to amino acids, peptides, and analogues (23 compounds); fatty acid esters (11 compounds); fatty acids and conjugates (10 compounds); and carbohydrates and carbohydrate conjugates (5 compounds). They are mainly involved with ammonia recycling; amino acid metabolism; arachidonic acid metabolism; lipid transport, metabolism, and peroxidation; fatty acid metabolism; cell signalling; galactose metabolism; nucleotide sugars metabolism; lactose degradation; and glycerolipid metabolism. There were 10 benzoic acids and derivatives, but only hippurate has a known pathway. Citrate was found in common for prediction of preeclampsia in any gestational age, [49] early [50] and late-onset [25] disease. Octenoylcarnitine, [46] octanoylcarnitine, [46] acetone, [25,50] linoleylcarnitine, [46] stearoylcarnitine, [46] tetradecenoylcarnitine [46] were predictive for both early and late preeclampsia. Vitamin D was the only metabolite involved simultaneously with preeclampsia, [41] late onset [24] preeclampsia, and gestational hypertension. [51]

For peer review only - http://bmjopen.bmj.com/site/about/guidelines.xhtml

Summary of included studies

The untargeted studies provided a great number of predictive metabolites. Kenny et al. showed a decrease of 13 metabolites (NMR) [39] and an increment of another 40 metabolites (LC-MS) [24] in blood analysis between 14-16 weeks of nulliparous pregnant women exclusively. In a validation phase with a list of 14 different metabolites, the odds ratio of developing preeclampsia, at any gestational age, was 23 (95% CI: 7-73), with AUC 0.92. [24] These same metabolites were validated in a different population, [33] and the dilinoleoyl-glycerol has achieved an AUC 0.70 (0.59-0.82).

Bahado-Singh et al. applied metabolomics to predict early- and late-onset cases. [25,50,52] Using maternal 1st trimester blood (11-13w), those authors reinforced the theory of the multiple phenotypes for preeclampsia. [57,58] 2-hydroxybutyrate was identified as predictor of early-onset preeclampsia, [50] whereas carnitine, pyruvate and acetone resulted in an AUC of 0.629 for late-onset disease. This latter AUC increased to 0.734 by combining metabolites and uterine Doppler velocimetry and maternal weight. [52] Finally, in a two-step evaluation in the 1st and 3rd trimester to predict term preeclampsia (≥37w), a combination of metabolomics and proteomics yielded an AUC of 0.817. [25]

Through an untargeted urinary metabolome analysis, Diaz et al. [49] showed decreased levels of acetate, formate, fumarate (all involved with the Krebs cycle), succinate, and isoleucine; and increased levels of 4-deoxyerythronic acid, a degradation product of 3-hydroxybutyrate [49] between 14-26w. Finally, still working with an untargeted approach, Sovio et al. demonstrated an increase of 4-hydroxyglutamate serum levels among women who developed early-onset preeclampsia, measured at 12w. [38] Its prediction performance was improved by addition of pregnancy-associated plasma protein A (PAPP-A) and PlGF. [38]

Regarding lipidomic or amino-acid studies, Odibo et al. adjusted four metabolites (hydroxyhexanoylcarnitine, phenylalanine, glutamate, alanine) for maternal variables (maternal body mass index, ethnicity, and diabetes) and the AUCs were similar with

For peer review only - http://bmjopen.bmj.com/site/about/guidelines.xhtml

the four-metabolite model (0.82) or when only the three amino acids were used for predicting preeclampsia (0.81). Additionally, the four metabolites have demonstrated equal AUCs for predicting preeclampsia at any gestational age (0.82) or in cases of early onset disease (0.84). [35]In the 1st trimester, Shanmugalingam et al. [53] showed lower levels of 15-epilipoxin-A4, which is possibly involved with the aspirin mechanism of preeclampsia prophylaxis. Chappell et al. showed an increment in 8-epi-prostaglandin F2α from 20 to 24 weeks in the preeclampsia group. [23] Recently, Bilodeau et al. reinforced that F2-isoprostanes isomers from class VI were increased in the preeclampsia group. [54] The prostaglandins' metabolites are markers of lipid peroxidation, which pointed to an oxidative stress status, but presented modest accuracy. [23,54]

Rijvers et al. [48] and Khalil et al. [42] studied the metabolism of asymmetric dimethylarginine (ADMA), which is considered an important inhibitor of nitric oxide action. Rijvers et al. found an increase of L-arginine/ADMA ratio at 12w in the preeclampsia group, [48] and a consistent decrease in L-arginine between 16w and 20w in the preeclampsia compared to the gestational hypertension group. This can be clinically relevant since L-arginine supplementation is possible. [59] Khalil et al. found lower levels of L-arginine and L-homoarginine at 11-13w in the early-onset preeclampsia, and unchanged levels in the late-onset group, even after the excluding chronic hypertensive women. [42]

Koster et al. [46] showed that stearoylcarnitine was a common biomarker for both early-onset and late-onset preeclampsia. In a model composed of stearoylcarnitine, prior risk and MAP, the detection rate improved by 45% and 21%, for early and late-onset cases of preeclampsia, respectively. Dobierzewska et al. [26] have studied sphingolipids that have been related to trophoblast differentiation and invasion. In the ceramide profile, the Cer 14:0 was decreased in both 1st and 3rd trimesters of preeclampsia group, while Cer 16:0 was significantly increased in cross-gestational preeclampsia plasma samples between 1st and 2nd trimesters. Plasma levels of dihydro-sphingosine-1 decreased from the 1st to the 2nd trimester among the preeclampsia group, and SM 18:0 increased through gestation of these participants. [26] Still

For peer review only - http://bmjopen.bmj.com/site/about/guidelines.xhtml

studying ceramides, Huang et al [40] have found decreasing levels of Ceramide(d18:1/25:0) from 5-29 weeks, which increased the Leptin/ Ceramide(d18:1/25:0) ratio. Lee SM et al. proposed a model constituted by SM C28:1, SM C30:1, LysoPC C19:0, LysoPE C20:0 and propane-1,3-diol, achieving an AUC of 0.868 for identifying preeclampsia. [30]

Gong et al. [28] analysed serum samples in different moments of pregnancy, but it was at 36w when N1, N12 diacetylspermine were higher in women who later present preeclampsia, especially in the case of a female fetus. [28] This was the only study in common with our previous systematic review about SGA infant's prediction using metabolomics. [56]

Regarding targeted vitamin D analysis, all studies have applied LC-MS. [29,34,36,41,44,51] In general, they found lower serum vitamin D concentrations in women who later developed preeclampsia, both in blood [36] and urine, [29] suggesting that dysregulation of vitamin D metabolism occurs early in pregnancy. Indeed, Woodham et al. have pointed reduced odds of 38% of developing severe preeclampsia for a 10nmol/L increase in 25(OH)D level between 15-20w. [41] However, results did not reach statistical significance when a threshold was applied. In nested case-controls [34,44] or cohort [51] studies there were no differences between vitamin D status among preeclampsia group and controls. In the model proposed by Woodham et al. combining 25OHD, VEGF and sFLT-1/PlGF ratio, the AUC was 0.851, a higher value than either marker alone. [41] Vitamin D3 derivatives were found in the untargeted analysis of Kenny et al., [24] but upregulated in preeclampsia cases. [41]

Two studies have evaluated maternal exposure to bisphenol A and its metabolites. [27,47] The di(2-ethylhexyl) phthalate was increased in all trimesters. [47] Its hazard ratio for development of preeclampsia varied from 1.79 (95% CI 1.30-1.52), with 4-16 weeks, to 2.92 (95% CI 1.61-5.28) with 33-38w. The mono(2-ethyl-5-oxohexyl) phthalate, the mono(2-ethyl-5-carboxypentyl) phthalate and the Σdi(2-ethylhexyl) phthalate were associated to increased hazard ratios for preeclampsia when evaluated in urine [47] from both the 1st and the 2nd half of pregnancy. When the findings were analysed according to fetal sex, exposure to bisphenol and di(2-ethylhexyl) phthalate

For peer review only - http://bmjopen.bmj.com/site/about/guidelines.xhtml

was only associated to preeclampsia in pregnancies of female fetuses.[47] In maternal serum, [27] free bisphenol was higher in preeclamptic women compared to normotensive controls, even when superimposed preeclampsia cases were excluded. The cut-off of >4.4 µg/L was associated with increased odds of 16.46 (95% CI 5.42-49.95) of developing preeclampsia (adjusted for maternal age, parity, and body mass index). [27]

In another analysis of environmental factors, Eichelberger et al. found no differences in paraxanthine or caffeine levels, but the paraxanthine/caffeine ratio was lower in preeclampsia (0.23 versus; normotensive women, 0.37, *p* 0.02). [45] This represented a decreased risk of developing preeclampsia with increasing paraxanthine/caffeine ratio (odds ratio 0.53, 95%CI 0.31-0.90). Finally, regarding the perfluoroalkyl substances (PFASs), Rylander et al. [31] and Huo et al. [37] have found no differences between preeclamptic women and normotensive controls in blood samples from the 1st or 2nd trimester, respectively. However, levels of perfluorohexane sulfonate between 0.53-0.78ng/mL were associated to higher odds of preeclampsia (aOR 1.67, 95% CI1.02-2.74); [31] and of perfluoroheptanoic acid, to higher odds of gestational hypertension (aOR 1.38; 95%CI 1.01-1.87).

In the last study to evaluate gestational hypertension, Harville et al. [32] identified higher concentrations of 2,6-Di-tert-butyl-4-hydroxymethylphenol in blood samples between 6-14w. This metabolite is derived from 2,6-Di-tert-butyl-4-methylphenol, a synthetic phenolic antioxidant used widely in foods, polymers, and cosmetics to slow oxidation. It has been linked to induce cellular DNA damage.

Quality assessment and risk of bias

The synthesized data for all included studies is shown on **Figure 3**. Regarding the risk of bias, the majority of studies were rated low for ‘Patient Selection’ and ‘Flow and Timing’ domains. On the contrary, most studies were classified as unknown risk of bias in ‘Index Test’ and ‘Reference Standard’ domains due to missing information whether diagnosis and metabolomics analysis were interpreted independently of each other.

For peer review only - http://bmjopen.bmj.com/site/about/guidelines.xhtml

Virtually, all studies were ranked as low with regard to applicability, with minor exceptions. [32,47,49,53,60] (Supplementary Material 5).

Meta-analysis

We found that a third of included studies were designed around a prediction aim [23–25,27,35,38,41,50,52] and we extracted accuracy measures when possible (**Tables 2 and 3**). The other studies only presented statistical differences between groups. Unfortunately, there were discrepancies regarding type of maternal sample or gestational age at sampling, and metabolomics technique. Then, it was not possible to perform a meta-analysis.

DISCUSSION

Hypertensive disorders constitute one of the leading causes of maternal morbidity and mortality worldwide. In this systematic review, we have found that amino acids and fatty acids were the most common chemical subclasses associated with pre-eclampsia. Similarly, the main pathways involved in preeclampsia were lipid metabolism, mitochondrial beta-oxidation of short chain saturated fatty acids, and cell signalling (**Figure 4**). Our findings can improve disease understanding and contribute to future studies as a guide of pathways to be explored with the purpose to better treat the disease.

Amino acids and related compounds

Amino acids, peptides, and analogues were the most common chemical subclass. Taurine, glycine and glycylglycine were decreased in women who developed pre-eclampsia. [39,43] The function of taurine has been associated with cytoprotection, involving the placentation process and the spiral artery remodelling. Nevertheless, a reduction in taurine activity may be associated with impaired placentation, which is correlated with the pathophysiology of pre-eclampsia. [43] Glycylglycine is strongly associated with homocysteine levels and participates in the metabolic pathway that antagonizes homocysteine: lower glycine levels promote higher homocysteine levels. Homocysteine is linked to cardiovascular risk and endothelial dysfunction.

For peer review only - http://bmjopen.bmj.com/site/about/guidelines.xhtml

Indeed, a reduction in arginine and homoarginine was observed among pre-eclampsia patients. [42] These are substrates for oxide nitric synthesis, a potent vasodilator with a major function in endothelial cells. ADMA is an inhibitor of the enzyme responsible for NO production; there is evidence that elevated ADMA levels are associated with endothelial dysfunction, and consequently, pre-eclampsia. [48,61] NO is an important regulator of trophoblast implantation, differentiation, motility, invasion and apoptosis. [42] Decreased levels of arginine and homoarginine in blood samples of pre-eclampsia patients reinforce the involvement of NO molecule in the aetiology of the disorder. [42,50]

Carnitine is a non-essential amino acid strongly associated with lipid metabolism. Its function is to bind to fatty acid, form acyl-carnitine, and shuttle it to mitochondria, to integrate the mitochondrial oxidative metabolism. [52] The increment of carnitine might be associated to the oxidative stress observed in pre-eclampsia. [24,25,35,46,49,52]

Lipids

Maternal serum lipids are markedly elevated in healthy pregnancies, probably a hormone- induced increase. [62] Preeclamptic women may have even higher levels. [63] The lipidome – the group of lipid metabolites - contains key mediators of vascular tone (sphingosine), inflammation (prostaglandins), and insulin sensitivity (free fatty acids). [24] Lipids are involved in the aetiology of pre-eclampsia either indirectly, as a substrate, or directly, as a disease mediator. [63] Lipid peroxides may cause endothelial cell activation and reinforce the action of diabetes mellitus and essential hypertension as important risk factors to pre-eclampsia. [23]

In a healthy pregnancy, the pro-angiogenic biochemical scenario, sphingosine-1-phosphate and its receptors are up-regulated. Ceramide levels are also increased, reflecting the trophoblastic apoptosis process, observed because of normal syncytial fusion of villous trophoblasts. [63] On the other hand, in pre-eclampsia, low levels of sphingosine-1-phosphate (angiogenic) and high levels of ceramides (pro-apoptotic) were found in serum analyses of 3rd trimester pre-eclampsia cases. [26] The increase of

For peer review only - http://bmjopen.bmj.com/site/about/guidelines.xhtml

sphingomyelin SM:18 might serve not only as atherogenic marker of pre-eclampsia development and progression, but also the marker of cardiovascular complications developed later in women who had previous preeclamptic pregnancies. Recent studies have demonstrated that serum elevation of sphingomyelin SM:18 positively correlates with parameters of insulin resistance and liver function in obese adults [64] and correlates with markers of NFkB activation and, thus, markers of intracellular inflammation. [64] The ceramides might be biomarkers for cardiovascular disease in later life, which points attention to its role in hypertension in pregnancy. [65] As ceramide and their synthesizing enzymes (e.g. ceramide synthases) have been shown to be significantly up-regulated in the mouse uterus during early gestation, [44,66] the role of ceramides in the normal human pregnancy and its relationship with pre-eclampsia etiopathogenesis needs to be further explored.

Pre-eclampsia has also been correlated with high amounts of circulating reactive oxygen species. Among the consequences of this, high levels of oxidized cholesterol and acylcarnitine can be attributable to oxidation impairment of phosphocoline and fatty acid, respectively. [66]

Glycerophosphocholines resulted from cell lysis, possibly correlated with apoptosis described in pre-eclampsia. For the propanoate pathway, late-onset pre-eclampsia showed higher concentrations of L- valine, 2-hydroxybutyric acid, lactic acid, acetone and propan-2-ol. [52] Decreased glycerol level has been regarded as an important biomarker of late-onset pre-eclampsia and this may be due to its conversion to triglycerides. [57,63]

Vitamin D

The included studies on vitamin D showed contradictory results. [29,34,36,41,44,51] The heterogeneity of results may be attributable to the measurement of only serum 25(OH)D3 vitamin D, which is considered simplistic. [29] Interestingly, all included studies have considered low risk women and have presented data irrespective of gestational age of pre-eclampsia diagnosis. The active metabolite - 1,25-dihydroxyvitamin D - is thought to increase vascular endothelial growth factor

For peer review only - http://bmjopen.bmj.com/site/about/guidelines.xhtml

transcription, [67] which is correlated to placenta functioning and adaptation. Besides, vitamin D deficiency may be associated with an increment of inflammatory cytokine levels, such as TNF-alpha, and to a tendency towards T-cell activity. [68] Thus, its deficiency could potentially contribute to the pathophysiology of pre-eclampsia: abnormal placental implantation, angiogenesis, excessive inflammation, immune dysfunction. [69] Accuracy performance of vitamin D was increased when combined with angiogenic biomarkers (VEGF + sFLT-1/PlGF).[41] Additionally, all targeted studies showed lower levels of vitamin D metabolites, both in serum and urine, while untargeted LC-MS evaluation observed upregulated levels. Then, based on our results, we cannot rule out vitamin D role in predicting pre-eclampsia in high-risk population or early- or late-onset of disease. It seems there is a place for investigating its role in composing a multifactorial model of prediction.

Environmental exposures

Knowledge of the actual contribution of endocrine disrupting chemicals (EDC), such as BPA, phthalates, and others to pre-eclampsia is relevant, given that behavioural attitude can change maternal exposure to these risk factors. It is hypothesized that BPA can cross the placenta and induce degeneration and necrosis of placental cells, disturbing angiogenesis, [47] and possibly acting through estrogenic-related receptor *gama.* Phthalates can affect placental gene expression determining decreased placental growth, [64] and may also cause a pro-inflammatory response and increase the oxidative stress. [70] Although their role in pre-eclampsia remains elusive, [27,31,47,60] we cannot rule out their role before pregnancy: BPA, phthalates and PFASs are widely present in many products of daily life, and usually have half-lives of many years.

Another environmental factor that was pointed in our review was caffeine. Caffeine metabolism occurs in the cytochrome P450 1A2 system, and the primary metabolite of dietary caffeine (80% of caffeine by-product) is the paraxanthine. The activity of this cytochrome can be demonstrated by the ratio of paraxanthine/caffeine. During pregnancy, the primary pathway for all caffeine clearance happens in maternal cytochrome P450 1A2 system, as neither placenta nor the foetus demonstrates this cytochrome activity. [71] The decreased risk of severe pre-eclampsia with increasing

For peer review only - http://bmjopen.bmj.com/site/about/guidelines.xhtml

paraxanthine/caffeine ratio suggests that faster caffeine metabolism may be associated with a lower risk of pre-eclampsia. [45] This is not exactly in accordance with other studies, which indicated a direct influence of caffeine levels on the risk of pre-eclampsia and failed to perform analysis of caffeine metabolism. [72] On the other hand, caffeine and its metabolites are antagonists of the adenosine A1 receptors, implicated in proximal tubular sodium reabsorption. [73] Although caffeine consumption may be associated to elevated blood pressure in the short term (≤3 hours), it does not represent a cardiovascular risk factor in the long term (2 weeks) for non-pregnant adults. [74] Future studies should consider daily intake of caffeine, which was not explored in the included study, and maternal smoking as potential bias since cigarette use induces the P450 1A2 system. [75]

Methodological quality

Although the majority of included studies were ranked having a low risk of bias and low concerns around applicability, there is still a place for improving data reporting in metabolomics. [76] Interpreting (or using) data from bench analyses in a clinical context requires the clear description of reference standards and index tests, and timing of index test analysis. As preeclampsia has a complex aetiology and is a multifactorial disease, improving patient selection should be a focus of attention in subsequent studies. Better accuracy could be achieved if data from low and high risk women were to be separated, or single versus multiple gestations.

Strengths and limitations

This systematic review can be useful as a guidance of metabolites and their performance as predictors of pre-eclampsia. We have applied a systematic review strategy in eight different electronic databases. Then, we presented possible biomarkers of pre-eclampsia that could ground future research in this area.

Unfortunately, there are limitations. The inclusion of case-control studies could add risk of bias, confounding and potential measurement error. However, we understand this is an important strategy to biomarkers selection and performance evaluation steps in metabolomics [6] in perinatal research. There was great heterogeneity among

For peer review only - http://bmjopen.bmj.com/site/about/guidelines.xhtml

included studies. *Per se*, metabolomics is an overly complex process, influenced by sample collection, storage and preparation, analytical platform applied, statistical tests performed, time of day collection, fasting or non-fasting state prior the samples. [6] Thus, results presented in the studies must consider all these variables. Because of this complexity and authentic heterogeneity of metabolomics, the lack of standardization of pre-eclampsia concept (early and late-onset pre-eclampsia) and time of sample collection represented a limitation to our study. Thus, the results could not be correlated with this important variable. Additionally, since only two studies evaluated pre-eclampsia prediction regarding fetal sex, our findings should be interpreted with caution.

CONCLUSION AND IMPLICATIONS FOR PRACTICE

We have presented a list of 122 different metabolites and their accuracy measures, when possible. They are mainly involved with ammonia recycling; amino acid and lipids metabolism; arachidonic acid metabolism; cell signalling; galactose, lactose, and nucleotide sugars metabolisms. Maternal blood seems to be the best specimen to predict early pre-eclampsia if sampled in the 1st trimester, and to predict late-onset pre-eclampsia in the 2nd trimester. Future metabolomics platforms should be as comprehensive as possible to evaluate the contributions of amino acids, peptides, fatty acid esters, and carbohydrates conjugates, as pre-eclampsia biomarkers.

Although individual metabolites have low predictive measures, they should be considered in multifactorial models of metabolites alone or in combination with other biomarkers. [25,33,38,41,43,46,50,52] Metabolite signature may contribute to further insights into the pathogenesis of pre-eclampsia and to design screening tests, which may contribute to early recognizing high risk women to hypertensive disorders In future, it is important to validate citrate and carnitines as common markers between early-onset and late-onset pre-eclampsia, considering the feasibility of universal screening. [25,49,50] Additionally, since pre-eclampsia and SGA are related to abnormal deep placentation, [77] it seems plausible to study both conditions simultaneously, at least considering the metabolites found in common (sphinganine 1-

For peer review only - http://bmjopen.bmj.com/site/about/guidelines.xhtml

phosphate, sphingosine 1-phosphate, tyrosine, vitamin D, N1, N12-diacetylspermine). [56]

References

1 Mol BWJ, Roberts CT, Thangaratinam S, *et al.* Pre-eclampsia. *Lancet* 2016;**387**:999–1011. doi:10.1016/S0140-6736(15)00070-7

2 L. C. *Chesley's Hypertensive Disorders in Pregnancy*. 4th ed. Elsevier 2015.

3 Roberts JM, Rich-Edwards JW, McElrath TF, *et al.* Subtypes of Preeclampsia: Recognition and Determining Clinical Usefulness. *Hypertension* 2021;:1430–41. doi:10.1161/HYPERTENSIONAHA.120.14781

4 Laganà AS, Favilli A, Triolo O, *et al.* Early serum markers of pre-eclampsia: are we stepping forward? *J Matern Fetal Neonatal Med* 2016;**29**:3019–23.

5 Nobakht M. Gh BF. Application of metabolomics to preeclampsia diagnosis. *Syst Biol Reprod Med* 2018;**64**:324–39. doi:10.1080/19396368.2018.1482968

6 Xia J, Broadhurst DI, Wilson M, *et al.* Translational biomarker discovery in clinical metabolomics: An introductory tutorial. *Metabolomics* 2013;**9**:280–99. doi:10.1007/s11306-012-0482-9

7 Guasch-Ferré M, Hruby A, Toledo E, *et al.* Metabolomics in prediabetes and diabetes: A systematic review and meta-analysis. *Diabetes Care* 2016;**39**:833–46. doi:10.2337/dc15-2251

8 Hoang G, Udupa S, Le A. *Application of metabolomics technologies toward cancer prognosis and therapy*. 1st ed. Elsevier Inc. 2019. doi:10.1016/bs.ircmb.2019.07.003

9 Mayrink J, Leite DFB, Costa ML, *et al.* Metabolomics for prediction of hypertension in pregnancy: A systematic review and meta-analysis protocol. *BMJ Open* 2020;**10**:1–5. doi:10.1136/bmjopen-2020-040652

10 Moher D, Liberati A, Tetzlaff J, *et al.* Preferred Reporting Items for Systematic Reviews and Meta-Analyses: the PRISMA statement. *Ann Intern Med* 2009;**151**:264–9. doi:10.1371/journal.pmed1000097

11 Tranquilli AL, Dekker G, Magee L, *et al.* The classification, diagnosis and management of the hypertensive disorders of pregnancy: A revised statement from the ISSHP. *Pregnancy Hypertens* 2014;**4**:97–104. doi:10.1016/j.preghy.2014.02.001

12 Brown MA, Hague WM, Higgins J, *et al.* The detection, investigation and management of hypertension in pregnancy: Executive summary. *Aust New Zeal J Obstet Gynaecol* 2000;**40**:133–8. doi:10.1111/j.1479-828X.2000.tb01136.x

13 ACOG Committee on Practice Bulletins--Obstetrics. ACOG practice bulletin. Diagnosis and management of preeclampsia and eclampsia. Number 33, January 2002. *Obstet Gynecol* 2002;**99**:159–67. doi:10.1097/00006250-200201000-00028

For peer review only - http://bmjopen.bmj.com/site/about/guidelines.xhtml

For peer review only

14 ACOG. Hypertension in Pregnancy. *Obstet Gynecol* 2013;**122**:1122–31. doi:10.1097/01.AOG.0000437382.03963.88

15 Report of the National High Blood Pressure Education Program Working Group on High Blood Pressure in Pregnancy. *Am J Obstet Gynecol* 2000;**183**:S1–22. doi:10.1067/mob.2000.107928

16 Geraldo J, Ramos L, Sass N, *et al.* Preeclampsia: Definitions of Hypertensive States during Pregnancy Pathophysiological Foundations. *Rev Bras Ginecol Obs* 2017;**39**:496–512.https://pubmed.ncbi.nlm.nih.gov/28793357/

17 Whiting PF, Rutjes AWS, Westwood ME, *et al.* QUADAS-2: A Revised Tool for the Quality Assessment of Diagnostic Accuracy Studies. *Ann Intern Med* 2011;**155**:529–36. doi:10.7326/0003-4819-155-8-201110180-00009

18 Wishart DS, Feunang YD, Marcu A, *et al.* HMDB 4.0: The human metabolome database for 2018. *Nucleic Acids Res* 2018;**46**:D608–17. doi:10.1093/nar/gkx1089

19 Kanehisa M, Goto S. Kyoto Encyclopedia of Genes and Genomes. *Nucleic Acids Res* 2000;**1**:27–30.https://www.genome.jp/kegg/ (accessed 24 Feb 2022).

20 Fahy E, Sud M, Cotter D, *et al.* LIPID MAPS online tools for lipid research. *Nucleic Acids Res* 2007;**35**:606–12. doi:10.1093/nar/gkm324

21 Rutter CM, Gatsonis CA. A hierarchical regression approach to meta-analysis of diagnostic test accuracy evaluations. *Stat Med* 2001;**20**:2865–84. doi:10.1002/sim.942

22 Deeks JJ, Macaskill P, Irwig L. The performance of tests of publication bias and other sample size effects in systematic reviews of diagnostic test accuracy was assessed. *J Clin Epidemiol* 2005;**58**:882–93. doi:10.1016/j.jclinepi.2005.01.016

23 Chappell L. A longitudinal study of biochemical variables in women at risk of preeclampsia. *Am J Obstet Gynecol* 2002;**187**:127–36. doi:10.1067/mob.2002.122969

24 Kenny LC, Broadhurst DI, Dunn W, *et al.* Robust early pregnancy prediction of later preeclampsia using metabolomic biomarkers. *Hypertension* 2010;**56**:741–9. doi:10.1161/HYPERTENSIONAHA.110.157297

25 Bahado-Singh R, Poon LC, Yilmaz A, *et al.* Integrated Proteomic and Metabolomic prediction of Term Preeclampsia. *Sci Rep* 2017;**7**:1–10. doi:10.1038/s41598-017-15882-9

26 Dobierzewska A, Soman S, Illanes SE, *et al.* Plasma cross-gestational sphingolipidomic analyses reveal potential first trimester biomarkers of preeclampsia. *PLoS One* 2017;**12**:1–16. doi:10.1371/journal.pone.0175118

27 Ye Y, Zhou Q, Feng L, *et al.* Maternal serum bisphenol A levels and risk of pre-eclampsia: A nested case-control study. *Eur J Public Health* 2017;**27**:1102–7. doi:10.1093/eurpub/ckx148

28 Gong S, Sovio U, Aye IL, *et al.* Placental polyamine metabolism differs by fetal sex, fetal growth restriction, and preeclampsia. *JCI insight* 2018;**3**:1–15. doi:10.1172/jci.insight.120723

29 Tamblyn JA, Jenkinson C, Larner DP, *et al.* Serum and urine vitamin D metabolite

For peer review only

For peer review only - http://bmjopen.bmj.com/site/about/guidelines.xhtml

analysis in early preeclampsia. *Endocr Connect* 2018;**7**:199–210. doi:10.1530/EC-17-0308

30 Lee SM, Kang Y, Lee EM, *et al.* Metabolomic biomarkers in midtrimester maternal plasma can accurately predict the development of preeclampsia. *Sci Rep* 2020;**10**:16142. doi:10.1038/s41598-020-72852-4

31 Rylander L, Lindh CH, Hansson SR, *et al.* Per- and Polyfluoroalkyl Substances in Early Pregnancy and Risk for Preeclampsia: A Case-Control Study in Southern Sweden. *Toxics* 2020;**8**. doi:10.3390/toxics8020043

32 Harville EW, Li Y-YY-Y, Pan K, *et al.* Untargeted analysis of first trimester serum to reveal biomarkers of pregnancy complications: a case-control discovery phase study. *Sci Rep* 2021;**11**:3468. doi:10.1038/s41598-021-82804-1

33 Kenny LC, Thomas G, Poston L, *et al.* Prediction of preeclampsia risk in first time pregnant women: Metabolite biomarkers for a clinical test. *PLoS One* 2020;**15**:1–19. doi:10.1371/journal.pone.0244369

34 Powe CE, Seely EW, Rana S, *et al.* First trimester vitamin D, vitamin D binding protein, and subsequent preeclampsia. *Hypertension* 2010;**56**:758–63. doi:10.1161/HYPERTENSIONAHA.110.158238

35 Odibo AO, Goetzinger KR, Odibo L, *et al.* First-trimester prediction of preeclampsia using metabolomic biomarkers: a discovery phase study. *Prenat Diagn* 2011;**31**:990–4.

36 Kiely ME, Zhang JY, Kinsella M, *et al.* Vitamin D status is associated with uteroplacental dysfunction indicated by pre-eclampsia and small-for-gestational-age birth in a large prospective pregnancy cohort in Ireland with low Vitamin D status. *Am J Clin Nutr* 2016;**104**:354–61. doi:10.3945/ajcn.116.130419

37 Huo X, Huang R, Gan Y, *et al.* Perfluoroalkyl substances in early pregnancy and risk of hypertensivedisorders of pregnancy: A prospective cohort study. *Environ Int* 2020;**138**. doi:10.1016/j.envint.2020.105656

38 Sovio U, McBride N, Wood AM, *et al.* 4-Hydroxyglutamate is a novel predictor of pre-eclampsia. *Int J Epidemiol* 2020;**49**:301–11. doi:10.1093/ije/dyz098

39 Kenny LC, Broadhurst DI, Hong W, *et al.* NMR Profiling in Early Pregnancy Reveals a Metabolomic Signature the Predicts the Subsequent Development Preeclampsia. *Reprod Sci* 2013;**20**:2641A. doi:10.1177/1933719113482088

40 Huang Q, Hao S, You J, *et al.* Early-pregnancy prediction of risk for pre-eclampsia using maternal blood leptin/ceramide ratio: Discovery and confirmation. *BMJ Open* 2021;**11**. doi:10.1136/bmjopen-2021-050963

41 Woodham PC, Brittain JE, Baker AM, *et al.* Midgestation maternal serum 25-hydroxyvitamin D level and soluble fms-like tyrosine kinase 1/placental growth factor ratio as predictors of severe preeclampsia. *Hypertension* 2011;**58**:1120–5. doi:10.1161/HYPERTENSIONAHA.111.179069

42 Khalil AA, Tsikas D, Akolekar R, *et al.* Asymmetric dimethylarginine, arginine and homoarginine at 11-13 weeks gestation and preeclampsia: A case-control study. *J Hum Hypertens* 2013;**27**:38–43. doi:10.1038/jhh.2011.109

For peer review only

For peer review only - http://bmjopen.bmj.com/site/about/guidelines.xhtml

43 Kuc S, Koster MPH, Pennings JLA, *et al.* Metabolomics profiling for identification of novel potential markers in early prediction of preeclampsia. *PLoS One* 2014;**9**. doi:10.1371/journal.pone.0098540

44 Wetta LA, Biggio JR, Cliver S, *et al.* Is midtrimester vitamin D status associated with spontaneous preterm birth and preeclampsia? *Am J Perinatol* 2014;**31**:541–6. doi:10.1055/s-0033-1356483

45 Eichelberger KY, Baker AM, Woodham PC, *et al.* Second-trimester maternal serum paraxanthine, CYP1A2 activity, and the risk of severe preeclampsia. *Obstet Gynecol* 2015;**126**:725–30. doi:10.1097/AOG.0000000000001041

46 Koster MPH, Vreeken RJ, Harms AC, *et al.* First-Trimester serum acylcarnitine levels to predict preeclampsia: A metabolomics approach. *Dis Markers* 2015;**2015**. doi:10.1155/2015/857108

47 Cantonwine DE, Meeker JD, Ferguson KK, *et al.* Urinary concentrations of bisphenol A and phthalate metabolites measured during pregnancy and risk of preeclampsia. *Environ Health Perspect* 2016;**124**:1651–5. doi:10.1289/EHP188

48 Rijvers CAH, Marzano S, Winkens B, *et al.* Early-pregnancy asymmetric dimethylarginine (ADMA) levels in women prone to develop recurrent hypertension. *Pregnancy Hypertens* 2013;**3**:118–23. doi:10.1016/j.preghy.2013.01.001

49 Diaz SO, Barros AS, Goodfellow BJ, *et al.* Second trimester maternal urine for the diagnosis of trisomy 21 and prediction of poor pregnancy outcomes. *J Proteome Res* 2013;**12**:2946–57. doi:10.1021/pr4002355

50 Bahado-Singh RO, Syngelaki A, Akolekar R, *et al.* Validation of metabolomic models for prediction of early-onset preeclampsia. *Am J Obstet Gynecol* 2015;**213**:530.e1-530.e10. doi:10.1016/j.ajog.2015.06.044

51 Ates S, Sevket O, Ozcan P, *et al.* Vitamin D status in the first-trimester: Effects of vitamin D deficiency on pregnancy outcomes. *Afr Health Sci* 2016;**16**:36–43. doi:10.4314/ahs.v16i1.5

52 Bahado-Singh RO, Syngelaki A, Mandal R, *et al.* Metabolomic determination of pathogenesis of late-onset preeclampsia. *J Matern Neonatal Med* 2017;**30**:658–64. doi:10.1080/14767058.2016.1185411

53 Shanmugalingam R, Wang X, Motum P, *et al.* The 15-Epilipoxin-A4 Pathway with Prophylactic Aspirin in PreventingPreeclampsia: A Longitudinal Cohort Study. *J Clin Endocrinol Metab* 2020;**105**. doi:10.1210/clinem/dgaa642

54 Bilodeau JF, Qin Wei S, Larose J, *et al.* Plasma F2-isoprostane class VI isomers at 12-18 weeks of pregnancy are associated with later occurrence of preeclampsia. *Free Radic Biol Med* 2015;**85**:282–7. doi:10.1016/j.freeradbiomed.2015.05.012

55 Gong S, Sovio U, Aye ILMH, *et al.* Placental polyamine metabolism differs by fetal sex, fetal growth restriction, and preeclampsia. *JCI Insight* 2018;**3**:1–15. doi:10.1172/jci.insight.120723

56 Leite DFB, Morillon A-C, Junior EF de M, *et al.* Examining the predictive accuracy of metabolomics for small for gestational age babies: a systematic review. *BMJ Open* 2019;**9**:e031238. doi:10.1136/ bmjopen-2019-031238

For peer review only

For peer review only - http://bmjopen.bmj.com/site/about/guidelines.xhtml

57 Bahado-Singh RO, Akolekar R, Mandal R, *et al.* First-trimester metabolomic detection of late-onset preeclampsia. *Am J Obstet Gynecol* 2013;**208**:58.e1-58.e7. doi:10.1016/j.ajog.2012.11.003

58 Nelson DB, Ziadie MS, McIntire DD, *et al.* Placental pathology suggesting that preeclampsia is more than one disease. *Am J Obstet Gynecol* 2014;**210**:66.e1-66.e7. doi:10.1016/j.ajog.2013.09.010

59 Khalaf D, Krüger M, Wehland M, *et al.* The effects of oral l-arginine and l-citrulline supplementation on blood pressure. *Nutrients* 2019;**11**:1–14. doi:10.3390/nu11071679

60 Huo X, Huang R, Gan Y, *et al.* Perfluoroalkyl substances in early pregnancy and risk of hypertensive disorders of pregnancy: A prospective cohort study. *Environ Int* 2020;**138**:1–7. doi:10.1016/j.envint.2020.105656

61 Levine RJ, Lam C, Qian C, *et al.* Soluble Endoglin and Other Circulating Antiangiogenic Factors in Preeclampsia. *N Engl J Med* 2006;**355**:992–1005.

62 Wang C, Kong L, Yang Y, *et al.* Recommended reference values for serum lipids during early and middle pregnancy: A retrospective study from China 11 Medical and Health Sciences 1114 Paediatrics and Reproductive Medicine. *Lipids Health Dis* 2018;**17**:1–16. doi:10.1186/s12944-018-0885-3

63 Wojcik-Baszko D, Charkiewicz K, Laudanski P. Role of dyslipidemia in preeclampsia—A review of lipidomic analysis of blood, placenta, syncytiotrophoblast microvesicles and umbilical cord artery from women with preeclampsia. *Prostaglandins Other Lipid Mediat* 2018;**139**:19–23. doi:10.1016/j.prostaglandins.2018.09.006

64 Aris A, Benali S, Ouellet A, *et al.* Potential Biomarkers of Preeclampsia: Inverse Correlation between Hydrogen Peroxide and Nitric Oxide Early in Maternal Circulation and at Term in Placenta of Women with Preeclampsia. *Placenta* 2009;**30**:342–7. doi:10.1016/j.placenta.2009.01.003

65 de Carvalho LP, Tan SH, Ow GS, *et al.* Plasma Ceramides as Prognostic Biomarkers and Their Arterial and Myocardial Tissue Correlates in Acute Myocardial Infarction. *JACC Basic to Transl Sci* 2018;**3**:163–75. doi:10.1016/j.jacbts.2017.12.005

66 Anand S, Young SA, Esplin MS, *et al.* Detection and confirmation of serum lipid biomarkers for preeclampsia using direct infusion mass spectrometry. *J Lipid Res* 2016;**57**:687–96. doi:10.1194/jlr.P064451

67 Cardus A, Panizo S, Encinas M, *et al.* 1,25-Dihydroxyvitamin D3 regulates VEGF production through a vitamin D response element in the VEGF promoter. *Atherosclerosis* 2009;**204**:85–9. doi:10.1016/j.atherosclerosis.2008.08.020

68 Baker AM, Haeri S, Camargo CA, *et al.* A nested case-control study of midgestation vitamin D deficiency and risk of severe preeclampsia. *J Clin Endocrinol Metab* 2010;**95**:5105–9. doi:10.1210/jc.2010-0996

69 Westwood M, Al-Saghir K, Finn-Sell S, *et al.* Vitamin D attenuates sphingosine-1-phosphate (S1P)-mediated inhibition of extravillous trophoblast migration. *Placenta* 2017;**60**:1–8. doi:10.1016/j.placenta.2017.09.009

70 Werner EF, Braun JM, Yolton K, *et al.* The association between maternal urinary phthalate concentrations and blood pressure in pregnancy: The HOME Study. *Environ*

For peer review only

For peer review only - http://bmjopen.bmj.com/site/about/guidelines.xhtml

Heal A Glob Access Sci Source 2015;**14**:1–9. doi:10.1186/s12940-015-0062-3

71 Grosso LM, Bracken MB. Caffeine metabolism, genetics, and perinatal outcomes: A review of exposure assessment considerations during pregnancy. *Ann Epidemiol* 2005;**15**:460–6. doi:10.1016/j.annepidem.2004.12.011

72 Bakker R, Steegers EAP, Raat H, *et al.* Maternal caffeine intake, blood pressure, and the risk of hypertensive complications during pregnancy. the generation R study. *Am J Hypertens* 2011;**24**:421–8. doi:10.1038/ajh.2010.242

73 Guessous I, Eap CB, Bochud M. Blood Pressure in Relation to Coffee and Caffeine Consumption. *Curr Hypertens Rep* 2014;**16**:1–9. doi:10.1007/s11906-014-0468-2

74 Mesas AE, Leon-Muñoz LM, Rodriguez-Artalejo F, *et al.* The effect of coffee on blood pressure and cardiovascular disease in hypertensive individuals: A systematic review and meta-analysis. *Am J Clin Nutr* 2011;**94**:1113–26. doi:10.3945/ajcn.111.016667

75 Dobrinas M, Cornuz J, Eap CB. Pharmacogenetics of CYP1A2 activity and inducibility in smokers and exsmokers. *Pharmacogenet Genomics* 2013;**23**:286–92. doi:10.1097/FPC.0b013e3283602e75

76 Goodacre R, Broadhurst D, Smilde AK, *et al.* Proposed minimum reporting standards for data analysis in metabolomics. *Metabolomics* 2007;**3**:231–41. doi:10.1007/s11306-007-0081-3

77 Brosens I, Pijnenborg R, Vercruysse L, *et al.* The ‘great obstetrical syndromes’ are associated with disorders of deep placentation. *Am J Obs Gynecol* 2011;**204**:193–201. doi:10.1016/j.ajog.2010.08.009.THE

For peer review only

For peer review only - http://bmjopen.bmj.com/site/about/guidelines.xhtml

Legends

Figures

Figure 1. Flow chart of study identification, screening, eligibility and inclusion.

Figure 2. Characterization of study selection regarding biological samples, sampling moment, and metabolomic analysis techniques applied

Figure 3. Assessment of risk of bias (left) and applicability concerns (right) of included studies.

Figure 4. Preeclampsia related metabolites, chemical classes, and biological processes.

Tables

Table 1. Characteristics of included studies.

Table 2. Metabolites and their accuracy measures for predicting preeclampsia according to gestational age of required delivery.

Table 3 – Accuracy measures of metabolites and other pregnancy-related biomarkers.

Supplementary Material

Supplementary material 1 Preferred Reported Items for Systematic Reviews and Meta-Analysis (PRISMA) statement: a PRISMA checklist

Supplementary material 2 – Full search strategy for each database.

Supplementary material 3- Excluded studies and reasons for exclusion.

Supplementary material 4 - Predictive metabolites of hypertensive disorders of pregnancy summarized according to their superclass, chemical class, subclass, and metabolic pathways.

Supplementary material 5 – Individual QUADAS-2 data for all included studies.

ACKNOWLEDGEMENTS

We are grateful to all members of the PRETERM-SAMBA study research group.

For peer review only - http://bmjopen.bmj.com/site/about/guidelines.xhtml

AUTHORS CONTRIBUTIONS

JM is the guarantor of this review. JM and DFBL elaborated the protocol, searched the literature, selected studies, extracted data, assessed risk of bias, and drafted the initial manuscript. MLC has helped in study inclusion, interpreting data, examining clinical applications of findings, and revising the manuscript. GN has helped in figures and tables elaboration and with the final version of the manuscript. JGC have supervised and approved all steps. All authors have read and agree with this submission.

FUNDING

DFBL (process number 88881.134512/2016-01) has awarded scholarship from Brazilian Federal Agency for Support and Evaluation of Graduate Education (CAPES). This research is part of the PRETERM-SAMBA study, which has granted sponsor from Brazilian National Research Council (CNPq) (Award 401636/2013-5) and from the Bill and Melinda Gates Foundation (grant OPP1107597). This specific analysis received no specific grant from any sectors. Our sponsors have not intervened in any step of this study design, development, or submission.

COMPETING INTERESTS

None to declare.

PATIENT CONSENT FOR PUBLICATION

Not required.

PROVENANCE AND PEER REVIEW

Not commissioned; externally peer reviewed.

DATA AVAILABILITY STATEMENT

No data are available.

For peer review only

Table 1. Characteristics of included studies.

Authors, year	Country of data collection	Study design	Classification of hypertensive syndrome	Comments
Chappell LC et al, 2002 [1]	United Kingdom	Case-control	ISSHP, 1988	17 women with preeclampsia; 21 without preeclampsia. Chronic hypertensive women included.
Powe CE et al, 2010 [2]	United States of America	Nested case-control	ICD codes	39 preeclampsia, 131 controls. Gestational diabetes excluded.
Kenny LC et al, 2010 [3]	New Zealand; Australia	Nested case-control	ASSHP, 2000	Discovery phase (New Zealand): 60 women with preeclampsia, 60 matched controls (age, ethnicity, body mass index) with uneventful pregnancies. Validation phase (Australia): 39 women with preeclampsia, 40 healthy matched pregnancies (SCOPE study).
Odibo AO et al, 2011 [4]	United States of America	Nested case-control	ACOG, 2002	41 women with preeclampsia, 41 normotensive controls. Pregestational diabetes included.
Woodham PC et al, 2011 [5]	United States of America	Nested case-control	ACOG, 2002	41 women with severe preeclampsia, 123 normotensive uncomplicated pregnancies at term. Chronic illness and multiple pregnancies excluded.
Rijvers CAH et al, 2013 [6]	Netherlands	Retrospective cohort	NHBP, 2000	06 women with preeclampsia, 11 with gestational hypertension, 18 normotensive women
Khalil AA et al, 2013 [7]	United Kingdom	Case-control	ISSHP, 2001	25 women with early preeclampsia, 50 cases of late preeclampsia, 300 normotensive controls (matching 1 case: 4 controls). Only singleton pregnancies; chronic hypertensive women included.
Diaz SO et al, 2013 [8]	Portugal	Prospective cohort	ACOG, 2002	09 women with preterm preeclampsia (31-37weeks); 84 women with healthy term pregnancies

For peer review only - http://bmjopen.bmj.com/site/about/guidelines.xhtml

Kenny LC et al, 2013 [9]	New Zealand; Australia	Nested case-control	ASSHP, 2000	49 women with preeclampsia and 49 normotensive nulliparous women. (SCOPE study).
Kuc S et al, 2014 [10]	Netherlands	Nested case-control	ISSHP, 2001	68 early-onset preeclampsia, 99 late-onset preeclampsia, 500 controls
Wetta LA et al, 2014 [11]	United States of America	Nested case-control	ACOG, 2002	89 women with preterm preeclampsia; 177 normotensive and term pregnancies
Bahado-Singh RO et al, 2015 [12]	United Kingdom	Prospective cohort	ISSHP, 2001	50 cases of early preeclampsia, 108 normotensive controls. Singleton pregnancies delivered at term; birthweight adequate for gestational age. HELLP syndrome cases excluded.
Eichelberger KY et al, 2015 [13]	United States of America	Nested case-control	ACOG, 2002	25 women with severe preeclampsia; 87 normotensive, healthy pregnancies delivered at term. Chronic hypertensive women and multiple pregnancies excluded.
Koster MPH et al, 2015 [14]	Netherlands	Nested case-control	ISSHP, 2001	68 early-onset preeclampsia, 99 late-onset preeclampsia, 500 controls
Bilodeau JF et al, 2015 [15]	Canada	Prospective cohort	CHS, 1997	33 preeclampsia, 60 controls (MIROS study)
Ates S et al, 2016 [16]	Turkey	Prospective cohort	ACOG, 2002	04 women with preeclampsia; 11 women with gestational hypertension; 214 normotensive women
Cantonwine DE et al, 2016 [17]	United States of America	Nested case-control	ACOG, 2002	50 women with preeclampsia, 431 without preeclampsia. Chronic hypertensive women included.
Kiely ME et al, 2016 [18]	Ireland	Nested case-control	ASSHP, 2000	68 preeclampsia, 1528 controls. SGA infants excluded from controls. (SCOPE Study)
Bahado-Singh RO et al,	United Kingdom	Prospective cohort	ISSHP, 2001	59 late-onset preeclampsia cases, 115 normotensive controls. Singleton

For peer review only - http://bmjopen.bmj.com/site/about/guidelines.xhtml

2017 [19]				pregnancies delivered at term; birthweight adequate for gestational age. HELLP syndrome cases excluded.
Bahado-Singh RO et al, 2017b [20]	United Kingdom	Nested case-control	ISSHP, 2001	35 term preeclampsia cases (≥37w), 65 normotensive controls. Singleton pregnancies, no major malformations; controls with birthweight adequate for gestational age.
Dobierzewska A et al, 2017 [21]	Chile	Case-control	ACOG, 2002	07 women with preeclampsia; 07 normotensive women
Ye Y et al, 2017 [22]	China	Nested case-control	ACOG, 2013	74 women with preeclampsia, 99 normotensive term pregnancies as controls. Only singleton pregnancies; chronic illness excluded in control group.
Gong S et al, 2018 [23]	United Kingdom	Case-control	ACOG, 2002	134 term preeclampsia, 259 normotensive controls. Nulliparous women, singleton pregnancies (POP Study).
Tamblyn JA et al, 2018 [24]	Ireland	Case-control	ASSHP, 2000	25 women with preeclampsia, 25 normotensive women (SCOPE Study).
Sovio U et al, 2020 [25]	United Kingdom	Case-cohort	ISSHP, 2001; ACOG, 2013	Training and test with POP Study: 194 women with preeclampsia, 323 controls. Validation within BiB Study: 95 women with preeclampsia, 953 normotensive controls.
Huo X et al, 2020 [26]	Shanghai, China	Case-Cohort	ACOG, 2013	64 gestational hypertension, 71 preeclampsia. Singleton gestation only; chronic hypertensive women excluded.
Rylander L et al, 2020 [27]	Sweden	Case-control	ICD codes	296 preeclampsia, 580 controls. Excluded pregnancies with SGA infants.
Lee SM et al, 2020 [28]	South Korea	Case-control	ACOG, 2013	33 preeclampsia, 66 controls. Singleton pregnancies, congenital

For peer review only - http://bmjopen.bmj.com/site/about/guidelines.xhtml

For peer review only

				malformations excluded.
Kenny L et al, 2020 [29]	Ireland and England	Nested case-control	ISSHP, 2018	97 preeclampsia (23 preterm preeclampsia and 74 term preeclampsia); 335 controls. Nulliparous women, singleton pregnancies. Chronic illness and fetal malformations excluded (SCOPE study)
Shanmugalingam R et al, 2020 [30]	Australia	Prospective cohort	ISSHP, 2018	21 preeclampsia, 103 controls. High risk women.
Harville EW et al, 2021 [31]	United States of America	Case-control	Not mentioned	18 preeclampsia, 109 controls. Only singleton pregnancies.
Huang Q et al, 2021 [32]	United States of America	Case-control	ACOG, 2013	20 preeclampsia, 20 controls (validation phase).

ACOG: American College of Obstetricians and Gynecologists; CHS, Canadian Hypertensive Society; ASSHP: Australasian Society of the Study of Hypertension in Pregnancy; ICD, International Classification of Diseases; BiB Study: Born in Bradford Study; ISSHP: International Society for the Study of Hypertension in Pregnancy; POP Study: Pregnancy Outcome Prediction; SCOPE Study: Screening for Pregnancy Endpoints; MIROS study: Maternal and Infant Research on Oxidative Stress; PTB: preterm birth; SGA, small for gestational age.

For peer review only

For peer review only - http://bmjopen.bmj.com/site/about/guidelines.xhtml

Table 2. Metabolites and their accuracy measures for predicting preeclampsia according to gestational age of required delivery.

Preeclampsia at any gestational age					
**Authors, year**	**Metabolomics methods**	**Maternal biological specimen; gestational age at sampling**	**Increased metabolites**	**Decreased metabolites**	**S/ Sp/ AUC (95% CI)**
Chappell LC et al, 2002 [1]	GC-MS	Blood; 20w	Isoprostane 8-epi-prostaglandin F2α		-/ -/ 0.62 (0.44-0.81)
		Blood; 24w	Isoprostane 8-epi-prostaglandin F2α		-/ -/ 0.55 (0.35-0.75)
Powe CE et al, 2010 [2]	Targeted LC-MS/MS	Blood (serum); 11w (±3w)		25(OH)vitamin D [a]	-/ -/ -
Odibo AO et al, 2011 [4]	Targeted LC-MS/MS	Blood (serum); 11-14w	Hydroxyhexanoylcarnitine, phenylalanine, glutamate, alanine		-/ -/ 0.82 (0.80–0.85)
Woodham PC et al, 2011 [5]	Targeted LC-MS/MS	Blood (serum); 15-20w		25(OH)vitamin D	-/ -/ 0.745
Diaz SO et al, 2013 [8]	Untargeted ^{1}H-NMR	Urine; 14-26w	*N*-methyl-2-pyridone-5-carboxamide, 3-methylhistidine, 4-deoxyerythronic acid, *cis*-aconitate, citrate, Glutamine, hippurate, indoxyl sulphate, *N*-methylnicotinamide, sucrose, U35	2-ketoglutarate, 4-OH-hippurate, acetate, carnitine, creatinine, formate, fumarate, galactose, isoleucine, lactose, phenylacetylglutamine, *p*-cresol sulphate, *scyllo*-ino, succinate, trigonelline, tyrosine, U14, U4, U10	-/ -/ -
Kenny LC et al, 2013 [9]	Untargeted H-NMR	Blood (serum); 15w		Acetamide, Glutamine, Trimethylamine, creatinine, dimethyl sulfone, valine,	-/-/-

For peer review only

For peer review only - http://bmjopen.bmj.com/site/about/guidelines.xhtml

				isoleucine, ornithine, glycine, methionine, betaine, acetate, proline	
Rijvers CAH et al, 2013 [6]	Targeted UPLC-MS/MS	Blood (plasma); 12w	L-arginine/ Asymmetric dimethylarginine ratio		-/ -/ -
		Blood (plasma); 16-20w		L-arginine	-/ -/ -
Wetta LA et al, 2014 [11]	Targeted LC-MS/MS	Blood (serum); 15-21w		25(OH)vitamin D [a]	-/ -/ -
Bilodeau JF et al, 2015 [15]	Targeted HPLC-MS/MS	Blood (plasma); 12-18w	(+/-) 5-iPF2α-VI, (+/-) 5-iPF2α-VI-(d11)		-/ -/ 0.67
	GC-FID	Blood (plasma); 12-18w		Alfa-linolenic acid, stearidonic acid, eicosatrienoic acid, eicosatetraenoic acid, eicosapentaenoic acid, docosapentaenoic acid, docosahexaenoic acid	-/ -/ -
Ates S et al, 2016 [16]	Targeted LC-MS/MS	Blood (serum); 11-14w		Vitamin D (<10mg/dL)	0.50/0.54
Cantonwine DE et al, 2016 [17]	Targeted HPLC-MS	Urine; 4-16w	Total bisphenol A, Σ di(2-ethylhexyl) phthalate, mono-ethyl phthalate	%mono(2-ethylhexyl) phthalate	-/ -/ -
		Urine; 22-29w	mono(2-ethylhexyl) phthalate, Σ di(2-ethylhexyl) phthalate, mono(3-carboxypropyl) phthalate	monoisobutyl phthalate	-/ -/ -
		Urine; 33-38w	mono(2-ethylhexyl) phthalate, Σ di(2-ethylhexyl) phthalate, mono(3-		-/ -/ -

			carboxypropyl) phthalate		
Kiely ME et al, 2016 [18]	Targeted LC-MS/MS	Blood (serum); 14-16w		25(OH)D3, 3-epi-25(OH)D3, and 25(OH)D2 [a]	-/ -/ -
Dobierzewska A et al, 2017 [21]	Targeted HPLC-MS	Blood (plasma); 11-14w		Sphingomyelin 16:0, sphingomyelin 18:0, ceramide 14:0	-/ -/ -
		Blood (plasma); 32-36w		Ceramide 14:0, ceramide 24:0	-/ -/ -
Ye Y et al, 2017 [22]	Targeted LC-MS	Blood (serum); 16-20w	Free bisphenol A		-/ -/ 0.73 (0.65-0.81)
Tamblyn JA et al, 2018 [24]	Targeted LC-MS/MS	Urine; 14-16w		25(OH) vitamin D3; 24,25(OH)2 vitamin D3[a]	-/ -/ -
Huo X et al, 2020 [26]	HPLC/MS-MS	Blood (serum); 13-17w	Perfluorooctanate; perfluorooctane sulfonate; perfluorodecanoic acid; perfluoroundecanoic acid; perfluorononanoic acid; perfluorohexanesulfonate; perfluoroheptanoic acid; perfluorobutane sulfonate; perfluorododecanoic acid; perfluorooctane sulfonamide[a]		-/ -/ -
Rylander L et al, 2020 [27]	Targeted LC-MS/MS	Blood (serum); 12-14w	Perfluorohexane sulfonate (0.53–0.78 ng/mL)		0.38/ 0.67/ -
Lee SM et al, 2020 [28]	GC-TOF MS	Blood (plasma); 16-24w		Propane-1,3-diol	0.75/ 0.83/ 0.868 (0.844-0.891)
	LC-Orbitrap	Blood (plasma);	LysoPE C20:0; SM C30:1	LysoPC C19:0; SM C28:1	

For peer review only

	MS	16-24w			
Kenny L et al, 2020 [29]	Targeted LC-MS/MS	Blood (plasma); 14-16w	Dilinoleoyl-glycerol (DLG)		0.39/ 0.90/0.70 (0.59-0.82)
Shanmugalingam R et al, 2020 [30]	Targeted LC-MS/MS	Blood (plasma); 12w		15-epilipoxin-A4	
Harville EW et al, 2021 [31]	Untargeted UPLC-HR-MS	Blood (serum); 6-14w	Cerasinone; bolasterone, 6.30_477.7721m/z, 5.86_313.1070m/z[b]		-/ -/ 0.95
	NMR	Blood (serum); 6-14w		Asparagine, N,N-Dimethylglycine, Trimethylamine	-/ -/ -
Huang Q et al, 2021 [32]	LC-MS	Blood (serum); 5-15w		Cer(d18:1/25:0)	-/-/0.868
		Blood (serum); 16-29w		Cer(d18:1/25:0)	-/-/0.747
**Early onset preeclampsia (<34w)**					
Odibo AO et al, 2011 [4]	Targeted LC-MS/MS	Blood (serum); 11-14w	Hydroxyhexanoylcarnitine, phenylalanine, glutamate, alanine		-/ -/ 0.84
Khalil AA et al, 2013 [7]	Targeted GC-MS; GC-MS/MS	Blood (plasma); 11-13w	asymmetric dimethylarginine/ L-arginine ratio; asymmetric dimethylarginine/ L-homoarginine ratio	L-arginine, L-homoarginine	-/ -/ -
Kuc S et al, 2014 [10]	Targeted UPLC-MS/MS	Blood (serum); 8-13w		Taurine, asparagine	-/ -/ -
Bahado-Singh RO et al, 2015 [12]	Untargeted ^{1}H-NMR	Blood (serum); 11-13w	2-hydroxybutyrate, 3-hydroxyisovalerate, citrate	Acetone, glycerol	0.75/ 0.74/ 0.835 (0.769-0.941)

For peer review only - http://bmjopen.bmj.com/site/about/guidelines.xhtml

Eichelberger KY et al, 2015 [13]	LC-MS	Blood (serum); 16-19w		Paraxanthine/caffeine ratio	-/ -/ -
Koster MPH et al, 2015 [14]	Targeted UPLC-MS/MS	Blood (serum); 8-13w	Hexanoylcarnitine, octenoylcarnitine, octanoylcarnitine, decenoylcarnitine, decanoylcarnitine, dodecanoylcarnitine, lauroylcarnitine, tetradecenoylcarnitine, hexadecenoylcarnitine	Isobutyrylcarnitine, linoleylcarnitine, stearoylcarnitine	-/ -/ -
Sovio U et al, 2020 [25]	Untargeted UPLC-MS/MS	Blood (serum); 12w<br>Blood (serum); 20w<br>Blood (serum); 28w	4-hydroxyglutamate		-/-/0.673<br>-/-/0.731<br>-/-/0.765
**Late onset preeclampsia (≥34w)**					
Kenny LC et al, 2010 [3]	Untargeted UPLC-MS	Blood (plasma); 14-16w	Monosaccharide(s), decanoylcarnitine, oleic acid, docosahexaenoic acid and/or docosatriynoic acid, gama-butyrolactone and/or oxolan-3-one, 2-oxovaleric acid and/or oxo-methylbutanoic acid, acetoacetic acid, hexadecenoyleicosatetraenoyl-Sn-glycerol, Di-(octadecadienoyl)-sn-glycerol, sphingosine 1-phosphate, sphinganine 1-phosphate, vitamin D3 derivatives	5-Hydroxytryptophan, methylglutaric acid and/or adipic acid	-/ -/ 0.92[c]

For peer review only

Kuc S et al, 2014 [10]	Targeted UPLC-MS/MS	Blood (serum); 8-13w		Glycylglycine	-/ -/ -
Koster MPH et al, 2015 [14]	Targeted UPLC-MS/MS	Blood (serum); 8-13w	Hexanoylcarnitine, octenoylcarnitine, octanoylcarnitine, decenoylcarnitine, decanoylcarnitine, dodecanoylcarnitine, tetradecenoylcarnitine	Linoleylcarnitine, palmitoylcarnitine, oleylcarnitine, stearoylcarnitine	-/ -/ -
Cantonwine DE et al, 2016 [17]	Targeted HPLC-MS	Urine; 4-16w	mono(2- ethyl-5-oxohexyl) phthalate, mono(2-ethyl-5-carboxypentyl) phthalate, Σ di(2-ethylhexyl) phthalate, mono-ethyl phthalate		-/ -/ -
		Urine; 33-38w	mono(2- ethyl-5-oxohexyl) phthalate, mono(2-ethyl-5-carboxypentyl) phthalate, Σ di(2-ethylhexyl) phthalate, Mono-*n*-butyl phthalate		-/ -/ -
Bahado-Singh RO et al, 2017 [19]	Untargeted ^{1}H-NMR	Blood (serum); 11-13w	Carnitine, pyruvate, acetone	-	0.30/ 0.80/ 0.629 (0.490-0.767)
Bahado-Singh RO et al, 2017b [20]	Untargeted ^{1}H-NMR, targeted LC-MS/MS	Blood (serum); 11-13w	-	Putrescine (LC-MS); urea, carnitine (H-NMR)	0.72/ 0.57/ 0.701
		Blood (serum); 32-33w	Methylhistidine, propylene glycol (H-NMR), citrate, hexose[d]	Serotonin (LC-MS)	0.74/ 0.72/ 0.761
Gong S et al, 2018	Untargeted	Blood (serum); 36w	N1, N12-diacetylspermine[e]	-	-

For peer review only - http://bmjopen.bmj.com/site/about/guidelines.xhtml

[23]	LC-MS/MS				
**Gestational hypertension**					
Ates S et al, 2016 [16]	Targeted LC-MS/MS	Blood (serum); 11-14w		Vitamin D (<10mg/dL)	0.45/0.54
Huo X et al, 2020 [26]	HPLC/ MS-MS	Blood (serum); 13-17w	Perfluoroheptanoic acid[f]		-/ -/ -
Harville EW et al, 2021 [31]	Untargeted UPLC-HR-MS	Blood (serum); 6-14w	Neomenthol-glucuronide, dibenzylamine, pilocarpine, 2,6-Di-*tert*-butyl-4-hydroxymethylphenol, 0.59_746.6045n, 8.66_762.1452m/z, 12.74_412.2842m/z		-/ - / 0.95[b]

AUC: area under the curve; w: weeks; GC-MS: gas-chromatography coupled to mass spectrometry; GC-FID: gas-chromatography coupled with a flame ionization detector; ^{1}H-NMR: hydrogen nuclear magnetic resonance; HPLC-MS: High Performance Liquid Chromatography- mass spectrometry; LC-MS: liquid- chromatography coupled to mass spectrometry; GC-TOF MS: gas chromatography time-of-flight mass spectrometry; LC-Orbitrap MS: liquid chromatography Orbitrap mass spectrometry. S, sensitivity; Sp, specificity; UPLC-MS: ultra- Performance Liquid Chromatography- mass spectrometry; 95% CI, 95% confidence interval.

[a]No statistical difference between preeclampsia and normotensive controls. [b]AUC for all metabolites model; some of them were not identified, thus they are presented as retention time and mass; it is unclear which metabolite was up or downregulated in preeclampsia cases. [c]Accuracy measures for the validation phase. [d]Unclear type of platform that has found this metabolite, or if it is increased or decreased in preeclampsia. [e]Higher preeclampsia risk with increasing N1, N12-diacetylspermine concentrations. [f] No statistical difference between preeclampsia normotensive controls.

For peer review only

Table 3 – Accuracy measures of metabolites and other pregnancy-related biomarkers.

Maternal specimen	Trimester of pregnancy	Metabolites	Additional variables considered in prediction models	AUC of metabolites + additional variables model (95% CI)
**Preeclampsia at any gestational age**				
Blood	1st	Dilinoleoyl-glycerol heptadecanoyl-2-hydroxy-sn-glycero-3-phosphocholine	PlGF	0.78 (0.69-0.88) [29]
		Ceramide(d18:1/25:0)[a]	Leptin	0.876 [32]
Blood	2nd	25OHD	VEGF + sFLT-1/PlGF	0.851 [5]
		Ceramide(d18:1/25:0)[a]	Leptin	0.892 [32]
Blood	3rd	4-hydroxyglutamate	s-Flt-1/PlGF	0.834 [25]
**Early onset preeclampsia (delivery <34w)**				
Blood	1st	Taurine	Prior risk+ MAP	0.93 [10]
		Stearoylcarnitine	Prior risk+ MAP	0.747 [14]
		3-hydroxyisovalerate, arginine, glycerol	UtPI	0.917 [12]
**Late onset preeclampsia (delivery ≥34w)**				
Blood	1st	Pyruvate, carnitine, glycerol	UtPI	0.722 [19]
Blood	1st	Stearoylcarnitine	Prior risk+ MAP	0.828 [14]
Blood	1st and 3rd	Urea, SM C18:1 (1st), Hexose, Citrate (3rd)	Maternal BMI (12w) + MAP (32w)	0.805 [20]

AUC: area under the curve; BMI: body mass index; MAP, mean arterial pressure; UtPI: uterine arteries pulsatility index; CI: confidence interval.

[a]Increased leptin/Ceramide(d18:1/25:0) ratio in preeclamptic women.

For peer review only

For peer review only - http://bmjopen.bmj.com/site/about/guidelines.xhtml

References

1 Chappell L. A longitudinal study of biochemical variables in women at risk of preeclampsia. *Am J Obstet Gynecol* 2002;**187**:127–36. doi:10.1067/mob.2002.122969

2 Powe CE, Seely EW, Rana S, *et al.* First trimester vitamin D, vitamin D binding protein, and subsequent preeclampsia. *Hypertension* 2010;**56**:758–63. doi:10.1161/HYPERTENSIONAHA.110.158238

3 Kenny LC, Broadhurst DI, Dunn W, *et al.* Robust early pregnancy prediction of later preeclampsia using metabolomic biomarkers. *Hypertension* 2010;**56**:741–9. doi:10.1161/HYPERTENSIONAHA.110.157297

4 Odibo AO, Goetzinger KR, Odibo L, *et al.* First-trimester prediction of preeclampsia using metabolomic biomarkers: a discovery phase study. *Prenat Diagn* 2011;**31**:990–4.

5 Woodham PC, Brittain JE, Baker AM, *et al.* Midgestation maternal serum 25-hydroxyvitamin D level and soluble fms-like tyrosine kinase 1/placental growth factor ratio as predictors of severe preeclampsia. *Hypertension* 2011;**58**:1120–5. doi:10.1161/HYPERTENSIONAHA.111.179069

6 Rijvers CAH, Marzano S, Winkens B, *et al.* Early-pregnancy asymmetric dimethylarginine (ADMA) levels in women prone to develop recurrent hypertension. *Pregnancy Hypertens* 2013;**3**:118–23. doi:10.1016/j.preghy.2013.01.001

7 Khalil AA, Tsikas D, Akolekar R, *et al.* Asymmetric dimethylarginine, arginine and homoarginine at 11-13 weeks gestation and preeclampsia: A case-control study. *J Hum Hypertens* 2013;**27**:38–43. doi:10.1038/jhh.2011.109

8 Diaz SO, Barros AS, Goodfellow BJ, *et al.* Second trimester maternal urine for the diagnosis of trisomy 21 and prediction of poor pregnancy outcomes. *J Proteome Res* 2013;**12**:2946–57. doi:10.1021/pr4002355

9 Kenny LC, Broadhurst DI, Hong W, *et al.* NMR Profiling in Early Pregnancy Reveals a Metabolomic Signature the Predicts the Subsequent Development Preeclampsia. *Reprod Sci* 2013;**20**:2641A. doi:10.1177/1933719113482088

10 Kuc S, Koster MPH, Pennings JLA, *et al.* Metabolomics profiling for identification of novel potential markers in early prediction of preeclampsia. *PLoS One* 2014;**9**. doi:10.1371/journal.pone.0098540

11 Wetta LA, Biggio JR, Cliver S, *et al.* Is midtrimester vitamin D status associated with spontaneous preterm birth and preeclampsia? *Am J Perinatol* 2014;**31**:541–6. doi:10.1055/s-0033-1356483

For peer review only - http://bmjopen.bmj.com/site/about/guidelines.xhtml

12 Bahado-Singh RO, Syngelaki A, Akolekar R, *et al.* Validation of metabolomic models for prediction of early-onset preeclampsia. *Am J Obstet Gynecol* 2015;**213**:530.e1-530.e10. doi:10.1016/j.ajog.2015.06.044

13 Eichelberger KY, Baker AM, Woodham PC, *et al.* Second-trimester maternal serum paraxanthine, CYP1A2 activity, and the risk of severe preeclampsia. *Obstet Gynecol* 2015;**126**:725–30. doi:10.1097/AOG.0000000000001041

14 Koster MPH, Vreeken RJ, Harms AC, *et al.* First-Trimester serum acylcarnitine levels to predict preeclampsia: A metabolomics approach. *Dis Markers* 2015;**2015**. doi:10.1155/2015/857108

15 Bilodeau JF, Qin Wei S, Larose J, *et al.* Plasma F2-isoprostane class VI isomers at 12-18 weeks of pregnancy are associated with later occurrence of preeclampsia. *Free Radic Biol Med* 2015;**85**:282–7. doi:10.1016/j.freeradbiomed.2015.05.012

16 Ates S, Sevket O, Ozcan P, *et al.* Vitamin D status in the first-trimester: Effects of vitamin D deficiency on pregnancy outcomes. *Afr Health Sci* 2016;**16**:36–43. doi:10.4314/ahs.v16i1.5

17 Cantonwine DE, Meeker JD, Ferguson KK, *et al.* Urinary concentrations of bisphenol A and phthalate metabolites measured during pregnancy and risk of preeclampsia. *Environ Health Perspect* 2016;**124**:1651–5. doi:10.1289/EHP188

18 Kiely ME, Zhang JY, Kinsella M, *et al.* Vitamin D status is associated with uteroplacental dysfunction indicated by pre-eclampsia and small-for-gestational-age birth in a large prospective pregnancy cohort in Ireland with low Vitamin D status. *Am J Clin Nutr* 2016;**104**:354–61. doi:10.3945/ajcn.116.130419

19 Bahado-Singh RO, Syngelaki A, Mandal R, *et al.* Metabolomic determination of pathogenesis of late-onset preeclampsia. *J Matern Neonatal Med* 2017;**30**:658–64. doi:10.1080/14767058.2016.1185411

20 Bahado-Singh R, Poon LC, Yilmaz A, *et al.* Integrated Proteomic and Metabolomic prediction of Term Preeclampsia. *Sci Rep* 2017;**7**:1–10. doi:10.1038/s41598-017-15882-9

21 Dobierzewska A, Soman S, Illanes SE, *et al.* Plasma cross-gestational sphingolipidomic analyses reveal potential first trimester biomarkers of preeclampsia. *PLoS One* 2017;**12**:1–16. doi:10.1371/journal.pone.0175118

22 Ye Y, Zhou Q, Feng L, *et al.* Maternal serum bisphenol A levels and risk of pre-eclampsia: A nested case-control study. *Eur J Public Health* 2017;**27**:1102–7. doi:10.1093/eurpub/ckx148

23 Gong S, Sovio U, Aye ILMH, *et al.* Placental polyamine metabolism differs by fetal sex, fetal growth restriction, and preeclampsia. *JCI Insight* 2018;**3**:1–15.

For peer review only

For peer review only - http://bmjopen.bmj.com/site/about/guidelines.xhtml

doi:10.1172/jci.insight.120723
24 Tamblyn JA, Jenkinson C, Larner DP, *et al.* Serum and urine vitamin D metabolite analysis in early preeclampsia. *Endocr Connect* 2018;**7**:199–210. doi:10.1530/EC-17-0308
25 Sovio U, McBride N, Wood AM, *et al.* 4-Hydroxyglutamate is a novel predictor of pre-eclampsia. *Int J Epidemiol* 2020;**49**:301–11. doi:10.1093/ije/dyz098
26 Huo X, Huang R, Gan Y, *et al.* Perfluoroalkyl substances in early pregnancy and risk of hypertensivedisorders of pregnancy: A prospective cohort study. *Environ Int* 2020;**138**. doi:10.1016/j.envint.2020.105656
27 Rylander L, Lindh CH, Hansson SR, *et al.* Per- and Polyfluoroalkyl Substances in Early Pregnancy and Risk for Preeclampsia: A Case-Control Study in Southern Sweden. *Toxics* 2020;**8**. doi:10.3390/toxics8020043
28 Lee SM, Kang Y, Lee EM, *et al.* Metabolomic biomarkers in midtrimester maternal plasma can accurately predict the development of preeclampsia. *Sci Rep* 2020;**10**:16142. doi:10.1038/s41598-020-72852-4
29 Kenny LC, Thomas G, Poston L, *et al.* Prediction of preeclampsia risk in first time pregnant women: Metabolite biomarkers for a clinical test. *PLoS One* 2020;**15**:1–19. doi:10.1371/journal.pone.0244369
30 Shanmugalingam R, Wang X, Motum P, *et al.* The 15-Epilipoxin-A4 Pathway with Prophylactic Aspirin in PreventingPreeclampsia: A Longitudinal Cohort Study. *J Clin Endocrinol Metab* 2020;**105**. doi:10.1210/clinem/dgaa642
31 Harville EW, Li Y-YY-Y, Pan K, *et al.* Untargeted analysis of first trimester serum to reveal biomarkers of pregnancy complications: a case-control discovery phase study. *Sci Rep* 2021;**11**:3468. doi:10.1038/s41598-021-82804-1
32 Huang Q, Hao S, You J, *et al.* Early-pregnancy prediction of risk for pre-eclampsia using maternal blood leptin/ceramide ratio: Discovery and confirmation. *BMJ Open* 2021;**11**. doi:10.1136/bmjopen-2021-050963

For peer review only - http://bmjopen.bmj.com/site/about/guidelines.xhtml

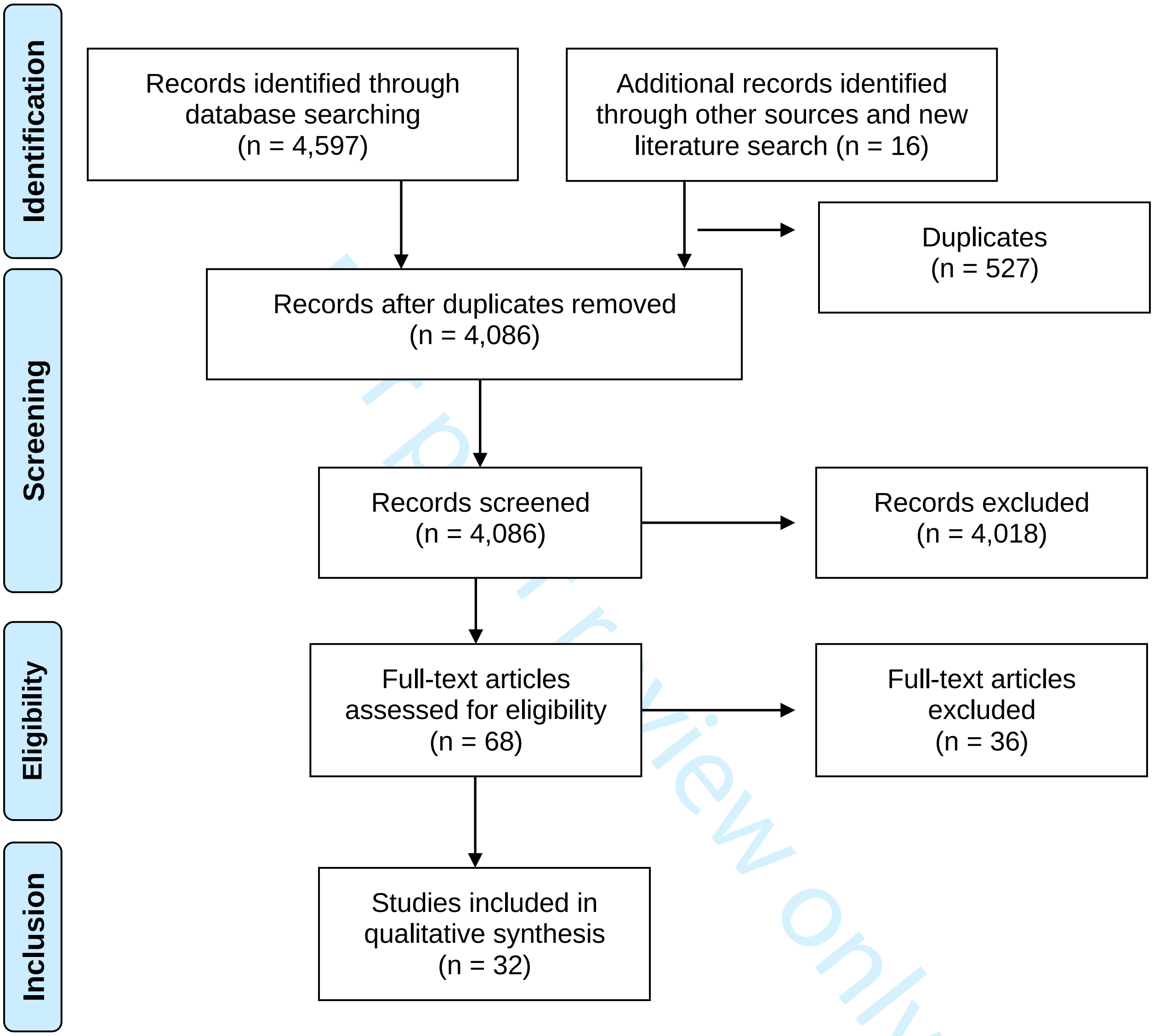

From: Moher D, Liberati A, Tetzlaff J, Altman DG, The PRISMA Group (2009). *P*referred *R*eporting *I*tems for *S*ystematic Reviews and *M*eta-*A*nalyses: The PRISMA Statement. PLoS Med 6(7): e1000097. doi:10.1371/journal.pmed1000097

For more information, visit www.prisma-statement.org.

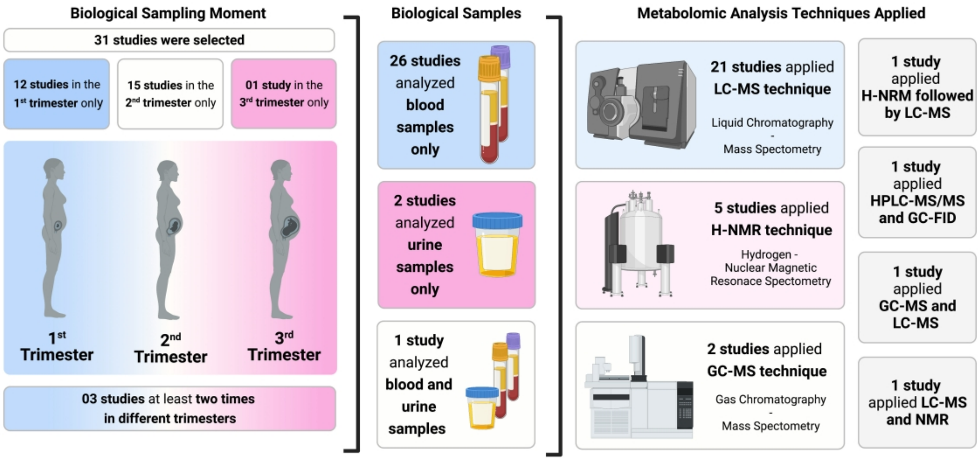

Figure 2

419x199mm (300 x 300 DPI)

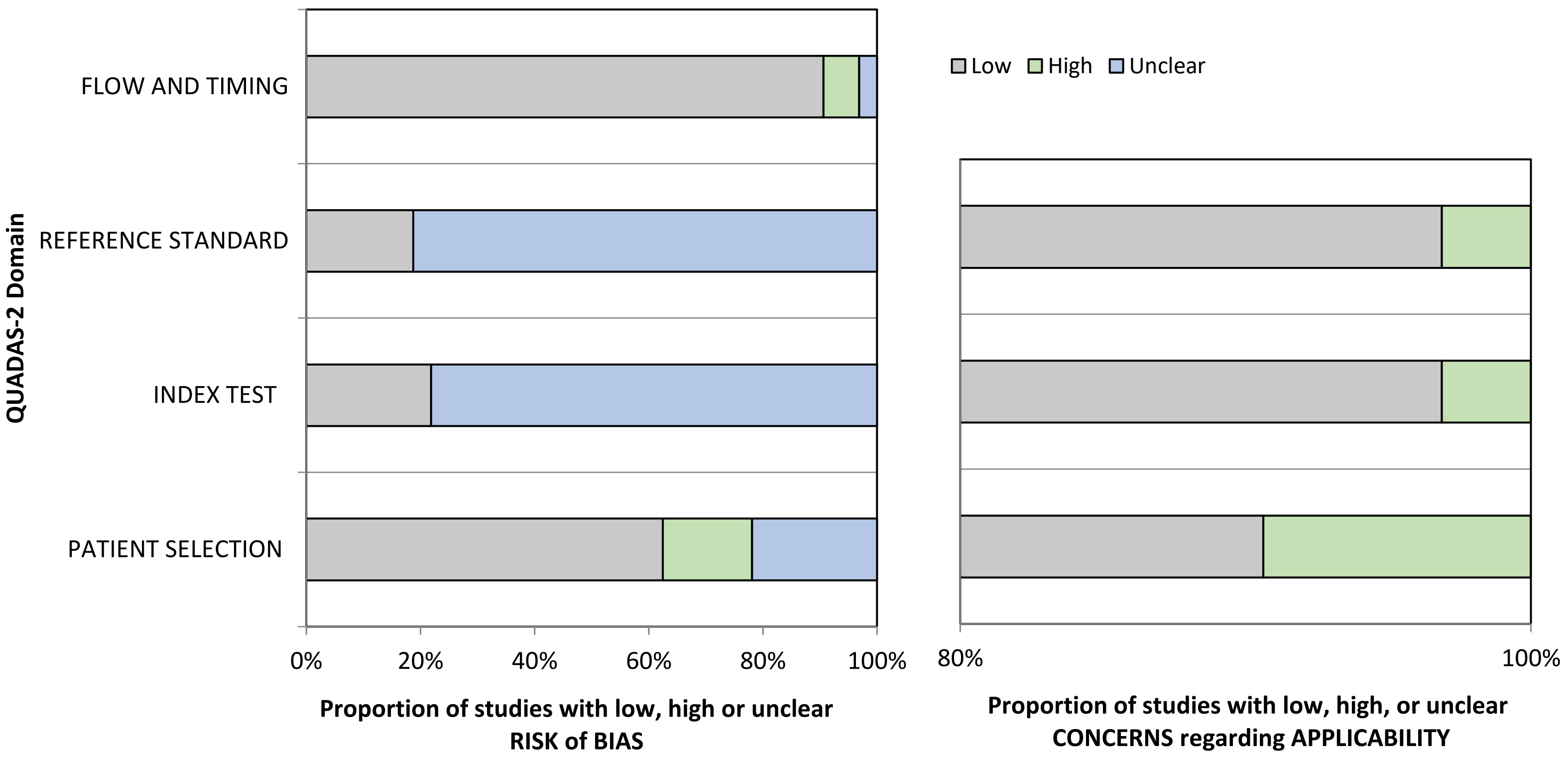

For peer review only - http://bmjopen.bmj.com/site/about/guidelines.xhtml

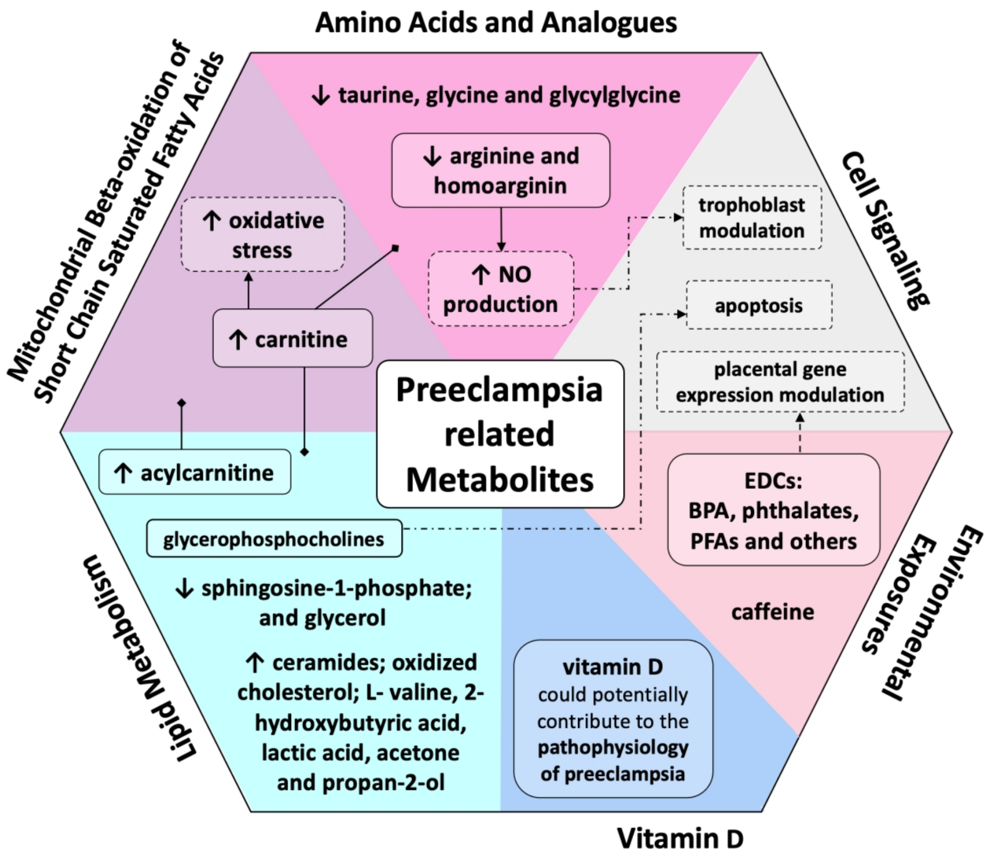

Figure 4

355x313mm (144 x 144 DPI)

Prediction of pregnancy-related hypertensive disorders using metabolomics: a systematic review

Jussara Mayrink, Debora Farias B. Leite, Guilherme M Nóbrega, Maria Laura Costa, José Guilherme Cecatti

Supplementary material 1 – PRISMA checklist

Section/topic	#	Checklist item	Section where the item was reported
**Title**			
Title	1	Identify the report as a systematic review, meta-analysis, or both.	Page 1/title
**ABSTRACT**			
Structured summary	2	Provide a structured summary including, as applicable: background; objectives; data sources; study eligibility criteria, participants, and interventions; study appraisal and synthesis methods; results; limitations; conclusions and implications of key findings; systematic review registration number.	Page 2/abstract
**INTRODUCTION**			
Rationale	3	Describe the rationale for the review in the context of what is already known.	Page 4
Objectives	4	Provide an explicit statement of questions being addressed with reference to participants, interventions, comparisons, outcomes, and study design (PICOS).	Page 5; Lines 11-12
**METHODS**			
Protocol and registration	5	Indicate if a review protocol exists, if and where it can be accessed (e.g., Web address), and, if available, provide registration information including registration number.	Page 3, Line 3<br>Page 5, Line 15
Eligibility criteria	6	Specify study characteristics (e.g., PICOS, length of follow-up) and report characteristics (e.g., years considered, language, publication status) used as criteria for eligibility, giving rationale.	Page 5, Lines 20-28<br>Page 6, Lines 1-13<br>Page 7, Lines 6-13
Information sources	7	Describe all information sources (e.g., databases with dates of coverage, contact with study authors to identify additional studies) in the search and date last searched.	Page 6, Lines 14-23<br>Page 8, Lines 10-11
Search	8	Present full electronic search strategy for at least one database, including any limits used, such that it could be repeated.	Supplementary Material 2
Study selection	9	State the process for selecting studies (i.e., screening, eligibility, included in systematic review, and, if applicable, included in the meta-analysis).	Page 7, Lines 16-27<br>Page 8, Lines 1-7
Data collection process	10	Describe method of data extraction from reports (e.g., piloted forms, independently, in duplicate) and any processes for obtaining and confirming data from investigators.	Page 7, Lines 16-27<br>Page 8, Lines 1-7
Data items	11	List and define all variables for which data were sought (e.g., PICOS, funding sources) and any assumptions and simplifications made.	Page 7, Lines 16-27<br>Page 8, Lines 1-7
Risk of bias in individual studies	12	Describe methods used for assessing risk of bias of individual studies (including specification of whether this was done at the study or outcome level), and how this information is to be used in any data synthesis.	Page 7, Lines 16-20
Summary measures	13	State the principal summary measures (e.g., risk ratio, difference in means).	Page 8, Lines 3-7

For peer review only

Section/topic	#	Checklist item	Section where the item was reported
Synthesis of results	14	Describe the methods of handling data and combining results of studies, if done, including measures of consistency (e.g., I^2) for each meta-analysis.	NA
Risk of bias across studies	15	Specify any assessment of risk of bias that may affect the cumulative evidence (e.g., publication bias, selective reporting within studies).	NA
Additional analyses	16	Describe methods of additional analyses (e.g., sensitivity or subgroup analyses, meta-regression), if done, indicating which were pre-specified.	NA
RESULTS			
Study selection	17	Give numbers of studies screened, assessed for eligibility, and included in the review, with reasons for exclusions at each stage, ideally with a flow diagram.	Page 8, Lines 11-27
Study characteristics	18	For each study, present characteristics for which data were extracted (e.g., study size, PICOS, follow-up period) and provide the citations.	Page 11, Line 11 – Page 15, Line 5
Risk of bias within studies	19	Present data on risk of bias of each study and, if available, any outcome level assessment (see item 12).	Page 15, Lines 6-15
Results of individual studies	20	For all outcomes considered (benefits or harms), present, for each study: (a) simple summary data for each intervention group (b) effect estimates and confidence intervals, ideally with a forest plot.	NA
Synthesis of results	21	Present results of each meta-analysis done, including confidence intervals and measures of consistency.	NA
Risk of bias across studies	22	Present results of any assessment of risk of bias across studies (see Item 15).	NA
Additional analysis	23	Give results of additional analyses, if done (e.g., sensitivity or subgroup analyses, meta-regression [see Item 16]).	NA
**DISCUSSION**			
Summary of evidence	24	Summarize the main findings including the strength of evidence for each main outcome; consider their relevance to key groups (e.g., healthcare providers, users, and policy makers).	Page 16, Line 6 – Page 19, Line 30
Limitations	25	Discuss limitations at study and outcome level (e.g., risk of bias), and at review-level (e.g., incomplete retrieval of identified research, reporting bias).	Page 20, Lines 15-28
Conclusions	26	Provide a general interpretation of the results in the context of other evidence, and implications for future research.	Page 21, Lines 1-20
**FUNDING**			
Funding	27	Describe sources of funding for the systematic review and other support (e.g., supply of data); role of funders for the systematic review.	Page 29, Lines 8-15

For peer review only

Supplementary material 2 – Full search strategy for each database.

PUBMED

((((((((((((preeclampsia) OR (pre-eclampsia)) OR (eclampsia)) OR (gestational hypertension)) OR (white coat hypertension)) OR (severe preeclampsia)) OR (late preeclampsia)) OR (early preeclampsia)) OR (pregnancy-induced hypertension)) OR (hypertensive syndromes of pregnancy)) AND ((((((((((((((((metabolomic*) OR (metabonomic*)) OR (metabolit*)) OR (HNMR)) OR (proton NMR)) OR (proton nuclear magnetic resonance)) OR (liquid chromatogra*)) OR (gas chromatogra*)) OR (UPLC)) OR (HPLC)) OR (high pressure liquid chromatograph*)) OR (ultra-performance liquid chromatograph*)) OR (ultra performance liquid chromatograph*)) OR (lipidomic*)) OR (mass spectrometr*))) AND (((screen*) OR (predict*)) OR (profil*)))

EMBASE

('preeclampsia' OR 'pre-eclampsia' OR 'eclampsia' OR 'gestational hypertension' OR 'white coat hypertension' OR 'severe preeclampsia' OR 'late preeclampsia' OR 'early preeclampsia' OR 'pregnancy-induced hypertension' OR 'hypertensive syndromes of pregnancy') AND ('metabolomic*' OR 'metabonomic*' OR 'metabolit*' OR 'HNMR' OR 'proton NMR' OR 'proton nuclear magnetic resonance' OR 'liquid chromatogra*' OR 'gas chromatogra*' OR 'UPLC' OR 'HPLC' OR 'high pressure liquid chromatograph*' OR 'ultra-performance liquid chromatograph*' OR 'ultra performance liquid chromatograph*' OR 'lipidomic*' OR 'mass spectrometr*') AND ('screen*' OR 'predict*' OR 'profil*').

SCOPUS

((TITLE-ABS-KEY (screen*) OR TITLE-ABS-KEY (predict*) OR TITLE-ABS-KEY (profil*))) AND ((TITLE-ABS-KEY (preeclampsia) OR TITLE-ABS-KEY (pre-eclampsia) OR TITLE-ABS-KEY (eclampsia) OR TITLE-ABS-KEY (gestational AND hypertension) OR TITLE-ABS-KEY (white AND coat AND hypertension) OR TITLE-ABS-KEY (severe AND preeclampsia) OR TITLE-ABS-KEY (late AND preeclampsia) OR TITLE-ABS-KEY (early AND preeclampsia) OR TITLE-ABS-KEY (pregnancy- AND induced AND hypertension) OR TITLE-ABS-KEY (hypertensive AND syndromes AND of AND pregnancy))) AND ((TITLE-ABS-KEY (metabolomic) OR TITLE-ABS-KEY (metabonomic*) OR TITLE-ABS-KEY (metabolit*) OR TITLE-ABS-KEY (hnmr) OR TITLE-ABS-KEY (proton AND nmr) OR TITLE-ABS-KEY (proton AND nuclear AND magnetic AND resonance) OR TITLE-ABS-KEY (liquid AND chromatogra*) OR TITLE-ABS-KEY (gas AND chromatogra*) OR TITLE-ABS-KEY (uplc) OR TITLE-ABS-KEY (hplc) OR TITLE-ABS-KEY (high AND pressure AND liquid AND chromatograph*) OR TITLE-ABS-KEY (ultra-performance AND liquid AND chromatograph*) OR TITLE-ABS-KEY (ultra AND performance AND liquid AND chromatograph*) OR TITLE-ABS-KEY (lipidomic*) OR TITLE-ABS-KEY (mass AND spectrometr*)))

Web of Knowledge

#1

For peer review only

((((((((ALL=(preeclampsia)) OR ALL=(pre-eclampsia)) OR ALL=(eclampsia)) OR ALL=(gestational hypertension)) OR ALL=(white coat hypertension)) OR ALL=(severe preeclampsia)) OR ALL=(late preeclampsia)) OR ALL=(early preeclampsia)) OR ALL=(pregnancy- induced hypertension)) OR ALL=(hypertensive syndromes of pregnancy)

#2

(((((((((((((ALL=(metabolomic*)) OR ALL=(metabonomic*)) OR ALL=(metabolit*)) OR ALL=(HNMR)) OR ALL=(proton NMR)) OR ALL=(proton nuclear magnetic resonance)) OR ALL=(liquid chromatogra*)) OR ALL=(gas chromatogra*)) OR ALL=(UPLC)) OR ALL=(HPLC)) OR ALL=(high pressure liquid chromatograph*)) OR ALL=(ultra-performance liquid chromatograph*)) OR ALL=(ultra performance liquid chromatograph*)) OR ALL=(lipidomic*)) OR ALL=(mass spectrometr*)

#3

((ALL=(screen*)) OR ALL=(predict*)) OR ALL=(profil*)

#4

#1 AND #2 AND #3

LILACS

(‘preeclampsia’ OR ‘pre-eclampsia’ OR ‘eclampsia’ OR ‘gestational hypertension’ OR ‘white coat hypertension’ OR ‘severe preeclampsia’ OR ‘late preeclampsia’ OR ‘early preeclampsia’ OR ‘pregnancy-induced hypertension’ OR ‘hypertensive syndromes of pregnancy’) AND (‘metabolomic*’ OR ‘metabonomic*’ OR ‘metabolit*’ OR ‘HNMR’ OR ‘proton NMR’ OR ‘proton nuclear magnetic resonance’ OR ‘liquid chromatogra*’ OR ‘gas chromatogra*’ OR ‘UPLC’ OR ‘HPLC’ OR ‘high pressure liquid chromatograph*’ OR ‘ultra-performance liquid chromatograph*’ OR ‘ultra performance liquid chromatograph*’ OR ‘lipidomic*’ OR ‘mass spectrometr*’) AND (‘screen*’ OR ‘predict*’ OR ‘profil*’)

SciELO

(‘preeclampsia’ OR ‘pre-eclampsia’ OR ‘eclampsia’ OR ‘gestational hypertension’ OR ‘white coat hypertension’ OR ‘severe preeclampsia’ OR ‘late preeclampsia’ OR ‘early preeclampsia’ OR ‘pregnancy-induced hypertension’ OR ‘hypertensive syndromes of pregnancy’) AND (‘metabolomic*’ OR ‘metabonomic*’ OR ‘metabolit*’ OR ‘HNMR’ OR ‘proton NMR’ OR ‘proton nuclear magnetic resonance’ OR ‘liquid chromatogra*’ OR ‘gas chromatogra*’ OR ‘UPLC’ OR ‘HPLC’ OR ‘high pressure liquid chromatograph*’ OR ‘ultra-performance liquid chromatograph*’ OR ‘ultra performance liquid chromatograph*’ OR ‘lipidomic*’ OR ‘mass spectrometr*’) AND (‘screen*’ OR ‘predict*’ OR ‘profil*’)

DARE and HTA

(‘preeclampsia’ OR ‘pre-eclampsia’ OR ‘eclampsia’ OR ‘gestational hypertension’ OR ‘white coat hypertension’ OR ‘severe preeclampsia’ OR ‘late preeclampsia’ OR ‘early preeclampsia’ OR ‘pregnancy-induced hypertension’ OR ‘hypertensive syndromes of pregnancy’) AND (‘metabolomic*’ OR ‘metabonomic*’ OR ‘metabolit*’ OR ‘HNMR’ OR ‘proton NMR’ OR ‘proton nuclear magnetic resonance’ OR ‘liquid chromatogra*’ OR ‘gas chromatogra*’ OR ‘UPLC’ OR ‘HPLC’ OR ‘high pressure liquid chromatograph*’

For peer review only

OR ‘ultra-performance liquid chromatograph*’ OR ‘ultra performance liquid chromatograph*’ OR ‘lipidomic*’ OR ‘mass spectrometr*’) AND (‘screen*’ OR ‘predict*’ OR ‘profil*’) IN DARE, HTA

For peer review only

Supplementary Material 3 - Excluded studies and reasons for exclusion.

Authors	DOI	Reason for exclusion
Kenny LC et al, 2008	10.1177/1933719108316908	Cross-sectional design.
Aris A et al, 2009	10.1016/j.placenta.2009.01.003	Non metabolomics research.
Kenny LC et al, 2009		Conference abstract. Duplicated data. Please check Kenny LC et al (10.1161/HYPERTENSIONAHA.110.157297)
Baker AM et al, 2010	10.1210/jc.2010-0996	Duplicated data. Please check Woodham PC et al, 2011 (10.1161/HYPERTENSIONAHA.111.179069)
Bahado-Singh RO et al, 2012	10.3109/14767058.2012.680254	Duplicated data. Please check Bahado-Singh RO et al, 2015 (10.1016/j.ajog.2015.06.044)
Austdal M et al, 2013	10.1016/j.preghy.2013.04.030	Cross-sectional design.
Bahado-Singh RO et al, 2013	10.1016/j.ajog.2012.11.003	Duplicated data. Please check Bahado-Singh RO et al, 2017 (10.1080/14767058.2016.1185411)
Senyavina NV et al, 2013	10.1007/s10517-013-2225-y	Cross-sectional design.
Bodnar et al, 2014	10.1097/EDE.0000000000000039	Imputation of data obtained in a small subset to the whole sample.
Austdal M et al, 2014	10.1371/journal.pone.0091923	Duplicated data. Please check Austdal M et al, 2015 (10.3390/ijms160921520)
Austdal M et al, 2015	10.1016/j.placenta.2015.10.019	Cross-sectional design.
Austdal M et al, 2015	10.3390/ijms160921520	Comparison of preeclampsia patients with women with normal blood pressure and gestational hypertension together.
Riley J et al, 2016		Conference abstract. Duplicated data. Please check Odibo A et al 2011 (10.1002/pd.2822)
Chen T et al, 2017	10.1016/j.bbrc.2017.02.032	Cross-sectional design.
Kelly RS et al, 2017	10.1007/s11306-016-1149-8	Randomized clinical trial.
Powell KL et al, 2018	10.1016/j.placenta.2018.04.005	Cross-sectional design.
Naase et al, 2018	10.1093/humrep/33.Supplement_1.1	Only mass spectrometry data.
Kawasaki K et al, 2019	10.1161/HYPERTENSIONAHA.118.12389	Cross-sectional design.
Liu Y et al, 2019	10.1111/1440-1681.13095	Cross-sectional design.
Sander KN et al, 2019	10.1007/s11306-019-1600-8	Cross-sectional design.
Chaudhry SH et al, 2019	10.1186/s12884-019-2219-5	Non metabolomics research.
Lewandowska M et al, 2019	10.3390/nu11051028	No specific data on preeclampsia.

For peer review only

Vieira MC et al, 2019	10.1177/1933719119834079	Randomized clinical trial. Only mass spectrometry data.
Odenkirk MT et al, 2020	10.1039/x0xx00000x	Cross-sectional design.
Ferranti et al, 2020	10.1155/2020/1515321	Mention of altered metabolic pathways, but not metabolites.
Kelly C et al, 2020	10.3390/nu12072048	Non metabolomics research.
Kyozuka H et al, 2020	10.1038/s41598-020-70974-3	No specific data on preeclampsia.
McBride N et al, 2020	10.1186/s12916-020-01819-z	Randomized clinical trial.
Jääskeläinen T et al, 2021	10.1007/s11306-020-01752-5	No specific data on preeclampsia compared to normotensive women.
Rodriguez et al, 2021	10.1016/j.arcmed.2021.01.012	Cross-sectional design.
McBride N et al, 2021	10.3390/ metabo11080530	Mention of accuracy measures of a group of metabolites, and of metabolites combined with other maternal risk factors, but do not mention specific metabolites.
Kivelä J et al, 2021	10.1210/clinem/dgab475	Randomized clinical trial.
Zhang-Rutledge K et al, 2021	10.1016/j.ajog.2020.12.267	Only mass spectrometry data.
Wahab et al, 2022	10.3390/ metabo12010013	No specific data on preeclampsia compared to normotensive women.
Herlambang H et al, 2022	10.1186/s12884-021-04313-3	Cross-sectional design.
Liu H, et al	10.1007/s12011-021-02988-5	Cross-sectional design.

For peer review only

Supplementary Material 4 - Predictive metabolites of hypertensive disorders of pregnancy summarized according to their superclass, chemical class, subclass, and metabolic pathways.

**Predictive metabolites**	**Chemical class**	**Chemical subclass**	**Metabolic pathway or process**	**Involved with**
		Organic acids and derivatives		
N1, N12-diacetylspermine	Carboximidic acids and derivatives	Carboximidic acids	NA	Late onset preeclampsia[1]
Acetamide	Carboximidic acids and derivatives	Carboximidic acids	NA	Preeclampsia [2]
Acetate	Carboxylic acids and derivatives	Carboxylic acids	Glycolysis, gluconeogenesis; pyruvate, propanoate, taurine and hypotaurine metabolism	Preeclampsia [2,3]
3-methylhistidine	Carboxylic acids and derivatives	Amino acids, peptides, and analogues	Beta-alanine, histidine and methylhistidine metabolism	Preeclampsia [3]
Methylhistidine	Carboxylic acids and derivatives	Amino acids, peptides, and analogues	Histidine Metabolism	Late onset preeclampsia [4]
Alanine	Carboxylic acids and derivatives	Amino acids, peptides, and analogues	Glutathione metabolism, glycine and serine metabolism, tryptophan metabolism, urea cycle, glutamate metabolism, glucose-alanine cycle	Preeclampsia [5], early onset [5] preeclampsia
Betaine	Carboxylic acids and derivatives	Amino acids, peptides, and analogues	Betaine metabolism, glycine and serine metabolism, methionine metabolism	Preeclampsia [2]
Asymmetric dimethylarginine	Carboxylic acids and derivatives	Amino acids, peptides, and analogues	NA	Preeclampsia [6], early onset [7] preeclampsia
L-arginine	Carboxylic acids and derivatives	Amino acids, peptides, and analogues	Urea cycle, arginine and proline metabolism, glycine and serine metabolism, transcription/translation	Preeclampsia [6], early onset [7] preeclampsia
L-homoarginine	Carboxylic acids and derivatives	Amino acids, peptides, and analogues	NA	Early onset [7] preeclampsia

For peer review only

Creatinine	Carboxylic acids and derivatives	Amino acids, peptides, and analogues	Arginine and proline metabolism	Preeclampsia [2,3]
Glutamine	Carboxylic acids and derivatives	Amino acids, peptides, and analogues	Pyrimidine metabolism, glutamate metabolism, purine metabolism, transcription/translation, urea cycle	Preeclampsia [2,3]
Glutamate	Carboxylic acids and derivatives	Amino acids, peptides, and analogues	Alanine metabolism, histidine metabolism, ammonia recycling, cysteine metabolism, arachidonic acid metabolism	Preeclampsia [5], early onset [5] preeclampsia
4-hydroxyglutamate	Carboxylic acids and derivatives	Amino acids, peptides, and analogues	Arginine and proline metabolism	Preeclampsia [8]
Glycine	Carboxylic acids and derivatives	Amino acids, peptides, and analogues	Ammonia recycling, porphyrin metabolism, glutathione metabolism, glycine and serine metabolism	Preeclampsia [2]
Glycylglycine	Carboxylic acids and derivatives	Amino acids, peptides, and analogues	NA	Late onset [9] preeclampsia
Isoleucine	Carboxylic acids and derivatives	Amino acids, peptides, and analogues	Valine, leucine and isoleucine biosynthesis and degradation	Preeclampsia [2,3]
Asparagine	Carboxylic acids and derivatives	Amino acids, peptides, and analogues	Alanine, aspartate and glutamate metabolism; protein digestion and absorption	Preeclampsia [10]
Methionine	Carboxylic acids and derivatives	Amino acids, peptides, and analogues	Betaine metabolism, glycine and serine metabolism; spermidine and spermine biosynthesis	Preeclampsia [2]
N,N-Dimethylglycine	Carboxylic acids and derivatives	Amino acids, peptides, and analogues	Glycine, serine, and threonine metabolism	Preeclampsia [10]
Ornithine	Carboxylic acids and derivatives	Amino acids, peptides, and analogues	Urea cycle, arginine and proline metabolism; glycine and serine	Preeclampsia [2]

For peer review only

			metabolism; Spermidine and Spermine Biosynthesis	
Phenylacetylglutamine	Carboxylic acids and derivatives	Amino acids, peptides, and analogues	Phenylacetate metabolism, phenylalanine metabolism	Preeclampsia [3]
Phenylalanine	Carboxylic acids and derivatives	Amino acids, peptides, and analogues	Phenylalanine, tyrosine and tryptophan biosynthesis; phenylalanine metabolismo;	Preeclampsia [5], early onset [5] preeclampsia
Proline	Carboxylic acids and derivatives	Amino acids, peptides, and analogues	Arginine and proline metabolism	Preeclampsia [2]
Tyrosine	Carboxylic acids and derivatives	Amino acids, peptides, and analogues	Tyrosine metabolism, phenylalanine and tyrosine metabolism, catecholamine biosynthesis, transcription/translation	Preeclampsia [3]
Valine	Carboxylic acids and derivatives	Amino acids, peptides, and analogues	Valine, leucine and isoleucine biosynthesis and degradation; propanoate metabolism	Preeclampsia [2]
Formate	Carboxylic acids and derivatives	Carboxylic acids	Folate metabolism, pterine biosynthesis	Preeclampsia [3]
Fumarate	Carboxylic acids and derivatives	Dicarboxylic acids and derivatives	Phenylalanine and tyrosine metabolism, arginine and proline metabolism, aspartate metabolism, urea cycle, mitochondrial electron transport chain	Preeclampsia [3]
Succinate	Carboxylic acids and derivatives	Dicarboxylic acids and derivatives	Citrate cycle, oxidative phosphorylation; alanine, aspartate and glutamate metabolism; pyruvate metabolism; cAMP signalling pathway; GABAergic synapse; glucagon signalling pathway	Preeclampsia [3]
Cis-aconitate	Carboxylic acids and derivatives	Tricarboxylic acids and derivatives	Citric acid cycle	Preeclampsia [3]

Citrate	Carboxylic acids and derivatives	Tricarboxylic acids and derivatives	Citric acid cycle, transfer of acetyl groups into mitochondria	Preeclampsia [3], early onset [11] preeclampsia, late onset [4] preeclampsia
2-hydroxybutyrate	Hydroxy acids and derivatives	Alpha hydroxy acids and derivatives	Propanoate metabolism	Early onset [11] preeclampsia
2-ketoglutarate	Keto acids and derivatives	Gamma-keto acids and derivatives	Citric acid cycle, gluconeogenesis, oxidation of branched-chain fatty acids, urea cycle, branched-chain amino acids degradation	Preeclampsia [3]
2-oxovaleric acid	Keto acids and derivatives	Short-chain keto acids and derivatives	NA	Late onset [12] preeclampsia
Acetoacetic acid	Keto acids and derivatives	Short-chain keto acids and derivatives	Ketone body metabolism, fatty acid biosynthesis, branched-chain amino acids degradation, tyrosine metabolism, phenylalanine and tyrosine metabolism	Late onset [12] preeclampsia
Oxo-methylbutanoic acid	Keto acids and derivatives	Short-chain keto acids and derivatives	Cell signaling; valine, leucine and isoleucine Degradation	Late onset [12] preeclampsia
Pyruvate	Keto acids and derivatives	Alpha keto acids and derivatives	Urea Cycle, glucose-alanine cycle, glycine and serine Metabolism, pyruvate metabolism, alanine metabolism	Late onset [13] preeclampsia
Urea	Organic carbonic acids and derivatives	Ureas	Urea cycle; D-arginine and D-ornithine metabolism; arginine and proline metabolism	Late onset [4] preeclampsia
Taurine	Organic sulfonic acids and derivatives	Organosulfonic acids and derivatives	Taurine and hypotaurine metabolism	Early onset [9] preeclampsia
Indoxyl sulphate	Organic sulfuric acids and derivatives	Arylsulfates	NA	Preeclampsia [3]
p-cresol sulphate	Organic sulfuric acids and derivatives	Arylsulfates	NA	Preeclampsia [3]

		Lipids and lipid-likes molecules		
3-hydroxyisovalerate	Fatty acyls	Fatty acids and conjugates	Lipid transport, metabolism, and peroxidation; fatty acid metabolism	Early onset [11] preeclampsia
Adipic acid	Fatty acyls	Fatty acids and conjugates	Lipid transport, metabolism, and peroxidation; fatty acid metabolism	Late onset [12] preeclampsia
Docosapentaenoic acid	Fatty acyls	Fatty acids and conjugates	Lipid transport, metabolism, and peroxidation; fatty acid metabolism; cell signalling; alpha linolenic acid and linoleic acid metabolism	Preeclampsia [14]
Docosahexaenoic acid	Fatty acyls	Fatty acids and conjugates	Lipid transport, metabolism, and peroxidation; fatty acid metabolism; cell signalling; alpha linolenic acid and linoleic acid metabolism	Preeclampsia [14], Late onset [12] preeclampsia
Docosatriynoic acid	Fatty acyls	Fatty acids and conjugates	Lipid transport, metabolism, and peroxidation; fatty acid metabolism; cell signalling	Late onset [12] preeclampsia
Eicosatrienoic acid	Fatty acyls	Fatty acids and conjugates	Lipid transport, metabolism, and peroxidation; fatty acid metabolism; cell signalling	Preeclampsia [14]
Eicosatetraenoic acid	Fatty acyls	Fatty acids and conjugates	Lipid transport, metabolism, and peroxidation; fatty acid metabolism; cell signalling; alpha linolenic acid and linoleic acid metabolism.	Preeclampsia [14]
Eicosapentaenoic acid	Fatty acyls	Fatty acids and conjugates	Lipid transport, metabolism, and peroxidation; fatty acid metabolism; cell	Preeclampsia [14]

For peer review only

			signalling; alpha linolenic acid and linoleic acid metabolism.	
Methylglutaric acid	Fatty acyls	Fatty acid and conjugates	Lipid transport, metabolism, and peroxidation; fatty acid metabolism; cell signalling	Late onset [12] preeclampsia
Oleic acid	Fatty acyls	Fatty acids and conjugates	Fatty acid biosynthesis, biosynthesis of unsaturated fatty acids	Late onset [12] preeclampsia
Decanoylcarnitine	Fatty acyls	Fatty acid esters	Lipid transport and peroxidation; fatty acid metabolism; cell signaling	Early onset [15] preeclampsia, late onset [12] preeclampsia
Decenoylcarnitine	Fatty acyls	Fatty acid esters	NA	Early onset [15] preeclampsia, late onset preeclampsia
Lauroylcarnitine	Fatty acyls	Fatty acid esters	Lipid transport, metabolism, and peroxidation; fatty acid metabolism; cell signalling	Early onset [15] preeclampsia
Hexadecenoylcarnitine	Fatty acyls	Fatty acid esters	Lipid transport, metabolism, and peroxidation; fatty acid metabolism; cell signalling	Early onset [15] preeclampsia
Hexanoylcarnitine	Fatty acyls	Fatty acid esters	Lipid transport, metabolism, and peroxidation; fatty acid metabolism; cell signalling	Early onset [15] preeclampsia, late onset [15] preeclampsia
Hydroxyhexanoylcarnitine	Fatty acyls	Fatty acid esters	Lipid transport, metabolism, and peroxidation; fatty acid metabolism; cell signalling	Preeclampsia [5], early onset [5] preeclampsia
Isobutyrylcarnitine	Fatty acyls	Fatty acid esters	Lipid transport, metabolism, and peroxidation; fatty acid metabolism; cell signalling	Early onset [15] preeclampsia
Octanoylcarnitine	Fatty acyls	Fatty acid esters	Mitochondrial beta-oxidation of short chain saturated fatty acids	Early onset [15] preeclampsia, late onset [15] preeclampsia

For peer review only

Octenoylcarnitine	Fatty acyls	Fatty acid esters	Lipid transport, metabolism, and peroxidation; fatty acid metabolism; cell signalling	Early onset [15] preeclampsia, late onset [15] preeclampsia
Oleylcarnitine	Fatty acyls	Fatty acid esters	NA	Late onset [15] preeclampsia
Palmitoylcarnitine	Fatty acyls	Fatty acid esters	Fatty acid metabolism	Late onset [15] preeclampsia
Isoprostane 8-epi-prostaglandin F2α	Fatty acyls	Eicosanoids	Arachidonic acid metabolism	Preeclampsia [16]
(+/-) 5-iPF2α-VI	Fatty acyls	Eicosanoids	Lipid peroxidation	Preeclampsia [14]
(+/-) 5-iPF2α-VI-(d11)	Fatty acyls	Eicosanoids	Lipid peroxidation	Preeclampsia [14]
15-epilipoxin-A4	Fatty acyls	Eicosanoids	Lipid transport, metabolism, and peroxidation; fatty acid metabolism; cell signalling	Preeclampsia [17]
Alfa-linolenic acid	Fatty acyls	Lineolic acids and derivatives	Lipid transport, metabolism, and peroxidation; fatty acid metabolism; cell signalling; alpha linolenic acid and linoleic acid metabolism.	Preeclampsia [14]
Stearidonic acid	Fatty acyls	Lineolic acids and derivatives	Lipid transport, metabolism, and peroxidation; fatty acid metabolism; cell signalling; alpha linolenic acid and linoleic acid metabolism.	Preeclampsia [14]
Di-(octadecadienoyl)-sn-glycerol	Glycerophospholipids	Glycerophosphates	Lipid transport, metabolism, and peroxidation; fatty acid metabolism; cell signalling	Late onset [12] preeclampsia
Heptadecanoyl-2-hydroxy-sn-glycero-3-phosphocholine	Glycerophospholipids	Glycerophosphocholines	Lipid transport, metabolism, and peroxidation; fatty acid metabolism; cell signalling; phospholipid and glycerophospholipid metabolism.	Preeclampsia [18]
Dilinoleoyl-glycerol	Glycerophospholipids	Glycerophosphocholines	NA	Preeclampsia [18]

For peer review only

LysoPE C20:0	Glycerophospholipids	Glycerophosphoethanolamines	Lipid peroxidation and metabolism; glycerophospholipid metabolism; fatty acid metabolism cell signalling.	Preeclampsia [19]
Neomenthol-glucuronide	Prenol lipids	Terpene glycosides	Lipid peroxidation and metabolism; glycerophospholipid metabolism; fatty acid metabolism cell signalling.	Gestational hypertension [10]
Sphinganine 1-phosphate	Sphingolipids	Phosphosphingolipids	Sphingolipid metabolism, neuroactive ligand-receptor interaction	Late onset [12] preeclampsia
Sphingomyelin 16:0	Sphingolipids	Phosphosphingolipids	Lipid transport, metabolism, and peroxidation; fatty acid metabolism; cell signalling; apoptosis	Preeclampsia [20]
Sphingomyelin 18:0	Sphingolipids	Phosphosphingolipids	Phospholipid metabolism; lipid transport, metabolism, and peroxidation; fatty acid metabolism; cell signalling; apoptosis	Preeclampsia [20]
Sphingosine 1-phosphate	Sphingolipids	Phosphosphingolipids	Sphingolipid metabolism; lipid transport, metabolism, and peroxidation; fatty acid metabolism; cell signalling	Late onset [12] preeclampsia
Ceramide 14:0	Sphingolipids	Ceramides	NA	Preeclampsia [20]
Ceramide 24:0	Sphingolipids	Ceramides	NA	Preeclampsia [20]
Ceramide(d18:1/25:0)	Sphingolipids	Ceramides	NA	Preeclampsia [21]
Vitamin D	Steroids and steroids derivatives	Vitamin D and derivatives	Lipid transport, metabolism, and peroxidation; fatty acid metabolism; cell signalling	Preeclampsia [22], late onset [12] preeclampsia, gestational hypertension [23]
Bolasterone	Steroids and steroids derivatives	Androstan steroids	Lipid transport, metabolism, and peroxidation; fatty acid metabolism; cell signalling	Preeclampsia [10]
		Organoheterocyclic compounds		

Caffeine	Imidazopyrimidines	Purines and purine derivatives	Caffeine metabolism	Early onset [24] preeclampsia
Paraxanthine	Imidazopyrimidines	Purines and purine derivatives	Caffeine Metabolism	Early onset [24] preeclampsia
5-Hydroxytryptophan	Indoles and derivatives	Tryptamines and derivatives	Tryptophan metabolism	Late onset [12] preeclampsia
Serotonin	Indoles and derivatives	Tryptamines and derivatives	Tryptophan metabolism; nicotine action pathway	Late onset [4] preeclampsia
Gama-butyrolactone	Lactones	Gamma butyrolactones	NA	Late onset [12] preeclampsia
N-methyl-2-pyridone-5-carboxamide	Pyridines and derivatives	Pyridinecarboxylic acids and derivatives	Nicotinate and Nicotinamide Metabolism	Preeclampsia [3]
N-methylnicotinamide	Pyridines and derivatives	Pyridinecarboxylic acids and derivatives	NA	Preeclampsia [3]
Oxolan-3-one	Dihydrofurans	Furanones	NA	Late onset [12] preeclampsia
		Organic oxygen compounds		
4-deoxyerythronic acid	Organooxygen compounds	Carbohydrates and carbohydrate conjugates	NA	Preeclampsia [3]
Galactose	Organooxygen compounds	Carbohydrates and carbohydrate conjugates	Galactose metabolism, nucleotide sugars metabolismo, lactose degradation	Preeclampsia [3]
Glycerol	Organooxygen compounds	Carbohydrates and carbohydrate conjugates	Glycerolipid metabolism, galactose metabolism, glycerolipid metabolism	Early onset [11] preeclampsia
Lactose	Organooxygen compounds	Carbohydrates and carbohydrate conjugates	Galactose metabolism, Phosphotransferase system, carbohydrate digestion and absorption	Preeclampsia [3]
Sucrose	Organooxygen compounds	Carbohydrates and carbohydrate conjugates	Starch and sucrose metabolism, galactose metabolism	Preeclampsia [3]
Acetone	Organooxygen compounds	Carbonyl compounds	Ketone body metabolism	Early onset [11] preeclampsia, late onset [4] preeclampsia
		Organic nitrogen compounds		
Scyllo-inositol	Organonitrogen compounds	Alcohols and polyols	Inositol phosphate metabolism	Preeclampsia [3]

For peer review only

Propylene glycol	Organooxygen compounds	Alcohols and polyols	Pyruvate metabolism	Late onset [4] preeclampsia
Propane-1,3-diol	Organooxygen compounds	Alcohols and polyols	Glycerolipid metabolism	Preeclampsia [19]
Putrescine	Organonitrogen compounds	Amines	Methionine metabolism, spermidine and spermine biosynthesis	Late onset [4] preeclampsia
Trimethylamine	Organonitrogen compounds	Amines	Methane and carbon metabolisms.	Preeclampsia [2,10]
Carnitine	Organonitrogen compounds	Quaternary ammonium salts	Beta oxidation of very long chain and branched chain fatty acids, mitochondrial beta-oxidation of saturated fatty acids	Preeclampsia [3], late onset [4,13] preeclampsia
Linoleylcarnitine	Organonitrogen compounds	Quaternary ammonium salts	Lipid transport, metabolism, and peroxidation; fatty acid metabolism; cell signalling	Early onset [15] preeclampsia, late onset [15] preeclampsia
Stearoylcarnitine	Organonitrogen compounds	Quaternary ammonium salts	Mitochondrial beta-oxidation of long chain saturated fatty acids	Early onset [15] preeclampsia, late onset [15] preeclampsia
Tetradecenoylcarnitine	Organonitrogen compounds	Quaternary ammonium salts	Lipid transport, metabolism, and peroxidation; fatty acid metabolism; cell signalling	Early onset [15] preeclampsia, late onset [15] preeclampsia
		Benzenoids		
Bisphenol A	Benzene and substituted derivatives	Diphenylmethanes	Bisphenol degradation	Preeclampsia [25]
Hippurate	Benzene and substituted derivatives	Benzoic acids and derivatives	Phenylalanine metabolism	Preeclampsia [3]
4-OH-hippurate	Benzene and substituted derivatives	Benzoic acids and derivatives	NA	Preeclampsia [3]
Mono(2- ethyl-5-oxohexyl) phthalate	Benzene and substituted derivatives	Benzoic acids and derivatives	NA	Late onset [25] preeclampsia

For peer review only

Mono(2-ethyl-5-carboxypentyl) phthalate	Benzene and substituted derivatives	Benzoic acids and derivatives	NA	Late onset [25] preeclampsia
Mono(2-ethylhexyl) phthalate	Benzene and substituted derivatives	Benzoic acids and derivatives	NA	Preeclampsia [25]
Mono(3-carboxypropyl) phthalate	Benzene and substituted derivatives	Benzoic acids and derivatives	NA	Preeclampsia [25]
Mono-ethyl phthalate	Benzene and substituted derivatives	Benzoic acids and derivatives	NA	Preeclampsia [25], late onset [25] preeclampsia
Monoisobutyl phthalate	Benzene and substituted derivatives	Benzoic acids and derivatives	NA	Preeclampsia [25]
Mono-*n*-butyl phthalate	Benzene and substituted derivatives	Benzoic acids and derivatives	NA	Late onset [25] preeclampsia
Σ di(2-ethylhexyl) phthalate	Benzene and substituted derivatives	Benzoic acids and derivatives	NA	Preeclampsia [25], Late onset [25] preeclampsia
2,6-Di-tert-butyl-4-hydroxymethylphenol	Benzene and substituted derivatives	Phenylpropanes	NA	Gestational hypertension [10]
		Organohalogen compounds		
Perfluorohexane sulfonate	Alkyl halides	Alkyl fluorides	NA	Preeclampsia [26]
		Alkaloids and derivatives		
Trigonelline	Alkaloids derived from nicotinic acid	Pyridine alkaloids	Nicotinate and nicotinamide metabolism	Preeclampsia [3]
Pilocarpine	NA	NA	Biosynthesis of alkaloids derived from histidine and purine	Gestational hypertension [10]
		Phenylpropanoids and polyketides		
Cerasinone	Flavonoids	O-methylated flavonoids	NA	Preeclampsia [10]
		Organosulfur compounds		
Dimethyl sulfone	Sulfonyls	Sulfones	NA	Preeclampsia [2]

NA = not available

For peer review only

References

1 Gong S, Sovio U, Aye ILMH, *et al.* Placental polyamine metabolism differs by fetal sex, fetal growth restriction, and preeclampsia. *JCI Insight* 2018;**3**:1–15. doi:10.1172/jci.insight.120723

2 Kenny LC, Broadhurst DI, Hong W, *et al.* NMR Profiling in Early Pregnancy Reveals a Metabolomic Signature the Predicts the Subsequent Development Preeclampsia. *Reprod Sci* 2013;**20**:2641A. doi:10.1177/1933719113482088

3 Diaz SO, Barros AS, Goodfellow BJ, *et al.* Second trimester maternal urine for the diagnosis of trisomy 21 and prediction of poor pregnancy outcomes. *J Proteome Res* 2013;**12**:2946–57. doi:10.1021/pr4002355

4 Bahado-Singh R, Poon LC, Yilmaz A, *et al.* Integrated Proteomic and Metabolomic prediction of Term Preeclampsia. *Sci Rep* 2017;**7**:1–10. doi:10.1038/s41598-017-15882-9

5 Odibo AO, Goetzinger KR, Odibo L, *et al.* First-trimester prediction of preeclampsia using metabolomic biomarkers: a discovery phase study. *Prenat Diagn* 2011;**31**:990–4.

6 Rijvers CAH, Marzano S, Winkens B, *et al.* Early-pregnancy asymmetric dimethylarginine (ADMA) levels in women prone to develop recurrent hypertension. *Pregnancy Hypertens* 2013;**3**:118–23. doi:10.1016/j.preghy.2013.01.001

7 Khalil AA, Tsikas D, Akolekar R, *et al.* Asymmetric dimethylarginine, arginine and homoarginine at 11-13 weeks gestation and preeclampsia: A case-control study. *J Hum Hypertens* 2013;**27**:38–43. doi:10.1038/jhh.2011.109

8 Sovio U, McBride N, Wood AM, *et al.* 4-Hydroxyglutamate is a novel predictor of pre-eclampsia. *Int J Epidemiol* 2020;**49**:301–11. doi:10.1093/ije/dyz098

9 Kuc S, Koster MPH, Pennings JLA, *et al.* Metabolomics profiling for identification of novel potential markers in early prediction of preeclampsia. *PLoS One* 2014;**9**. doi:10.1371/journal.pone.0098540

10 Harville EW, Li Y-YY-Y, Pan K, *et al.* Untargeted analysis of first trimester serum to reveal biomarkers of pregnancy complications: a case-control discovery phase study. *Sci Rep* 2021;**11**:3468. doi:10.1038/s41598-021-82804-1

11 Bahado-Singh RO, Syngelaki A, Akolekar R, *et al.* Validation of metabolomic models for prediction of early-onset preeclampsia. *Am J Obstet Gynecol* 2015;**213**:530.e1-530.e10. doi:10.1016/j.ajog.2015.06.044

12 Kenny LC, Broadhurst DI, Dunn W, *et al.* Robust early pregnancy prediction of later preeclampsia using metabolomic biomarkers. *Hypertension* 2010;**56**:741–9. doi:10.1161/HYPERTENSIONAHA.110.157297

13 Bahado-Singh RO, Syngelaki A, Mandal R, *et al.* Metabolomic determination of pathogenesis of late-onset preeclampsia. *J Matern Neonatal Med* 2017;**30**:658–64. doi:10.1080/14767058.2016.1185411

14 Bilodeau JF, Qin Wei S, Larose J, *et al.* Plasma F2-isoprostane class VI isomers at 12-18 weeks of pregnancy are associated with later occurrence of preeclampsia. *Free Radic Biol Med* 2015;**85**:282–7. doi:10.1016/j.freeradbiomed.2015.05.012

For peer review only

15 Koster MPH, Vreeken RJ, Harms AC, *et al.* First-Trimester serum acylcarnitine levels to predict preeclampsia: A metabolomics approach. *Dis Markers* 2015;**2015**. doi:10.1155/2015/857108

16 Chappell L. A longitudinal study of biochemical variables in women at risk of preeclampsia. *Am J Obstet Gynecol* 2002;**187**:127–36. doi:10.1067/mob.2002.122969

17 Shanmugalingam R, Wang X, Motum P, *et al.* The 15-Epilipoxin-A4 Pathway with Prophylactic Aspirin in PreventingPreeclampsia: A Longitudinal Cohort Study. *J Clin Endocrinol Metab* 2020;**105**. doi:10.1210/clinem/dgaa642

18 Kenny LC, Thomas G, Poston L, *et al.* Prediction of preeclampsia risk in first time pregnant women: Metabolite biomarkers for a clinical test. *PLoS One* 2020;**15**:1–19. doi:10.1371/journal.pone.0244369

19 Lee SM, Kang Y, Lee EM, *et al.* Metabolomic biomarkers in midtrimester maternal plasma can accurately predict the development of preeclampsia. *Sci Rep* 2020;**10**:16142. doi:10.1038/s41598-020-72852-4

20 Dobierzewska A, Soman S, Illanes SE, *et al.* Plasma cross-gestational sphingolipidomic analyses reveal potential first trimester biomarkers of preeclampsia. *PLoS One* 2017;**12**:1–16. doi:10.1371/journal.pone.0175118

21 Huang Q, Hao S, You J, *et al.* Early-pregnancy prediction of risk for pre-eclampsia using maternal blood leptin/ceramide ratio: Discovery and confirmation. *BMJ Open* 2021;**11**. doi:10.1136/bmjopen-2021-050963

22 Woodham PC, Brittain JE, Baker AM, *et al.* Midgestation maternal serum 25-hydroxyvitamin D level and soluble fms-like tyrosine kinase 1/placental growth factor ratio as predictors of severe preeclampsia. *Hypertension* 2011;**58**:1120–5. doi:10.1161/HYPERTENSIONAHA.111.179069

23 Ates S, Sevket O, Ozcan P, *et al.* Vitamin D status in the first-trimester: Effects of vitamin D deficiency on pregnancy outcomes. *Afr Health Sci* 2016;**16**:36–43. doi:10.4314/ahs.v16i1.5

24 Eichelberger KY, Baker AM, Woodham PC, *et al.* Second-trimester maternal serum paraxanthine, CYP1A2 activity, and the risk of severe preeclampsia. *Obstet Gynecol* 2015;**126**:725–30. doi:10.1097/AOG.0000000000001041

25 Cantonwine DE, Meeker JD, Ferguson KK, *et al.* Urinary concentrations of bisphenol A and phthalate metabolites measured during pregnancy and risk of preeclampsia. *Environ Health Perspect* 2016;**124**:1651–5. doi:10.1289/EHP188

26 Rylander L, Lindh CH, Hansson SR, *et al.* Per- and Polyfluoroalkyl Substances in Early Pregnancy and Risk for Preeclampsia: A Case-Control Study in Southern Sweden. *Toxics* 2020;**8**. doi:10.3390/toxics8020043

For peer review only

Studies	Patient selection		Index test	
	Was a consecutive or random sample of patients enrolled?	Did the study avoid inappropriate exclusions?	Were the index test results interpreted without knowledge of the results of the reference standard?	If a threshold was used, was it pre-specified?
Chappell LC et al, 2002	Yes	No	Unclear	Yes
Powe et al, 2010	Yes	Yes	Unclear	Yes
Kenny LC et al, 2010	Yes	Yes	Yes	Yes
Odibo AO et al, 2011	Yes	No	Unclear	Yes
Woodham PC et al, 2011	Yes	Yes	Unclear	Yes
Rijvers CAH et al, 2013	Unclear	Unclear	Unclear	Yes
Khalil AA et al, 2013	Yes	Yes	Yes	Yes
Diaz SO et al, 2013	Yes	Unclear	Unclear	Yes
Kenny LC et al, 2013	Unclear	Unclear	Unclear	Yes
Kuc S et al, 2014	Yes	Yes	Unclear	Yes
Wetta LA et al, 2014	Yes	Yes	Unclear	Yes
Bahado-Singh RO et al, 2015	Yes	Yes	Unclear	Yes
Eichelberger KY et al, 2015	Yes	Yes	Yes	Yes
Koster MPH et al, 2015	Yes	Yes	Unclear	Yes
Bilodeau JF et al, 2015	Yes	Yes	Unclear	Yes
Ates S et al, 2016	Yes	Yes	Unclear	Yes
Cantonwine DE et al, 2016	Yes	Yes	Unclear	Yes
Kiely M et al, 2016	Yes	Yes	Yes	Yes
Bahado-Singh RO et al, 2017	Yes	Yes	Unclear	Yes
Bahado-Singh RO et al, 2017b	Yes	Yes	Unclear	Yes
Dobierzewska A et al, 2017	Yes	Yes	Unclear	Yes
Ye Y et al, 2017	Yes	No	Yes	Yes
Gong S et al, 2018	Yes	Yes	Unclear	Yes
Tamblyn JA et al, 2018	Yes	Yes	Yes	Yes
Sovio U et al, 2020	Yes	Yes	Unclear	Yes
Huo X et al, 2020	Yes	No	Unclear	Yes
Rylander L et al, 2020	Yes	Unclear	Unclear	Yes

For peer review only

Lee SM et al, 2020	Unclear	Unclear	Unclear	Yes
Kenny L et al, 2020	Yes	Yes	Yes	Yes
Shanmugalingam R et al, 2020	Yes	No	Unclear	Yes
Harville EW et al, 2021	Yes	Unclear	Unclear	Yes
Huang Q et al, 2021	Unclear	Yes	Unclear	Yes

For peer review only

Risk of bias				
Reference standard			Flow and timing	
Is the reference standard likely to correctly classify the target condition?	Were the reference standard results interpreted without knowledge of the results of the index test?	Was there an appropriate interval between index test and reference standard?	Did all patients receive the same reference standard?	Were all patients included in the analysis?
Yes	Yes	Yes	Yes	Yes
Yes	Unclear	Yes	Yes	Yes
Yes	Yes	Yes	Yes	Yes
Yes	Unclear	Yes	Yes	Yes
Yes	Unclear	Yes	Yes	Yes
Yes	Unclear	Yes	Yes	Yes
Yes	Unclear	Yes	Yes	Yes
Yes	Unclear	Unclear	Yes	Yes
Yes	Unclear	Yes	Yes	Yes
Yes	Unclear	Yes	Yes	Yes
Yes	Yes	Yes	Yes	Yes
Yes	Unclear	Yes	Yes	Yes
Yes	Unclear	Yes	Yes	Yes
Yes	Unclear	Yes	Yes	Yes
Yes	Unclear	Yes	Yes	Yes
Yes	Unclear	Yes	Yes	Yes
Yes	Unclear	Unclear	Yes	No
Yes	Yes	Yes	Yes	Yes
Yes	Unclear	Yes	Yes	Yes
Yes	Unclear	Yes	Yes	Yes
Yes	Unclear	Yes	Yes	Yes
Yes	Unclear	Yes	Yes	Yes
Yes	Unclear	Yes	Yes	Yes
Yes	Yes	Yes	Yes	Yes
Yes	Unclear	Yes	Yes	Yes
Yes	Unclear	Yes	Yes	Yes
Yes	Unclear	Yes	Yes	No

For peer review only

Yes	Unclear	Yes	Yes	Yes
Yes	Yes	Yes	Yes	Yes
Yes	Unclear	Yes	Yes	No
Unclear	Unclear	Yes	Yes	Yes
Yes	Unclear	Yes	Yes	Yes

For peer review only

Applicability concerns		
**Patient selection**	**Index test**	**Reference standard**
Are there concerns that the included patients do not match the review question?	Are there concerns that the index test, its conduct, or interpretation differ from the review question?	Are there concerns that the target condition as defined by the reference standard does not match the review
No	No	No
No	No	No
No	No	No
No	No	No
No	No	No
No	No	No
No	No	No
Yes	No	No
No	No	No
No	No	No
No	No	No
No	No	No
No	No	No
No	No	No
No	No	No
No	No	No
No	Yes	No
No	No	No
No	No	No
No	No	No
No	No	No
No	No	No
No	No	No
No	No	No
No	No	No
Yes	No	No
No	No	No

For peer review only

No	No	No
No	No	No
Yes	No	No
No	No	Yes
No	No	No

For peer review only

BMJ Open

Prediction of pregnancy-related hypertensive disorders using metabolomics: a systematic review

Journal:	*BMJ Open*
Manuscript ID	bmjopen-2021-054697.R3
Article Type:	Original research
Date Submitted by the Author:	03-Apr-2022
Complete List of Authors:	Mayrink, Jussara; State University of Campinas Faculty of Medical Sciences, Obstetrics and Gynecology<br>Leite, Debora; State University of Campinas Faculty of Medical Sciences, Obstetrics and Gynecology<br>Nobrega, Guilherme; State University of Campinas Faculty of Medical Sciences, Department of Obstetrics and Gynecology<br>Costa, Maria Laura; State University of Campinas Faculty of Medical Sciences, Obstetrics and Gynecology<br>Cecatti, Jose; State University of Campinas Faculty of Medical Sciences, Obstetrics and Gynecology
**Primary Subject Heading**:	Obstetrics and gynaecology
Secondary Subject Heading:	Diagnostics
Keywords:	Hypertension < CARDIOLOGY, Maternal medicine < OBSTETRICS, EPIDEMIOLOGY

SCHOLARONE™
Manuscripts

I, the Submitting Author has the right to grant and does grant on behalf of all authors of the Work (as defined in the below author licence), an exclusive licence and/or a non-exclusive licence for contributions from authors who are: i) UK Crown employees; ii) where BMJ has agreed a CC-BY licence shall apply, and/or iii) in accordance with the terms applicable for US Federal Government officers or employees acting as part of their official duties; on a worldwide, perpetual, irrevocable, royalty-free basis to BMJ Publishing Group Ltd ("BMJ") its licensees and where the relevant Journal is co-owned by BMJ to the co-owners of the Journal, to publish the Work in this journal and any other BMJ products and to exploit all rights, as set out in our licence.

The Submitting Author accepts and understands that any supply made under these terms is made by BMJ to the Submitting Author unless you are acting as an employee on behalf of your employer or a postgraduate student of an affiliated institution which is paying any applicable article publishing charge ("APC") for Open Access articles. Where the Submitting Author wishes to make the Work available on an Open Access basis (and intends to pay the relevant APC), the terms of reuse of such Open Access shall be governed by a Creative Commons licence – details of these licences and which Creative Commons licence will apply to this Work are set out in our licence referred to above.

Other than as permitted in any relevant BMJ Author's Self Archiving Policies, I confirm this Work has not been accepted for publication elsewhere, is not being considered for publication elsewhere and does not duplicate material already published. I confirm all authors consent to publication of this Work and authorise the granting of this licence.

For peer review only

SYSTEMATIC REVIEW

Prediction of pregnancy-related hypertensive disorders using metabolomics: a systematic review

Jussara Mayrink, MD, PhD [1,2] (0000-0001-5957-4365; jussaramayrink@gmail.com)
Debora Farias B. Leite, MD, PhD [1,3] (0000-0001-8839-3934; debora.leite@ufpe.br)
Guilherme Moraes Nobrega, BSc [1] (0000-0001-9406-4256; guigo.nobrega@hotmail.com)
Maria Laura Costa, MD, PhD [1] **(**0000-0001-8280-3234; mlaura@unicamp.br)
José Guilherme Cecatti, MD, PhD [1] (0000-0003-1285-8445; cecatti@unicamp.br)

[1]Department of Obstetrics and Gynecology, University of Campinas, School of Medicine, Brazil
[2]Federal University of Minas Gerais, Belo Horizonte, Minas Gerais, Brazil.
[3]Department of Gynecology and Obstetrics, Federal University of Pernambuco, Recife, Pernambuco, Brazil.

Correspondence:
José Guilherme Cecatti
Rua Alexander Fleming, 101 Cidade Universitaria Zeferino Vaz.
13083-889. Campinas, Sao Paulo, Brazil.
Telephone: +55 19-35219482
Email: cecatti@unicamp.br

Word Count:
Abstract: 300 words
Main text: 5,144 words

For peer review only

Abstract

Objective: To determine the accuracy of metabolomics in predicting hypertensive disorders in pregnancy.

Design: Systematic Review of observational studies.

Data sources and study eligibility criteria: An electronic literature search was performed in June, 2019 and February, 2022. Two researchers independently selected studies published between 1998 and 2022 on metabolomic techniques applied to predict the condition; subsequently, they extracted data and performed quality assessment. Discrepancies were dealt with a third reviewer. The primary outcome was pre-eclampsia. Cohort or case-control studies were eligible when maternal samples were taken before diagnosis of the hypertensive disorder.

Study appraisal and synthesis methods: Data on study design, maternal characteristics, how hypertension was diagnosed, metabolomics details and metabolites, and accuracy were independently extracted by two authors.

Results: Among 4,613 initially identified studies on metabolomics, 68 were read in full text and 32 articles were included. Studies were excluded due to duplicated data, study design or lack of identification of metabolites. Metabolomics was applied mainly in the 2nd trimester; the most common technique was liquid-chromatography coupled to mass spectrometry. Among the 122 different metabolites found, there were 23 amino acids and 21 fatty acids. Most of the metabolites were involved with ammonia recycling; amino acid metabolism; arachidonic acid metabolism; lipid transport, metabolism, and peroxidation; fatty acid metabolism; cell signalling; galactose metabolism; nucleotide sugars metabolism; lactose degradation; and glycerolipid metabolism. Only citrate was a common metabolite for prediction of early- and late-onset pre-eclampsia. Vitamin D was the only metabolite in common for pre-eclampsia and gestational hypertension prediction. Meta-analysis was not performed due to lack of appropriate standardized data.

Conclusions and Implications: Metabolite signatures may contribute to further insights into the pathogenesis of preeclampsia and support screening tests. Nevertheless, it is mandatory to validate such methods in larger studies with a heterogeneous population to ascertain the potential for their use in clinical practice.

PROSPERO registration: CRD42018097409.

For peer review only - http://bmjopen.bmj.com/site/about/guidelines.xhtml

Keywords: preeclampsia, systematic review, hypertension, metabolomics, metabolome, prediction.

Strengths and limitations of this study

- This systematic review presents possible biomarkers of pre-eclampsia that could provide a basis for further research in this area.
- There was great heterogeneity among included studies due to the complexity of metabolomics procedures, which are influenced by sample collection, storage and preparation, analytical platform applied, and statistical tests performed.
- Our findings must be treated with caution due to the lack of standardization of pre-eclampsia definition (early and late-onset pre-eclampsia) and timing of sample collection in pregnancy.

Original protocol: Mayrink J, Leite DFB, Costa ML, *et al*. Metabolomics for prediction of hypertension in pregnancy: a systematic review and meta-analysis protocol. *BMJ Open* 2020;10:e040652. doi:10.1136/bmjopen-2020-040652.

For peer review only

INTRODUCTION

Hypertensive disorders have been reported as associated with adverse outcomes in pregnancy since the 1970s. They are a heterogeneous group of conditions that include chronic hypertension, preeclampsia, gestational hypertension and preeclampsia superimposed on chronic hypertension.[1]

Chronic hypertension is the condition of systolic blood pressure ≥140 mmHg and/or diastolic blood pressure ≥ 90 mmHg before twenty weeks of gestation or even before pregnancy. [2] Gestational hypertension is the isolated elevation of pressure after twenty weeks of gestation. It is less damaging than preeclampsia but around 30% of these cases progress to pre-eclampsia.[3] The most prevalent form – pre-eclampsia - is a serious and potentially-life threatening condition to the mother and the baby, [2] and its high prevalence rate persists over the years, ranging from 3 to 10% of all pregnancies, according to the country studied. Unfortunately, treatment has been the same over decades and relies on the management of clinical features, such as new onset of hypertension and end-organ dysfunction, that characterizes preeclampsia. Outcomes mostly depend upon accurate diagnosis and decision on timing of childbirth since there are few predictive tools available. [3] As a result, one of the most urgent priorities is to identify those women at high-risk for the disease, who would be candidates for prophylactic and increased surveillance measures. The increased understanding on pathophysiology would also shed light into possible new therapeutic approaches.

Several biomarkers have been studied as predictive tools for preeclampsia, e.g. soluble fms-like tyrosine kinase-1, soluble endoglin, markers of apoptosis and inflammation, placental protein 13, C-reactive protein, and markers of placental hypoxia and distress. [4] However, none are sufficiently sensitive or specific to predict preeclampsia in advance.

Recently, novel technologies, such as metabolomics, have been applied to predict preeclampsia. Metabolomics is known as the newest member of the “omics” family. Metabolome - a collection of metabolites – is defined and used in research to pursue

For peer review only - http://bmjopen.bmj.com/site/about/guidelines.xhtml

the phenotypic signature of a disease of interest. Metabolites are low-molecular-weight chemicals (<1500 Da) resulting from changes in gene, protein expression [5] and environmental interferences. Metabolomics methods include hydrogen nuclear magnetic resonance spectroscopy (^{1}H-NMR) and gas- and liquid- chromatography-mass spectrometry (GC- and LC-MS, respectively). [5,6] Metabolomics has been applied to improve disease biomarkers and understand the pathogenesis of many conditions, such as cancer and diabetes. [7,8] One could argue its value in evaluating preeclampsia, which has a complex aetiology and is considered a multifactorial disease. Metabolomics, however, a high throughput technique, seems perfectly adequate, since it can simultaneously encompass a wide range of metabolic pathways. The objective of this systematic review was to determine the accuracy of metabolomics in predicting pregnancy-induced hypertensive disorders.

METHODS

This systematic review was conducted based on our previously published protocol [9] (CRD42018097409) and is reported in accordance with the Preferred Reported Items for Systematic Reviews and Meta-Analysis (PRISMA) statement; [10] a PRISMA checklist is provided as a Supplementary Material 1. Our research question was: "what is the performance of metabolomics for predicting gestational hypertensive disorders?"

Population and Interventions

Our domain of interest was any form of hypertensive disorder developed during pregnancy, in relation to either single or multiple gestations. Metabolomics was the intervention studied. Papers eligible for inclusion in our review should mention metabolites potentially differentiating hypertensive from normotensive pregnant women. Maternal samples should have been drawn during pregnancy and before diagnosis of pregnancy-induced hypertensive disorder. We excluded studies in which blood samples were collected after the diagnosis of hypertension was established, or when preeclampsia and gestational hypertension were analysed as the same outcome.

For peer review only - http://bmjopen.bmj.com/site/about/guidelines.xhtml

Comparison and Outcomes

We included studies in which women with any form of pregnancy-induced hypertension were compared to pregnant women without hypertension. Preeclampsia was the primary outcome, diagnosed by criteria defined by the authors. [11–15]

Secondary outcomes were: a) early-onset preeclampsia, characterized by delivery <34 weeks due to preeclampsia; [3] b) late-onset preeclampsia, requiring delivery ≥34 weeks; [3] c) gestational hypertension, when hypertension is the only clinical finding after 20 weeks gestation; d) preeclampsia superimposed on chronic hypertension; e) white coat hypertension, i.e., normal blood pressure recorded in a 24h ambulatory monitoring; f) masked hypertension, characterized as normal blood pressure in the office or clinic and altered under other circumstances; and g) transient gestational hypertension, higher blood pressure at levels during a certain period that is later normalized after repeated blood pressure readings. [11,16]

Search strategy

Two independent researchers (JM, DFBL) performed an electronic database search in PubMed, EMBASE, Scopus, Web of Knowledge, Latin American and Caribbean Health Sciences Literature (LILACS), Scientific Electronic Library Online (SciELO), Health Technology Assessment (HTA), and Database of Abstracts of Reviews of Effects (DARE), including studies published from 1998 to 2022. The full search strategies are provided as Supplementary Material 2. We have applied no filters, except for SCOPUS (we have used the Title, Abstract and Keyword filter). The grey literature (conference abstracts) and the reference list of included studies were searched for additional articles for inclusion.

There were no language restrictions; only cohort and case control studies were included. Letters to the editor, editorials, comments, expert opinions, any type of review, experimental animal studies, case reports, intervention trials, and cross-sectional studies were all excluded. We intend to show the capacity of metabolomics

For peer review only - http://bmjopen.bmj.com/site/about/guidelines.xhtml

to predict hypertensive disorders without interference of any intervention. Thus, it is absolutely necessary to guarantee the temporal relation between timing of sample (before) and outcome (after). This is the reason for including just cohort and case control studies.

Data extraction and quality assessment

Trained reviewers (JM, DFBL) proceeded studies' screening (using EndNote® software), data extraction and synthesis, and risk of bias evaluation (Quality Assessment of Diagnostic Accuracy Studies (QUADAS-2) [17]). Disagreements were solved by a third reviewer (MLC).

Summary of data and meta-analysis

For all included studies, we have extracted data on research characteristics and metabolomics details. If the study comprised training and validation datasets, we have extracted metabolomics data from the validation group of women. When the study has not definitively identified a given metabolite, we have extracted all possible metabolites. We have extracted data of metabolites combined with other biomarkers, when available. We have used the Human Metabolome Database [18], the Kyoto Encyclopaedia of Genes and Genomes [19] and the Lipid Maps [20] for matching each metabolite chemical class and subclass, and metabolic pathways and processes.

When available, data on accuracy measures on metabolomics performance in predicting hypertensive disorders of pregnancy were extracted. We planned to perform the estimation of likelihood ratios and hierarchical summary receiver operator characteristic curve, [21] and the assessment of heterogeneity and publication bias. [22]

Ethical aspects

This is a systematic review dealing with data from articles already published and, therefore, not directly involving human subjects. It has then no need for ethical assessment and approval according to the national rules.

For peer review only - http://bmjopen.bmj.com/site/about/guidelines.xhtml

Patient and Public Involvement

Considering this is a systematic review, patients and the public were not involved in this study at all.

RESULTS

Main findings

The literature search for this systematic review was performed in June 2019 with a further re-run in February 2022. **Figure 1** summarizes the flow chart of studies' selection and final inclusion. We excluded studies (Supplementary Material 3) due to study design (25 studies) or lack of identification of metabolites (05 studies). Regarding duplicated data, only the most recent and complete study was included (06 studies).

Overall, 32 studies were selected: 25 case control studies [23–47] and seven cohort studies [48–54] (**Table 1**). Studies from the same research project but evaluating different metabolites [43,46] or populations [24,28,29,33,36,38,39] were included. Most studies were developed in Europe [23,25,28,29,31,33,36,38,39,42,43,46,48–52] and North America; [32,34,35,40,41,44,45,47,54] there was only one research from Latin America, [26] two from Oceania [24,53] and another three from Asia. [27,30,37] Twelve studies have sampled maternal specimens in the 1st trimester, [31,32,34,35,42,43,46,48,50–53] 15 studies have sampled in the 2nd trimester, [23,24,27,29,30,33,36–39,41,44,45,49,54] four studies have sampled at least two times [25,26,40,47] in pregnancy, and one study included women in late 3rd trimester [28] (**Figure 2**). Four studies [24,33,38,40] have performed training and validation with different populations.

Studies have defined preeclampsia as hypertension developed in pregnancy associated with proteinuria [11,13–15] or end-organ dysfunction. [12] Although not consistently, chronic hypertension or other illnesses, [24,27,29,33,36–39,41,45,55] and multiple pregnancies [25,27,30,32,33,36,37,39,41,42,45,52] were reasons for participants exclusion. When chronic hypertensive women were included, there were women taking aspirin as prophylaxis. [23,53] Then, there are no specific data on superimposed

For peer review only - http://bmjopen.bmj.com/site/about/guidelines.xhtml

preeclampsia or multiple pregnancies. Similarly, there are no studies on white coat hypertension, masked hypertension, or transient gestational hypertension. Only three studies have brought data on gestational hypertension. [32,37,51] Small for gestational age (SGA) infants were fully excluded [31] or included only in preeclampsia group, either because they were excluded from control groups, [23,34] or because this variable was not used as for matching cases and controls. [24,29,36]

In **Table 2**, characteristics of the included studies regarding metabolomics techniques and accuracy measures are shown according to the primary and secondary outcomes. The majority of studies did not present data according to gestational age of delivery. The most common technique was LC-MS, [24,26,27,29,31,33–38,40,41,43–48,51,53,55] followed by H-NMR [25,39,49,50,52] and GC-MS. [23,42] Bahado-Singh et al. [25] (H-NMR followed by LC-MS), Bilodeau et al. [54] (HPLC-MS/MS and GC-FID), Lee et al. [30] (GC-MS and LC-MS), and Harville et al. [32] (LC-MS and NMR) have used two complementary techniques. Most studies analysed blood samples, [23,24,26–28,30–46,48,50–54] two studies analysed urine [47,49] and one study analysed both, urine and blood [29] (**Figure 2**). There were no studies carrying out analyses on maternal hair or amniotic fluid. All studies that used H-NMR [25,32,39,49,50,52] and four that applied LC-MS [24,28,32,38] were untargeted evaluations. Eleven studies reported lipidomic analysis and/or amino acid analysis. [23,26,30,35,40,42,43,46,48,53,54] Six studies described vitamin D analysis, all of them using LC-MS.[29,34,36,41,44,51] Five studies reported environmental exposures, focusing on caffeine, [45] bisphenol A, [27] phthalates [47] and per-and polyfluoroalkyl substances (PFAS). [31,37] Two studies have looked for differences in preeclampsia prediction according to foetal sex. [28,47] There were five metabolites in common between preeclampsia and SGA prediction (sphinganine 1-phosphate, sphingosine 1-phosphate, tyrosine, vitamin D, N1, N12-diacetylspermine). [56]

For preeclampsia requiring delivery at any gestational age, the lowest area under the curve (AUC) was for isoprostane 8-epi-prostaglandin F2α (0.55), [23] and the highest (AUC 0.95) was for a model composed by cerasinone, bolasterone, and two unidentified metabolites, presented as retention time and mass (6.30_477.7721m/z,

For peer review only - http://bmjopen.bmj.com/site/about/guidelines.xhtml

5.86_313.1070m/z).[32] For early onset preeclampsia, the AUC of untargeted metabolomics in 1st trimester maternal blood varied from 0.673 (UPLC-MS/MS) [38] to 0.835 (H-NMR). [50] For late onset preeclampsia, the AUC of untargeted metabolomics in 1st trimester maternal blood was 0.629 (H-NMR); [52] in 2nd trimester, 0.92 (UPLC-MS); [24] and in 3rd trimester, 0.761 (H-NMR and LC-MS/MS). [25]

When analysed together with non-metabolomics biomarkers (**Table 3**), the best performance was achieved by taurine combined with maternal prior risk and mean arterial blood pressure (MAP) (AUC 0.93 in 1st trimester for early onset preeclampsia); [43] followed by 3-hydroxyisovalerate, arginine, glycerol combined with uterine arteries pulsatility index (UtPI) (AUC 0.917 in 1st trimester for early onset preeclampsia) [50]; and leptin/ceramide(d18:1/25:0) ratio in in 1st and 2nd trimester (AUC 0.876 and 0.892, respectively) for preeclampsia at any gestational age. [40]

All predictive metabolites of hypertensive disorders of pregnancy according to chemical class and subclass, and metabolic pathways possibly involved are presented as Supplementary Material 4. Ten metabolites could not be found in the metabolome databases (U4, U10, U14, U35, [49] hexadecenoyleicosatetraenoyl-Sn-glycerol; [24] SM C28:1, SM C30:1, LysoPC C19:0 [30], dibenzylamine [32], perfluoroheptanoic acid [37]). There were 121 different metabolites, from ten different super classes. The majority of metabolites corresponded to amino acids, peptides, and analogues (23 compounds); fatty acid esters (11 compounds); fatty acids and conjugates (10 compounds); and carbohydrates and carbohydrate conjugates (5 compounds). They are mainly involved with ammonia recycling; amino acid metabolism; arachidonic acid metabolism; lipid transport, metabolism, and peroxidation; fatty acid metabolism; cell signalling; galactose metabolism; nucleotide sugars metabolism; lactose degradation; and glycerolipid metabolism. There were 10 benzoic acids and derivatives, but only hippurate has a known pathway. Citrate was found in common for prediction of preeclampsia in any gestational age, [49] early [50] and late-onset [25] disease. Octenoylcarnitine, [46] octanoylcarnitine, [46] acetone, [25,50] linoleylcarnitine, [46] stearoylcarnitine, [46] tetradecenoylcarnitine [46] were predictive for both early and

For peer review only - http://bmjopen.bmj.com/site/about/guidelines.xhtml

late preeclampsia. Vitamin D was the only metabolite involved simultaneously with preeclampsia, [41] late onset [24] preeclampsia, and gestational hypertension. [51]

Summary of included studies

The untargeted studies provided a great number of predictive metabolites. Kenny et al. showed a decrease of 13 metabolites (NMR) [39] and an increment of another 40 metabolites (LC-MS) [24] in blood analysis between 14-16 weeks of nulliparous pregnant women exclusively. In a validation phase with a list of 14 different metabolites, the odds ratio of developing preeclampsia, at any gestational age, was 23 (95% CI: 7-73), with AUC 0.92. [24] These same metabolites were validated in a different population, [33] and the dilinoleoyl-glycerol has achieved an AUC 0.70 (0.59-0.82).

Bahado-Singh et al. applied metabolomics to predict early- and late-onset cases. [25,50,52] Using maternal 1st trimester blood (11-13w), those authors reinforced the theory of the multiple phenotypes for preeclampsia. [57,58] 2-hydroxybutyrate was identified as predictor of early-onset preeclampsia, [50] whereas carnitine, pyruvate and acetone resulted in an AUC of 0.629 for late-onset disease. This latter AUC increased to 0.734 by combining metabolites and uterine Doppler velocimetry and maternal weight. [52] Finally, in a two-step evaluation in the 1st and 3rd trimester to predict term preeclampsia (≥37w), a combination of metabolomics and proteomics yielded an AUC of 0.817. [25]

Through an untargeted urinary metabolome analysis, Diaz et al. [49] showed decreased levels of acetate, formate, fumarate (all involved with the Krebs cycle), succinate, and isoleucine; and increased levels of 4-deoxyerythronic acid, a degradation product of 3-hydroxybutyrate [49] between 14-26w. Finally, still working with an untargeted approach, Sovio et al. demonstrated an increase of 4-hydroxyglutamate serum levels among women who developed early-onset preeclampsia, measured at 12w. [38] Its prediction performance was improved by addition of pregnancy-associated plasma protein A (PAPP-A) and PlGF. [38]

For peer review only - http://bmjopen.bmj.com/site/about/guidelines.xhtml

Regarding lipidomic or amino-acid studies, Odibo et al. adjusted four metabolites (hydroxyhexanoylcarnitine, phenylalanine, glutamate, alanine) for maternal variables (maternal body mass index, ethnicity, and diabetes) and the AUCs were similar with the four-metabolite model (0.82) or when only the three amino acids were used for predicting preeclampsia (0.81). Additionally, the four metabolites have demonstrated equal AUCs for predicting preeclampsia at any gestational age (0.82) or in cases of early onset disease (0.84). [35]In the 1st trimester, Shanmugalingam et al. [53] showed lower levels of 15-epilipoxin-A4, which is possibly involved with the aspirin mechanism of preeclampsia prophylaxis. Chappell et al. showed an increment in 8-epi-prostaglandin F2α from 20 to 24 weeks in the preeclampsia group. [23] Recently, Bilodeau et al. reinforced that F2-isoprostanes isomers from class VI were increased in the preeclampsia group. [54] The prostaglandins' metabolites are markers of lipid peroxidation, which pointed to an oxidative stress status, but presented modest accuracy. [23,54]

Rijvers et al. [48] and Khalil et al. [42] studied the metabolism of asymmetric dimethylarginine (ADMA), which is considered an important inhibitor of nitric oxide action. Rijvers et al. found an increase of L-arginine/ADMA ratio at 12w in the preeclampsia group, [48] and a consistent decrease in L-arginine between 16w and 20w in the preeclampsia compared to the gestational hypertension group. This can be clinically relevant since L-arginine supplementation is possible. [59] Khalil et al. found lower levels of L-arginine and L-homoarginine at 11-13w in the early-onset preeclampsia, and unchanged levels in the late-onset group, even after the excluding chronic hypertensive women. [42]

Koster et al. [46] showed that stearoylcarnitine was a common biomarker for both early-onset and late-onset preeclampsia. In a model composed of stearoylcarnitine, prior risk and MAP, the detection rate improved by 45% and 21%, for early and late-onset cases of preeclampsia, respectively. Dobierzewska et al. [26] have studied sphingolipids that have been related to trophoblast differentiation and invasion. In the ceramide profile, the Cer 14:0 was decreased in both 1st and 3rd trimesters of preeclampsia group, while Cer 16:0 was significantly increased in cross-gestational

For peer review only - http://bmjopen.bmj.com/site/about/guidelines.xhtml

preeclampsia plasma samples between 1st and 2nd trimesters. Plasma levels of dihydro-sphingosine-1 decreased from the 1st to the 2nd trimester among the preeclampsia group, and SM 18:0 increased through gestation of these participants. [26] Still studying ceramides, Huang et al [40] have found decreasing levels of Ceramide(d18:1/25:0) from 5-29 weeks, which increased the Leptin/ Ceramide(d18:1/25:0) ratio. Lee SM et al. proposed a model constituted by SM C28:1, SM C30:1, LysoPC C19:0, LysoPE C20:0 and propane-1,3-diol, achieving an AUC of 0.868 for identifying preeclampsia. [30]

Gong et al. [28] analysed serum samples in different moments of pregnancy, but it was at 36w when N1, N12 diacetylspermine were higher in women who later present preeclampsia, especially in the case of a female fetus. [28] This was the only study in common with our previous systematic review about SGA infant's prediction using metabolomics. [56]

Regarding targeted vitamin D analysis, all studies have applied LC-MS. [29,34,36,41,44,51] In general, they found lower serum vitamin D concentrations in women who later developed preeclampsia, both in blood [36] and urine, [29] suggesting that dysregulation of vitamin D metabolism occurs early in pregnancy. Indeed, Woodham et al. have pointed reduced odds of 38% of developing severe preeclampsia for a 10nmol/L increase in 25(OH)D level between 15-20w. [41] However, results did not reach statistical significance when a threshold was applied. In nested case-controls [34,44] or cohort [51] studies there were no differences between vitamin D status among preeclampsia group and controls. In the model proposed by Woodham et al. combining 25OHD, VEGF and sFLT-1/PlGF ratio, the AUC was 0.851, a higher value than either marker alone. [41] Vitamin D3 derivatives were found in the untargeted analysis of Kenny et al., [24] but upregulated in preeclampsia cases. [41]

Two studies have evaluated maternal exposure to bisphenol A and its metabolites. [27,47] The di(2-ethylhexyl) phthalate was increased in all trimesters. [47] Its hazard ratio for development of preeclampsia varied from 1.79 (95% CI 1.30-1.52), with 4-16 weeks, to 2.92 (95% CI 1.61-5.28) with 33-38w. The mono(2-ethyl-5-oxohexyl) phthalate, the mono(2-ethyl-5-carboxypentyl) phthalate and the Σdi(2-ethylhexyl)

For peer review only - http://bmjopen.bmj.com/site/about/guidelines.xhtml

phthalate were associated to increased hazard ratios for preeclampsia when evaluated in urine [47] from both the 1st and the 2nd half of pregnancy. When the findings were analysed according to fetal sex, exposure to bisphenol and di(2-ethylhexyl) phthalate was only associated to preeclampsia in pregnancies of female fetuses.[47] In maternal serum, [27] free bisphenol was higher in preeclamptic women compared to normotensive controls, even when superimposed preeclampsia cases were excluded. The cut-off of >4.4 µg/L was associated with increased odds of 16.46 (95% CI 5.42-49.95) of developing preeclampsia (adjusted for maternal age, parity, and body mass index). [27]

In another analysis of environmental factors, Eichelberger et al. found no differences in paraxanthine or caffeine levels, but the paraxanthine/caffeine ratio was lower in preeclampsia (0.23 versus; normotensive women, 0.37, p 0.02). [45] This represented a decreased risk of developing preeclampsia with increasing paraxanthine/caffeine ratio (odds ratio 0.53, 95%CI 0.31-0.90). Finally, regarding the perfluoroalkyl substances (PFASs), Rylander et al. [31] and Huo et al. [37] have found no differences between preeclamptic women and normotensive controls in blood samples from the 1st or 2nd trimester, respectively. However, levels of perfluorohexane sulfonate between 0.53-0.78ng/mL were associated to higher odds of preeclampsia (aOR 1.67, 95% CI1.02-2.74); [31] and of perfluoroheptanoic acid, to higher odds of gestational hypertension (aOR 1.38; 95%CI 1.01-1.87).

In the last study to evaluate gestational hypertension, Harville et al. [32] identified higher concentrations of 2,6-Di-tert-butyl-4-hydroxymethylphenol in blood samples between 6-14w. This metabolite is derived from 2,6-Di-tert-butyl-4-methylphenol, a synthetic phenolic antioxidant used widely in foods, polymers, and cosmetics to slow oxidation. It has been linked to induce cellular DNA damage.

Quality assessment and risk of bias

The synthesized data for all included studies is shown on **Figure 3**. Regarding the risk of bias, the majority of studies were rated low for 'Patient Selection' and 'Flow and Timing' domains. On the contrary, most studies were classified as unknown risk of bias

For peer review only - http://bmjopen.bmj.com/site/about/guidelines.xhtml

in 'Index Test' and 'Reference Standard' domains due to missing information whether diagnosis and metabolomics analysis were interpreted independently of each other. Virtually, all studies were ranked as low with regard to applicability, with minor exceptions. [32,47,49,53,60] (Supplementary Material 5).

Meta-analysis

We found that a third of included studies were designed around a prediction aim [23–25,27,35,38,41,50,52] and we extracted accuracy measures when possible (**Tables 2 and 3**). The other studies only presented statistical differences between groups. Unfortunately, there were discrepancies regarding type of maternal sample or gestational age at sampling, and metabolomics technique. Then, it was not possible to perform a meta-analysis.

DISCUSSION

Hypertensive disorders constitute one of the leading causes of maternal morbidity and mortality worldwide. In this systematic review, we have found that amino acids and fatty acids were the most common chemical subclasses associated with pre-eclampsia. Similarly, the main pathways involved in preeclampsia were lipid metabolism, mitochondrial beta-oxidation of short chain saturated fatty acids, and cell signalling (**Figure 4**). Our findings can improve disease understanding and contribute to future studies as a guide of pathways to be explored with the purpose to better treat the disease.

Amino acids and related compounds

Amino acids, peptides, and analogues were the most common chemical subclass. Taurine, glycine and glycylglycine were decreased in women who developed pre-eclampsia. [39,43] The function of taurine has been associated with cytoprotection, involving the placentation process and the spiral artery remodelling. Nevertheless, a reduction in taurine activity may be associated with impaired placentation, which is correlated with the pathophysiology of pre-eclampsia. [43] Glycylglycine is strongly associated with homocysteine levels and participates in the metabolic pathway that

For peer review only - http://bmjopen.bmj.com/site/about/guidelines.xhtml

antagonizes homocysteine: lower glycine levels promote higher homocysteine levels. Homocysteine is linked to cardiovascular risk and endothelial dysfunction.

Indeed, a reduction in arginine and homoarginine was observed among pre-eclampsia patients. [42] These are substrates for oxide nitric synthesis, a potent vasodilator with a major function in endothelial cells. ADMA is an inhibitor of the enzyme responsible for NO production; there is evidence that elevated ADMA levels are associated with endothelial dysfunction, and consequently, pre-eclampsia. [48,61] NO is an important regulator of trophoblast implantation, differentiation, motility, invasion and apoptosis. [42] Decreased levels of arginine and homoarginine in blood samples of pre-eclampsia patients reinforce the involvement of NO molecule in the aetiology of the disorder. [42,50]

Carnitine is a non-essential amino acid strongly associated with lipid metabolism. Its function is to bind to fatty acid, form acyl-carnitine, and shuttle it to mitochondria, to integrate the mitochondrial oxidative metabolism. [52] The increment of carnitine might be associated to the oxidative stress observed in pre-eclampsia. [24,25,35,46,49,52]

Lipids

Maternal serum lipids are markedly elevated in healthy pregnancies, probably a hormone- induced increase. [62] Preeclamptic women may have even higher levels. [63] The lipidome – the group of lipid metabolites - contains key mediators of vascular tone (sphingosine), inflammation (prostaglandins), and insulin sensitivity (free fatty acids). [24] Lipids are involved in the aetiology of pre-eclampsia either indirectly, as a substrate, or directly, as a disease mediator. [63] Lipid peroxides may cause endothelial cell activation and reinforce the action of diabetes mellitus and essential hypertension as important risk factors to pre-eclampsia. [23]

In a healthy pregnancy, the pro-angiogenic biochemical scenario, sphingosine-1-phosphate and its receptors are up-regulated. Ceramide levels are also increased, reflecting the trophoblastic apoptosis process, observed because of normal syncytial fusion of villous trophoblasts. [63] On the other hand, in pre-eclampsia, low levels of

For peer review only - http://bmjopen.bmj.com/site/about/guidelines.xhtml

sphingosine-1-phosphate (angiogenic) and high levels of ceramides (pro-apoptotic) were found in serum analyses of 3rd trimester pre-eclampsia cases. [26] The increase of sphingomyelin SM:18 might serve not only as atherogenic marker of pre-eclampsia development and progression, but also the marker of cardiovascular complications developed later in women who had previous preeclamptic pregnancies. Recent studies have demonstrated that serum elevation of sphingomyelin SM:18 positively correlates with parameters of insulin resistance and liver function in obese adults [64] and correlates with markers of NFkB activation and, thus, markers of intracellular inflammation. [64] The ceramides might be biomarkers for cardiovascular disease in later life, which points attention to its role in hypertension in pregnancy. [65] As ceramide and their synthesizing enzymes (e.g. ceramide synthases) have been shown to be significantly up-regulated in the mouse uterus during early gestation, [44,66] the role of ceramides in the normal human pregnancy and its relationship with pre-eclampsia etiopathogenesis needs to be further explored.

Pre-eclampsia has also been correlated with high amounts of circulating reactive oxygen species. Among the consequences of this, high levels of oxidized cholesterol and acylcarnitine can be attributable to oxidation impairment of phosphocoline and fatty acid, respectively. [66]

Glycerophosphocholines resulted from cell lysis, possibly correlated with apoptosis described in pre-eclampsia. For the propanoate pathway, late-onset pre-eclampsia showed higher concentrations of L- valine, 2-hydroxybutyric acid, lactic acid, acetone and propan-2-ol. [52] Decreased glycerol level has been regarded as an important biomarker of late-onset pre-eclampsia and this may be due to its conversion to triglycerides. [57,63]

Vitamin D

The included studies on vitamin D showed contradictory results. [29,34,36,41,44,51] The heterogeneity of results may be attributable to the measurement of only serum 25(OH)D3 vitamin D, which is considered simplistic. [29] Interestingly, all included studies have considered low risk women and have presented data irrespective of

For peer review only - http://bmjopen.bmj.com/site/about/guidelines.xhtml

gestational age of pre-eclampsia diagnosis. The active metabolite - 1,25-dihydroxyvitamin D - is thought to increase vascular endothelial growth factor transcription, [67] which is correlated to placenta functioning and adaptation. Besides, vitamin D deficiency may be associated with an increment of inflammatory cytokine levels, such as TNF-alpha, and to a tendency towards T-cell activity. [68] Thus, its deficiency could potentially contribute to the pathophysiology of pre-eclampsia: abnormal placental implantation, angiogenesis, excessive inflammation, immune dysfunction. [69] Accuracy performance of vitamin D was increased when combined with angiogenic biomarkers (VEGF + sFLT-1/PlGF).[41] Additionally, all targeted studies showed lower levels of vitamin D metabolites, both in serum and urine, while untargeted LC-MS evaluation observed upregulated levels. Then, based on our results, we cannot rule out vitamin D role in predicting pre-eclampsia in high-risk population or early- or late-onset of disease. It seems there is a place for investigating its role in composing a multifactorial model of prediction.

Environmental exposures

Knowledge of the actual contribution of endocrine disrupting chemicals (EDC), such as BPA, phthalates, and others to pre-eclampsia is relevant, given that behavioural attitude can change maternal exposure to these risk factors. It is hypothesized that BPA can cross the placenta and induce degeneration and necrosis of placental cells, disturbing angiogenesis, [47] and possibly acting through estrogenic-related receptor *gama.* Phthalates can affect placental gene expression determining decreased placental growth, [64] and may also cause a pro-inflammatory response and increase the oxidative stress. [70] Although their role in pre-eclampsia remains elusive, [27,31,47,60] we cannot rule out their role before pregnancy: BPA, phthalates and PFASs are widely present in many products of daily life, and usually have half-lives of many years.

Another environmental factor that was pointed in our review was caffeine. Caffeine metabolism occurs in the cytochrome P450 1A2 system, and the primary metabolite of dietary caffeine (80% of caffeine by-product) is the paraxanthine. The activity of this cytochrome can be demonstrated by the ratio of paraxanthine/caffeine. During pregnancy, the primary pathway for all caffeine clearance happens in maternal

For peer review only - http://bmjopen.bmj.com/site/about/guidelines.xhtml

cytochrome P450 1A2 system, as neither placenta nor the foetus demonstrates this cytochrome activity. [71] The decreased risk of severe pre-eclampsia with increasing paraxanthine/caffeine ratio suggests that faster caffeine metabolism may be associated with a lower risk of pre-eclampsia. [45] This is not exactly in accordance with other studies, which indicated a direct influence of caffeine levels on the risk of pre-eclampsia and failed to perform analysis of caffeine metabolism. [72] On the other hand, caffeine and its metabolites are antagonists of the adenosine A1 receptors, implicated in proximal tubular sodium reabsorption. [73] Although caffeine consumption may be associated to elevated blood pressure in the short term (≤3 hours), it does not represent a cardiovascular risk factor in the long term (2 weeks) for non-pregnant adults. [74] Future studies should consider daily intake of caffeine, which was not explored in the included study, and maternal smoking as potential bias since cigarette use induces the P450 1A2 system. [75]

Methodological quality

Although the majority of included studies were ranked having a low risk of bias and low concerns around applicability, there is still a place for improving data reporting in metabolomics. [76] Interpreting (or using) data from bench analyses in a clinical context requires the clear description of reference standards and index tests, and timing of index test analysis. As preeclampsia has a complex aetiology and is a multifactorial disease, improving patient selection should be a focus of attention in subsequent studies. Better accuracy could be achieved if data from low and high risk women were to be separated, or single versus multiple gestations.

Strengths and limitations

This systematic review can be useful as a guidance of metabolites and their performance as predictors of pre-eclampsia. We have applied a systematic review strategy in eight different electronic databases. Then, we presented possible biomarkers of pre-eclampsia that could ground future research in this area.

Unfortunately, there are limitations. The inclusion of case-control studies could add risk of bias, confounding and potential measurement error. However, we understand

For peer review only - http://bmjopen.bmj.com/site/about/guidelines.xhtml

this is an important strategy to biomarkers selection and performance evaluation steps in metabolomics [6] in perinatal research. There was great heterogeneity among included studies. *Per se*, metabolomics is an overly complex process, influenced by sample collection, storage and preparation, analytical platform applied, statistical tests performed, time of day collection, fasting or non-fasting state prior the samples. [6] Thus, results presented in the studies must consider all these variables. Because of this complexity and authentic heterogeneity of metabolomics, the lack of standardization of pre-eclampsia concept (early and late-onset pre-eclampsia) and time of sample collection represented a limitation to our study. Thus, the results could not be correlated with this important variable. Additionally, since only two studies evaluated pre-eclampsia prediction regarding fetal sex, our findings should be interpreted with caution.

CONCLUSION AND IMPLICATIONS FOR PRACTICE

We have presented a list of 122 different metabolites and their accuracy measures, when possible. They are mainly involved with ammonia recycling; amino acid and lipids metabolism; arachidonic acid metabolism; cell signalling; galactose, lactose, and nucleotide sugars metabolisms. Maternal blood seems to be the best specimen to predict early pre-eclampsia if sampled in the 1st trimester, and to predict late-onset pre-eclampsia in the 2nd trimester. Future metabolomics platforms should be as comprehensive as possible to evaluate the contributions of amino acids, peptides, fatty acid esters, and carbohydrates conjugates, as pre-eclampsia biomarkers.

Although individual metabolites have low predictive measures, they should be considered in multifactorial models of metabolites alone or in combination with other biomarkers. [25,33,38,41,43,46,50,52] Metabolite signature may contribute to further insights into the pathogenesis of pre-eclampsia and to design screening tests, which may contribute to early recognizing high risk women to hypertensive disorders In future, it is important to validate citrate and carnitines as common markers between early-onset and late-onset pre-eclampsia, considering the feasibility of universal screening. [25,49,50] Additionally, since pre-eclampsia and SGA are related to abnormal deep placentation, [77] it seems plausible to study both conditions

For peer review only - http://bmjopen.bmj.com/site/about/guidelines.xhtml

simultaneously, at least considering the metabolites found in common (sphinganine 1-phosphate, sphingosine 1-phosphate, tyrosine, vitamin D, N1, N12-diacetylspermine). [56]

References

1 Mol BWJ, Roberts CT, Thangaratinam S, *et al.* Pre-eclampsia. *Lancet* 2016;**387**:999–1011. doi:10.1016/S0140-6736(15)00070-7

2 L. C. *Chesley's Hypertensive Disorders in Pregnancy*. 4th ed. Elsevier 2015.

3 Roberts JM, Rich-Edwards JW, McElrath TF, *et al.* Subtypes of Preeclampsia: Recognition and Determining Clinical Usefulness. *Hypertension* 2021;:1430–41. doi:10.1161/HYPERTENSIONAHA.120.14781

4 Laganà AS, Favilli A, Triolo O, *et al.* Early serum markers of pre-eclampsia: are we stepping forward? *J Matern Fetal Neonatal Med* 2016;**29**:3019–23.

5 Nobakht M. Gh BF. Application of metabolomics to preeclampsia diagnosis. *Syst Biol Reprod Med* 2018;**64**:324–39. doi:10.1080/19396368.2018.1482968

6 Xia J, Broadhurst DI, Wilson M, *et al.* Translational biomarker discovery in clinical metabolomics: An introductory tutorial. *Metabolomics* 2013;**9**:280–99. doi:10.1007/s11306-012-0482-9

7 Guasch-Ferré M, Hruby A, Toledo E, *et al.* Metabolomics in prediabetes and diabetes: A systematic review and meta-analysis. *Diabetes Care* 2016;**39**:833–46. doi:10.2337/dc15-2251

8 Hoang G, Udupa S, Le A. *Application of metabolomics technologies toward cancer prognosis and therapy*. 1st ed. Elsevier Inc. 2019. doi:10.1016/bs.ircmb.2019.07.003

9 Mayrink J, Leite DFB, Costa ML, *et al.* Metabolomics for prediction of hypertension in pregnancy: A systematic review and meta-analysis protocol. *BMJ Open* 2020;**10**:1–5. doi:10.1136/bmjopen-2020-040652

10 Moher D, Liberati A, Tetzlaff J, *et al.* Preferred Reporting Items for Systematic Reviews and Meta-Analyses: the PRISMA statement. *Ann Intern Med* 2009;**151**:264–9. doi:10.1371/journal.pmed1000097

11 Tranquilli AL, Dekker G, Magee L, *et al.* The classification, diagnosis and management of the hypertensive disorders of pregnancy: A revised statement from the ISSHP. *Pregnancy Hypertens* 2014;**4**:97–104. doi:10.1016/j.preghy.2014.02.001

12 Brown MA, Hague WM, Higgins J, *et al.* The detection, investigation and management of hypertension in pregnancy: Executive summary. *Aust New Zeal J Obstet Gynaecol* 2000;**40**:133–8. doi:10.1111/j.1479-828X.2000.tb01136.x

13 ACOG Committee on Practice Bulletins--Obstetrics. ACOG practice bulletin. Diagnosis and management of preeclampsia and eclampsia. Number 33, January 2002. *Obstet*

For peer review only

For peer review only - http://bmjopen.bmj.com/site/about/guidelines.xhtml

Gynecol 2002;**99**:159–67. doi:10.1097/00006250-200201000-00028

14 ACOG. Hypertension in Pregnancy. *Obstet Gynecol* 2013;**122**:1122–31. doi:10.1097/01.AOG.0000437382.03963.88

15 Report of the National High Blood Pressure Education Program Working Group on High Blood Pressure in Pregnancy. *Am J Obstet Gynecol* 2000;**183**:S1–22. doi:10.1067/mob.2000.107928

16 Geraldo J, Ramos L, Sass N, *et al.* Preeclampsia: Definitions of Hypertensive States during Pregnancy Pathophysiological Foundations. *Rev Bras Ginecol Obs* 2017;**39**:496–512.https://pubmed.ncbi.nlm.nih.gov/28793357/

17 Whiting PF, Rutjes AWS, Westwood ME, *et al.* QUADAS-2: A Revised Tool for the Quality Assessment of Diagnostic Accuracy Studies. *Ann Intern Med* 2011;**155**:529–36. doi:10.7326/0003-4819-155-8-201110180-00009

18 Wishart DS, Feunang YD, Marcu A, *et al.* HMDB 4.0: The human metabolome database for 2018. *Nucleic Acids Res* 2018;**46**:D608–17. doi:10.1093/nar/gkx1089

19 Kanehisa M, Goto S. Kyoto Encyclopedia of Genes and Genomes. *Nucleic Acids Res* 2000;**1**:27–30.https://www.genome.jp/kegg/ (accessed 24 Feb 2022).

20 Fahy E, Sud M, Cotter D, *et al.* LIPID MAPS online tools for lipid research. *Nucleic Acids Res* 2007;**35**:606–12. doi:10.1093/nar/gkm324

21 Rutter CM, Gatsonis CA. A hierarchical regression approach to meta-analysis of diagnostic test accuracy evaluations. *Stat Med* 2001;**20**:2865–84. doi:10.1002/sim.942

22 Deeks JJ, Macaskill P, Irwig L. The performance of tests of publication bias and other sample size effects in systematic reviews of diagnostic test accuracy was assessed. *J Clin Epidemiol* 2005;**58**:882–93. doi:10.1016/j.jclinepi.2005.01.016

23 Chappell L. A longitudinal study of biochemical variables in women at risk of preeclampsia. *Am J Obstet Gynecol* 2002;**187**:127–36. doi:10.1067/mob.2002.122969

24 Kenny LC, Broadhurst DI, Dunn W, *et al.* Robust early pregnancy prediction of later preeclampsia using metabolomic biomarkers. *Hypertension* 2010;**56**:741–9. doi:10.1161/HYPERTENSIONAHA.110.157297

25 Bahado-Singh R, Poon LC, Yilmaz A, *et al.* Integrated Proteomic and Metabolomic prediction of Term Preeclampsia. *Sci Rep* 2017;**7**:1–10. doi:10.1038/s41598-017-15882-9

26 Dobierzewska A, Soman S, Illanes SE, *et al.* Plasma cross-gestational sphingolipidomic analyses reveal potential first trimester biomarkers of preeclampsia. *PLoS One* 2017;**12**:1–16. doi:10.1371/journal.pone.0175118

27 Ye Y, Zhou Q, Feng L, *et al.* Maternal serum bisphenol A levels and risk of pre-eclampsia: A nested case-control study. *Eur J Public Health* 2017;**27**:1102–7. doi:10.1093/eurpub/ckx148

28 Gong S, Sovio U, Aye IL, *et al.* Placental polyamine metabolism differs by fetal sex, fetal growth restriction, and preeclampsia. *JCI insight* 2018;**3**:1–15. doi:10.1172/jci.insight.120723

For peer review only

For peer review only - http://bmjopen.bmj.com/site/about/guidelines.xhtml

29 Tamblyn JA, Jenkinson C, Larner DP, *et al.* Serum and urine vitamin D metabolite analysis in early preeclampsia. *Endocr Connect* 2018;**7**:199–210. doi:10.1530/EC-17-0308

30 Lee SM, Kang Y, Lee EM, *et al.* Metabolomic biomarkers in midtrimester maternal plasma can accurately predict the development of preeclampsia. *Sci Rep* 2020;**10**:16142. doi:10.1038/s41598-020-72852-4

31 Rylander L, Lindh CH, Hansson SR, *et al.* Per- and Polyfluoroalkyl Substances in Early Pregnancy and Risk for Preeclampsia: A Case-Control Study in Southern Sweden. *Toxics* 2020;**8**. doi:10.3390/toxics8020043

32 Harville EW, Li Y-YY-Y, Pan K, *et al.* Untargeted analysis of first trimester serum to reveal biomarkers of pregnancy complications: a case-control discovery phase study. *Sci Rep* 2021;**11**:3468. doi:10.1038/s41598-021-82804-1

33 Kenny LC, Thomas G, Poston L, *et al.* Prediction of preeclampsia risk in first time pregnant women: Metabolite biomarkers for a clinical test. *PLoS One* 2020;**15**:1–19. doi:10.1371/journal.pone.0244369

34 Powe CE, Seely EW, Rana S, *et al.* First trimester vitamin D, vitamin D binding protein, and subsequent preeclampsia. *Hypertension* 2010;**56**:758–63. doi:10.1161/HYPERTENSIONAHA.110.158238

35 Odibo AO, Goetzinger KR, Odibo L, *et al.* First-trimester prediction of preeclampsia using metabolomic biomarkers: a discovery phase study. *Prenat Diagn* 2011;**31**:990–4.

36 Kiely ME, Zhang JY, Kinsella M, *et al.* Vitamin D status is associated with uteroplacental dysfunction indicated by pre-eclampsia and small-for-gestational-age birth in a large prospective pregnancy cohort in Ireland with low Vitamin D status. *Am J Clin Nutr* 2016;**104**:354–61. doi:10.3945/ajcn.116.130419

37 Huo X, Huang R, Gan Y, *et al.* Perfluoroalkyl substances in early pregnancy and risk of hypertensivedisorders of pregnancy: A prospective cohort study. *Environ Int* 2020;**138**. doi:10.1016/j.envint.2020.105656

38 Sovio U, McBride N, Wood AM, *et al.* 4-Hydroxyglutamate is a novel predictor of pre-eclampsia. *Int J Epidemiol* 2020;**49**:301–11. doi:10.1093/ije/dyz098

39 Kenny LC, Broadhurst DI, Hong W, *et al.* NMR Profiling in Early Pregnancy Reveals a Metabolomic Signature the Predicts the Subsequent Development Preeclampsia. *Reprod Sci* 2013;**20**:2641A. doi:10.1177/1933719113482088

40 Huang Q, Hao S, You J, *et al.* Early-pregnancy prediction of risk for pre-eclampsia using maternal blood leptin/ceramide ratio: Discovery and confirmation. *BMJ Open* 2021;**11**. doi:10.1136/bmjopen-2021-050963

41 Woodham PC, Brittain JE, Baker AM, *et al.* Midgestation maternal serum 25-hydroxyvitamin D level and soluble fms-like tyrosine kinase 1/placental growth factor ratio as predictors of severe preeclampsia. *Hypertension* 2011;**58**:1120–5. doi:10.1161/HYPERTENSIONAHA.111.179069

42 Khalil AA, Tsikas D, Akolekar R, *et al.* Asymmetric dimethylarginine, arginine and homoarginine at 11-13 weeks gestation and preeclampsia: A case-control study. *J Hum Hypertens* 2013;**27**:38–43. doi:10.1038/jhh.2011.109

For peer review only - http://bmjopen.bmj.com/site/about/guidelines.xhtml

43 Kuc S, Koster MPH, Pennings JLA, *et al.* Metabolomics profiling for identification of novel potential markers in early prediction of preeclampsia. *PLoS One* 2014;**9**. doi:10.1371/journal.pone.0098540

44 Wetta LA, Biggio JR, Cliver S, *et al.* Is midtrimester vitamin D status associated with spontaneous preterm birth and preeclampsia? *Am J Perinatol* 2014;**31**:541–6. doi:10.1055/s-0033-1356483

45 Eichelberger KY, Baker AM, Woodham PC, *et al.* Second-trimester maternal serum paraxanthine, CYP1A2 activity, and the risk of severe preeclampsia. *Obstet Gynecol* 2015;**126**:725–30. doi:10.1097/AOG.0000000000001041

46 Koster MPH, Vreeken RJ, Harms AC, *et al.* First-Trimester serum acylcarnitine levels to predict preeclampsia: A metabolomics approach. *Dis Markers* 2015;**2015**. doi:10.1155/2015/857108

47 Cantonwine DE, Meeker JD, Ferguson KK, *et al.* Urinary concentrations of bisphenol A and phthalate metabolites measured during pregnancy and risk of preeclampsia. *Environ Health Perspect* 2016;**124**:1651–5. doi:10.1289/EHP188

48 Rijvers CAH, Marzano S, Winkens B, *et al.* Early-pregnancy asymmetric dimethylarginine (ADMA) levels in women prone to develop recurrent hypertension. *Pregnancy Hypertens* 2013;**3**:118–23. doi:10.1016/j.preghy.2013.01.001

49 Diaz SO, Barros AS, Goodfellow BJ, *et al.* Second trimester maternal urine for the diagnosis of trisomy 21 and prediction of poor pregnancy outcomes. *J Proteome Res* 2013;**12**:2946–57. doi:10.1021/pr4002355

50 Bahado-Singh RO, Syngelaki A, Akolekar R, *et al.* Validation of metabolomic models for prediction of early-onset preeclampsia. *Am J Obstet Gynecol* 2015;**213**:530.e1-530.e10. doi:10.1016/j.ajog.2015.06.044

51 Ates S, Sevket O, Ozcan P, *et al.* Vitamin D status in the first-trimester: Effects of vitamin D deficiency on pregnancy outcomes. *Afr Health Sci* 2016;**16**:36–43. doi:10.4314/ahs.v16i1.5

52 Bahado-Singh RO, Syngelaki A, Mandal R, *et al.* Metabolomic determination of pathogenesis of late-onset preeclampsia. *J Matern Neonatal Med* 2017;**30**:658–64. doi:10.1080/14767058.2016.1185411

53 Shanmugalingam R, Wang X, Motum P, *et al.* The 15-Epilipoxin-A4 Pathway with Prophylactic Aspirin in PreventingPreeclampsia: A Longitudinal Cohort Study. *J Clin Endocrinol Metab* 2020;**105**. doi:10.1210/clinem/dgaa642

54 Bilodeau JF, Qin Wei S, Larose J, *et al.* Plasma F2-isoprostane class VI isomers at 12-18 weeks of pregnancy are associated with later occurrence of preeclampsia. *Free Radic Biol Med* 2015;**85**:282–7. doi:10.1016/j.freeradbiomed.2015.05.012

55 Gong S, Sovio U, Aye ILMH, *et al.* Placental polyamine metabolism differs by fetal sex, fetal growth restriction, and preeclampsia. *JCI Insight* 2018;**3**:1–15. doi:10.1172/jci.insight.120723

56 Leite DFB, Morillon A-C, Junior EF de M, *et al.* Examining the predictive accuracy of metabolomics for small for gestational age babies: a systematic review. *BMJ Open* 2019;**9**:e031238. doi:10.1136/ bmjopen-2019-031238

For peer review only - http://bmjopen.bmj.com/site/about/guidelines.xhtml

57 Bahado-Singh RO, Akolekar R, Mandal R, *et al.* First-trimester metabolomic detection of late-onset preeclampsia. *Am J Obstet Gynecol* 2013;**208**:58.e1-58.e7. doi:10.1016/j.ajog.2012.11.003

58 Nelson DB, Ziadie MS, McIntire DD, *et al.* Placental pathology suggesting that preeclampsia is more than one disease. *Am J Obstet Gynecol* 2014;**210**:66.e1-66.e7. doi:10.1016/j.ajog.2013.09.010

59 Khalaf D, Krüger M, Wehland M, *et al.* The effects of oral l-arginine and l-citrulline supplementation on blood pressure. *Nutrients* 2019;**11**:1–14. doi:10.3390/nu11071679

60 Huo X, Huang R, Gan Y, *et al.* Perfluoroalkyl substances in early pregnancy and risk of hypertensive disorders of pregnancy: A prospective cohort study. *Environ Int* 2020;**138**:1–7. doi:10.1016/j.envint.2020.105656

61 Levine RJ, Lam C, Qian C, *et al.* Soluble Endoglin and Other Circulating Antiangiogenic Factors in Preeclampsia. *N Engl J Med* 2006;**355**:992–1005.

62 Wang C, Kong L, Yang Y, *et al.* Recommended reference values for serum lipids during early and middle pregnancy: A retrospective study from China 11 Medical and Health Sciences 1114 Paediatrics and Reproductive Medicine. *Lipids Health Dis* 2018;**17**:1–16. doi:10.1186/s12944-018-0885-3

63 Wojcik-Baszko D, Charkiewicz K, Laudanski P. Role of dyslipidemia in preeclampsia—A review of lipidomic analysis of blood, placenta, syncytiotrophoblast microvesicles and umbilical cord artery from women with preeclampsia. *Prostaglandins Other Lipid Mediat* 2018;**139**:19–23. doi:10.1016/j.prostaglandins.2018.09.006

64 Aris A, Benali S, Ouellet A, *et al.* Potential Biomarkers of Preeclampsia: Inverse Correlation between Hydrogen Peroxide and Nitric Oxide Early in Maternal Circulation and at Term in Placenta of Women with Preeclampsia. *Placenta* 2009;**30**:342–7. doi:10.1016/j.placenta.2009.01.003

65 de Carvalho LP, Tan SH, Ow GS, *et al.* Plasma Ceramides as Prognostic Biomarkers and Their Arterial and Myocardial Tissue Correlates in Acute Myocardial Infarction. *JACC Basic to Transl Sci* 2018;**3**:163–75. doi:10.1016/j.jacbts.2017.12.005

66 Anand S, Young SA, Esplin MS, *et al.* Detection and confirmation of serum lipid biomarkers for preeclampsia using direct infusion mass spectrometry. *J Lipid Res* 2016;**57**:687–96. doi:10.1194/jlr.P064451

67 Cardus A, Panizo S, Encinas M, *et al.* 1,25-Dihydroxyvitamin D3 regulates VEGF production through a vitamin D response element in the VEGF promoter. *Atherosclerosis* 2009;**204**:85–9. doi:10.1016/j.atherosclerosis.2008.08.020

68 Baker AM, Haeri S, Camargo CA, *et al.* A nested case-control study of midgestation vitamin D deficiency and risk of severe preeclampsia. *J Clin Endocrinol Metab* 2010;**95**:5105–9. doi:10.1210/jc.2010-0996

69 Westwood M, Al-Saghir K, Finn-Sell S, *et al.* Vitamin D attenuates sphingosine-1-phosphate (S1P)-mediated inhibition of extravillous trophoblast migration. *Placenta* 2017;**60**:1–8. doi:10.1016/j.placenta.2017.09.009

70 Werner EF, Braun JM, Yolton K, *et al.* The association between maternal urinary phthalate concentrations and blood pressure in pregnancy: The HOME Study. *Environ*

For peer review only

Heal A Glob Access Sci Source 2015;**14**:1–9. doi:10.1186/s12940-015-0062-3

71 Grosso LM, Bracken MB. Caffeine metabolism, genetics, and perinatal outcomes: A review of exposure assessment considerations during pregnancy. *Ann Epidemiol* 2005;**15**:460–6. doi:10.1016/j.annepidem.2004.12.011

72 Bakker R, Steegers EAP, Raat H, *et al.* Maternal caffeine intake, blood pressure, and the risk of hypertensive complications during pregnancy. the generation R study. *Am J Hypertens* 2011;**24**:421–8. doi:10.1038/ajh.2010.242

73 Guessous I, Eap CB, Bochud M. Blood Pressure in Relation to Coffee and Caffeine Consumption. *Curr Hypertens Rep* 2014;**16**:1–9. doi:10.1007/s11906-014-0468-2

74 Mesas AE, Leon-Muñoz LM, Rodriguez-Artalejo F, *et al.* The effect of coffee on blood pressure and cardiovascular disease in hypertensive individuals: A systematic review and meta-analysis. *Am J Clin Nutr* 2011;**94**:1113–26. doi:10.3945/ajcn.111.016667

75 Dobrinas M, Cornuz J, Eap CB. Pharmacogenetics of CYP1A2 activity and inducibility in smokers and exsmokers. *Pharmacogenet Genomics* 2013;**23**:286–92. doi:10.1097/FPC.0b013e3283602e75

76 Goodacre R, Broadhurst D, Smilde AK, *et al.* Proposed minimum reporting standards for data analysis in metabolomics. *Metabolomics* 2007;**3**:231–41. doi:10.1007/s11306-007-0081-3

77 Brosens I, Pijnenborg R, Vercruysse L, *et al.* The 'great obstetrical syndromes' are associated with disorders of deep placentation. *Am J Obs Gynecol* 2011;**204**:193–201. doi:10.1016/j.ajog.2010.08.009.THE

For peer review only

For peer review only - http://bmjopen.bmj.com/site/about/guidelines.xhtml

Legends

Figures

Figure 1. Flow chart of study identification, screening, eligibility and inclusion.

Figure 2. Characterization of study selection regarding biological samples, sampling moment, and metabolomic analysis techniques applied

Figure 3. Assessment of risk of bias (left) and applicability concerns (right) of included studies.

Figure 4. Preeclampsia related metabolites, chemical classes, and biological processes.

Tables

Table 1. Characteristics of included studies.

Table 2. Metabolites and their accuracy measures for predicting preeclampsia according to gestational age of required delivery.

Table 3 – Accuracy measures of metabolites and other pregnancy-related biomarkers.

Supplementary Material

Supplementary material 1 Preferred Reported Items for Systematic Reviews and Meta-Analysis (PRISMA) statement: a PRISMA checklist

Supplementary material 2 – Full search strategy for each database.

Supplementary material 3- Excluded studies and reasons for exclusion.

Supplementary material 4 - Predictive metabolites of hypertensive disorders of pregnancy summarized according to their superclass, chemical class, subclass, and metabolic pathways.

Supplementary material 5 – Individual QUADAS-2 data for all included studies.

ACKNOWLEDGEMENTS

We are grateful to all members of the PRETERM-SAMBA study research group.

For peer review only - http://bmjopen.bmj.com/site/about/guidelines.xhtml

AUTHORS CONTRIBUTIONS

JM is the guarantor of this review. JM and DFBL elaborated the protocol, searched the literature, selected studies, extracted data, assessed risk of bias, and drafted the initial manuscript. MLC has helped in study inclusion, interpreting data, examining clinical applications of findings, and revising the manuscript. GN has helped in figures and tables elaboration and with the final version of the manuscript. JGC have supervised and approved all steps. All authors have read and agree with this submission.

FUNDING

DFBL (process number 88881.134512/2016-01) has awarded scholarship from Brazilian Federal Agency for Support and Evaluation of Graduate Education (CAPES). This research is part of the PRETERM-SAMBA study, which has granted sponsor from Brazilian National Research Council (CNPq) (Award 401636/2013-5) and from the Bill and Melinda Gates Foundation (grant OPP1107597). This specific analysis received no specific grant from any sectors. Our sponsors have not intervened in any step of this study design, development, or submission.

For peer review only

COMPETING INTERESTS

None to declare.

PATIENT CONSENT FOR PUBLICATION

Not required.

PROVENANCE AND PEER REVIEW

Not commissioned; externally peer reviewed.

DATA AVAILABILITY STATEMENT

No data are available.

For peer review only - http://bmjopen.bmj.com/site/about/guidelines.xhtml

1 **Table 1. Characteristics of included studies.**

Authors, year	Country of data collection	Study design	Classification of hypertensive syndrome	Comments
Chappell LC et al, 2002 [1]	United Kingdom	Case-control	ISSHP, 1988	17 women with preeclampsia; 21 without preeclampsia. Chronic hypertensive women included.
Powe CE et al, 2010 [2]	United States of America	Nested case-control	ICD codes	39 preeclampsia, 131 controls. Gestational diabetes excluded.
Kenny LC et al, 2010 [3]	New Zealand; Australia	Nested case-control	ASSHP, 2000	Discovery phase (New Zealand): 60 women with preeclampsia, 60 matched controls (age, ethnicity, body mass index) with uneventful pregnancies. Validation phase (Australia): 39 women with preeclampsia, 40 healthy matched pregnancies (SCOPE study).
Odibo AO et al, 2011 [4]	United States of America	Nested case-control	ACOG, 2002	41 women with preeclampsia, 41 normotensive controls. Pregestational diabetes included.
Woodham PC et al, 2011 [5]	United States of America	Nested case-control	ACOG, 2002	41 women with severe preeclampsia, 123 normotensive uncomplicated pregnancies at term. Chronic illness and multiple pregnancies excluded.
Rijvers CAH et al, 2013 [6]	Netherlands	Retrospective cohort	NHBP, 2000	06 women with preeclampsia, 11 with gestational hypertension, 18 normotensive women
Khalil AA et al, 2013 [7]	United Kingdom	Case-control	ISSHP, 2001	25 women with early preeclampsia, 50 cases of late preeclampsia, 300 normotensive controls (matching 1 case: 4 controls). Only singleton pregnancies; chronic hypertensive women included.
Diaz SO et al, 2013 [8]	Portugal	Prospective cohort	ACOG, 2002	09 women with preterm preeclampsia (31-37weeks); 84 women with healthy term pregnancies

For peer review only - http://bmjopen.bmj.com/site/about/guidelines.xhtml

Kenny LC et al, 2013 [9]	New Zealand; Australia	Nested case-control	ASSHP, 2000	49 women with preeclampsia and 49 normotensive nulliparous women. (SCOPE study).
Kuc S et al, 2014 [10]	Netherlands	Nested case-control	ISSHP, 2001	68 early-onset preeclampsia, 99 late-onset preeclampsia, 500 controls
Wetta LA et al, 2014 [11]	United States of America	Nested case-control	ACOG, 2002	89 women with preterm preeclampsia; 177 normotensive and term pregnancies
Bahado-Singh RO et al, 2015 [12]	United Kingdom	Prospective cohort	ISSHP, 2001	50 cases of early preeclampsia, 108 normotensive controls. Singleton pregnancies delivered at term; birthweight adequate for gestational age. HELLP syndrome cases excluded.
Eichelberger KY et al, 2015 [13]	United States of America	Nested case-control	ACOG, 2002	25 women with severe preeclampsia; 87 normotensive, healthy pregnancies delivered at term. Chronic hypertensive women and multiple pregnancies excluded.
Koster MPH et al, 2015 [14]	Netherlands	Nested case-control	ISSHP, 2001	68 early-onset preeclampsia, 99 late-onset preeclampsia, 500 controls
Bilodeau JF et al, 2015 [15]	Canada	Prospective cohort	CHS, 1997	33 preeclampsia, 60 controls (MIROS study)
Ates S et al, 2016 [16]	Turkey	Prospective cohort	ACOG, 2002	04 women with preeclampsia; 11 women with gestational hypertension; 214 normotensive women
Cantonwine DE et al, 2016 [17]	United States of America	Nested case-control	ACOG, 2002	50 women with preeclampsia, 431 without preeclampsia. Chronic hypertensive women included.
Kiely ME et al, 2016 [18]	Ireland	Nested case-control	ASSHP, 2000	68 preeclampsia, 1528 controls. SGA infants excluded from controls. (SCOPE Study)
Bahado-Singh RO et al,	United Kingdom	Prospective cohort	ISSHP, 2001	59 late-onset preeclampsia cases, 115 normotensive controls. Singleton

For peer review only - http://bmjopen.bmj.com/site/about/guidelines.xhtml

2017 [19]				pregnancies delivered at term; birthweight adequate for gestational age. HELLP syndrome cases excluded.
Bahado-Singh RO et al, 2017b [20]	United Kingdom	Nested case-control	ISSHP, 2001	35 term preeclampsia cases (≥37w), 65 normotensive controls. Singleton pregnancies, no major malformations; controls with birthweight adequate for gestational age.
Dobierzewska A et al, 2017 [21]	Chile	Case-control	ACOG, 2002	07 women with preeclampsia; 07 normotensive women
Ye Y et al, 2017 [22]	China	Nested case-control	ACOG, 2013	74 women with preeclampsia, 99 normotensive term pregnancies as controls. Only singleton pregnancies; chronic illness excluded in control group.
Gong S et al, 2018 [23]	United Kingdom	Case-control	ACOG, 2002	134 term preeclampsia, 259 normotensive controls. Nulliparous women, singleton pregnancies (POP Study).
Tamblyn JA et al, 2018 [24]	Ireland	Case-control	ASSHP, 2000	25 women with preeclampsia, 25 normotensive women (SCOPE Study).
Sovio U et al, 2020 [25]	United Kingdom	Case-cohort	ISSHP, 2001; ACOG, 2013	Training and test with POP Study: 194 women with preeclampsia, 323 controls. Validation within BiB Study: 95 women with preeclampsia, 953 normotensive controls.
Huo X et al, 2020 [26]	Shanghai, China	Case-Cohort	ACOG, 2013	64 gestational hypertension, 71 preeclampsia. Singleton gestation only; chronic hypertensive women excluded.
Rylander L et al, 2020 [27]	Sweden	Case-control	ICD codes	296 preeclampsia, 580 controls. Excluded pregnancies with SGA infants.
Lee SM et al, 2020 [28]	South Korea	Case-control	ACOG, 2013	33 preeclampsia, 66 controls. Singleton pregnancies, congenital

For peer review only - http://bmjopen.bmj.com/site/about/guidelines.xhtml

				malformations excluded.
Kenny L et al, 2020 [29]	Ireland and England	Nested case-control	ISSHP, 2018	97 preeclampsia (23 preterm preeclampsia and 74 term preeclampsia); 335 controls. Nulliparous women, singleton pregnancies. Chronic illness and fetal malformations excluded (SCOPE study)
Shanmugalingam R et al, 2020 [30]	Australia	Prospective cohort	ISSHP, 2018	21 preeclampsia, 103 controls. High risk women.
Harville EW et al, 2021 [31]	United States of America	Case-control	Not mentioned	18 preeclampsia, 109 controls. Only singleton pregnancies.
Huang Q et al, 2021 [32]	United States of America	Case-control	ACOG, 2013	20 preeclampsia, 20 controls (validation phase).

ACOG: American College of Obstetricians and Gynecologists; CHS, Canadian Hypertensive Society; ASSHP: Australasian Society of the Study of Hypertension in Pregnancy; ICD, International Classification of Diseases; BiB Study: Born in Bradford Study; ISSHP: International Society for the Study of Hypertension in Pregnancy; POP Study: Pregnancy Outcome Prediction; SCOPE Study: Screening for Pregnancy Endpoints; MIROS study: Maternal and Infant Research on Oxidative Stress; PTB: preterm birth; SGA, small for gestational age.

For peer review only

For peer review only - http://bmjopen.bmj.com/site/about/guidelines.xhtml

Table 2. Metabolites and their accuracy measures for predicting preeclampsia according to gestational age of required delivery.

Preeclampsia at any gestational age

Authors, year	Metabolomics methods	Maternal biological specimen; gestational age at sampling	Increased metabolites	Decreased metabolites	S/ Sp/ AUC (95% CI)
Chappell LC et al, 2002 [1]	GC-MS	Blood; 20w	Isoprostane 8-epi-prostaglandin F2α		-/ -/ 0.62 (0.44-0.81)
		Blood; 24w	Isoprostane 8-epi-prostaglandin F2α		-/ -/ 0.55 (0.35-0.75)
Powe CE et al, 2010 [2]	Targeted LC-MS/MS	Blood (serum); 11w (±3w)		25(OH)vitamin D [a]	-/ -/ -
Odibo AO et al, 2011 [4]	Targeted LC-MS/MS	Blood (serum); 11-14w	Hydroxyhexanoylcarnitine, phenylalanine, glutamate, alanine		-/ -/ 0.82 (0.80–0.85)
Woodham PC et al, 2011 [5]	Targeted LC-MS/MS	Blood (serum); 15-20w		25(OH)vitamin D	-/ -/ 0.745
Diaz SO et al, 2013 [8]	Untargeted ^{1}H-NMR	Urine; 14-26w	*N*-methyl-2-pyridone-5-carboxamide, 3-methylhistidine, 4-deoxyerythronic acid, *cis*-aconitate, citrate, Glutamine, hippurate, indoxyl sulphate, *N*-methylnicotinamide, sucrose, U35	2-ketoglutarate, 4-OH-hippurate, acetate, carnitine, creatinine, formate, fumarate, galactose, isoleucine, lactose, phenylacetylglutamine, *p*-cresol sulphate, *scyllo*-ino, succinate, trigonelline, tyrosine, U14, U4, U10	-/ -/ -
Kenny LC et al, 2013 [9]	Untargeted H-NMR	Blood (serum); 15w		Acetamide, Glutamine, Trimethylamine, creatinine, dimethyl sulfone, valine,	-/-/-

For peer review only - http://bmjopen.bmj.com/site/about/guidelines.xhtml

				isoleucine, ornithine, glycine, methionine, betaine, acetate, proline	
Rijvers CAH et al, 2013 [6]	Targeted UPLC-MS/MS	Blood (plasma); 12w	L-arginine/ Asymmetric dimethylarginine ratio		-/ -/ -
		Blood (plasma); 16-20w		L-arginine	-/ -/ -
Wetta LA et al, 2014 [11]	Targeted LC-MS/MS	Blood (serum); 15-21w		25(OH)vitamin D [a]	-/ -/ -
Bilodeau JF et al, 2015 [15]	Targeted HPLC-MS/MS	Blood (plasma); 12-18w	(+/-) 5-iPF2α-VI, (+/-) 5-iPF2α-VI-(d11)		-/ -/ 0.67
	GC-FID	Blood (plasma); 12-18w		Alfa-linolenic acid, stearidonic acid, eicosatrienoic acid, eicosatetraenoic acid, eicosapentaenoic acid, docosapentaenoic acid, docosahexaenoic acid	-/ -/ -
Ates S et al, 2016 [16]	Targeted LC-MS/MS	Blood (serum); 11-14w		Vitamin D (<10mg/dL)	0.50/0.54
Cantonwine DE et al, 2016 [17]	Targeted HPLC-MS	Urine; 4-16w	Total bisphenol A, Σ di(2-ethylhexyl) phthalate, mono-ethyl phthalate	%mono(2-ethylhexyl) phthalate	-/ -/ -
		Urine; 22-29w	mono(2-ethylhexyl) phthalate, Σ di(2-ethylhexyl) phthalate, mono(3-carboxypropyl) phthalate	monoisobutyl phthalate	-/ -/ -
		Urine; 33-38w	mono(2-ethylhexyl) phthalate, Σ di(2-ethylhexyl) phthalate, mono(3-		-/ -/ -

For peer review only - http://bmjopen.bmj.com/site/about/guidelines.xhtml

			carboxypropyl) phthalate		
Kiely ME et al, 2016 [18]	Targeted LC-MS/MS	Blood (serum); 14-16w		25(OH)D3, 3-epi-25(OH)D3, and 25(OH)D2 [a]	-/ -/ -
Dobierzewska A et al, 2017 [21]	Targeted HPLC-MS	Blood (plasma); 11-14w		Sphingomyelin 16:0, sphingomyelin 18:0, ceramide 14:0	-/ -/ -
		Blood (plasma); 32-36w		Ceramide 14:0, ceramide 24:0	-/ -/ -
Ye Y et al, 2017 [22]	Targeted LC-MS	Blood (serum); 16-20w	Free bisphenol A		-/ -/ 0.73 (0.65-0.81)
Tamblyn JA et al, 2018 [24]	Targeted LC-MS/MS	Urine; 14-16w		25(OH) vitamin D3; 24,25(OH)2 vitamin D3[a]	-/ -/ -
Huo X et al, 2020 [26]	HPLC/MS-MS	Blood (serum); 13-17w	Perfluorooctanate; perfluorooctane sulfonate; perfluorodecanoic acid; perfluoroundecanoic acid; perfluorononanoic acid; perfluorohexanesulfonate; perfluoroheptanoic acid; perfluorobutane sulfonate; perfluorododecanoic acid; perfluorooctane sulfonamide[a]		-/ -/ -
Rylander L et al, 2020 [27]	Targeted LC-MS/MS	Blood (serum); 12-14w	Perfluorohexane sulfonate (0.53–0.78 ng/mL)		0.38/ 0.67/ -
Lee SM et al, 2020 [28]	GC-TOF MS	Blood (plasma); 16-24w		Propane-1,3-diol	0.75/ 0.83/ 0.868 (0.844-0.891)
	LC-Orbitrap	Blood (plasma);	LysoPE C20:0; SM C30:1	LysoPC C19:0; SM C28:1	

For peer review only

For peer review only - http://bmjopen.bmj.com/site/about/guidelines.xhtml

	MS	16-24w			
Kenny L et al, 2020 [29]	Targeted LC-MS/MS	Blood (plasma); 14-16w	Dilinoleoyl-glycerol (DLG)		0.39/ 0.90/0.70 (0.59-0.82)
Shanmugalingam R et al, 2020 [30]	Targeted LC-MS/MS	Blood (plasma); 12w		15-epilipoxin-A4	
Harville EW et al, 2021 [31]	Untargeted UPLC-HR-MS	Blood (serum); 6-14w	Cerasinone; bolasterone, 6.30_477.7721m/z, 5.86_313.1070m/z[b]		-/ -/ 0.95
	NMR	Blood (serum); 6-14w		Asparagine, N,N-Dimethylglycine, Trimethylamine	-/ -/ -
Huang Q et al, 2021 [32]	LC-MS	Blood (serum); 5-15w		Cer(d18:1/25:0)	-/-/0.868
		Blood (serum); 16-29w		Cer(d18:1/25:0)	-/-/0.747
**Early onset preeclampsia (<34w)**					
Odibo AO et al, 2011 [4]	Targeted LC-MS/MS	Blood (serum); 11-14w	Hydroxyhexanoylcarnitine, phenylalanine, glutamate, alanine		-/ -/ 0.84
Khalil AA et al, 2013 [7]	Targeted GC-MS; GC-MS/MS	Blood (plasma); 11-13w	asymmetric dimethylarginine/ L-arginine ratio; asymmetric dimethylarginine/ L-homoarginine ratio	L-arginine, L-homoarginine	-/ -/ -
Kuc S et al, 2014 [10]	Targeted UPLC-MS/MS	Blood (serum); 8-13w		Taurine, asparagine	-/ -/ -
Bahado-Singh RO et al, 2015 [12]	Untargeted ^{1}H-NMR	Blood (serum); 11-13w	2-hydroxybutyrate, 3-hydroxyisovalerate, citrate	Acetone, glycerol	0.75/ 0.74/ 0.835 (0.769-0.941)

For peer review only - http://bmjopen.bmj.com/site/about/guidelines.xhtml

Eichelberger KY et al, 2015 [13]	LC-MS	Blood (serum); 16-19w		Paraxanthine/caffeine ratio	-/ -/ -
Koster MPH et al, 2015 [14]	Targeted UPLC-MS/MS	Blood (serum); 8-13w	Hexanoylcarnitine, octenoylcarnitine, octanoylcarnitine, decenoylcarnitine, decanoylcarnitine, dodecanoylcarnitine, lauroylcarnitine, tetradecenoylcarnitine, hexadecenoylcarnitine	Isobutyrylcarnitine, linoleylcarnitine, stearoylcarnitine	-/ -/ -
Sovio U et al, 2020 [25]	Untargeted UPLC-MS/MS	Blood (serum); 12w<br>Blood (serum); 20w<br>Blood (serum); 28w	4-hydroxyglutamate		-/-/0.673<br>-/-/0.731<br>-/-/0.765
**Late onset preeclampsia (≥34w)**					
Kenny LC et al, 2010 [3]	Untargeted UPLC-MS	Blood (plasma); 14-16w	Monosaccharide(s), decanoylcarnitine, oleic acid, docosahexaenoic acid and/or docosatriynoic acid, gama-butyrolactone and/or oxolan-3-one, 2-oxovaleric acid and/or oxo-methylbutanoic acid, acetoacetic acid, hexadecenoyleicosatetraenoyl-Sn-glycerol, Di-(octadecadienoyl)-sn-glycerol, sphingosine 1-phosphate, sphinganine 1-phosphate, vitamin D3 derivatives	5-Hydroxytryptophan, methylglutaric acid and/or adipic acid	-/ -/ 0.92[c]

For peer review only - http://bmjopen.bmj.com/site/about/guidelines.xhtml

Kuc S et al, 2014 [10]	Targeted UPLC-MS/MS	Blood (serum); 8-13w		Glycylglycine	-/ -/ -
Koster MPH et al, 2015 [14]	Targeted UPLC-MS/MS	Blood (serum); 8-13w	Hexanoylcarnitine, octenoylcarnitine, octanoylcarnitine, decenoylcarnitine, decanoylcarnitine, dodecanoylcarnitine, tetradecenoylcarnitine	Linoleylcarnitine, palmitoylcarnitine, oleylcarnitine, stearoylcarnitine	-/ -/ -
Cantonwine DE et al, 2016 [17]	Targeted HPLC-MS	Urine; 4-16w	mono(2- ethyl-5-oxohexyl) phthalate, mono(2-ethyl-5-carboxypentyl) phthalate, Σ di(2-ethylhexyl) phthalate, mono-ethyl phthalate		-/ -/ -
		Urine; 33-38w	mono(2- ethyl-5-oxohexyl) phthalate, mono(2-ethyl-5-carboxypentyl) phthalate, Σ di(2-ethylhexyl) phthalate, Mono-*n*-butyl phthalate		-/ -/ -
Bahado-Singh RO et al, 2017 [19]	Untargeted ^{1}H-NMR	Blood (serum); 11-13w	Carnitine, pyruvate, acetone	-	0.30/ 0.80/ 0.629 (0.490-0.767)
Bahado-Singh RO et al, 2017b [20]	Untargeted ^{1}H-NMR, targeted LC-MS/MS	Blood (serum); 11-13w	-	Putrescine (LC-MS); urea, carnitine (H-NMR)	0.72/ 0.57/ 0.701
		Blood (serum); 32-33w	Methylhistidine, propylene glycol (H-NMR), citrate, hexose[d]	Serotonin (LC-MS)	0.74/ 0.72/ 0.761
Gong S et al, 2018	Untargeted	Blood (serum); 36w	N1, N12-diacetylspermine[e]	-	-

For peer review only - http://bmjopen.bmj.com/site/about/guidelines.xhtml

[23]	LC-MS/MS				
**Gestational hypertension**					
Ates S et al, 2016 [16]	Targeted LC-MS/MS	Blood (serum); 11-14w		Vitamin D (<10mg/dL)	0.45/0.54
Huo X et al, 2020 [26]	HPLC/ MS-MS	Blood (serum); 13-17w	Perfluoroheptanoic acid[f]		-/ -/ -
Harville EW et al, 2021 [31]	Untargeted UPLC-HR-MS	Blood (serum); 6-14w	Neomenthol-glucuronide, dibenzylamine, pilocarpine, 2,6-Di-*tert*-butyl-4-hydroxymethylphenol, 0.59_746.6045n, 8.66_762.1452m/z, 12.74_412.2842m/z		-/ - / 0.95[b]

AUC: area under the curve; w: weeks; GC-MS: gas-chromatography coupled to mass spectrometry; GC-FID: gas-chromatography coupled with a flame ionization detector; ^{1}H-NMR: hydrogen nuclear magnetic resonance; HPLC-MS: High Performance Liquid Chromatography- mass spectrometry; LC-MS: liquid- chromatography coupled to mass spectrometry; GC-TOF MS: gas chromatography time-of-flight mass spectrometry; LC-Orbitrap MS: liquid chromatography Orbitrap mass spectrometry. S, sensitivity; Sp, specificity; UPLC-MS: ultra- Performance Liquid Chromatography- mass spectrometry; 95% CI, 95% confidence interval.

[a]No statistical difference between preeclampsia and normotensive controls. [b]AUC for all metabolites model; some of them were not identified, thus they are presented as retention time and mass; it is unclear which metabolite was up or downregulated in preeclampsia cases. [c]Accuracy measures for the validation phase. [d]Unclear type of platform that has found this metabolite, or if it is increased or decreased in preeclampsia. [e]Higher preeclampsia risk with increasing N1, N12-diacetylspermine concentrations. [f] No statistical difference between preeclampsia normotensive controls.

For peer review only - http://bmjopen.bmj.com/site/about/guidelines.xhtml

Table 3 – Accuracy measures of metabolites and other pregnancy-related biomarkers.

Maternal specimen	Trimester of pregnancy	Metabolites	Additional variables considered in prediction models	AUC of metabolites + additional variables model (95% CI)
**Preeclampsia at any gestational age**				
Blood	1st	Dilinoleoyl-glycerol heptadecanoyl-2-hydroxy-sn-glycero-3-phosphocholine	PlGF	0.78 (0.69-0.88) [29]
		Ceramide(d18:1/25:0)[a]	Leptin	0.876 [32]
Blood	2nd	25OHD	VEGF + sFLT-1/PlGF	0.851 [5]
		Ceramide(d18:1/25:0)[a]	Leptin	0.892 [32]
Blood	3rd	4-hydroxyglutamate	s-Flt-1/PlGF	0.834 [25]
**Early onset preeclampsia (delivery <34w)**				
Blood	1st	Taurine	Prior risk+ MAP	0.93 [10]
		Stearoylcarnitine	Prior risk+ MAP	0.747 [14]
		3-hydroxyisovalerate, arginine, glycerol	UtPI	0.917 [12]
**Late onset preeclampsia (delivery ≥34w)**				
Blood	1st	Pyruvate, carnitine, glycerol	UtPI	0.722 [19]
Blood	1st	Stearoylcarnitine	Prior risk+ MAP	0.828 [14]
Blood	1st and 3rd	Urea, SM C18:1 (1st), Hexose, Citrate (3rd)	Maternal BMI (12w) + MAP (32w)	0.805 [20]

AUC: area under the curve; BMI: body mass index; MAP, mean arterial pressure; UtPI: uterine arteries pulsatility index; CI: confidence interval.

[a]Increased leptin/Ceramide(d18:1/25:0) ratio in preeclamptic women.

For peer review only

References

1 Chappell L. A longitudinal study of biochemical variables in women at risk of preeclampsia. *Am J Obstet Gynecol* 2002;**187**:127–36. doi:10.1067/mob.2002.122969

2 Powe CE, Seely EW, Rana S, *et al.* First trimester vitamin D, vitamin D binding protein, and subsequent preeclampsia. *Hypertension* 2010;**56**:758–63. doi:10.1161/HYPERTENSIONAHA.110.158238

3 Kenny LC, Broadhurst DI, Dunn W, *et al.* Robust early pregnancy prediction of later preeclampsia using metabolomic biomarkers. *Hypertension* 2010;**56**:741–9. doi:10.1161/HYPERTENSIONAHA.110.157297

4 Odibo AO, Goetzinger KR, Odibo L, *et al.* First-trimester prediction of preeclampsia using metabolomic biomarkers: a discovery phase study. *Prenat Diagn* 2011;**31**:990–4.

5 Woodham PC, Brittain JE, Baker AM, *et al.* Midgestation maternal serum 25-hydroxyvitamin D level and soluble fms-like tyrosine kinase 1/placental growth factor ratio as predictors of severe preeclampsia. *Hypertension* 2011;**58**:1120–5. doi:10.1161/HYPERTENSIONAHA.111.179069

6 Rijvers CAH, Marzano S, Winkens B, *et al.* Early-pregnancy asymmetric dimethylarginine (ADMA) levels in women prone to develop recurrent hypertension. *Pregnancy Hypertens* 2013;**3**:118–23. doi:10.1016/j.preghy.2013.01.001

7 Khalil AA, Tsikas D, Akolekar R, *et al.* Asymmetric dimethylarginine, arginine and homoarginine at 11-13 weeks gestation and preeclampsia: A case-control study. *J Hum Hypertens* 2013;**27**:38–43. doi:10.1038/jhh.2011.109

8 Diaz SO, Barros AS, Goodfellow BJ, *et al.* Second trimester maternal urine for the diagnosis of trisomy 21 and prediction of poor pregnancy outcomes. *J Proteome Res* 2013;**12**:2946–57. doi:10.1021/pr4002355

9 Kenny LC, Broadhurst DI, Hong W, *et al.* NMR Profiling in Early Pregnancy Reveals a Metabolomic Signature the Predicts the Subsequent Development Preeclampsia. *Reprod Sci* 2013;**20**:2641A. doi:10.1177/1933719113482088

10 Kuc S, Koster MPH, Pennings JLA, *et al.* Metabolomics profiling for identification of novel potential markers in early prediction of preeclampsia. *PLoS One* 2014;**9**. doi:10.1371/journal.pone.0098540

For peer review only

For peer review only - http://bmjopen.bmj.com/site/about/guidelines.xhtml

11 Wetta LA, Biggio JR, Cliver S, *et al.* Is midtrimester vitamin D status associated with spontaneous preterm birth and preeclampsia? *Am J Perinatol* 2014;**31**:541–6. doi:10.1055/s-0033-1356483

12 Bahado-Singh RO, Syngelaki A, Akolekar R, *et al.* Validation of metabolomic models for prediction of early-onset preeclampsia. *Am J Obstet Gynecol* 2015;**213**:530.e1-530.e10. doi:10.1016/j.ajog.2015.06.044

13 Eichelberger KY, Baker AM, Woodham PC, *et al.* Second-trimester maternal serum paraxanthine, CYP1A2 activity, and the risk of severe preeclampsia. *Obstet Gynecol* 2015;**126**:725–30. doi:10.1097/AOG.0000000000001041

14 Koster MPH, Vreeken RJ, Harms AC, *et al.* First-Trimester serum acylcarnitine levels to predict preeclampsia: A metabolomics approach. *Dis Markers* 2015;**2015**. doi:10.1155/2015/857108

15 Bilodeau JF, Qin Wei S, Larose J, *et al.* Plasma F2-isoprostane class VI isomers at 12-18 weeks of pregnancy are associated with later occurrence of preeclampsia. *Free Radic Biol Med* 2015;**85**:282–7. doi:10.1016/j.freeradbiomed.2015.05.012

16 Ates S, Sevket O, Ozcan P, *et al.* Vitamin D status in the first-trimester: Effects of vitamin D deficiency on pregnancy outcomes. *Afr Health Sci* 2016;**16**:36–43. doi:10.4314/ahs.v16i1.5

17 Cantonwine DE, Meeker JD, Ferguson KK, *et al.* Urinary concentrations of bisphenol A and phthalate metabolites measured during pregnancy and risk of preeclampsia. *Environ Health Perspect* 2016;**124**:1651–5. doi:10.1289/EHP188

18 Kiely ME, Zhang JY, Kinsella M, *et al.* Vitamin D status is associated with uteroplacental dysfunction indicated by pre-eclampsia and small-for-gestational-age birth in a large prospective pregnancy cohort in Ireland with low Vitamin D status. *Am J Clin Nutr* 2016;**104**:354–61. doi:10.3945/ajcn.116.130419

19 Bahado-Singh RO, Syngelaki A, Mandal R, *et al.* Metabolomic determination of pathogenesis of late-onset preeclampsia. *J Matern Neonatal Med* 2017;**30**:658–64. doi:10.1080/14767058.2016.1185411

20 Bahado-Singh R, Poon LC, Yilmaz A, *et al.* Integrated Proteomic and Metabolomic prediction of Term Preeclampsia. *Sci Rep* 2017;**7**:1–10. doi:10.1038/s41598-017-15882-9

21 Dobierzewska A, Soman S, Illanes SE, *et al.* Plasma cross-gestational sphingolipidomic analyses reveal potential first trimester biomarkers of preeclampsia. *PLoS One* 2017;**12**:1–16. doi:10.1371/journal.pone.0175118

For peer review only

22 Ye Y, Zhou Q, Feng L, *et al.* Maternal serum bisphenol A levels and risk of pre-eclampsia: A nested case-control study. *Eur J Public Health* 2017;**27**:1102–7. doi:10.1093/eurpub/ckx148

23 Gong S, Sovio U, Aye ILMH, *et al.* Placental polyamine metabolism differs by fetal sex, fetal growth restriction, and preeclampsia. *JCI Insight* 2018;**3**:1–15. doi:10.1172/jci.insight.120723

24 Tamblyn JA, Jenkinson C, Larner DP, *et al.* Serum and urine vitamin D metabolite analysis in early preeclampsia. *Endocr Connect* 2018;**7**:199–210. doi:10.1530/EC-17-0308

25 Sovio U, McBride N, Wood AM, *et al.* 4-Hydroxyglutamate is a novel predictor of pre-eclampsia. *Int J Epidemiol* 2020;**49**:301–11. doi:10.1093/ije/dyz098

26 Huo X, Huang R, Gan Y, *et al.* Perfluoroalkyl substances in early pregnancy and risk of hypertensivedisorders of pregnancy: A prospective cohort study. *Environ Int* 2020;**138**. doi:10.1016/j.envint.2020.105656

27 Rylander L, Lindh CH, Hansson SR, *et al.* Per- and Polyfluoroalkyl Substances in Early Pregnancy and Risk for Preeclampsia: A Case-Control Study in Southern Sweden. *Toxics* 2020;**8**. doi:10.3390/toxics8020043

28 Lee SM, Kang Y, Lee EM, *et al.* Metabolomic biomarkers in midtrimester maternal plasma can accurately predict the development of preeclampsia. *Sci Rep* 2020;**10**:16142. doi:10.1038/s41598-020-72852-4

29 Kenny LC, Thomas G, Poston L, *et al.* Prediction of preeclampsia risk in first time pregnant women: Metabolite biomarkers for a clinical test. *PLoS One* 2020;**15**:1–19. doi:10.1371/journal.pone.0244369

30 Shanmugalingam R, Wang X, Motum P, *et al.* The 15-Epilipoxin-A4 Pathway with Prophylactic Aspirin in PreventingPreeclampsia: A Longitudinal Cohort Study. *J Clin Endocrinol Metab* 2020;**105**. doi:10.1210/clinem/dgaa642

31 Harville EW, Li Y-YY-Y, Pan K, *et al.* Untargeted analysis of first trimester serum to reveal biomarkers of pregnancy complications: a case-control discovery phase study. *Sci Rep* 2021;**11**:3468. doi:10.1038/s41598-021-82804-1

32 Huang Q, Hao S, You J, *et al.* Early-pregnancy prediction of risk for pre-eclampsia using maternal blood leptin/ceramide ratio: Discovery and confirmation. *BMJ Open* 2021;**11**. doi:10.1136/bmjopen-2021-050963

For peer review only - http://bmjopen.bmj.com/site/about/guidelines.xhtml

For peer review only

For peer review only - http://bmjopen.bmj.com/site/about/guidelines.xhtml

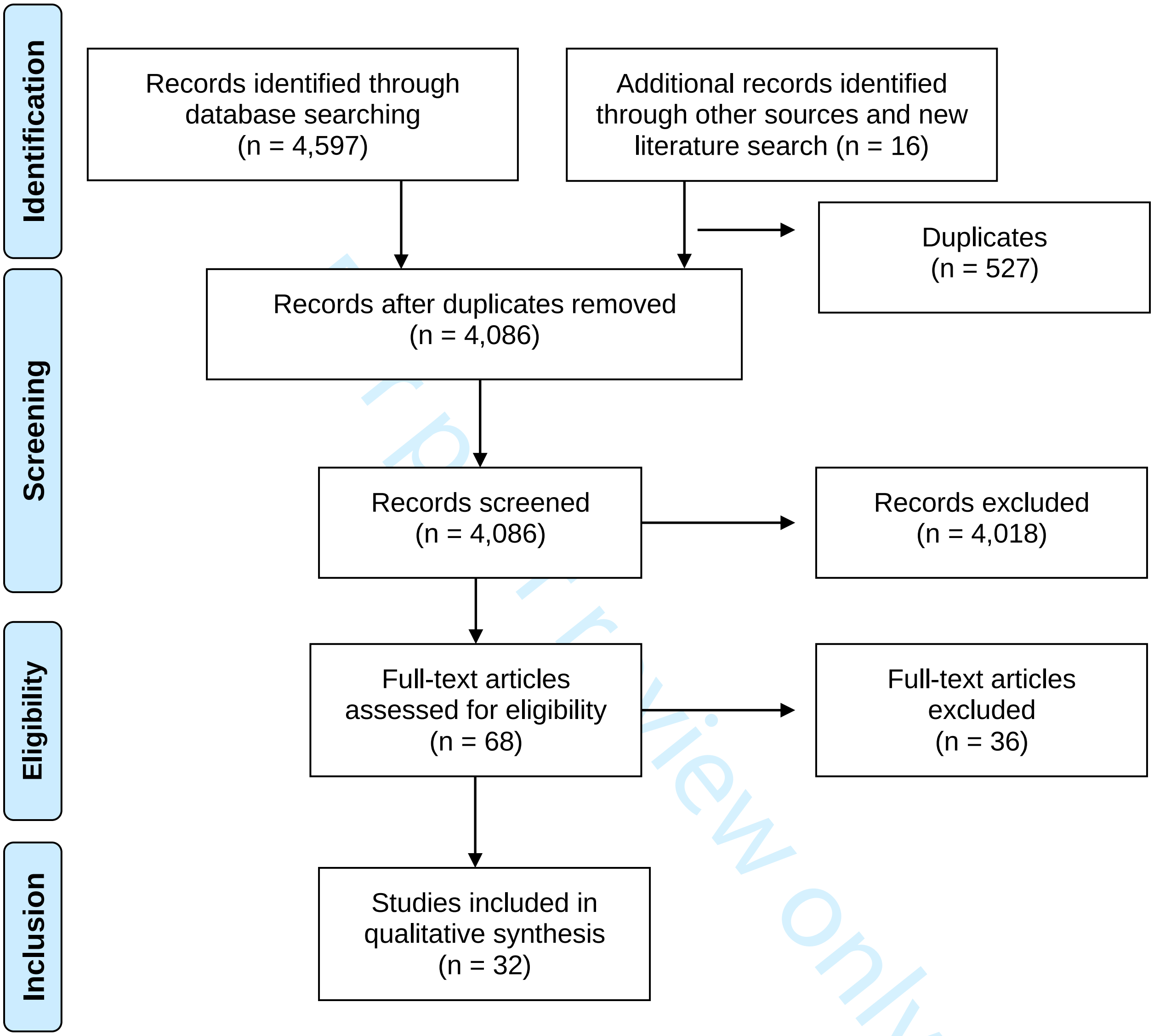

From: Moher D, Liberati A, Tetzlaff J, Altman DG, The PRISMA Group (2009). *P*referred *R*eporting *I*tems for *S*ystematic Reviews and *M*eta-*A*nalyses: The PRISMA Statement. PLoS Med 6(7): e1000097. doi:10.1371/journal.pmed1000097

For more information, visit www.prisma-statement.org.

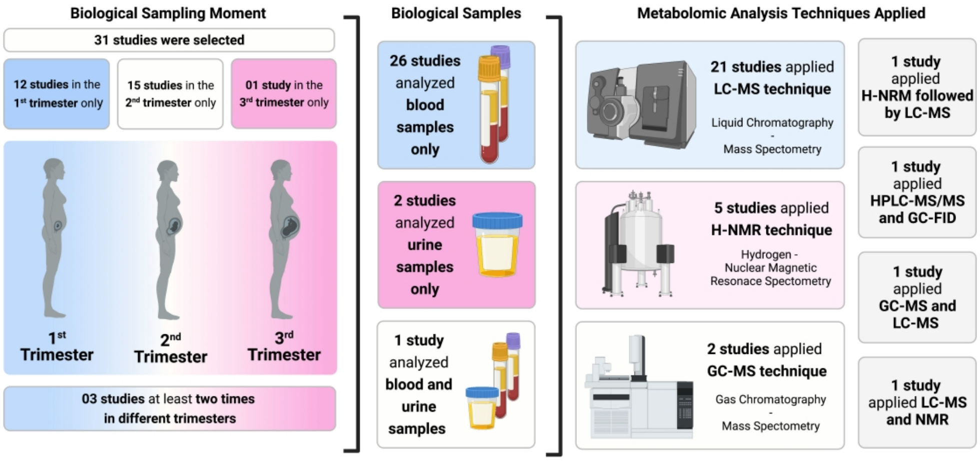

Figure 2

419x199mm (300 x 300 DPI)

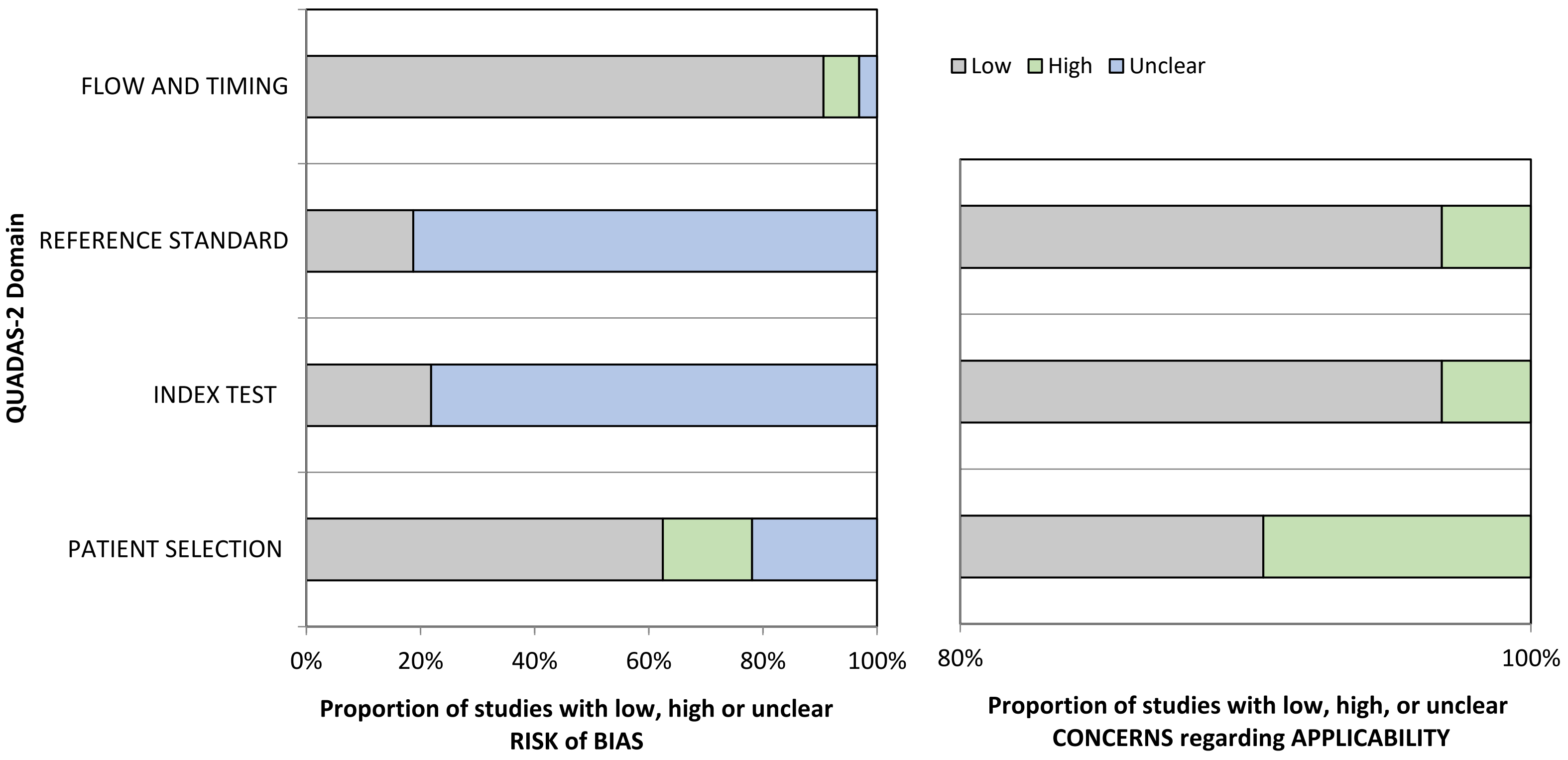
QUADAS-2 Domain
FLOW AND TIMING
REFERENCE STANDARD
INDEX TEST
PATIENT SELECTION
Low
High
Unclear
0%
20%
40%
60%
80%
100%
80%
100%
Proportion of studies with low, high or unclear RISK of BIAS
Proportion of studies with low, high, or unclear CONCERNS regarding APPLICABILITY

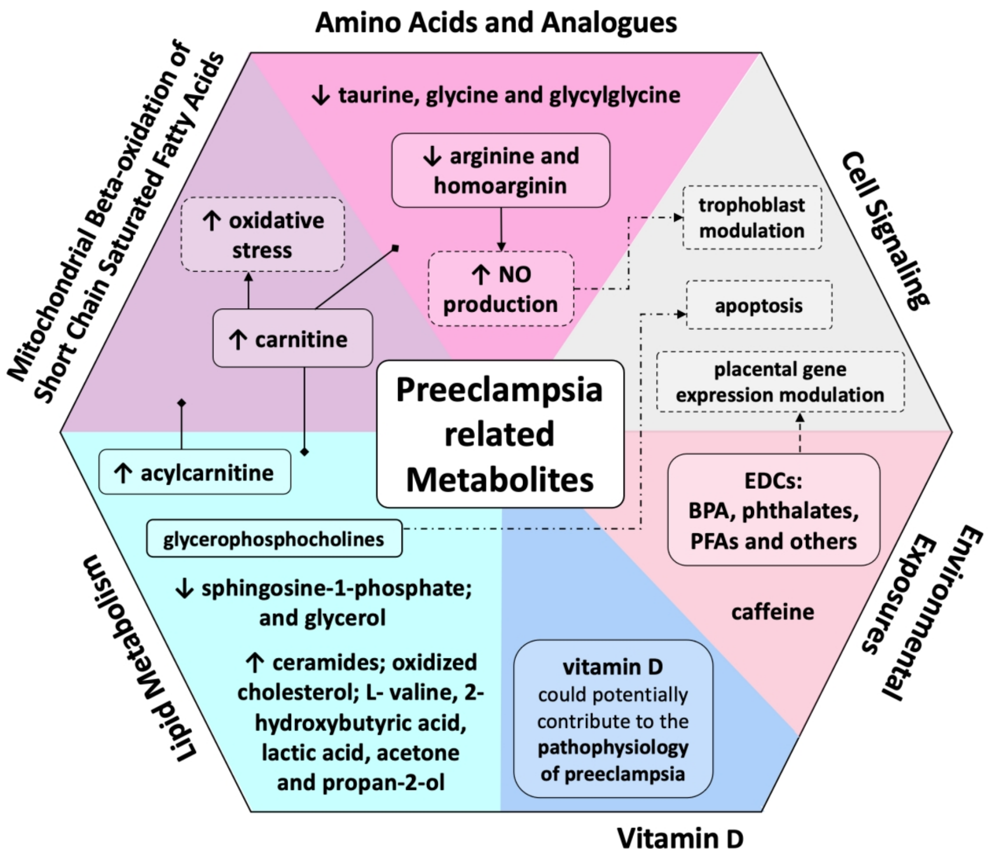

Figure 4

355x313mm (144 x 144 DPI)

Prediction of pregnancy-related hypertensive disorders using metabolomics: a systematic review

Jussara Mayrink, Debora Farias B. Leite, Guilherme M Nóbrega, Maria Laura Costa, José Guilherme Cecatti

Supplementary material 1 – PRISMA checklist

Section/topic	#	Checklist item	Section where the item was reported
**Title**			
Title	1	Identify the report as a systematic review, meta-analysis, or both.	Page 1/title
**ABSTRACT**			
Structured summary	2	Provide a structured summary including, as applicable: background; objectives; data sources; study eligibility criteria, participants, and interventions; study appraisal and synthesis methods; results; limitations; conclusions and implications of key findings; systematic review registration number.	Page 2/abstract
**INTRODUCTION**			
Rationale	3	Describe the rationale for the review in the context of what is already known.	Page 4
Objectives	4	Provide an explicit statement of questions being addressed with reference to participants, interventions, comparisons, outcomes, and study design (PICOS).	Page 5; Lines 11-12
**METHODS**			
Protocol and registration	5	Indicate if a review protocol exists, if and where it can be accessed (e.g., Web address), and, if available, provide registration information including registration number.	Page 3, Line 3<br>Page 5, Line 15
Eligibility criteria	6	Specify study characteristics (e.g., PICOS, length of follow-up) and report characteristics (e.g., years considered, language, publication status) used as criteria for eligibility, giving rationale.	Page 5, Lines 20-28<br>Page 6, Lines 1-13<br>Page 7, Lines 6-13
Information sources	7	Describe all information sources (e.g., databases with dates of coverage, contact with study authors to identify additional studies) in the search and date last searched.	Page 6, Lines 14-23<br>Page 8, Lines 10-11
Search	8	Present full electronic search strategy for at least one database, including any limits used, such that it could be repeated.	Supplementary Material 2
Study selection	9	State the process for selecting studies (i.e., screening, eligibility, included in systematic review, and, if applicable, included in the meta-analysis).	Page 7, Lines 16-27<br>Page 8, Lines 1-7
Data collection process	10	Describe method of data extraction from reports (e.g., piloted forms, independently, in duplicate) and any processes for obtaining and confirming data from investigators.	Page 7, Lines 16-27<br>Page 8, Lines 1-7
Data items	11	List and define all variables for which data were sought (e.g., PICOS, funding sources) and any assumptions and simplifications made.	Page 7, Lines 16-27<br>Page 8, Lines 1-7
Risk of bias in individual studies	12	Describe methods used for assessing risk of bias of individual studies (including specification of whether this was done at the study or outcome level), and how this information is to be used in any data synthesis.	Page 7, Lines 16-20
Summary measures	13	State the principal summary measures (e.g., risk ratio, difference in means).	Page 8, Lines 3-7

For peer review only

Section/topic	#	Checklist item	Section where the item was reported
Synthesis of results	14	Describe the methods of handling data and combining results of studies, if done, including measures of consistency (e.g., I^2) for each meta-analysis.	NA
Risk of bias across studies	15	Specify any assessment of risk of bias that may affect the cumulative evidence (e.g., publication bias, selective reporting within studies).	NA
Additional analyses	16	Describe methods of additional analyses (e.g., sensitivity or subgroup analyses, meta-regression), if done, indicating which were pre-specified.	NA
RESULTS			
Study selection	17	Give numbers of studies screened, assessed for eligibility, and included in the review, with reasons for exclusions at each stage, ideally with a flow diagram.	Page 8, Lines 11-27
Study characteristics	18	For each study, present characteristics for which data were extracted (e.g., study size, PICOS, follow-up period) and provide the citations.	Page 11, Line 11 – Page 15, Line 5
Risk of bias within studies	19	Present data on risk of bias of each study and, if available, any outcome level assessment (see item 12).	Page 15, Lines 6-15
Results of individual studies	20	For all outcomes considered (benefits or harms), present, for each study: (a) simple summary data for each intervention group (b) effect estimates and confidence intervals, ideally with a forest plot.	NA
Synthesis of results	21	Present results of each meta-analysis done, including confidence intervals and measures of consistency.	NA
Risk of bias across studies	22	Present results of any assessment of risk of bias across studies (see Item 15).	NA
Additional analysis	23	Give results of additional analyses, if done (e.g., sensitivity or subgroup analyses, meta-regression [see Item 16]).	NA
**DISCUSSION**			
Summary of evidence	24	Summarize the main findings including the strength of evidence for each main outcome; consider their relevance to key groups (e.g., healthcare providers, users, and policy makers).	Page 16, Line 6 – Page 19, Line 30
Limitations	25	Discuss limitations at study and outcome level (e.g., risk of bias), and at review-level (e.g., incomplete retrieval of identified research, reporting bias).	Page 20, Lines 15-28
Conclusions	26	Provide a general interpretation of the results in the context of other evidence, and implications for future research.	Page 21, Lines 1-20
**FUNDING**			
Funding	27	Describe sources of funding for the systematic review and other support (e.g., supply of data); role of funders for the systematic review.	Page 29, Lines 8-15

For peer review only - http://bmjopen.bmj.com/site/about/guidelines.xhtml

Supplementary material 2 – Full search strategy for each database.

PUBMED

(((((((((((preeclampsia) OR (pre-eclampsia)) OR (eclampsia)) OR (gestational hypertension)) OR (white coat hypertension)) OR (severe preeclampsia)) OR (late preeclampsia)) OR (early preeclampsia)) OR (pregnancy-induced hypertension)) OR (hypertensive syndromes of pregnancy)) AND ((((((((((((((metabolomic*) OR (metabonomic*)) OR (metabolit*)) OR (HNMR)) OR (proton NMR)) OR (proton nuclear magnetic resonance)) OR (liquid chromatogra*)) OR (gas chromatogra*)) OR (UPLC)) OR (HPLC)) OR (high pressure liquid chromatograph*)) OR (ultra-performance liquid chromatograph*)) OR (ultra performance liquid chromatograph*)) OR (lipidomic*)) OR (mass spectrometr*))) AND (((screen*) OR (predict*)) OR (profil*)))

EMBASE

('preeclampsia' OR 'pre-eclampsia' OR 'eclampsia' OR 'gestational hypertension' OR 'white coat hypertension' OR 'severe preeclampsia' OR 'late preeclampsia' OR 'early preeclampsia' OR 'pregnancy-induced hypertension' OR 'hypertensive syndromes of pregnancy') AND ('metabolomic*' OR 'metabonomic*' OR 'metabolit*' OR 'HNMR' OR 'proton NMR' OR 'proton nuclear magnetic resonance' OR 'liquid chromatogra*' OR 'gas chromatogra*' OR 'UPLC' OR 'HPLC' OR 'high pressure liquid chromatograph*' OR 'ultra-performance liquid chromatograph*' OR 'ultra performance liquid chromatograph*' OR 'lipidomic*' OR 'mass spectrometr*') AND ('screen*' OR 'predict*' OR 'profil*').

SCOPUS

((TITLE-ABS-KEY (screen*) OR TITLE-ABS-KEY (predict*) OR TITLE-ABS-KEY (profil*))) AND ((TITLE-ABS-KEY (preeclampsia) OR TITLE-ABS-KEY (pre-eclampsia) OR TITLE-ABS-KEY (eclampsia) OR TITLE-ABS-KEY (gestational AND hypertension) OR TITLE-ABS-KEY (white AND coat AND hypertension) OR TITLE-ABS-KEY (severe AND preeclampsia) OR TITLE-ABS-KEY (late AND preeclampsia) OR TITLE-ABS-KEY (early AND preeclampsia) OR TITLE-ABS-KEY (pregnancy- AND induced AND hypertension) OR TITLE-ABS-KEY (hypertensive AND syndromes AND of AND pregnancy))) AND ((TITLE-ABS-KEY (metabolomic) OR TITLE-ABS-KEY (metabonomic*) OR TITLE-ABS-KEY (metabolit*) OR TITLE-ABS-KEY (hnmr) OR TITLE-ABS-KEY (proton AND nmr) OR TITLE-ABS-KEY (proton AND nuclear AND magnetic AND resonance) OR TITLE-ABS-KEY (liquid AND chromatogra*) OR TITLE-ABS-KEY (gas AND chromatogra*) OR TITLE-ABS-KEY (uplc) OR TITLE-ABS-KEY (hplc) OR TITLE-ABS-KEY (high AND pressure AND liquid AND chromatograph*) OR TITLE-ABS-KEY (ultra-performance AND liquid AND chromatograph*) OR TITLE-ABS-KEY (ultra AND performance AND liquid AND chromatograph*) OR TITLE-ABS-KEY (lipidomic*) OR TITLE-ABS-KEY (mass AND spectrometr*)))

Web of Knowledge

#1

For peer review only

((((((((ALL=(preeclampsia)) OR ALL=(pre-eclampsia)) OR ALL=(eclampsia)) OR ALL=(gestational hypertension)) OR ALL=(white coat hypertension)) OR ALL=(severe preeclampsia)) OR ALL=(late preeclampsia)) OR ALL=(early preeclampsia)) OR ALL=(pregnancy- induced hypertension)) OR ALL=(hypertensive syndromes of pregnancy)

#2

(((((((((((((ALL=(metabolomic*)) OR ALL=(metabonomic*)) OR ALL=(metabolit*)) OR ALL=(HNMR)) OR ALL=(proton NMR)) OR ALL=(proton nuclear magnetic resonance)) OR ALL=(liquid chromatogra*)) OR ALL=(gas chromatogra*)) OR ALL=(UPLC)) OR ALL=(HPLC)) OR ALL=(high pressure liquid chromatograph*)) OR ALL=(ultra-performance liquid chromatograph*)) OR ALL=(ultra performance liquid chromatograph*)) OR ALL=(lipidomic*)) OR ALL=(mass spectrometr*)

#3

((ALL=(screen*)) OR ALL=(predict*)) OR ALL=(profil*)

#4

#1 AND #2 AND #3

LILACS

('preeclampsia' OR 'pre-eclampsia' OR 'eclampsia' OR 'gestational hypertension' OR 'white coat hypertension' OR 'severe preeclampsia' OR 'late preeclampsia' OR 'early preeclampsia' OR 'pregnancy-induced hypertension' OR 'hypertensive syndromes of pregnancy') AND ('metabolomic*' OR 'metabonomic*' OR 'metabolit*' OR 'HNMR' OR 'proton NMR' OR 'proton nuclear magnetic resonance' OR 'liquid chromatogra*' OR 'gas chromatogra*' OR 'UPLC' OR 'HPLC' OR 'high pressure liquid chromatograph*' OR 'ultra-performance liquid chromatograph*' OR 'ultra performance liquid chromatograph*' OR 'lipidomic*' OR 'mass spectrometr*') AND ('screen*' OR 'predict*' OR 'profil*')

SciELO

('preeclampsia' OR 'pre-eclampsia' OR 'eclampsia' OR 'gestational hypertension' OR 'white coat hypertension' OR 'severe preeclampsia' OR 'late preeclampsia' OR 'early preeclampsia' OR 'pregnancy-induced hypertension' OR 'hypertensive syndromes of pregnancy') AND ('metabolomic*' OR 'metabonomic*' OR 'metabolit*' OR 'HNMR' OR 'proton NMR' OR 'proton nuclear magnetic resonance' OR 'liquid chromatogra*' OR 'gas chromatogra*' OR 'UPLC' OR 'HPLC' OR 'high pressure liquid chromatograph*' OR 'ultra-performance liquid chromatograph*' OR 'ultra performance liquid chromatograph*' OR 'lipidomic*' OR 'mass spectrometr*') AND ('screen*' OR 'predict*' OR 'profil*')

DARE and HTA

('preeclampsia' OR 'pre-eclampsia' OR 'eclampsia' OR 'gestational hypertension' OR 'white coat hypertension' OR 'severe preeclampsia' OR 'late preeclampsia' OR 'early preeclampsia' OR 'pregnancy-induced hypertension' OR 'hypertensive syndromes of pregnancy') AND ('metabolomic*' OR 'metabonomic*' OR 'metabolit*' OR 'HNMR' OR 'proton NMR' OR 'proton nuclear magnetic resonance' OR 'liquid chromatogra*' OR 'gas chromatogra*' OR 'UPLC' OR 'HPLC' OR 'high pressure liquid chromatograph*'

For peer review only

OR ‘ultra-performance liquid chromatograph*’ OR ‘ultra performance liquid chromatograph*’ OR ‘lipidomic*’ OR ‘mass spectrometr*’) AND (‘screen*’ OR ‘predict*’ OR ‘profil*’) IN DARE, HTA

For peer review only

Supplementary Material 3 - Excluded studies and reasons for exclusion.

Authors	DOI	Reason for exclusion
Kenny LC et al, 2008	10.1177/1933719108316908	Cross-sectional design.
Aris A et al, 2009	10.1016/j.placenta.2009.01.003	Non metabolomics research.
Kenny LC et al, 2009		Conference abstract. Duplicated data. Please check Kenny LC et al (10.1161/HYPERTENSIONAHA.110.157297)
Baker AM et al, 2010	10.1210/jc.2010-0996	Duplicated data. Please check Woodham PC et al, 2011 (10.1161/HYPERTENSIONAHA.111.179069)
Bahado-Singh RO et al, 2012	10.3109/14767058.2012.680254	Duplicated data. Please check Bahado-Singh RO et al, 2015 (10.1016/j.ajog.2015.06.044)
Austdal M et al, 2013	10.1016/j.preghy.2013.04.030	Cross-sectional design.
Bahado-Singh RO et al, 2013	10.1016/j.ajog.2012.11.003	Duplicated data. Please check Bahado-Singh RO et al, 2017 (10.1080/14767058.2016.1185411)
Senyavina NV et al, 2013	10.1007/s10517-013-2225-y	Cross-sectional design.
Bodnar et al, 2014	10.1097/EDE.0000000000000039	Imputation of data obtained in a small subset to the whole sample.
Austdal M et al, 2014	10.1371/journal.pone.0091923	Duplicated data. Please check Austdal M et al, 2015 (10.3390/ijms160921520)
Austdal M et al, 2015	10.1016/j.placenta.2015.10.019	Cross-sectional design.
Austdal M et al, 2015	10.3390/ijms160921520	Comparison of preeclampsia patients with women with normal blood pressure and gestational hypertension together.
Riley J et al, 2016		Conference abstract. Duplicated data. Please check Odibo A et al 2011 (10.1002/pd.2822)
Chen T et al, 2017	10.1016/j.bbrc.2017.02.032	Cross-sectional design.
Kelly RS et al, 2017	10.1007/s11306-016-1149-8	Randomized clinical trial.
Powell KL et al, 2018	10.1016/j.placenta.2018.04.005	Cross-sectional design.
Naase et al, 2018	10.1093/humrep/33.Supplement_1.1	Only mass spectrometry data.
Kawasaki K et al, 2019	10.1161/HYPERTENSIONAHA.118.12389	Cross-sectional design.
Liu Y et al, 2019	10.1111/1440-1681.13095	Cross-sectional design.
Sander KN et al, 2019	10.1007/s11306-019-1600-8	Cross-sectional design.
Chaudhry SH et al, 2019	10.1186/s12884-019-2219-5	Non metabolomics research.
Lewandowska M et al, 2019	10.3390/nu11051028	No specific data on preeclampsia.

For peer review only

Vieira MC et al, 2019	10.1177/1933719119834079	Randomized clinical trial. Only mass spectrometry data.
Odenkirk MT et al, 2020	10.1039/x0xx00000x	Cross-sectional design.
Ferranti et al, 2020	10.1155/2020/1515321	Mention of altered metabolic pathways, but not metabolites.
Kelly C et al, 2020	10.3390/nu12072048	Non metabolomics research.
Kyozuka H et al, 2020	10.1038/s41598-020-70974-3	No specific data on preeclampsia.
McBride N et al, 2020	10.1186/s12916-020-01819-z	Randomized clinical trial.
Jääskeläinen T et al, 2021	10.1007/s11306-020-01752-5	No specific data on preeclampsia compared to normotensive women.
Rodriguez et al, 2021	10.1016/j.arcmed.2021.01.012	Cross-sectional design.
McBride N et al, 2021	10.3390/ metabo11080530	Mention of accuracy measures of a group of metabolites, and of metabolites combined with other maternal risk factors, but do not mention specific metabolites.
Kivelä J et al, 2021	10.1210/clinem/dgab475	Randomized clinical trial.
Zhang-Rutledge K et al, 2021	10.1016/j.ajog.2020.12.267	Only mass spectrometry data.
Wahab et al, 2022	10.3390/ metabo12010013	No specific data on preeclampsia compared to normotensive women.
Herlambang H et al, 2022	10.1186/s12884-021-04313-3	Cross-sectional design.
Liu H, et al	10.1007/s12011-021-02988-5	Cross-sectional design.

For peer review only

Supplementary Material 4 - Predictive metabolites of hypertensive disorders of pregnancy summarized according to their superclass, chemical class, subclass, and metabolic pathways.

Predictive metabolites	Chemical class	Chemical subclass	Metabolic pathway or process	Involved with
		Organic acids and derivatives		
N1, N12-diacetylspermine	Carboximidic acids and derivatives	Carboximidic acids	NA	Late onset preeclampsia[1]
Acetamide	Carboximidic acids and derivatives	Carboximidic acids	NA	Preeclampsia [2]
Acetate	Carboxylic acids and derivatives	Carboxylic acids	Glycolysis, gluconeogenesis; pyruvate, propanoate, taurine and hypotaurine metabolism	Preeclampsia [2,3]
3-methylhistidine	Carboxylic acids and derivatives	Amino acids, peptides, and analogues	Beta-alanine, histidine and methylhistidine metabolism	Preeclampsia [3]
Methylhistidine	Carboxylic acids and derivatives	Amino acids, peptides, and analogues	Histidine Metabolism	Late onset preeclampsia [4]
Alanine	Carboxylic acids and derivatives	Amino acids, peptides, and analogues	Glutathione metabolism, glycine and serine metabolism, tryptophan metabolism, urea cycle, glutamate metabolism, glucose-alanine cycle	Preeclampsia [5], early onset [5] preeclampsia
Betaine	Carboxylic acids and derivatives	Amino acids, peptides, and analogues	Betaine metabolism, glycine and serine metabolism, methionine metabolism	Preeclampsia [2]
Asymmetric dimethylarginine	Carboxylic acids and derivatives	Amino acids, peptides, and analogues	NA	Preeclampsia [6], early onset [7] preeclampsia
L-arginine	Carboxylic acids and derivatives	Amino acids, peptides, and analogues	Urea cycle, arginine and proline metabolism, glycine and serine metabolism, transcription/translation	Preeclampsia [6], early onset [7] preeclampsia
L-homoarginine	Carboxylic acids and derivatives	Amino acids, peptides, and analogues	NA	Early onset [7] preeclampsia

Creatinine	Carboxylic acids and derivatives	Amino acids, peptides, and analogues	Arginine and proline metabolism	Preeclampsia [2,3]
Glutamine	Carboxylic acids and derivatives	Amino acids, peptides, and analogues	Pyrimidine metabolism, glutamate metabolism, purine metabolism, transcription/translation, urea cycle	Preeclampsia [2,3]
Glutamate	Carboxylic acids and derivatives	Amino acids, peptides, and analogues	Alanine metabolism, histidine metabolism, ammonia recycling, cysteine metabolism, arachidonic acid metabolism	Preeclampsia [5], early onset [5] preeclampsia
4-hydroxyglutamate	Carboxylic acids and derivatives	Amino acids, peptides, and analogues	Arginine and proline metabolism	Preeclampsia [8]
Glycine	Carboxylic acids and derivatives	Amino acids, peptides, and analogues	Ammonia recycling, porphyrin metabolism, glutathione metabolism, glycine and serine metabolism	Preeclampsia [2]
Glycylglycine	Carboxylic acids and derivatives	Amino acids, peptides, and analogues	NA	Late onset [9] preeclampsia
Isoleucine	Carboxylic acids and derivatives	Amino acids, peptides, and analogues	Valine, leucine and isoleucine biosynthesis and degradation	Preeclampsia [2,3]
Asparagine	Carboxylic acids and derivatives	Amino acids, peptides, and analogues	Alanine, aspartate and glutamate metabolism; protein digestion and absorption	Preeclampsia [10]
Methionine	Carboxylic acids and derivatives	Amino acids, peptides, and analogues	Betaine metabolism, glycine and serine metabolism; spermidine and spermine biosynthesis	Preeclampsia [2]
N,N-Dimethylglycine	Carboxylic acids and derivatives	Amino acids, peptides, and analogues	Glycine, serine, and threonine metabolism	Preeclampsia [10]
Ornithine	Carboxylic acids and derivatives	Amino acids, peptides, and analogues	Urea cycle, arginine and proline metabolism; glycine and serine	Preeclampsia [2]

For peer review only

			metabolism; Spermidine and Spermine Biosynthesis	
Phenylacetylglutamine	Carboxylic acids and derivatives	Amino acids, peptides, and analogues	Phenylacetate metabolism, phenylalanine metabolism	Preeclampsia [3]
Phenylalanine	Carboxylic acids and derivatives	Amino acids, peptides, and analogues	Phenylalanine, tyrosine and tryptophan biosynthesis; phenylalanine metabolismo;	Preeclampsia [5], early onset [5] preeclampsia
Proline	Carboxylic acids and derivatives	Amino acids, peptides, and analogues	Arginine and proline metabolism	Preeclampsia [2]
Tyrosine	Carboxylic acids and derivatives	Amino acids, peptides, and analogues	Tyrosine metabolism, phenylalanine and tyrosine metabolism, catecholamine biosynthesis, transcription/translation	Preeclampsia [3]
Valine	Carboxylic acids and derivatives	Amino acids, peptides, and analogues	Valine, leucine and isoleucine biosynthesis and degradation; propanoate metabolism	Preeclampsia [2]
Formate	Carboxylic acids and derivatives	Carboxylic acids	Folate metabolism, pterine biosynthesis	Preeclampsia [3]
Fumarate	Carboxylic acids and derivatives	Dicarboxylic acids and derivatives	Phenylalanine and tyrosine metabolism, arginine and proline metabolism, aspartate metabolism, urea cycle, mitochondrial electron transport chain	Preeclampsia [3]
Succinate	Carboxylic acids and derivatives	Dicarboxylic acids and derivatives	Citrate cycle, oxidative phosphorylation; alanine, aspartate and glutamate metabolism; pyruvate metabolism; cAMP signalling pathway; GABAergic synapse; glucagon signalling pathway	Preeclampsia [3]
Cis-aconitate	Carboxylic acids and derivatives	Tricarboxylic acids and derivatives	Citric acid cycle	Preeclampsia [3]

For peer review only

Citrate	Carboxylic acids and derivatives	Tricarboxylic acids and derivatives	Citric acid cycle, transfer of acetyl groups into mitochondria	Preeclampsia [3], early onset [11] preeclampsia, late onset [4] preeclampsia
2-hydroxybutyrate	Hydroxy acids and derivatives	Alpha hydroxy acids and derivatives	Propanoate metabolism	Early onset [11] preeclampsia
2-ketoglutarate	Keto acids and derivatives	Gamma-keto acids and derivatives	Citric acid cycle, gluconeogenesis, oxidation of branched-chain fatty acids, urea cycle, branched-chain amino acids degradation	Preeclampsia [3]
2-oxovaleric acid	Keto acids and derivatives	Short-chain keto acids and derivatives	NA	Late onset [12] preeclampsia
Acetoacetic acid	Keto acids and derivatives	Short-chain keto acids and derivatives	Ketone body metabolism, fatty acid biosynthesis, branched-chain amino acids degradation, tyrosine metabolism, phenylalanine and tyrosine metabolism	Late onset [12] preeclampsia
Oxo-methylbutanoic acid	Keto acids and derivatives	Short-chain keto acids and derivatives	Cell signaling; valine, leucine and isoleucine Degradation	Late onset [12] preeclampsia
Pyruvate	Keto acids and derivatives	Alpha keto acids and derivatives	Urea Cycle, glucose-alanine cycle, glycine and serine Metabolism, pyruvate metabolism, alanine metabolism	Late onset [13] preeclampsia
Urea	Organic carbonic acids and derivatives	Ureas	Urea cycle; D-arginine and D-ornithine metabolism; arginine and proline metabolism	Late onset [4] preeclampsia
Taurine	Organic sulfonic acids and derivatives	Organosulfonic acids and derivatives	Taurine and hypotaurine metabolism	Early onset [9] preeclampsia
Indoxyl sulphate	Organic sulfuric acids and derivatives	Arylsulfates	NA	Preeclampsia [3]
p-cresol sulphate	Organic sulfuric acids and derivatives	Arylsulfates	NA	Preeclampsia [3]

For peer review only

Lipids and lipid-likes molecules				
3-hydroxyisovalerate	Fatty acyls	Fatty acids and conjugates	Lipid transport, metabolism, and peroxidation; fatty acid metabolism	Early onset [11] preeclampsia
Adipic acid	Fatty acyls	Fatty acids and conjugates	Lipid transport, metabolism, and peroxidation; fatty acid metabolism	Late onset [12] preeclampsia
Docosapentaenoic acid	Fatty acyls	Fatty acids and conjugates	Lipid transport, metabolism, and peroxidation; fatty acid metabolism; cell signalling; alpha linolenic acid and linoleic acid metabolism	Preeclampsia [14]
Docosahexaenoic acid	Fatty acyls	Fatty acids and conjugates	Lipid transport, metabolism, and peroxidation; fatty acid metabolism; cell signalling; alpha linolenic acid and linoleic acid metabolism	Preeclampsia [14], Late onset [12] preeclampsia
Docosatriynoic acid	Fatty acyls	Fatty acids and conjugates	Lipid transport, metabolism, and peroxidation; fatty acid metabolism; cell signalling	Late onset [12] preeclampsia
Eicosatrienoic acid	Fatty acyls	Fatty acids and conjugates	Lipid transport, metabolism, and peroxidation; fatty acid metabolism; cell signalling	Preeclampsia [14]
Eicosatetraenoic acid	Fatty acyls	Fatty acids and conjugates	Lipid transport, metabolism, and peroxidation; fatty acid metabolism; cell signalling; alpha linolenic acid and linoleic acid metabolism.	Preeclampsia [14]
Eicosapentaenoic acid	Fatty acyls	Fatty acids and conjugates	Lipid transport, metabolism, and peroxidation; fatty acid metabolism; cell	Preeclampsia [14]

For peer review only

			signalling; alpha linolenic acid and linoleic acid metabolism.	
Methylglutaric acid	Fatty acyls	Fatty acid and conjugates	Lipid transport, metabolism, and peroxidation; fatty acid metabolism; cell signalling	Late onset [12] preeclampsia
Oleic acid	Fatty acyls	Fatty acids and conjugates	Fatty acid biosynthesis, biosynthesis of unsaturated fatty acids	Late onset [12] preeclampsia
Decanoylcarnitine	Fatty acyls	Fatty acid esters	Lipid transport and peroxidation; fatty acid metabolism; cell signaling	Early onset [15] preeclampsia, late onset [12] preeclampsia
Decenoylcarnitine	Fatty acyls	Fatty acid esters	NA	Early onset [15] preeclampsia, late onset preeclampsia
Lauroylcarnitine	Fatty acyls	Fatty acid esters	Lipid transport, metabolism, and peroxidation; fatty acid metabolism; cell signalling	Early onset [15] preeclampsia
Hexadecenoylcarnitine	Fatty acyls	Fatty acid esters	Lipid transport, metabolism, and peroxidation; fatty acid metabolism; cell signalling	Early onset [15] preeclampsia
Hexanoylcarnitine	Fatty acyls	Fatty acid esters	Lipid transport, metabolism, and peroxidation; fatty acid metabolism; cell signalling	Early onset [15] preeclampsia, late onset [15] preeclampsia
Hydroxyhexanoylcarnitine	Fatty acyls	Fatty acid esters	Lipid transport, metabolism, and peroxidation; fatty acid metabolism; cell signalling	Preeclampsia [5], early onset [5] preeclampsia
Isobutyrylcarnitine	Fatty acyls	Fatty acid esters	Lipid transport, metabolism, and peroxidation; fatty acid metabolism; cell signalling	Early onset [15] preeclampsia
Octanoylcarnitine	Fatty acyls	Fatty acid esters	Mitochondrial beta-oxidation of short chain saturated fatty acids	Early onset [15] preeclampsia, late onset [15] preeclampsia

For peer review only

Octenoylcarnitine	Fatty acyls	Fatty acid esters	Lipid transport, metabolism, and peroxidation; fatty acid metabolism; cell signalling	Early onset [15] preeclampsia, late onset [15] preeclampsia
Oleylcarnitine	Fatty acyls	Fatty acid esters	NA	Late onset [15] preeclampsia
Palmitoylcarnitine	Fatty acyls	Fatty acid esters	Fatty acid metabolism	Late onset [15] preeclampsia
Isoprostane 8-epi-prostaglandin F2α	Fatty acyls	Eicosanoids	Arachidonic acid metabolism	Preeclampsia [16]
(+/-) 5-iPF2α-VI	Fatty acyls	Eicosanoids	Lipid peroxidation	Preeclampsia [14]
(+/-) 5-iPF2α-VI-(d11)	Fatty acyls	Eicosanoids	Lipid peroxidation	Preeclampsia [14]
15-epilipoxin-A4	Fatty acyls	Eicosanoids	Lipid transport, metabolism, and peroxidation; fatty acid metabolism; cell signalling	Preeclampsia [17]
Alfa-linolenic acid	Fatty acyls	Lineolic acids and derivatives	Lipid transport, metabolism, and peroxidation; fatty acid metabolism; cell signalling; alpha linolenic acid and linoleic acid metabolism.	Preeclampsia [14]
Stearidonic acid	Fatty acyls	Lineolic acids and derivatives	Lipid transport, metabolism, and peroxidation; fatty acid metabolism; cell signalling; alpha linolenic acid and linoleic acid metabolism.	Preeclampsia [14]
Di-(octadecadienoyl)-sn-glycerol	Glycerophospholipids	Glycerophosphates	Lipid transport, metabolism, and peroxidation; fatty acid metabolism; cell signalling	Late onset [12] preeclampsia
Heptadecanoyl-2-hydroxy-sn-glycero-3-phosphocholine	Glycerophospholipids	Glycerophosphocholines	Lipid transport, metabolism, and peroxidation; fatty acid metabolism; cell signalling; phospholipid and glycerophospholipid metabolism.	Preeclampsia [18]
Dilinoleoyl-glycerol	Glycerophospholipids	Glycerophosphocholines	NA	Preeclampsia [18]

For peer review only - http://bmjopen.bmj.com/site/about/guidelines.xhtml

LysoPE C20:0	Glycerophospholipids	Glycerophosphoethanolamines	Lipid peroxidation and metabolism; glycerophospholipid metabolism; fatty acid metabolism cell signalling.	Preeclampsia [19]
Neomenthol-glucuronide	Prenol lipids	Terpene glycosides	Lipid peroxidation and metabolism; glycerophospholipid metabolism; fatty acid metabolism cell signalling.	Gestational hypertension [10]
Sphinganine 1-phosphate	Sphingolipids	Phosphosphingolipids	Sphingolipid metabolism, neuroactive ligand-receptor interaction	Late onset [12] preeclampsia
Sphingomyelin 16:0	Sphingolipids	Phosphosphingolipids	Lipid transport, metabolism, and peroxidation; fatty acid metabolism; cell signalling; apoptosis	Preeclampsia [20]
Sphingomyelin 18:0	Sphingolipids	Phosphosphingolipids	Phospholipid metabolism; lipid transport, metabolism, and peroxidation; fatty acid metabolism; cell signalling; apoptosis	Preeclampsia [20]
Sphingosine 1-phosphate	Sphingolipids	Phosphosphingolipids	Sphingolipid metabolism; lipid transport, metabolism, and peroxidation; fatty acid metabolism; cell signalling	Late onset [12] preeclampsia
Ceramide 14:0	Sphingolipids	Ceramides	NA	Preeclampsia [20]
Ceramide 24:0	Sphingolipids	Ceramides	NA	Preeclampsia [20]
Ceramide(d18:1/25:0)	Sphingolipids	Ceramides	NA	Preeclampsia [21]
Vitamin D	Steroids and steroids derivatives	Vitamin D and derivatives	Lipid transport, metabolism, and peroxidation; fatty acid metabolism; cell signalling	Preeclampsia [22], late onset [12] preeclampsia, gestational hypertension [23]
Bolasterone	Steroids and steroids derivatives	Androstan steroids	Lipid transport, metabolism, and peroxidation; fatty acid metabolism; cell signalling	Preeclampsia [10]
Organoheterocyclic compounds				

For peer review only - http://bmjopen.bmj.com/site/about/guidelines.xhtml

Caffeine	Imidazopyrimidines	Purines and purine derivatives	Caffeine metabolism	Early onset [24] preeclampsia
Paraxanthine	Imidazopyrimidines	Purines and purine derivatives	Caffeine Metabolism	Early onset [24] preeclampsia
5-Hydroxytryptophan	Indoles and derivatives	Tryptamines and derivatives	Tryptophan metabolism	Late onset [12] preeclampsia
Serotonin	Indoles and derivatives	Tryptamines and derivatives	Tryptophan metabolism; nicotine action pathway	Late onset [4] preeclampsia
Gama-butyrolactone	Lactones	Gamma butyrolactones	NA	Late onset [12] preeclampsia
N-methyl-2-pyridone-5-carboxamide	Pyridines and derivatives	Pyridinecarboxylic acids and derivatives	Nicotinate and Nicotinamide Metabolism	Preeclampsia [3]
N-methylnicotinamide	Pyridines and derivatives	Pyridinecarboxylic acids and derivatives	NA	Preeclampsia [3]
Oxolan-3-one	Dihydrofurans	Furanones	NA	Late onset [12] preeclampsia
		Organic oxygen compounds		
4-deoxyerythronic acid	Organooxygen compounds	Carbohydrates and carbohydrate conjugates	NA	Preeclampsia [3]
Galactose	Organooxygen compounds	Carbohydrates and carbohydrate conjugates	Galactose metabolism, nucleotide sugars metabolismo, lactose degradation	Preeclampsia [3]
Glycerol	Organooxygen compounds	Carbohydrates and carbohydrate conjugates	Glycerolipid metabolism, galactose metabolism, glycerolipid metabolism	Early onset [11] preeclampsia
Lactose	Organooxygen compounds	Carbohydrates and carbohydrate conjugates	Galactose metabolism, Phosphotransferase system, carbohydrate digestion and absorption	Preeclampsia [3]
Sucrose	Organooxygen compounds	Carbohydrates and carbohydrate conjugates	Starch and sucrose metabolism, galactose metabolism	Preeclampsia [3]
Acetone	Organooxygen compounds	Carbonyl compounds	Ketone body metabolism	Early onset [11] preeclampsia, late onset [4] preeclampsia
		Organic nitrogen compounds		
Scyllo-inositol	Organonitrogen compounds	Alcohols and polyols	Inositol phosphate metabolism	Preeclampsia [3]

For peer review only

Propylene glycol	Organooxygen compounds	Alcohols and polyols	Pyruvate metabolism	Late onset [4] preeclampsia
Propane-1,3-diol	Organooxygen compounds	Alcohols and polyols	Glycerolipid metabolism	Preeclampsia [19]
Putrescine	Organonitrogen compounds	Amines	Methionine metabolism, spermidine and spermine biosynthesis	Late onset [4] preeclampsia
Trimethylamine	Organonitrogen compounds	Amines	Methane and carbon metabolisms.	Preeclampsia [2,10]
Carnitine	Organonitrogen compounds	Quaternary ammonium salts	Beta oxidation of very long chain and branched chain fatty acids, mitochondrial beta-oxidation of saturated fatty acids	Preeclampsia [3], late onset [4,13] preeclampsia
Linoleylcarnitine	Organonitrogen compounds	Quaternary ammonium salts	Lipid transport, metabolism, and peroxidation; fatty acid metabolism; cell signalling	Early onset [15] preeclampsia, late onset [15] preeclampsia
Stearoylcarnitine	Organonitrogen compounds	Quaternary ammonium salts	Mitochondrial beta-oxidation of long chain saturated fatty acids	Early onset [15] preeclampsia, late onset [15] preeclampsia
Tetradecenoylcarnitine	Organonitrogen compounds	Quaternary ammonium salts	Lipid transport, metabolism, and peroxidation; fatty acid metabolism; cell signalling	Early onset [15] preeclampsia, late onset [15] preeclampsia
		Benzenoids		
Bisphenol A	Benzene and substituted derivatives	Diphenylmethanes	Bisphenol degradation	Preeclampsia [25]
Hippurate	Benzene and substituted derivatives	Benzoic acids and derivatives	Phenylalanine metabolism	Preeclampsia [3]
4-OH-hippurate	Benzene and substituted derivatives	Benzoic acids and derivatives	NA	Preeclampsia [3]
Mono(2- ethyl-5-oxohexyl) phthalate	Benzene and substituted derivatives	Benzoic acids and derivatives	NA	Late onset [25] preeclampsia

For peer review only

Mono(2-ethyl-5-carboxypentyl) phthalate	Benzene and substituted derivatives	Benzoic acids and derivatives	NA	Late onset [25] preeclampsia
Mono(2-ethylhexyl) phthalate	Benzene and substituted derivatives	Benzoic acids and derivatives	NA	Preeclampsia [25]
Mono(3-carboxypropyl) phthalate	Benzene and substituted derivatives	Benzoic acids and derivatives	NA	Preeclampsia [25]
Mono-ethyl phthalate	Benzene and substituted derivatives	Benzoic acids and derivatives	NA	Preeclampsia [25], late onset [25] preeclampsia
Monoisobutyl phthalate	Benzene and substituted derivatives	Benzoic acids and derivatives	NA	Preeclampsia [25]
Mono-*n*-butyl phthalate	Benzene and substituted derivatives	Benzoic acids and derivatives	NA	Late onset [25] preeclampsia
Σ di(2-ethylhexyl) phthalate	Benzene and substituted derivatives	Benzoic acids and derivatives	NA	Preeclampsia [25], Late onset [25] preeclampsia
2,6-Di-tert-butyl-4-hydroxymethylphenol	Benzene and substituted derivatives	Phenylpropanes	NA	Gestational hypertension [10]
		Organohalogen compounds		
Perfluorohexane sulfonate	Alkyl halides	Alkyl fluorides	NA	Preeclampsia [26]
		Alkaloids and derivatives		
Trigonelline	Alkaloids derived from nicotinic acid	Pyridine alkaloids	Nicotinate and nicotinamide metabolism	Preeclampsia [3]
Pilocarpine	NA	NA	Biosynthesis of alkaloids derived from histidine and purine	Gestational hypertension [10]
		Phenylpropanoids and polyketides		
Cerasinone	Flavonoids	O-methylated flavonoids	NA	Preeclampsia [10]
		Organosulfur compounds		
Dimethyl sulfone	Sulfonyls	Sulfones	NA	Preeclampsia [2]

NA = not available

For peer review only

References

1 Gong S, Sovio U, Aye ILMH, *et al.* Placental polyamine metabolism differs by fetal sex, fetal growth restriction, and preeclampsia. *JCI Insight* 2018;**3**:1–15. doi:10.1172/jci.insight.120723

2 Kenny LC, Broadhurst DI, Hong W, *et al.* NMR Profiling in Early Pregnancy Reveals a Metabolomic Signature the Predicts the Subsequent Development Preeclampsia. *Reprod Sci* 2013;**20**:2641A. doi:10.1177/1933719113482088

3 Diaz SO, Barros AS, Goodfellow BJ, *et al.* Second trimester maternal urine for the diagnosis of trisomy 21 and prediction of poor pregnancy outcomes. *J Proteome Res* 2013;**12**:2946–57. doi:10.1021/pr4002355

4 Bahado-Singh R, Poon LC, Yilmaz A, *et al.* Integrated Proteomic and Metabolomic prediction of Term Preeclampsia. *Sci Rep* 2017;**7**:1–10. doi:10.1038/s41598-017-15882-9

5 Odibo AO, Goetzinger KR, Odibo L, *et al.* First-trimester prediction of preeclampsia using metabolomic biomarkers: a discovery phase study. *Prenat Diagn* 2011;**31**:990–4.

6 Rijvers CAH, Marzano S, Winkens B, *et al.* Early-pregnancy asymmetric dimethylarginine (ADMA) levels in women prone to develop recurrent hypertension. *Pregnancy Hypertens* 2013;**3**:118–23. doi:10.1016/j.preghy.2013.01.001

7 Khalil AA, Tsikas D, Akolekar R, *et al.* Asymmetric dimethylarginine, arginine and homoarginine at 11-13 weeks gestation and preeclampsia: A case-control study. *J Hum Hypertens* 2013;**27**:38–43. doi:10.1038/jhh.2011.109

8 Sovio U, McBride N, Wood AM, *et al.* 4-Hydroxyglutamate is a novel predictor of pre-eclampsia. *Int J Epidemiol* 2020;**49**:301–11. doi:10.1093/ije/dyz098

9 Kuc S, Koster MPH, Pennings JLA, *et al.* Metabolomics profiling for identification of novel potential markers in early prediction of preeclampsia. *PLoS One* 2014;**9**. doi:10.1371/journal.pone.0098540

10 Harville EW, Li Y-YY-Y, Pan K, *et al.* Untargeted analysis of first trimester serum to reveal biomarkers of pregnancy complications: a case-control discovery phase study. *Sci Rep* 2021;**11**:3468. doi:10.1038/s41598-021-82804-1

11 Bahado-Singh RO, Syngelaki A, Akolekar R, *et al.* Validation of metabolomic models for prediction of early-onset preeclampsia. *Am J Obstet Gynecol* 2015;**213**:530.e1-530.e10. doi:10.1016/j.ajog.2015.06.044

12 Kenny LC, Broadhurst DI, Dunn W, *et al.* Robust early pregnancy prediction of later preeclampsia using metabolomic biomarkers. *Hypertension* 2010;**56**:741–9. doi:10.1161/HYPERTENSIONAHA.110.157297

13 Bahado-Singh RO, Syngelaki A, Mandal R, *et al.* Metabolomic determination of pathogenesis of late-onset preeclampsia. *J Matern Neonatal Med* 2017;**30**:658–64. doi:10.1080/14767058.2016.1185411

14 Bilodeau JF, Qin Wei S, Larose J, *et al.* Plasma F2-isoprostane class VI isomers at 12-18 weeks of pregnancy are associated with later occurrence of preeclampsia. *Free Radic Biol Med* 2015;**85**:282–7. doi:10.1016/j.freeradbiomed.2015.05.012

For peer review only

15 Koster MPH, Vreeken RJ, Harms AC, *et al.* First-Trimester serum acylcarnitine levels to predict preeclampsia: A metabolomics approach. *Dis Markers* 2015;**2015**. doi:10.1155/2015/857108

16 Chappell L. A longitudinal study of biochemical variables in women at risk of preeclampsia. *Am J Obstet Gynecol* 2002;**187**:127–36. doi:10.1067/mob.2002.122969

17 Shanmugalingam R, Wang X, Motum P, *et al.* The 15-Epilipoxin-A4 Pathway with Prophylactic Aspirin in PreventingPreeclampsia: A Longitudinal Cohort Study. *J Clin Endocrinol Metab* 2020;**105**. doi:10.1210/clinem/dgaa642

18 Kenny LC, Thomas G, Poston L, *et al.* Prediction of preeclampsia risk in first time pregnant women: Metabolite biomarkers for a clinical test. *PLoS One* 2020;**15**:1–19. doi:10.1371/journal.pone.0244369

19 Lee SM, Kang Y, Lee EM, *et al.* Metabolomic biomarkers in midtrimester maternal plasma can accurately predict the development of preeclampsia. *Sci Rep* 2020;**10**:16142. doi:10.1038/s41598-020-72852-4

20 Dobierzewska A, Soman S, Illanes SE, *et al.* Plasma cross-gestational sphingolipidomic analyses reveal potential first trimester biomarkers of preeclampsia. *PLoS One* 2017;**12**:1–16. doi:10.1371/journal.pone.0175118

21 Huang Q, Hao S, You J, *et al.* Early-pregnancy prediction of risk for pre-eclampsia using maternal blood leptin/ceramide ratio: Discovery and confirmation. *BMJ Open* 2021;**11**. doi:10.1136/bmjopen-2021-050963

22 Woodham PC, Brittain JE, Baker AM, *et al.* Midgestation maternal serum 25-hydroxyvitamin D level and soluble fms-like tyrosine kinase 1/placental growth factor ratio as predictors of severe preeclampsia. *Hypertension* 2011;**58**:1120–5. doi:10.1161/HYPERTENSIONAHA.111.179069

23 Ates S, Sevket O, Ozcan P, *et al.* Vitamin D status in the first-trimester: Effects of vitamin D deficiency on pregnancy outcomes. *Afr Health Sci* 2016;**16**:36–43. doi:10.4314/ahs.v16i1.5

24 Eichelberger KY, Baker AM, Woodham PC, *et al.* Second-trimester maternal serum paraxanthine, CYP1A2 activity, and the risk of severe preeclampsia. *Obstet Gynecol* 2015;**126**:725–30. doi:10.1097/AOG.0000000000001041

25 Cantonwine DE, Meeker JD, Ferguson KK, *et al.* Urinary concentrations of bisphenol A and phthalate metabolites measured during pregnancy and risk of preeclampsia. *Environ Health Perspect* 2016;**124**:1651–5. doi:10.1289/EHP188

26 Rylander L, Lindh CH, Hansson SR, *et al.* Per- and Polyfluoroalkyl Substances in Early Pregnancy and Risk for Preeclampsia: A Case-Control Study in Southern Sweden. *Toxics* 2020;**8**. doi:10.3390/toxics8020043

For peer review only

Studies	Patient selection		Index test	
	Was a consecutive or random sample of patients enrolled?	Did the study avoid inappropriate exclusions?	Were the index test results interpreted without knowledge of the results of the reference standard?	If a threshold was used, was it pre-specified?
Chappell LC et al, 2002	Yes	No	Unclear	Yes
Powe et al, 2010	Yes	Yes	Unclear	Yes
Kenny LC et al, 2010	Yes	Yes	Yes	Yes
Odibo AO et al, 2011	Yes	No	Unclear	Yes
Woodham PC et al, 2011	Yes	Yes	Unclear	Yes
Rijvers CAH et al, 2013	Unclear	Unclear	Unclear	Yes
Khalil AA et al, 2013	Yes	Yes	Yes	Yes
Diaz SO et al, 2013	Yes	Unclear	Unclear	Yes
Kenny LC et al, 2013	Unclear	Unclear	Unclear	Yes
Kuc S et al, 2014	Yes	Yes	Unclear	Yes
Wetta LA et al, 2014	Yes	Yes	Unclear	Yes
Bahado-Singh RO et al, 2015	Yes	Yes	Unclear	Yes
Eichelberger KY et al, 2015	Yes	Yes	Yes	Yes
Koster MPH et al, 2015	Yes	Yes	Unclear	Yes
Bilodeau JF et al, 2015	Yes	Yes	Unclear	Yes
Ates S et al, 2016	Yes	Yes	Unclear	Yes
Cantonwine DE et al, 2016	Yes	Yes	Unclear	Yes
Kiely M et al, 2016	Yes	Yes	Yes	Yes
Bahado-Singh RO et al, 2017	Yes	Yes	Unclear	Yes
Bahado-Singh RO et al, 2017b	Yes	Yes	Unclear	Yes
Dobierzewska A et al, 2017	Yes	Yes	Unclear	Yes
Ye Y et al, 2017	Yes	No	Yes	Yes
Gong S et al, 2018	Yes	Yes	Unclear	Yes
Tamblyn JA et al, 2018	Yes	Yes	Yes	Yes
Sovio U et al, 2020	Yes	Yes	Unclear	Yes
Huo X et al, 2020	Yes	No	Unclear	Yes
Rylander L et al, 2020	Yes	Unclear	Unclear	Yes

For peer review only

Lee SM et al, 2020	Unclear	Unclear	Unclear	Yes
Kenny L et al, 2020	Yes	Yes	Yes	Yes
Shanmugalingam R et al, 2020	Yes	No	Unclear	Yes
Harville EW et al, 2021	Yes	Unclear	Unclear	Yes
Huang Q et al, 2021	Unclear	Yes	Unclear	Yes

For peer review only

Risk of bias				
Reference standard			Flow and timing	
Is the reference standard likely to correctly classify the target condition?	Were the reference standard results interpreted without knowledge of the results of the index test?	Was there an appropriate interval between index test and reference standard?	Did all patients receive the same reference standard?	Were all patients included in the analysis?
Yes	Yes	Yes	Yes	Yes
Yes	Unclear	Yes	Yes	Yes
Yes	Yes	Yes	Yes	Yes
Yes	Unclear	Yes	Yes	Yes
Yes	Unclear	Yes	Yes	Yes
Yes	Unclear	Yes	Yes	Yes
Yes	Unclear	Yes	Yes	Yes
Yes	Unclear	Unclear	Yes	Yes
Yes	Unclear	Yes	Yes	Yes
Yes	Unclear	Yes	Yes	Yes
Yes	Yes	Yes	Yes	Yes
Yes	Unclear	Yes	Yes	Yes
Yes	Unclear	Yes	Yes	Yes
Yes	Unclear	Yes	Yes	Yes
Yes	Unclear	Yes	Yes	Yes
Yes	Unclear	Yes	Yes	Yes
Yes	Unclear	Unclear	Yes	No
Yes	Yes	Yes	Yes	Yes
Yes	Unclear	Yes	Yes	Yes
Yes	Unclear	Yes	Yes	Yes
Yes	Unclear	Yes	Yes	Yes
Yes	Unclear	Yes	Yes	Yes
Yes	Unclear	Yes	Yes	Yes
Yes	Yes	Yes	Yes	Yes
Yes	Unclear	Yes	Yes	Yes
Yes	Unclear	Yes	Yes	Yes
Yes	Unclear	Yes	Yes	No

For peer review only

Yes	Unclear	Yes	Yes	Yes
Yes	Yes	Yes	Yes	Yes
Yes	Unclear	Yes	Yes	No
Unclear	Unclear	Yes	Yes	Yes
Yes	Unclear	Yes	Yes	Yes

For peer review only

Applicability concerns		
**Patient selection**	**Index test**	**Reference standard**
Are there concerns that the included patients do not match the review question?	Are there concerns that the index test, its conduct, or interpretation differ from the review question?	Are there concerns that the target condition as defined by the reference standard does not match the review
No	No	No
No	No	No
No	No	No
No	No	No
No	No	No
No	No	No
No	No	No
Yes	No	No
No	No	No
No	No	No
No	No	No
No	No	No
No	No	No
No	No	No
No	No	No
No	No	No
No	Yes	No
No	No	No
No	No	No
No	No	No
No	No	No
No	No	No
No	No	No
No	No	No
No	No	No
Yes	No	No
No	No	No

For peer review only

No	No	No
No	No	No
Yes	No	No
No	No	Yes
No	No	No

For peer review only
